# Recurrent Modification of a Conserved *Cis*-Regulatory Element Underlies Fruit Fly Pigmentation Diversity

**DOI:** 10.1371/journal.pgen.1003740

**Published:** 2013-08-29

**Authors:** William A. Rogers, Joseph R. Salomone, David J. Tacy, Eric M. Camino, Kristen A. Davis, Mark Rebeiz, Thomas M. Williams

**Affiliations:** 1 Department of Biology, University of Dayton, Dayton, Ohio, United States of America; 2 Department of Biological Sciences, University of Pittsburgh, Pittsburgh, Pennsylvania, United States of America; 3 Center for Tissue Regeneration and Engineering at Dayton, University of Dayton, Dayton, Ohio, United States of America; University of California Berkeley, United States of America

## Abstract

The development of morphological traits occurs through the collective action of networks of genes connected at the level of gene expression. As any node in a network may be a target of evolutionary change, the recurrent targeting of the same node would indicate that the path of evolution is biased for the relevant trait and network. Although examples of parallel evolution have implicated recurrent modification of the same gene and *cis*-regulatory element (CRE), little is known about the mutational and molecular paths of parallel CRE evolution. In *Drosophila melanogaster* fruit flies, the Bric-à-brac (Bab) transcription factors control the development of a suite of sexually dimorphic traits on the posterior abdomen. Female-specific Bab expression is regulated by the dimorphic element, a CRE that possesses direct inputs from body plan (ABD-B) and sex-determination (DSX) transcription factors. Here, we find that the recurrent evolutionary modification of this CRE underlies both intraspecific and interspecific variation in female pigmentation in the *melanogaster* species group. By reconstructing the sequence and regulatory activity of the ancestral *Drosophila melanogaster* dimorphic element, we demonstrate that a handful of mutations were sufficient to create independent CRE alleles with differing activities. Moreover, intraspecific and interspecific dimorphic element evolution proceeded with little to no alterations to the known body plan and sex-determination regulatory linkages. Collectively, our findings represent an example where the paths of evolution appear biased to a specific CRE, and drastic changes in function were accompanied by deep conservation of key regulatory linkages.

## Author Summary

Trait development occurs through networks of genes that are connected by interactions between transcription factor proteins and binding site sequences within *cis*-regulatory element (CRE) DNA sequences. These interactions enable CREs to function as switches that control the expression of a gene(s) they regulate. Little is known about the molecular paths by which CREs evolve. Here, we identify a CRE that has repeatedly been the target of mutations that generate diverse pigmentation phenotypes on the abdomen of *Drosophila melanogaster* and its close relatives. By reconstructing and testing the ancestral form of this enhancer *in vivo*, we demonstrate that individuals from widely distributed *Drosophila melanogaster* populations possess modified forms of this CRE. Interestingly, the majority of this divergence proceeded without modifying previously identified binding sites for body plan and sex determination transcription factors. This pattern of extreme functional divergence, with contrasting conservation of transcription factor inputs may reflect strong constraint against modifying regulatory sequences that are required for expression in multiple body regions through shared binding sites.

## Introduction

Recurrence is a widespread phenomenon in evolutionary biology [1], where similar derived traits have often been found to evolve in parallel. This theme of recurrence extends to the molecular level, as the same genes are often targeted by evolutionary change to generate convergent phenotypes [2]. Illustrative examples include *Pitx1* for pelvic reduction in stickleback fish [3], *Oca2* for cavefish albinism [4], *svb* for fruit fly larval trichome loss [5], *yellow* for fruit fly wing pigmentation spots [6], *Mc1r* for vertebrate melanism [7], [8], and *ATPα* for insect [9] and *RNASE1* for monkey dietary specializations [10]. These examples of mechanistically biased evolution include gene duplications [9], [10], amino acid altering mutations [4], [7]–[10], and mutations that modify gene regulatory sequences [6], [11], [12]. While the phenomenon of recurrent evolution of regulatory sequences is now well established, a mechanistic understanding of how transcriptional regulatory sequences change function is still in its infancy. Specifically, does bias in the path of evolutionary change extend to the level of individual protein-DNA interactions in the regulatory sequences that influence transcription?

Traits are generated during development through the combined activities of cooperating genes [13]–[15]. Most genes are composed of a coding sequence, and non-coding sequences that include one or more *cis*-regulatory elements (CREs) that control a gene's overall expression pattern [16]. CREs possess binding sites for numerous transcription factor proteins [17], where each unique transcription factor and binding site(s) interaction can be considered a “regulatory linkage”. The types of linkages and their organization form a “regulatory logic” that integrates the regulatory state of a cell, and thereby directs a spatial and temporal output pattern of gene expression [15]. For a given trait, the multitude of genes, their coding and non-coding regions, and CRE regulatory linkages present an abundance of mutational targets to alter the phenotype. Hence, it might be expected that the genetic path of evolution could proceed by many routes and resultantly, would appear unpredictable in retrospect. However, mutations that are pleiotropic often reduce fitness [18] and bear considerable deleterious effects [16]. As a result, evolution may more readily proceed by paths that minimize pleiotropy [19].

It is unclear whether and how pleiotropy constrains the path of regulatory logic evolution: the gain and loss of binding sites for transcription factors. Relatively few cases of CRE evolution have been characterized in sufficient detail [20]–[28], and often a connection remains to be made between the causative mutations and the molecular mechanisms of evolved activity [6], [29]–[38]. Furthermore, a small number of studies have investigated the pleiotropic consequences of a CRE's evolution. Thus, an important research goal is to advance a general understanding of the paths by which CRE function evolves. Extant CREs appear to be elegantly built with an intricate regulatory logic of transcription factor binding sites, and yet, when a CRE's function changes, how many steps does it take? Do the relevant mutations create or destroy binding sites for transcription factors that already interact with the CRE, or do they represent new factor inputs? If a model exists where independent paths of evolution can be traced in parallel, one could assess the general attributes of successful paths of CRE divergence. One suitable model is the sexually dimorphic abdominal pigmentation exhibited among species within the *Sophophora* subgenus of *Drosophila*, which includes the model organism species *Drosophila* (*D.*) *melanogaster*.

**Figure 1:**
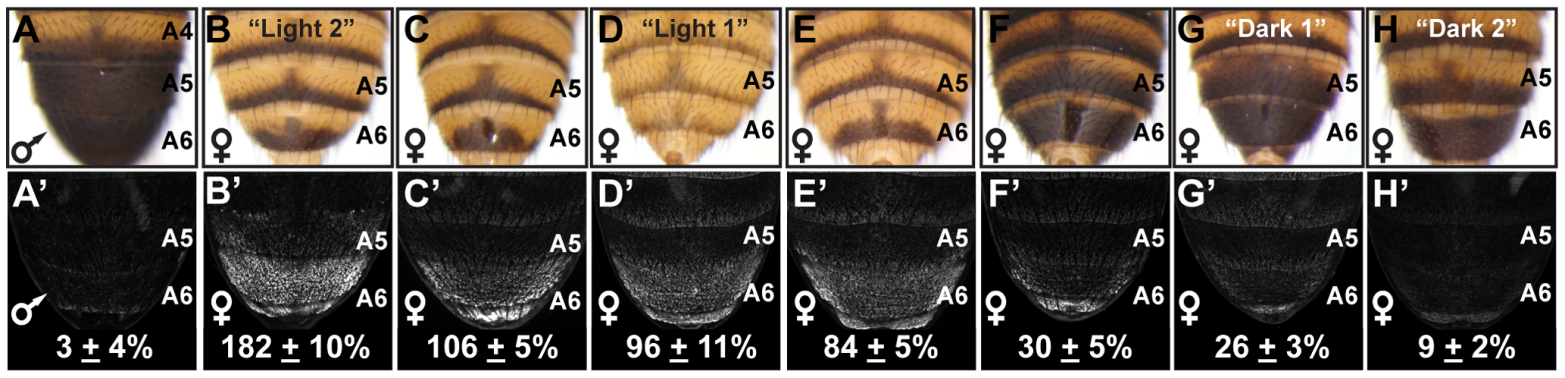
Abdomen pigmentation correlates with the regulatory activity of dimorphic element alleles. (A) The A5 and A6 segment dorsal tergites of *D. melanogaster* males are fully pigmented, (B–H) whereas the female A5 and A6 tergite pigmentation varies from “Light” to a male-like “Dark” phenotype. (A′–H′) GFP-reporter transgene activity was measured in transgenic pupae at 85 hours after puparium formation (hAPF) and activity measurements were represented as the % of the *D. melanogaster Canton^S^* allele female A6 mean ± SEM. (A′) The regulatory activity of a male *Canton^S^* pupae. The regulatory activity of alleles from the following locations were measured: (B′) Oaxaca, Mexico (called Light 2), (C′) Crete, Greece, (D′) Kuala Lumpur, Malaysia (called Light 1), (E′) Mumbai, India, (F′) Kisangani, Africa, (G′) Uganda, Africa (called Dark 1), and (H′) Bogota, Columbia (called Dark 2).

The fruit fly abdomen consists of ten abdominal segments (annotated A1–A10), the first seven of which are covered by dorsal cuticle plates (tergites). For *D. melanogaster*, tergite pigmentation is sexually dimorphic, where the male A5 and A6 tergites are completely pigmented (Figure 1A) and female pigmentation is typically restricted to a posterior stripe similar to that observed on the more anterior A2–A4 tergites of both sexes (Figure 1B). These sex-specific phenotypes are the outcomes of a regulatory network that includes prominent genes from the body plan and sex determination pathways. The HOX protein ABD-B is expressed in segments A5 and A6 of both sexes [39], [40], and positively activates a melanin synthesis enzyme that generates dark color [23]. While ABD-B provides body-plan positional information to activate pigmentation enzymes, their male-limited expression results from the sexually dimorphic expression of the tandem duplicate *bab1* and *bab2* genes (collectively *bab*, Figure S3A). These paralogous genes encode the transcription factors Bab1 and Bab2 (collectively Bab) that function as repressors of pigmentation development [41], [42]. In the pupal abdomen, both Bab1 [20] and Bab2 [42] are expressed in the A2–A7 segments of females, whereas male expression is limited to segments A2–A4.

Bab expression in female posterior abdominal segments is controlled by a CRE located in the first intron of *bab1* named the dimorphic element (Figure S3A). This CRE contains regulatory linkages with the Hox protein ABD-B and sex-specific DSX protein isoforms through its possession of multiple binding sites for these two transcription factors. Thus, the dimorphic element functions as a sexually dimorphic genetic switch controlling Bab expression. In males, ABD-B and DSX^M^ (male DSX isoform) binding to this CRE represses Bab expression in segments A5 and A6; whereas in females, ABD-B and DSX^F^ (female DSX isoform) binding activates Bab expression at increasing levels from the A5 segment to the more posterior A7 segment [20].

The *bab* genes have been implicated in both intraspecific and interspecific pigmentation evolution. Variation in female abdomen pigmentation exists among *D. melanogaster* populations [43]–[46] and in some cases this variation has been linked to genetic differences at the *bab* locus [47], [48]. Within the *Sophophora* subgenus of *Drosophila*, large-scale differences in pigmentation have been attributed to altered dimorphic element activity and consequent Bab expression [20]. Furthermore, male-specific pigmentation and underlying dimorphic Bab expression are inferred to be the derived state, evolving from an ancestor with sexually monomorphic Bab expression and pigmentation [23]. This ancestor possessed a CRE orthologous to the dimorphic element that drove Bab expression in the A7 and A8 segments (presumptive genitalia) of females [20], where it presumably regulated the development of other dimorphic traits [41], [42]. In the lineage of *D. melanogaster*, the dimorphic element was modified to drive female-specific expression in the more anterior A6 and A5 segments. This expanded Bab expression pattern was essential to limit full tergite pigmentation to the male A5 and A6 segments. Surprisingly, the ancestral dimorphic element was inferred to have possessed both the orthologous Dsx binding sites and 13 of the 14 Abd-B sites found in the *D. melanogaster* CRE. An amalgam of changes were introduced along an evolutionary path of greater than 30 million years to arrive at the derived activity; including Abd-B binding site number, Dsx site polarity, and the spacing between conserved binding sites [20]. Whether gains and losses of other regulatory linkages were a part of this transition remains unknown. Moreover, the simplicity and multiplicity of the mutations that occurred over this mesoevolutionary timescale [49], [50] inspired several questions: Do evolutionarily relevant mutations in the dimorphic element occur over microevolutionary time scales? Have orthologous dimorphic elements been repeatedly functionally modified? Do commonalities exist between independent cases of dimorphic element evolution?

Here, we implicate alterations in the *bab* dimorphic element as an underlying cause of the recurrently evolving diversity of female abdomen pigmentation at both the intraspecific and interspecific scales of comparison. Using this system to examine the evolution of regulatory logic along parallel paths, we characterized the mutational paths of dimorphic element divergence responsible for the diversification of intraspecific phenotypes using a gene reconstruction approach [51]. Inferring the ancestral dimorphic element sequence of extant *D. melanogaster* populations, we found that a small number of functionally-relevant mutations altered the ancestral CRE's regulatory activity to generate derived capabilities. Intriguingly, mutations largely avoided the ancestral ABD-B and DSX regulatory linkages, presumably to preserve the ancestral function of this CRE in the A7 segment and genitalia where it presides over other dimorphic aspects of abdominal development. While not definitive, these results can be viewed to support the notion that evolution can be biased to follow certain paths and such biases can pertain not only to certain genes in a network, or particular CREs, but that bias also permeates in how a CRE's encoded regulatory logic evolves.

## Results

### Allelic Variation in a Sexually Dimorphic *Cis*-Regulatory Element

Bab expression in the female A5 through A8 abdominal segments of *D. melanogaster* is driven by the dimorphic element. This regulatory activity evolved from an ancestral state limited to the female A7 and A8 segments since the most recent common ancestor of *D. melanogaster* and *D. willistoni*, species that diverged over 30 million years ago [20], [52]. It remained unknown whether the functional evolution of this CRE was limited to mesoevolutionary timescales, or whether recent transitions in activity occurred over microevolutionary timescales to diversify pigmentation patterns. Thus, we surveyed individuals from geographically diverse populations of *D. melanogaster* to identify those that differ in the extent of dimorphic abdominal pigmentation (Figure S1).

In contrast to the invariant male pigmentation phenotype (Figure S1 and Figure 1A), the extent of pigmentation varied greatly among the female A5 and A6 tergites (Figure S1, and Figure 1B–H). Phenotypes ranged from unpigmented tergites that bear only a posterior stripe of pigment (e.g. Figure 1B) to complete A6 pigmentation (Figure 1H), extending in one instance to the A5 tergite (Figure 1G). We suspected that these “Light” and “Dark” pigmentation phenotypes stem from differences in Bab expression, due to dimorphic element alleles with different regulatory activities. Indeed, sequencing of dimorphic element alleles isolated from twenty seven separate populations revealed many genetic differences (Figure S2). To test whether the observed genetic variation could cause divergent dimorphic element activities, we tested a subset of these alleles for the ability to drive *GFP* reporter gene expression (referred to as regulatory activity) in transgenic pupae. Relative to a previously characterized dimorphic element allele [20], we observed female regulatory activities ranging from 182±10% down to 9±2% (Figure 1B′–1H′), a 20 fold difference between the extreme alleles. Additionally, the level of dimorphic element activity generally correlated with the extent of female pigmentation (Figure 1), suggesting that this allelic variation is not coincidental but contributes to this variable phenotype.

### *bab* Genotypic Variation Underlies Pigmentation Variation

**Figure 2:**
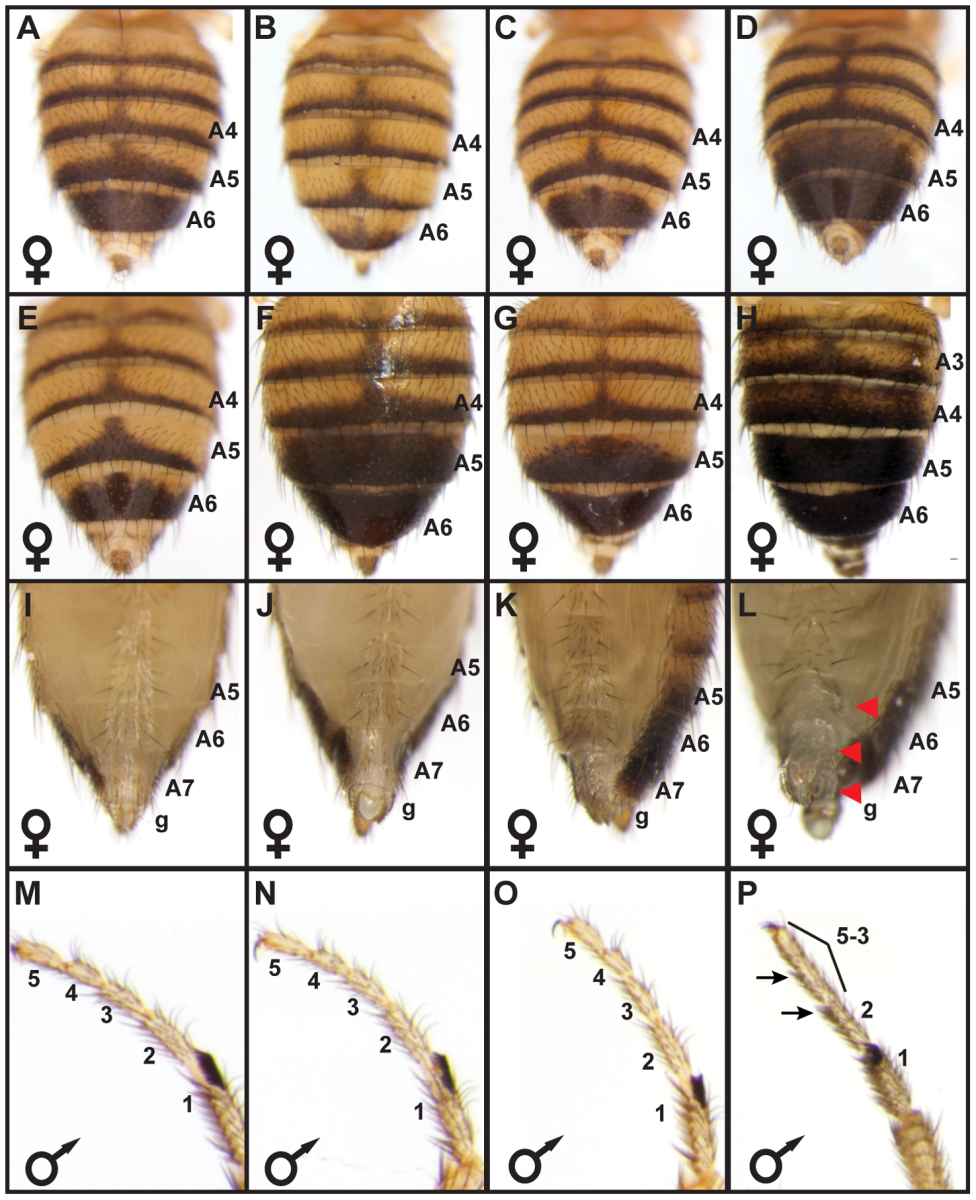
*bab locus* allelic variation underlies phenotypic variation. (A) The A5 and A6 tergite phenotype for F1 females were intermediate to those from the parental Light 1 and Dark 1 stocks. F2 females had pigmentation phenotypes that were (B) “Light”, (C) “Intermediate”, or (D) “Dark”. (E–P) Complementation tests for population stock *bab* loci with a *bab* locus null allele. (E) The Light 1 stock complemented the *bab* locus null allele with regards to abdomen tergite pigmentation, whereas the (F) Dark 1, and (G) Dark 2 stocks failed to complement the null allele in segments A5 and A6 but complemented the null allele for the A3 and A4 segments. Light 1, Dark 1, and Dark 2 stocks complemented the *bab* locus null allele for (I–K) posterior abdomen phenotypes and (M–O) for the development of the leg tarsal segments. Females with a homozygous *bab* locus null genotype displayed (F) ectopic pigmentation on segments A3 through A6, and (L) lacked bristles on the A6 and A7 ventral sternites and the genitalia (g) had altered bristles and morphology. (P) Individuals with a homozygous *bab* locus null genotype had tarsal segments 5, 4, and 3 fused, and altered bristle morphology on tarsal segments 2 and 3. Red arrowheads and black arrows respectively indicate the location abnormal posterior abdomen and tarsus features.

The correspondence between dimorphic element allele activity and pigmentation was suggestive of causation. Hence, we performed a series of genetic tests to further implicate the *bab* locus, and more importantly, the dimorphic element. First, we sought a genetic association between dimorphic element allele genotype and pigmentation phenotype. Males from a stock that produces a “Light” female pigmentation phenotype (called Light 1, Figure 1D and S1A) were separately crossed to females from two different population stocks that exhibit a “Dark” female pigmentation phenotype (called Dark 1, Figure 1G and S1AM; and called Dark 2, Figure 1H and S1AJ). F1 siblings were crossed to derive F2 progeny. The phenotypes of 102 F2 female progeny from the Light 1× Dark 1 cross were evaluated and 25, 54, and 23 respectively had Light, Intermediate, and Dark female pigmentation (Figure 2B–2D). This near 1∶2∶1 ratio (chi square *p* = 0.787) is indicative that this variable phenotype is largely due to a single semi-dominant gene. A subset of the F2 progeny were genotyped for a *BstXI* restriction fragment length polymorphism (RFLP) present in the Light 1 dimorphic element allele but not the Dark 1 allele. We found an invariant association between female progeny with the Light (Figure 2B) and Dark (Figure 2D) phenotypes respectively with homozygous genotypes for the Light 1 and Dark 1 dimorphic element alleles (Table S1). Moreover, females with an intermediate phenotype were heterozygous for this RFLP. We also found a similar genetic association for the F2 progeny hailing from the cross of Light 1 and Dark 2 (Table S2). After backcrossing the Dark 1 phenotype into the Light 1 genetic background for ten generations, we found that two independent backcross lines retained a Dark 1 *bab* locus haplotype (Figure S3F). Thus, the *bab* locus or something in close linkage causes this strain's Dark phenotype.

We performed genetic complementation tests to rule out the possibility that the genotype-phenotype associations were due to a variant linked to the *bab* locus. Light 1 and Dark 1 individuals were separately crossed to individuals with a *bab* locus null allele and pigmentation phenotypes were assessed for F1 progeny. Homozygous *bab* null mutants exhibit phenotypes present in both sexes, including fusion of the TS5, TS4, and TS3 leg tarsal segments and ectopic pigmentation on the A2–A4 segment tergites (Figure 2P and 2H), and several phenotypes limited to females. These female phenotypes include male-like pigmentation on the A5 and A6 tergites, posterior to anterior transformations of the A6, A7 and A8 (genitalia) segment morphologies [41], [42] (Figure 2H and 2L). While the *Light 1*, *Dark 1*, and *Dark 2 bab* loci complemented the *bab* null allele (*bab-*) with respect to the leg, A2–A4 tergite pigmentation, and female A7–A8 segment phenotypes, only the *Light 1* locus fully-complemented the *bab* null allele with respect to female A5 and A6 tergite pigmentation (compare Figure 2E to 2F and 2G). These same patterns of complementation and non-complementation were reproduced when Light and Dark lines were crossed to a deficiency line that included the entire *bab* locus (not shown), suggesting that the abdominal pigmentation phenotype is not due to mutations in the genetic background of the *bab* null allele, but rather allelic variation at *bab* between Light and Dark strains. Collectively, the most parsimonious conclusion from the genotype-phenotype association, genetic mapping, and complementation results is that the genetic basis for these Light and Dark female pigmentation phenotypes reside largely within the *bab* locus.

The failure of Dark lines to complement female A5/A6 phenotypes, whilst otherwise rescuing body-wide phenotypes of the *bab* null allele, suggested the existence of regulatory mutations underlying this phenotypic variation. Although a small number (6) of non-synonymous mutations were found that could potentially contribute to variation in abdominal pigmentation by altering Bab protein function (Figure S4), we pursued the hypothesis that relevant mutations would be located in the dimorphic element since this CRE controls Bab activity in the segments where *bab*-regulated phenotypes vary among the studied populations.

### Variation in Bab1 and Bab2 Expression

**Figure 3:**
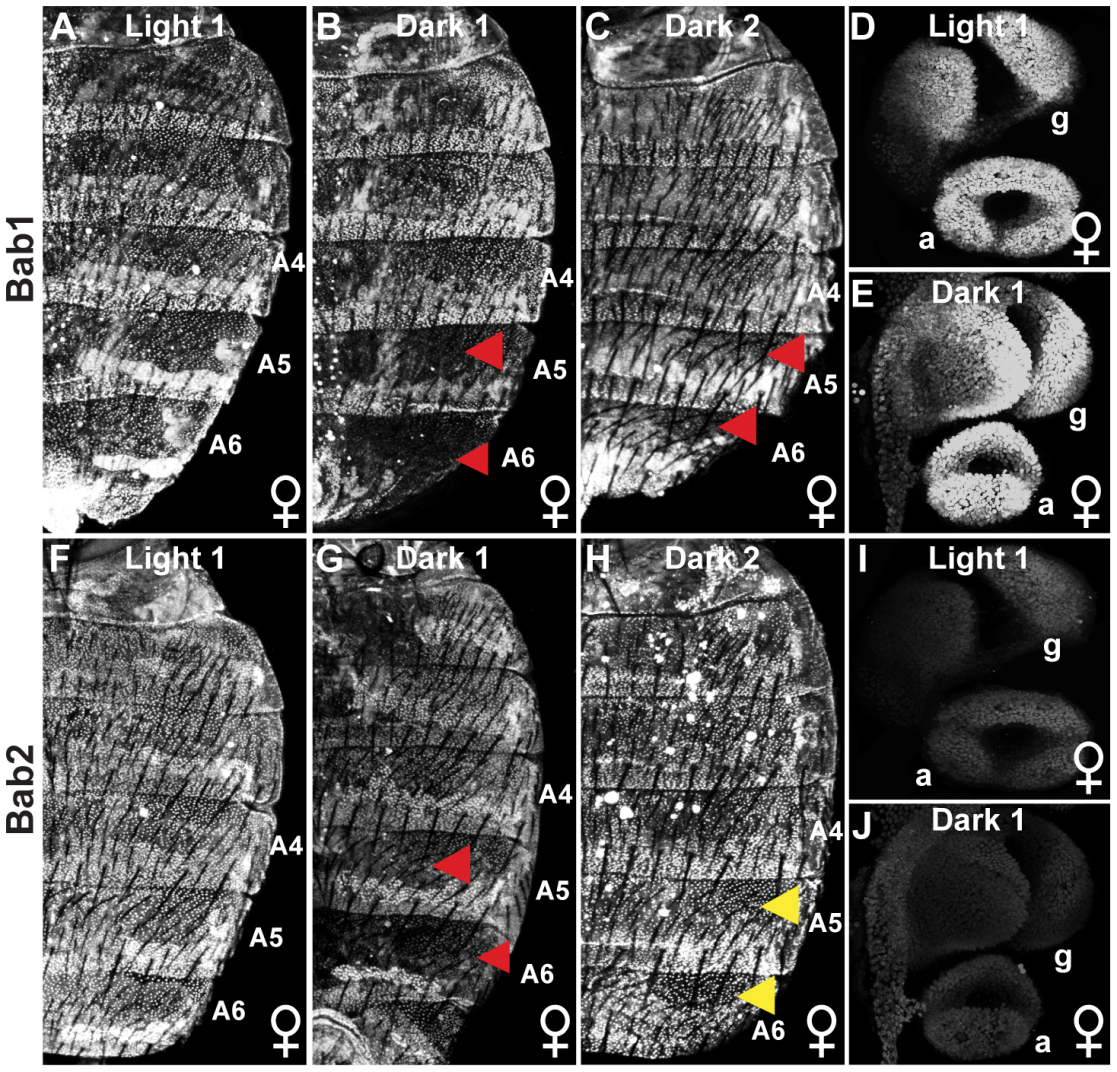
Population level differences in Bab paralog expression. (A–C) The expression of Bab1 in the dorsal abdomens of female pupae at 85 hAPF. (A) Light 1 females display uniform Bab1 expression throughout segments A2-A6, whereas expression is reduced in the A5 and A6 segments of (B) Dark 1 and (C) Dark 2 females. (D and E) Expression of Bab1 in the female genitalia (g) and analia (a) at 29 hAPF. (F–H) Bab2 expression in the dorsal abdomen of female pupae is at 85 hAPF. Bab2 expression is (F) uniform throughout the A2–A6 segments of Light 1 females, (G) reduced in the A5 and A6 segments of Dark 1 females, and (H) uniform throughout the A2–A6 of Dark 2 females. (I and J) Expression of Bab2 in the female genitalia (g) and analia (a) is at 29 hAPF. Red arrowheads indicate segments where expression is reduced compared to more anterior segments, whereas yellow arrowheads indicate the segments where Bab2 is expressed at a higher level than that observed for Bab1 for Dark 2 females.

Considering that the phenotypic effects of these naturally occurring dimorphic element alleles and pigmentation phenotypes were restricted to the A6 and to a lesser extent the A5 abdominal segment (Figure 1), we suspected that mutations in the dimorphic element could cause the observed differences in pigmentation. This hypothesis would be supported by differing levels and/or patterns of Bab expression in the pupal abdominal epidermis for females that develop different pigmentation phenotypes. Thus, we characterized the pattern of Bab expression in the abdominal epidermis at the end of pupal development when tergite pigmentation is being specified. If the regulatory activity for the dimorphic element alleles identified in reporter transgene assays (Figure 1) were indicative of the endogenous Bab expression, then Bab1 and Bab2 expression should be elevated in females with Light tergite pigmentation compared to those with Dark pigmentation. Consistent with this expectation, Bab1 and Bab2 were expressed robustly throughout the A2–A7 abdominal segments of Light 1 females (Figure 3A and 3F), while Bab1 and Bab2 expression were reduced in the A5 and A6 abdominal segments of Dark 1 female pupae (Figure 3B and 3G, red arrowheads). This reduction corresponds with the reduced regulatory activity of this strain's dimorphic element allele (Figure 1G′) and where the pigmentation develops on adult females (Figure 1G). Compared to Dark 1 females that possess expanded pigmentation on the A5 and A6 tergites, expanded pigmentation is limited to the A6 tergite of Dark 2 females (Figure 1H). Consistent with the Dark 2 phenotype, the expression of Bab1, but not Bab2, was reduced in the A6 segment and to a lesser extent the A5 segment (Figure 3C and 3H). These patterns of expression are consistent with the finding that the *bab1* null pigmentation phenotype is limited to the female A6 tergite, whereas a *bab2* null phenotype affects both the A6 and A5 tergite [41]. We also characterized Bab expression in the developing female genitalia and analia that respectively develop from the A8 and A9/A10 segments. In contrast to the reduced expression seen in the A5 and A6 segments epidermis of Dark 1 females, expression in these more posterior structures was comparable to that observed for Light 1 females (compare Figure 3D and 3I to 3E and 3J).

Collectively, the genetic and expression data strongly supports the conclusion that the conspicuous Light and Dark female pigmentation phenotypes are due, at least in part, to allelic differences in dimorphic element regulatory activity. We were interested in revealing how these modified regulatory activities evolved. To accomplish this, it was essential to know the ancestral sequence and regulatory state.

### Resurrection of an Ancestral Dimorphic Element

Ancestral Sequence Reconstruction (ASR) has been an effective approach to study the path of protein functional evolution [51], [53]. This approach, to our knowledge, had been used only sparingly to study CRE evolution in *Drosophila* [36], and primates [34], [54], presumably due to the fact that CRE sequences evolve at an accelerated rate compared to protein coding sequence [55]–[57], making reconstruction untenable when comparing organisms of distantly-related taxa. In the case here, the dimorphic element alleles share an ∼98% sequence identity (Figure S2) and a most recent common ancestor of extant *Drosophila melanogaster* populations that existed ∼60,000 years ago [58]. Hence, we suspected that the ancestral sequence for these populations could be reasonably inferred.

The dimorphic elements from 27 populations of *D. melanogaster* were sequenced and aligned to those from several outgroup species. From this alignment (Figure S2), we used the principle of parsimony to infer the nucleotide state at each position for the most recent common ancestor of the *D. melanogaster* populations, including 52 polymorphic sites; a sequence that was named the “Concestor element” [59]. For this sequence, the ancestral nucleotide states were unambiguous at 44 of the 52 sites. To test the robustness of this sequence's regulatory activity to the ambiguous eight sites, we tested alternate reconstructions that differed in the nucleotide states for these sites. We determined the regulatory activities for these reconstructions were comparable to that for the Concestor element (See “Evolutionary Robustness in Dimorphic Element Reconstruction”, Figure S2 and S6). Therefore, we sought to identify which of the 44 unambiguous derived mutations were responsible for the diverse regulatory activities possessed by the Light and Dark alleles. From this point forward, the Concestor element sequence was utilized for the ancestral sequence and regulatory activity state.

**Figure 4:**
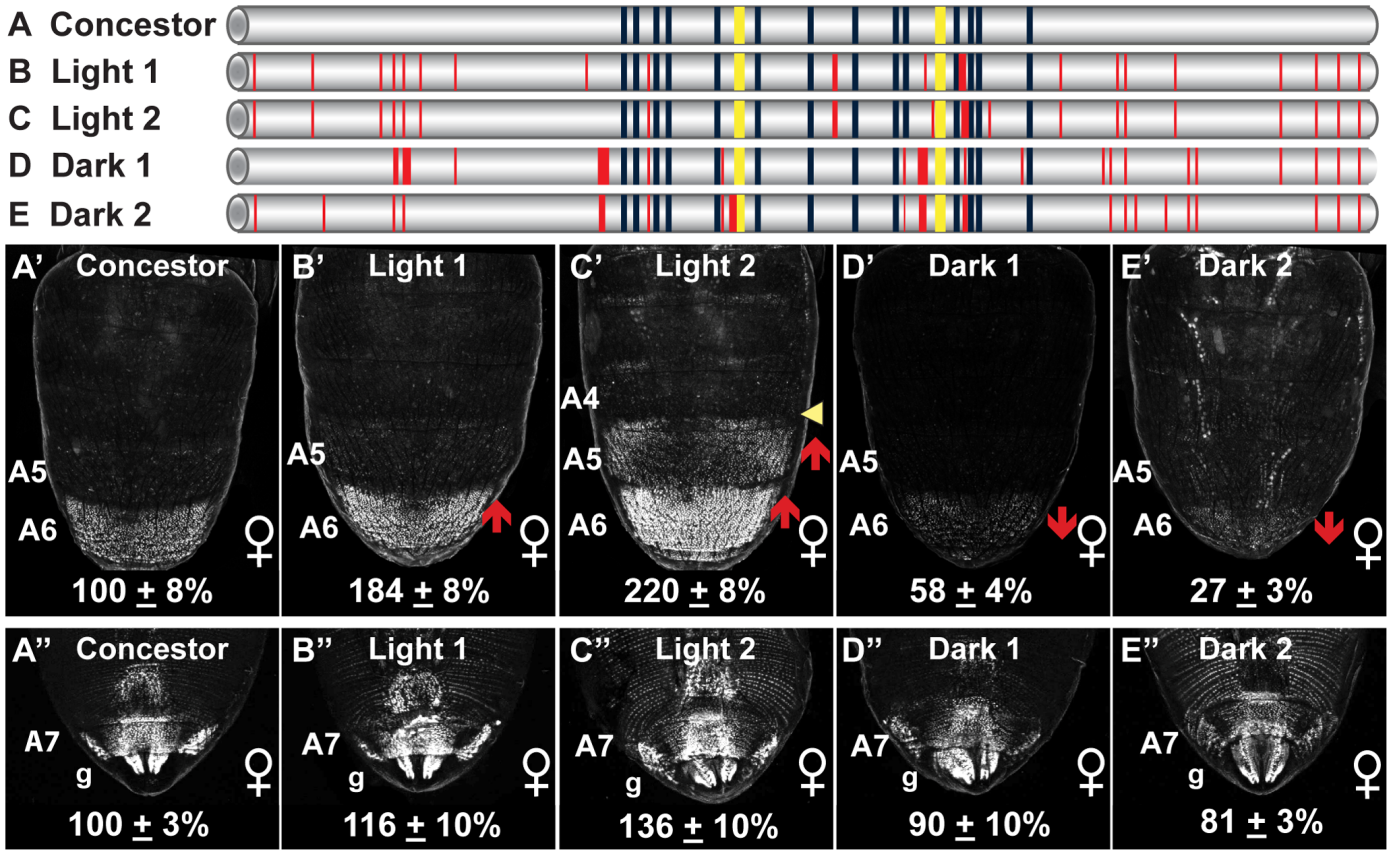
Dimorphic element alleles diverged from an ancestral state. (A–E) To scale representations of various dimorphic elements, including the (A, Concestor) inferred allele for the most recent common ancestor of extant *D. melanogaster* populations, and alleles from populations with Light (B, Light 1; C, Light 2) and Dark (D, Dark1; E, Dark 2) female pigmentation phenotypes. Dark blue and yellow rectangles respectively represent the fourteen ABD-B and two DSX binding sites. Thin and thick red lines respectively represent derived point and indel mutations. (A′–E′ and A″–E″) Comparison of GFP-reporter gene activities in female transgenic pupae was at 85 hAPF. Activity measurements are represented as the % of the *D. melanogaster* Concestor element female (A′–E′) A6 mean ± SEM and (A″–E″) A7 mean ± SEM. Red upward and downward arrows respectively indicate segments with increased and decreased regulatory activity. Yellow arrowhead indicates a region of expanded regulatory activity. Lowercase letter “g” indicates expression in the genitalia.

Several observations were made from a comparison of the Concestor element sequence to the dimorphic element alleles (Figure 4A–4E). First, the Concestor element possessed all of the ABD-B (14) and DSX (two) sites that were characterized for the *D. melanogaster Canton^S^* strain sequence [20]. Second, the Light 1, Light 2, Dark 1, and Dark 2 alleles respectively differ from the Concestor element by 20, 20, 22, and 20 derived mutations (Figure 4A–4E, vertical red lines), many of which are common to multiple alleles (Figure S2). Third, we observed an excess of nucleotide substitutions relative to indel mutations (Figure 4B–4E, thin versus thick red lines). Fourth, of the known binding sites, the only site gain/loss event caused by a derived mutation was ABD-B binding site 10, which was lost in the Dark 1 and Dark 2 alleles (caused by mutation “G”, Figure S2).

With the dimorphic element alleles differing in regulatory activity by up to 20 fold (Figure 1), we wanted to evaluate how these activities compare to that of the Concestor element. The regulatory activities were evaluated for the Light 1, Light 2, Dark 1, Dark 2, and Concestor element in a quantitative reporter transgene assay [60]. The Concestor element drove GFP expression in females throughout the epidermis of the A6 and A7 abdominal segments and the genitalia, and at a comparatively lower level in segment A5 (Figure 4A′ and 4A″). Compared to the Concestor element's regulatory activity, the Light 1 and 2 alleles' activities were increased in the A6 segment to 184±8% and 220±8% of concestor, respectively (Figure 4B′ and 4C′). Moreover, the Light 2 activity was increased in the A5 segment and expanded into the posterior region of segment A4. Conversely, compared to the Concestor element the A6 segment regulatory activities for the Dark 1 and Dark 2 alleles were reduced to 58±4% and 27±3% respectively (Figure 4D′ and 4E′). Additionally, the range of regulatory activities for the A6 segment was much greater than that for the A7 segment and genitalia (Figure 4A″–4E″). These results demonstrate that the ancestral dimorphic element for extant *D. melanogaster* populations drove low, modest, and high levels of *bab* expression respectively in the female A5, A6, and A7–A8 segments (Figure 4). This ancestral regulatory element was modified by mutation events resulting in derived alleles that include increased, expanded, and reduced activities in the relatively more anterior abdominal segments. We next sought to determine which of the derived mutations were functionally-relevant to the evolved regulatory activities.

### Derived Regulatory Activities Stem from Few Functionally-Relevant Mutations

In order to identify allele sub-regions that possess functionally-relevant mutations, we created a series of chimeric dimorphic elements and quantitatively compared their regulatory activities to that of the Concestor element. Each chimeric element was composed in part of Light 2 or Dark 1 allele sequence and the remaining sequence was from the Concestor element (Figure S5). For the chimeric elements containing some Light 2 dimorphic element sequence, most of this allele's derived activity was conveyed by the central “core” region that is occupied by the previously characterized binding sites for the ABD-B and DSX transcription factors. The Light 2 core flanked by Concestor element sequences had a regulatory activity of 239±5%, compared to 153±10% when the Concestor element core was within Light 2 flanks (Figure S5E and S5F). A similar outcome was found for the Dark 1 dimorphic element. When this allele's core sequence was flanked by Concestor element sequences, the chimeric element had an activity of 58±5%, whereas the reciprocal swap had no regulatory activity effect (106±2%; compare Figure S5J to S5K). Thus, for these two derived dimorphic element alleles, their unique regulatory activities principally stem from mutations in the core region.

**Figure 5:**
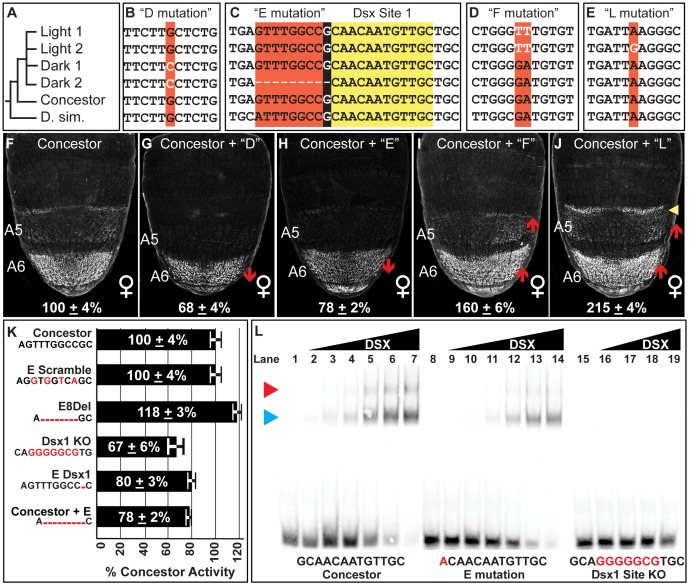
Functionally-relevant mutations in dimorphic element alleles. (A) Dimorphic element allele phylogeny, including the outgroup species *D. simulans* (D. sim.). Alignment of sequences encompassing the (B) “D” mutation, (C) “E” mutation, (D) “F” mutation, (E) and the “L” mutation. Black background color for the E mutation indicates the 1 base pair overlap for the derived deletion and the adjacent DSX binding site. (F–J) Comparison of GFP-reporter activity in female transgenic pupae at 85 hAPF, represented as the % of the *D. melanogaster* Concestor element female A6 mean ± SEM. Red upward and downward arrow respectively indicate segments with increased and decreased regulatory activity. Yellow arrowhead indicates expanded regulatory activity. Regulatory activities differing from the Concestor element due to the following derived mutations: (G) D mutation; (H) E mutation; (I) F mutation; and (J) L mutation. (K) Summary for the female A6 regulatory activities for modifications to the E mutation region. The Concestor element sequence is provided and the introduced modifications indicated by red bases. (L) Gel shift assays for annealed oligonucleotide probes containing the wild type (Concestor element, lanes 1–7), E mutation (lanes 8–14), and mutant (Dsx1 KO, lanes 15–19) Dsx1 binding site. The binding site sequences are included with mutant bases in red. For the Concestor element and E mutation probes, binding reactions used increasing amounts of the DSX protein (from left to right: 0 ng, 8 ng, 16 ng, 31 ng, 63 ng, 125 ng, 250 ng, and 500 ng). For the Dsx1 KO probe, binding reactions used the following amounts of protein (from left to right: 0 ng, 8 ng, 31 ng, 125 ng, 500 ng). Blue and red arrowheads point to the respective locations of single or pair of DSX monomers bound to the probe.

The Light 2 core region has seven derived mutations (referred to as the “C”, “F”, “H”, “J”, “K”, “L”, and “N” mutations, Figure S2), four of which also reside in the Light 1 core (C, F, K, and N). We individually substituted each of these mutations into the Concestor element in place of the ancestral nucleotide, and then tested whether these substitutions caused measurable effects on regulatory activity (Figure S6). Large mutational effects were only measured for the C, F, and L mutations; respectively these substitutions increased Concestor element activity to 140±6%, 160±6%, and 215±4% (Figure S6G, 5I and 5J). The C mutation is present in both the Light and Dark alleles being studied (Figure S2) and hence, cannot account for their differences in regulatory activity. When the F and L mutation were substituted together, regulatory activity was measured at a nearly additive 241±9% (Figure S6S). The Light 1 core differs from that of Light 2 by possessing a derived mutation, called “I” and lacking the L mutation. However, the I mutation had no affect on regulatory activity when it was substituted into the Concestor element (Figure S6M). Collectively, the derived regulatory activities of the Light 1 and 2 dimorphic element alleles both require the F mutation (Figure 5D and 5I), and the further increased and spatially expanded activity of the Light 2 allele requires the L mutation (Figure 5E and 5J).

The Dark 1 core sequence possesses six derived mutations that include: the “C”, “D”, and “G” mutations, each of which also reside in the Dark 2 allele, and the “M” mutation that is unique to the Dark 1. This core also has the “H” and “K” mutations that alter the C and T nucleotide expansions, though these occur in the Light alleles and were found not to cause significant regulatory effects (Figure S6L and S6O). Interestingly, the G mutation had no measurable effect on activity (Figure S6K), although it was the only one found to alter a known ADB-B site among the surveyed dimorphic element alleles. We conclude that the diversity of regulatory activities observed did not involve changes to the regulatory linkage between ABD-B and the dimorphic element. Testing the D and M mutations highlighted the functional relevance of the D mutation. When individually substituted into the concestor element, the D and M mutations respectively altered regulatory activity to 68±4% and 118±3% of the Concestor element (Figure S6H and S6Q). Though, when both the D and M mutations were substituted together, the net result was an activity of 68±3% (Figure S6T). Thus, the strong effect of the D mutation is epistatic to the moderate effect of M. As the complete Dark 1 core inserted between Concestor element flanking sequences had a regulatory activity of 58±5%, one or more core mutations must further reduce the Dark 1 allele's activity, either by increments below our capability to detect or through epistatic interactions. However, the D mutation is responsible for most of this allele's reduced regulatory activity (Figure 5B and 5G).

We next sought to find mutations underlying the further reduced regulatory activity of the Dark 2 allele. Like Dark 1, this allele possesses the D mutation, indicating the existence of an additional functionally-relevant mutation(s) in the core element. The only mutation unique to the Dark 2 core region was a 9 base pair deletion referred to as the “E” mutation. When the E mutation was substituted into the Concestor element, regulatory activity was reduced to 78±2% (Figure 5C and 5H). Moreover, the Dark 1 allele's activity was 58±4%. The addition of the E mutation to this allele lowered activity to 34±2%, near the 27±3% activity of the Dark 2 allele (Figure S6U). Collectively, the evolutionary paths of the Dark 1 and Dark 2 alleles include one shared functionally-relevant mutation and one that is unique to the Dark 2 allele.

### A Derived Mutation Disrupts a Conserved Transcription Factor Binding Site

The derived E mutation deletes nine base pairs, and the 9^th^ base pair is the first base pair for a DSX binding site (called Dsx1, Figure 5C), though this mutation creates a sequence that still matches the consensus motif for Dsx binding [61]. Mutational ablation of the Dsx1 site reduced the Concestor element's regulatory activity in the female A6 segment to 67±6% and raised activity in males from 6±2% to 73±5% (Figure S6Y-S6AA). This demonstrated that the Dsx1 site was necessary for robust female-specific regulatory activity. *A priori*, the E mutation could alter the quality of this Dsx1 site or reduce this allele's activity through other mechanisms. Such alternate mechanisms include: removing a binding site for a neighboring transcriptional activator, the formation of a novel binding site for a repressor, or by placing the Dsx1 site close to an adjacent transcription factor site. To obtain evidence supporting either of these mechanisms, we created and measured the regulatory activities for a set of modified Concestor elements with alterations to ancestral sequence at the E mutation region (Figure 5K). First, we introduced non-complementary transversions in the Concestor element at the 2^nd^, 4^th^, 6^th^, and 8^th^ base pairs of the E mutation (E Scramble). Here, the 9^th^ base pair and hence the consensus DSX binding site was not altered, but the other mutations would seemingly degrade an adjacent transcription factor binding site. This set of mutations did not alter Concestor element activity, indicating the E mutation did not delete a binding site adjacent to that of the DSX site. To disentangle regulatory effects due to the loss of sequence next to the Dsx1 site from loss of the 1^st^ base pair of the DSX site, we created two separate modifications to the Concestor element. One modification was a deletion of the first eight base pairs of the E mutation (called E8Del), and the second removed only the ninth base pair of the E mutation, which is the first of the Dsx1 site (called E Dsx1). Surprisingly, the 8 base pair deletion modestly increased Concestor activity to 118±3%, indicating that the E mutation's impact was not due to reduced spacing between the Dsx1 site and a more remote transcription factor binding site. The other modification, a deletion of only the 9^th^ base pair of the E mutation, reduced Concestor element activity to 80±3%. This reduction was nearly equal to that induced by the complete E mutation (Figure 5K). Collectively, these results demonstrate that the E mutation rendered the Dsx1 site less functional. One possible mechanism is that the E mutation made a derivative Dsx1 site with reduced affinity for the DSX protein. In order to validate this possibility, we compared the binding of the DSX DNA-binding domain (DBD) to the Concestor element, E mutant, and knockout (KO) Dsx1 site sequences in gel shift assays (Figure 5L). The Concestor element sequence was bound with high affinity by the DSX protein, and specifically as the KO site sequence is not readily bound (compare 5L lanes 1–7 to lanes 15–19). In comparison, DSX bound the site with the E mutation with reduced affinity compared to the wild type site (Figure 5L, lanes 8–14). A shift of the Concestor Dsx1 site was evident with as low as 16 ng of DSX protein, whereas binding of the E mutant site was not detected with this amount of DSX, but was with 32 ng (compare Figure 5L lane 3 to lanes 10 and 11). From these data, we estimate that the E mutation resulted in a Dsx1 site with ∼50% of the Concestor element site's affinity for the DSX protein.

Of the four prominent functionally-relevant mutations identified for the Light and Dark dimorphic element alleles (Figure 5), only one affects a known regulatory linkage. Specifically, the E mutation weakens the regulatory linkage between DSX and the dimorphic element by creating a lower affinity binding site. The D, F, and L mutations appear unremarkable compared to the other mutations that had no measureable regulatory effects (Figure S6). Moreover, the D, F, and L mutations caused regulatory effects comparable in magnitude to mutations implicated in the mesoevolutionary expansion of dimorphic element activity into the A6 and A5 segments [20]. Hence, it can be concluded that short mutational paths are sufficient to evolve pronounced alterations in this CRE's activity. This conclusion inspired the hypothesis that changes in female abdominal pigmentation may frequently occur through the alteration of the dimorphic element via similarly short paths.

### Correspondence between Dimorphic Element and Interspecific Pigmentation Evolution

**Figure 6:**
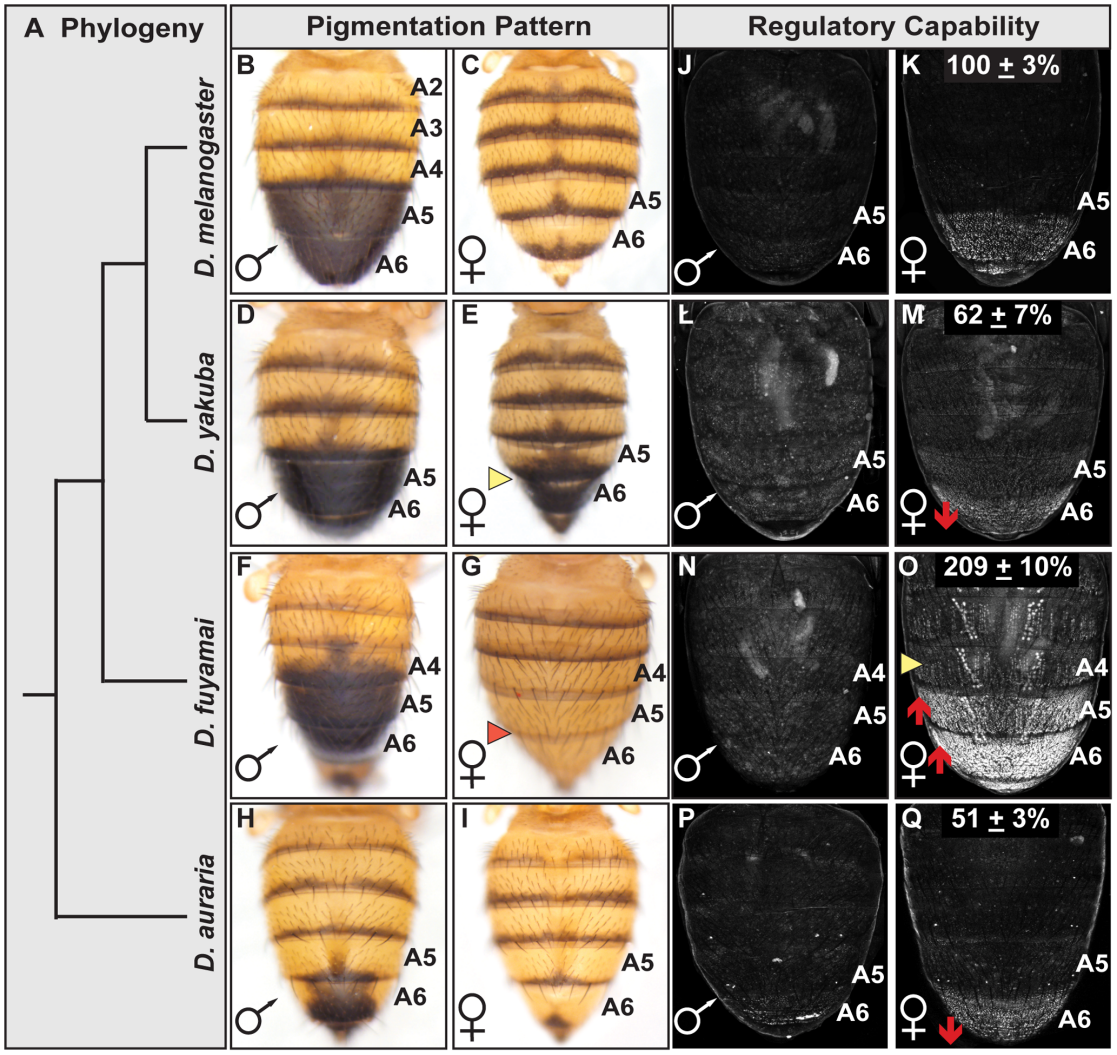
Interspecific evolution of pigmentation and dimorphic element activity. (A) Phylogeny for species that differ in the extent of sexually dimorphic pigmentation. (B–I) Dorsal view of adult abdomens, pigmentation of the (E) *D. yakuba* female A5 and A6 segments is more (D) male-like, whereas pigmentation is altogether absent on the A5 and A6 segments of (G) *D. fuyamai* females. (J–Q) Comparison of GFP-reporter gene activity in female transgenic pupae at 85 hr APF. Activity measurements are represented as the % of the (K) Concestor element female A6 mean ± SEM for (M) *D. yakuba*, (O) *D. fuyamai*, and (Q) *D. auraria*.

In the oriental lineage of the *Sophophora* subgenus, males of extant species generally are fully pigmented on the A5 and A6 tergites [23]. Female pigmentation is more variable, ranging from the complete absence of pigmentation like that seen for *D. fuyamai*, to a more male-like pattern like that seen for *D. yakuba* (Figure 6). Bab2 expression was found to be robustly sexually dimorphic for *D. fuyamai* [42], and Bab1 expression is reduced in the A5 and A6 segments of females (Salomone and Williams, unpublished data). These observations suggest that differences in Bab expression contribute to these different female pigmentation patterns. Multiple mechanisms could underlie these differences in Bab expression, including a change in the activity of or the expression pattern for a *trans*-acting regulator of the dimorphic element (*trans*-regulatory evolution). An alternative mechanism is through changes in orthologous dimorphic elements that result in differing responses to a conserved set of *trans*-regulators (*cis*-regulatory evolution).

An effective test to distinguish between instances of *cis*- and *trans*-regulatory evolution is to compare the activities of CREs in a common genetic background and observe whether reporter expression patterns resemble that of the host species (*trans*-regulatory evolution) or the species from which the CRE was derived (*cis*-regulatory evolution) [62]. We isolated orthologous dimorphic elements from *D. yakuba*, *D. fuyamai*, and an outgroup species *D. auraria* (from the *Sophophora* montium group) that is also sexually dimorphic for pigmentation and Bab expression though limited to the A6 segment [42]. The regulatory activities for these orthologous CREs were evaluated in transgenic *D. melanogaster* pupae and normalized to the Concestor element (Figure 6). The *D. auraria* dimorphic element exhibited an A6 segment regulatory activity of 51±3% of the Concestor element's activity (Figure 6Q). The regulatory activity of the *D. fuyamai* element was 209±10% (Figure 6O) and extended into segments A5-A2. The A6 regulatory activity for *D. yakuba* was 62±7% (Figure 6M). These results support a scenario where evolutionary changes in the extents of female posterior abdomen pigmentation for the presented clade (Figure 6) occurred, at least in part, via *cis*-regulatory evolution that altered the activity of orthologous dimorphic elements. Interestingly, of the 14 ABD-B and two DSX sites typical of the *D. melanogaster* dimorphic element, the orthologous *D. yakuba* and *D. fuyamai* sequences had the same 13 of the 14 ABD-B sites and both DSX sites (Figure S2B). Even the *D. auraria* dimorphic element, the most distantly related in this comparison, possessed 12 ABD-B sites and both DSX sites. Thus, like the situation for the *D. melanogaster* dimorphic element alleles, the functional diversification of these orthologous CREs occurred largely, if not entirely, by modifying CRE properties other than the ABD-B and DSX regulatory linkages.

## Discussion

**Figure 7:**
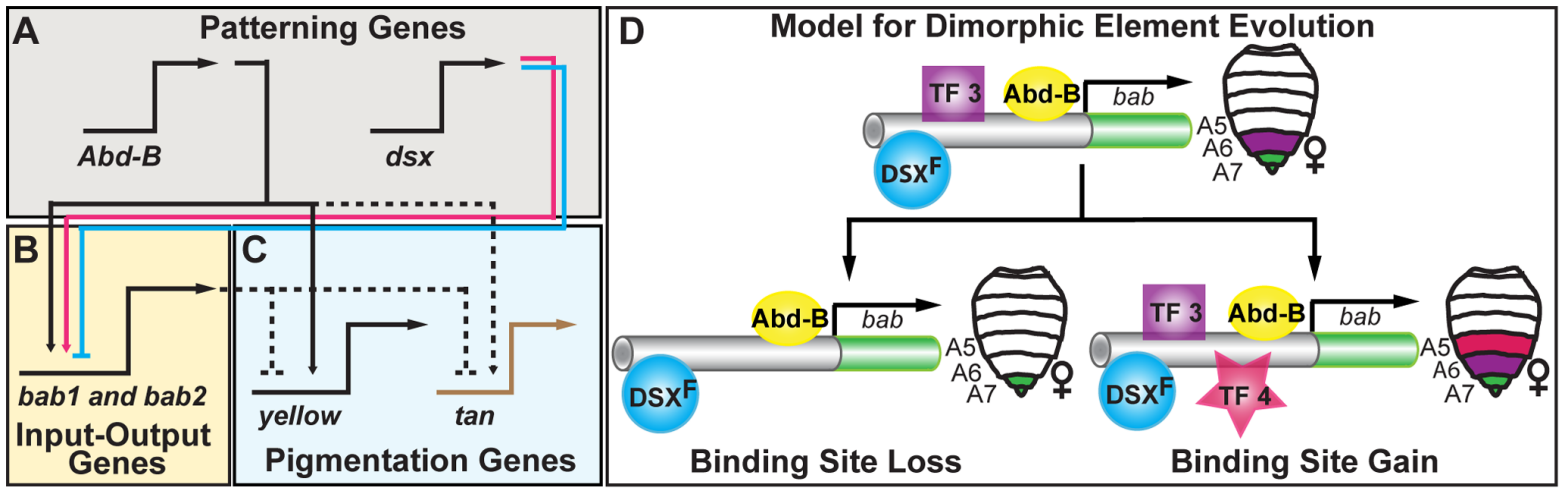
Pigmentation gene network model and the evolution of an ancestral CRE regulatory logic. (A–C) Schematic of the hierarchical structure of the *D. melanogaster* pigmentation gene network. Direct regulation is represented as solid connections and dashed connections represent connections where regulation has not been shown to be direct. Activation and repression are respectively indicated by the arrowhead and nail-head shapes. This network includes an (A) upper level of patterning genes, including *Abd-B* and *dsx* respectively of the body plan and sex-determination pathways, (B) a mid-level tier that integrates patterning inputs, (C) and a lower level that includes pigmentation genes whose encoded products function in pigment metabolism. Although *Abd-B* directly regulates the pigmentation gene *yellow*, sexually dimorphic expression of the *yellow* and *tan* genes results from the sexually dimorphic output of the *bab* locus that acts to repress *tan* and *yellow* expression in females. (D) A model for the evolution of diverse dimorphic element regulatory activities. The common ancestor of *D. melanogaster* populations and related species possessed a dimorphic element with both DSX and ABD-B regulatory linkages and that drove expression in the female A6–A8 segments. This ancestral regulatory logic was recurrently modified to increase the levels and expand the segmental domain of activity, or to decrease and contract activity. These changes occurred amidst the preservation of the core ABD-B and DSX regulatory linkages, perhaps though the loss (TF 3) and/or gain (TF 4) of other transcription factor linkages.

Here, we have shown that the *D. melanogaster* dimorphic element, a CRE that regulates a suite of sexually dimorphic traits, has alleles of strikingly different regulatory activities that impact just one of these traits, female abdomen pigmentation. By reconstructing the ancestral dimorphic element sequence for these alleles and determining its regulatory activity, we were able to identify the derived mutations responsible for the divergent activities of various alleles. These functionally-relevant mutations were few in number, each responsible for measureable effects on regulatory activity, and all but one modify a property other than the known ABD-B and DSX regulatory linkages identified previously [20]. Furthermore, we discovered that species related to *D. melanogaster* harbored evolutionarily relevant mutations in this same CRE, altering its regulatory activity in magnitudes and patterns comparable to the *D. melanogaster* alleles. These CRE modifications likely contribute to the divergent patterns of abdomen pigmentation for females of these species. These interspecific differences in dimorphic element activity occurred in the absence of noteworthy alterations to the known ancestrally encoded body plan and sex-determination pathway regulatory linkages. As a result, this CRE's regulatory activity in the terminal body segments (A7 and genitalia) has been conserved, while activity in more anterior segments has diverged. Collectively, this study can be interpreted to support a model where recurrent evolution can be biased to target certain genes and CREs (Figure 7A–7C), while preserving certain ancestral linkages (Figure 7D).

### Genetic Networks, CREs, and the Predictability of Evolution

The collaborative interactions of genes during development are hierarchically structured through the formation of a gene network at the level of expression [15]. At the top of these networks are patterning genes, prominently transcription factors that can form connections directly with CREs of differentiation genes, or with CRE(s) of intermediate level transcription factors that act as “Input-Output switches” [15], [19]. For the latter, the inputs are converted into a regulatory output that is directed to multiple target genes. On one hand, mutations altering a patterning gene may be sufficient to alter a network's phenotype, but these highly pleiotropic mutations tend to alter other phenotypes too, typically in a deleterious manner [63]. On the other hand, mutations altering the function of a single differentiation gene, while generally less pleiotropic often are insufficient to alter a phenotype. For these reasons, evolution may be biased to target Input-Output genes, an expectation that has been observed for several traits [19].

In the *D. melanogaster* pigmentation network, the *bab* genes function as an Input-Output node through the dimorphic element's integration of patterning inputs that include body plan (ABD-B) and sex determination (DSX) pathway inputs (Figure 7A). These inputs are converted into a female-specific pattern of expression that culminates in the repression of the differentiation genes *yellow* and *tan* in females [23], (Figure 7C). In principle, changes in the expression or activity of a patterning gene, differentiation gene, or the Input-Output gene (*bab*) could alter pigmentation phenotypes. In application though, it is logical that *bab* expression and dimorphic element encodings were modified as those alterations minimize negative pleiotropic effects while being sufficient to alter the female pigmentation phenotype. For example, ectopic *yellow* expression failed to create additional melanic pigmentation [64], [65], and changes in either DSX or ABD-B expression result in ectopic abdominal pigmentation in addition to several other trait phenotypes [20], [23], [66]. Thus, sufficiency for pigmentation is counterbalanced by the negative pleiotropic affects for these genes. In contrast, increased Bab expression in the A5 and A6 segments was sufficient to suppress pigmentation, and ectopic abdomen pigmentation develops in *bab* heterozygous and homozygous null mutant females (Figure 2E and 2H).

Bab though is not dedicated to pigmentation [41], [42]. In the pupa, Bab expression includes the leg tarsal segments, abdomen epidermis, sensory organ precursor cells, oenocytes, and dorsal abdominal muscles, and each of these expression patterns are governed by a modular CRE (s) [20]. Thus, Bab itself is highly pleiotropic, however it's CREs are far less pleiotropic. For this reason, mutations altering female pigmentation would maximize sufficiency and minimize pleiotropy if they occurred in the dimorphic element, an expectation borne out in this study. Pigmentation of the A5 and A6 segments, though, is only one of many traits influenced by the regulatory activity of the dimorphic element. This CRE drives Bab expression in the female A7 and A8 segments, regulating numerous female-specific traits, including the size, shape, trichome density, and bristle morphologies of the resident dorsal tergites and ventral sternites [41]. As expression in these more posterior segments require the ABD-B and DSX regulatory linkages, these regulatory linkages remain highly pleiotropic. For this reason, it seems logical that evolution would disfavor mutations that have deleterious consequences to these linkages and favor mutations that alter other CRE properties. This scenario reflects how dimorphic element function was modified in both the intraspecific and interspecific comparisons presented here as well as the long term conservation of the ABD-B and DSX linkages previously described [20].

### The Relationship between CRE Sequence and Functional Conservation

Our findings provide a unique contrast with previous investigations of the relationship between CRE conservation and CRE evolution. Although *Drosophila* non-coding DNA, including CRE sequences, evolves slower than synonymous sites [55], several well studied CREs were found to undergo substantial sequence evolution without matching regulatory activity evolution. During *Drosophila* embryonic development, the pair-rule gene *even-skipped* (*eve*) is expressed in seven stripes along the anteroposterior axis, with the second stripe of *eve* expression being specified by the stripe 2 element (S2E) CRE. In *D. melanogaster*, the S2E possesses binding sites for four transcription factors that collectively specify the *eve* expression output [67], [68]. The orthologous S2E from the species *D. pseudoobscura* differs in sequence for numerous binding sites, the overall content of binding sites, and spacing between conserved binding sites [69], [70], yet the orthologous S2Es function equivalently *in vivo* [71]. Hence, the S2E is an exemplar as to how selection acting at the level of the character (*eve* stripe expression) can accommodate a surprising amount of CRE evolution. Similarly, CRE sequence evolution without corresponding functional evolution was found between *Drosophila* species for the sparkling (spa) CRE that directs cone cell expression for the *dPax2* gene [72]. The content and spatial proximity of binding sites for neurogenic ectoderm enhancers (NEEs) evolved in order to conserve expression pattern outputs in response to changing regulatory inputs [24]. These case studies, demonstrate how CRE sequence conservation is not a prerequisite for CRE functional conservation.

In contrast, we found little divergence in the content and sequence of known binding sites for the *D. melanogaster* dimorphic element alleles and orthologous sequences. At the sequence level, these CRE alleles and orthologs respectively posses identities of ∼98% and ∼80%. Indeed, the vast majority of binding sites in the dimorphic element have been conserved for over 30 million years, showing conservation to *D. willistoni* [20]. At the functional level, these CREs exhibited striking differences in their regulatory activities (Figure 4 and Figure 6). Thus, in contrast to S2E, spa, and the NEEs, the dimorphic element demonstrates how CREs can derive dramatic changes in function that drive phenotypic divergence, with little-to-no alteration to the characterized pre-existing regulatory linkages.

### Integrating CRE Evolution into the Context of the Gene Locus

While the regulatory activity of the Light and Dark dimorphic elements alleles correlated with female A5 and A6 pigmentation (Figure 1), some outcomes suggest that these variant sequences are affected by other features within or perhaps outside of the *bab* locus. For instance, the Light 2 and Dark 2 alleles exhibit the highest and lowest regulatory activities respectively. Surprisingly, the Light 1 and Dark 1 alleles and their intermediate regulatory activities are associated with the more extreme Light and Dark female pigmentation phenotypes. At the expression level, Bab1 and Bab2 showed similar patterns in females from the Light 1 (prominent expression in segments A5 and A6) and Dark 1 (reduced expression is A5 and A6) strains (Figure 3). In the Dark 2 strain, Bab1 but not Bab2 expression was reduced in females. Several possible explanations might explain the uncoupled expression of the Bab paralogs in Dark 2. For example, it is possible that a separate, as of yet unidentified CRE controls Bab2 expression. However, a screen of the entire ∼160 kb locus failed to identify such a CRE [20]. A second possibility is that a mutation(s) in the Dark 2 allele has paralog-specific regulatory effects, perhaps by modifying an interaction with the promoter for *bab1* but not that of *bab2*.

Another possible explanation would involve the existence of CREs that coordinate communication between *bab1* and *bab2*. In such a scenario, the Dark 2 allele could contain mutations that alter interaction with coordinating elements to result in paralog-specific expression patterns in the female A5 and A6 segments. This possibility is consistent with observations of *bab* locus evolution in another population where females differ in A6 segment pigmentation [47]. For this population, fine-scale genetic mapping found that three disparate non-coding regions of the *bab* locus collaborate to compose a major effect QTL [48]. One of these regions spans the dimorphic element, though no mutations reside with this CRE's core element. The other two regions include an intergenic sequence between *bab1* and *bab2* and a large sequence that includes the *bab2* promoter. In the future, it will be important to understand what roles these other regions serve, and how they may interact with polymorphisms in the dimorphic element to produce paralog-specific effects on gene expression.

### Resurrecting Ancestral *Cis*-Regulatory Elements

With the centrality of CREs and their evolution to the diversification of phenotypic traits [16], [73], a major obstacle to reaching this goal is understanding the processes by which CRE regulatory logics were modified to contemporary forms [74]. Often studies of CRE evolution involve comparisons of two divergent derived regulatory states, where one sequence assumes the role of a surrogate for the ancestral function [20], [21], [35], [65], [74], [75]. This approach has been successful in making inferences about the ancestral states for regulatory linkages and identifying gains and losses of other key derived transcription factor binding sites. However, it is important to acknowledge a key limitation of this comparative approach; a CRE derived from an outgroup species that serves as a surrogate for the ancestor has also evolved along a unique lineage since divergence.

Studies into the evolution of divergent protein activities encountered a similar problem when comparing extant proteins forms [53]. For several cases, key amino acid residues necessary for a derived function were identified. When substituted into the surrogate ancestral protein, these changes were insufficient to impart the derived function and thereby indicating that the paths of evolution were more intricate. As a solution, the reconstruction of ancestral protein sequences, combined with functional testing of inferred ancestral proteins has allowed a more realistic simulation of evolutionary events. As a result, inferences about the paths of protein evolution were made that likely would not have been found from comparisons of extant proteins [51], [53].

A more ideal research program to study CRE evolution would include reconstruction of ancestral CREs as a starting point to trace the paths of evolutionarily relevant mutations. To our knowledge, few studies have used CRE reconstruction [34], [36], [54]. For one study, a novel optic lobe expression pattern for the *D. santomea Nep-1* gene occurred via the modification of a CRE that drove an eye field pattern of expression for an ancestor that existed ∼0.5 million years ago [36]. Importantly, by reconstructing and evaluating the ancestral CRE, the wrong conclusion - that this optic lobe activity evolved *de novo* – was avoided and the correct conclusion was found - a latent optic lobe CRE activity was augmented into a robust derived state. In our study, had the Concestor element not been reconstructed, the Dark 1 and Dark 2 dimorphic element sequences would have been considered hypomorphic CRE alleles compared to the robust wild type-like activity of the Light 1 and Light 2 alleles. The Light alleles possessed activities more similar to a previously characterized dimorphic element allele [20] and consistent with the narrative of *D. melanogaster* being a sexually dimorphic species where females lack posterior abdominal pigmentation. Reconstruction of the dimorphic element revealed a more complex reality, where neither alleles were good surrogates for the ancestral state. Using ancestral sequences as a starting point, we found that the evolutionary paths for these alleles to be short in number of steps (one to two mutations) and in time frame (in the last ∼60,000 years) [58]. Thus, demonstrating how simple and rapid an existing CRE regulatory logic can evolve.

The cases of *Nep1* optic lobe CRE and the *bab* dimorphic element evolution demonstrate the utility for reconstructing ancestral CRE states; though it must be pointed out that these cases involved comparisons of very closely-related species/populations. As a result of these short time frames for divergence, the extant CRE forms differ at fewer than two percent of the nucleotide sites. This made possible ancestral sequence reconstruction by the principle of parsimony. However, not all compelling instances of functional CRE evolution occur over similarly short time frames. Therefore, studies will need to reconstruct CREs that existed further in the past and for which the method of parsimony will need to be replaced by methods of maximum likelihood-based inference coupled with the testing of multiple alternate reconstructions [51].

## Materials and Methods

### Fly Stocks and Genetic Manipulations

*D. melanogaster* populations from disparate geographical regions were obtained from the San Diego *Drosophila* Stock Center and are identified in Figure S1. Dark 1 stock was obtained from M. Rebeiz [29], stocks for other species were obtained from S.B. Carroll. Reporter transgenes in Figure 1 were introduced into the *attP* site VK00006 on the X chromosome [76], all other reporter transgenes were introduced into the *attP2* site on chromosome 3L [77]. Complementation test progeny were obtained by crossing individuals from a *D. melanogaster* population stock to a line possessing the *bab* locus null allele *bab^AR07^* [41]. The homozygous *bab* null genotype was a heteroallelic combination of the *bab^AR07^* and the deficiency chromosome *Df(3L)BSC799* for which the entire *bab* locus is deleted.

### Sequencing *bab* Gene Exons and Splice Junctions

*bab1* and *bab2* protein coding exons from Light 1 and Dark 1 *bab* loci were amplified by PCR (Primer details in Table S3), cloned into the pGEMT-Easy vector (Promega), sequenced by the Sanger method (DNA Analysis LLC), and the resulting chromatograms were analyzed using the Staden software package [78].

### Introgression, Fine-Scale Genetic Mapping, and Association Testing

The Dark 1 female phenotype was introgressed for up 10 generations into the Light 1 genetic background. For each backcross generation, female progeny with a phenotype intermediate to that of the Light 1 and Dark 1 females (Figure 2C) were selected and mated to Light 1 males. Following 10 generations of backcrossing, male and female progeny were mated to generate pure lines for which females exhibited the Dark 1 phenotype (Figure S3F). Four *bab* locus marker genotypes were determined by PCR. These markers include #3, a *BstXI* restriction fragment length polymorphism (RFLP), and markers #1, #2, and #4 for which the PCR products differ in size when amplified from the Light 1 and Dark 1 stocks. PCR primers and population stock-specific allele sizes are provided in Table S4. For the RFLP analysis, the BstXI Fwd 1 and BstXI Rvs 1 primers (Table S4) were used to amplify a ∼381 base pair (bp) product from F2 progeny genomic DNA. PCR products were purified and digested with the BstXI restriction endonuclease and then size fractioned by agarose gel electrophoresis. PCR products from the Light 1 allele were cut into fragments of 235 and 146 bp, whereas products from the Dark 1 and Dark 2 alleles remained at 381 bp. The to-scale representation of the *bab* locus shown in Figure S3 was made using the Gene Palette software tool [79].

Genetic association tests were performed by crossing individuals from Dark 1 and separately Dark 2 stocks with individuals from Light 1 stock. F1 progeny were then intercrossed to generate an F2 generation. The abdomens of adult F2 progeny were imaged and then used to extract genomic DNA from (DNeasy Blood & Tissue Kit, Qiagen) for genotypic assays. F2 progeny genomic DNAs were then genotyped for the *BstXI* RFLP.

### Immunohistochemistry

Pupal abdomens were dissected for immunohistochemistry at ∼29 and ∼85 hours after puparium formation (hAPF), the former a time point when Bab1 and Bab2 are expressed in the developing genitalia and analia and the latter a time point when the dimorphic element drives high levels of reporter gene expression in the A5–A7 segments, and downstream targets of *bab* repression have begun to be expressed in males [23], [31]. The primary antibodies used were rabbit anti-Bab1 [20] and rat anti-Bab2 [80] at a dilution of 1∶250 and 1∶400 respectively. The secondary antibodies used were goat anti-rat Alexa Fluor 488 (Invitrogen) and goat anti-rabbit Alexa Fluor 647 (Invitrogen) at a dilution of 1∶500. The expression patterns presented are consistent with patterns seen in replicate specimens.

### Ancestral Sequence Reconstruction

Thirty one dimorphic element sequences were isolated from twenty seven world-wide populations of *D. melanogaster*. These sequences were used as an ingroup and aligned to seven outgroup sequences from related species by the Chaos+Dialign alignment tool [81]. From this alignment (Figure S2), using the parsimony principle we reconstructed the sequence (named the “Concestor element”) possessed by the most recent common ancestor of the surveyed *D. melanogaster* population stocks. This ancestral reconstructed sequence was synthesized (GenScript) for use in reporter transgene analyses.

Outgroup species relationship were based on a published phylogeny [23]. Polymorphic sites among *D. melanogaster* population alleles are distinguished in the alignment as red text on a black background. *D. melanogaster* dimorphic element alleles in the alignment are referred to as mel.##.#, which refers to the species name, stock number (from the San Diego Drosophila Species Stock Center), and the clone number assigned to the sequence cloned into the BPS3aG vector. Sequence references that include “Ug”, were isolated from chromosome extractions from a Uganda Africa population [29], [82]. Orthologous dimorphic element sequences for outgroup species are referred to by the species three letter abbreviation and clone number assigned to the sequence when cloned into the BPS3aG vector.

Derived mutations in the region where characterized ABD-B and DSX binding sites reside [20], referred to as the “core” (Figure S5), are identified by a alphabetic letter designation above the nucleotide position (Figure S2). Polymorphic sites in regions flanking the core were assigned a numerical designation that is listed above the variable nucleotide position in the alignment. The characterized binding sites for ABD-B (14 sites for *D. melanogaster*) are indicated by white text on a blue background, whereas the two DSX binding sties (Dsx1 and Dsx2 sites) are indicated by black text on a yellow background. The sites were previously found to be bound by these transcription factors *in vitro* [20] and their sequences respectively match the empirically derived consensus motifs for ABD-B (TTTAY) and DSX (RNNACWAWGTNNY) [61], [83]. Ambiguously reconstructed Concestor element nucleotide states are indicated as blue or black text on a gray background. The ggcgcgcc and cctgcagg sequences respectively at the 5′ and 3′ ends of the dimorphic sequences are not part of the endogenous *bab* sequences, but are respectively *AscI* and *SbfI* restriction endonuclease sites that were included by PCR for cloning into the BPS3aG vector. The polymorphic *BstXI* restriction endonuclease site (CCANNNNNNTGG) is indicated by white text on a dark red background (Figure S2).

### Reporter Transgenes

GFP reporter transgenes were used as a proxy to measure the *in vivo* gene-regulatory activity of CREs. In brief, CREs are cloned into a vector upstream of the green fluorescent protein (GFP) coding sequence forming a “reporter transgene”. Transgenes were individually inserted into the *D. melanogaster* germline at the same genomic location via site-specific integration methods to avoid confounding position effects, which permits a quantitative comparison of CRE regulatory capabilities [20], [60], [77] (BestGene Inc.). All dimorphic element sequences were amplified using the sub1orthoF1 and dimorphic Rvs1 primers that were designed to sequences conserved between species from the most divergent *Sophophora* lineages (Table S5). Dimorphic elements were cloned into the *AscI* and *SbfI* sites in the vector BPS3aG, a vector derived from the S3aG vector [60] by the inclusion of a 119 bp sequence from the *bab2* promoter inserted between the *BamHI* and *XhoI* sites.

Regulatory activities were determined as the mean GFP intensities and standard error of the mean (SEM) for female dorsal abdominal segment A6 expression as previously described [20], [60]. For each transgene, a preliminary analysis was done for several independent transgenic lines to gauge the level and pattern of activity and variation between replicate specimens. Regulatory activities were then determined using three or more newly acquired specimens that were at the same developmental time point (85 hAPF). The samples sizes (n) for Figure 1A′–1H′ respectively were: 5, 8, 9, 4, 6, 8, 11, and 4. The samples sizes (n) for Figure 4A′–4E′ respectively were: 34, 9, 6, 6, and 3. The samples sizes (n) for Figure 5F–5J respectively were: 34, 10, 6, 11, and 14. In Figure 5K, the n values for Concestor, E scramble, E8Del, Dsx KO, E Dsx1, and Concestor+E respectively were: 34, 15, 28, 23, 22, and 6. The samples sizes (n) for Figure 6K, 6M, 6O, and 6Q respectively were: 9, 3, 6, and 18. The samples sizes (n) for –S5L respectively were: 6, 6, 6, 10, 7, 6, 6, 6, 8, 14, and 6. The samples sizes (n) for Figure S6A–S6AA respectively were: 34, 44, 6, 9, 6, 3, 45, 10, 6, 11, 12, 14, 15, 22, 23, 14, 22, 12, 13, 30, 21, 15, 28, 22, 23, 17, and 6. Activities reported in Figure 1 were normalized to an allele from the *Canton^S^* strain [20]. All other regulatory activities used the Concestor element transgenic lines for normalization.

Derived mutations that alter dimorphic element function were mapped by the construction and transgenic evaluation of chimeric reporter transgenes [74]. In brief, a series of chimeric dimorphic elements were constructed in which a broad region(s) from the Concestor element was combined with the complementary region from a Light or Dark dimorphic element allele. Regions of alleles sufficient to impart some of the evolved activity on an otherwise Concestor element were refined to find smaller regions responsible for or contributing to the activity differences. This culminated with tests of individual mutations.

### Evaluating Robustness in Dimorphic Element Reconstruction

Ancestral sequence inferences occur with a certain degree of ambiguity that can result in incorrect evolutionary conclusions. One way to estimate the confidence in a particular reconstruction, is to test the function of other possible ancestral sequences [51]. In the reconstructed Concestor element sequence, we were uncertain of the ancestral nucleotide state at eight sites (sites 1, 17, 19, H, K, 27, 30, and 31; Figure S2A). Two of these sites were the “H” and “K” mutations that respectively occur at repeat tracts of C and T nucleotides. The difference in number of nucleotides among the surveyed alleles ranged between 0–7 for the C tract and 0–3 for the T tract (Figure S2). Length differences occur in the Light 1 allele and both Dark alleles, suggesting these differences would not be responsible for the allele-specific regulatory activities. To test this suggestion, we made two modified Concestor elements, one where four C nucleotides were added to the H mutation site, and the other where three T nucleotides were added to the K mutation site. These alterations had no significant effect on the Concestor element's regulatory activity (Figure S6L and S6O), thus, supporting that this reconstruction was robust to inference uncertainty at these two sites, and ruling out the H and K mutations as being functionally-relevant. We also synthesized an ancestral sequence, called Concestor 2, which differed from the Concestor element at six sites (Figure S2; sites 1, 17, 19, 27, 30, and 31). While this sequence had an activity of 125±1% of the Concestor element (Figure S6B), this difference was quite modest compared to the activities of the Light and Dark alleles. Moreover, this result supported the evolutionary conclusion that the regulatory activity of the dimorphic element possessed by the most recent common ancestor of the surveyed population stock alleles was intermediate to the alleles with reduced and increased activity in the female A6 segment. Chimeric constructs and tests of derived mutations were done using the Concestor element sequence.

### Gel Shift Assays

Gel shift assays used the DSX DNA-binding domain proteins and wild type and mutant Dsx1 sites as previously published [20]. Sequences for oligonucleotides used for gel shift assay probes are presented in Table S6. Reverse complementary oligonucleotides were synthesized (Integrated DNA Technologies) that contain the Concestor element, E mutation variant, and a null mutation for Dsx1 site sequence, each flanked by endogenous dimorphic element sequence. Each oligonucleotide was biotin-labeled on their 3′ end using the DNA 3′ End Biotinylation Kit (Thermo Scientific). Labeled complementary oligonucleotides were annealed by standard protocol to make binding sites for gel shift assays. Labeling efficiency for each binding site was determined using a quantitative Dot Blot assay (DNA 3′ End Biotinylation Kit, Thermo Scientific). All gel shift reactions included 20 fmol of one labeled binding site and GST-DSX DNA Binding Domain (DBD) fusion protein [20] in General Footprint Buffer (50 mM HEPES pH 7.9, 100 mM KCl, 1 mM DTT, 12.5 mM MgCl2, 0.05 mM EDTA, 17% glycerol). For each binding site, a reaction was done that included an amount of DSX protein ranging from 500 ng down to 8 ng. For each binding site, a control reaction was done that lacked DSX protein. Binding reactions were carried out for 30 minutes on ice. Reactions were then separated through a 5% non-denaturing polyacrylamide gel for 2 hours at 200 V. Following electrophoresis, reactions were transferred and cross linked to a Hybond-N+ membrane (GE Healthcare Amersham) for chemiluminescent detection using the Chemiluminescent Nucleic Acid Detection Module and manufacture's protocol (Thermo Scientific). Chemiluminescent images were taken using a BioChemi gel documentation system (UVP). The results shown in Figure 5 were representative of those obtained in independent replicate experiments (n = 3).

### Imaging of Fly Abdomens

Whole-mount images were taken using an Olympus SZX16 Zoom Stereoscope outfitted with an Olympus DP72 digital camera. Projection images for immunohistochemistry and reporter transgenes where obtained using an Olympus Fluoview FV 1000 confocal microscope and software. All TIFF images used in a specific comparison were processed through the same modification using Photoshop CS3 (Adobe).

## Supporting Information

Figure S1 Abdomen pigmentation phenotypes for *Drosophila melanogaster* population stocks. (A-AN) Whole mount images of adult male and female dorsal abdomens. Geographic locations for the populations from which lab stocks were started are listed and, when applicable, in parentheses are the Drosophila Species Stock Center stock numbers. Representative images for the stocks referred to as (A) Light 1 population, (D) Light 2, (AM) Dark 1, and (AJ) Dark 2.
(TIF)

Figure S2 Sequence alignments for dimorphic elements. (A) Annotated alignment of dimorphic elements used to reconstruct ancestral sequences (Concestor and Concestor 2) from extant *D. melanogaster* populations. Dimorphic elements from *D. mauritiana* (mau.5), *D. sechellia* (sec.38), *D. simulans* (sim.33), *D. yakuba* (yak.25), *D. lucipennis* (luc.41), *D. eugracilis* (eug.20), and *D. fuyamai* (fuy.9) were used as out groups. (B) Annotated alignment of orthologous dimorphic elements from *D. melanogaster* Light 1 allele, *D. yakuba* (D. yak), *D. fuyamai* (D. fuy), and *D. auraria* (D. aur). White font on purple background indicates the *AscI* and *SbfI* restriction enzyme sites that were introduced for cloning purposes. Red font on black background indicates polymorphisms among the population-stock alleles. At the top of alignment is the number or letter designation assigned to each polymorphism. Ambiguous sites in the reconstructed concestor sequences are indicated by a gray background color. Characterized ABD-B and DSX binding sites are indicated respectively by white font on a blue background and black font on a yellow background. The *BstXI* restriction enzyme site used for genotyping is indicated by white font on a maroon background.
(DOC)

Figure S3 Mapping of the *bab* genotype-phenotype association. (A) To scale representation of the ∼155 kb *bab* locus, where the *bab1* and *bab2* genes are situated between the *CG13912* and *trio* genes. Exons are indicated as the tall rectangles, and sites and directions for each gene's transcription are indicated by the black arrows. The location of polymorphic markers used to establish *bab* loci haplotypes are indicated by “1”, “2”, “3”, and “4”, and downward projecting red lines. Polymorphism 3 is the *BstXI* site polymorphism that resides within the Light 1 and Light 2 dimorphic element alleles. Blue dot with arrow indicates location of the dimorphic element. Representative female phenotypes (B–F) for the A5 and A6 segment tergites (Left) and the inferred *bab* locus haplotypes associated with the pigmentation phenotype (Right). (B) Dark 1 and (C) Light 1 specimens were homozygous for alternate nucleotide states at the four *bab* locus markers, establishing a Dark 1 and Light 1 haplotypes (Black and Yellow bars respectively). (D) Female F1 progeny from Dark 1 and Light 1 cross were heterozygous for *bab* locus markers. (E) Phenotypically Dark F2 progeny from parental Dark 1 and Light 1 cross were homozygous for the Dark 1 nucleotide state at each of the four evaluated *bab* locus markers. (F) Following 10 generations of backcrossing the Dark 1 phenotype into the Light 1 genetic background, a pure line was established where females exhibit the Dark 1 phenotype. This line was homozygous for the Dark 1 nucleotide state at each of the four evaluated *bab* locus markers.
(TIF)

Figure S4 Protein coding sequence variation for the *bab* alleles. To scale representations of the (A) Bab1 and (B) Bab2 proteins, including the BTB Domain (red) and Bab conserved domain (CD, blue). The positions of nonsynonymous differences between the Light 1 and Dark 1 sequences are annotated and compared to the amino acid states for the *D. melanogaster* genome strain and the outgroup species *D. sechellia*. The aligned DNA sequences for (C) *bab1* and (D) *bab2* protein-coding exons and adjacent splice donor and acceptor sequences (shown with black text on gray background.
(DOC)

Figure S5 Chimeric dimorphic elements map functionally-relevant derived mutations to the core region. (A) To scale representation of the dimorphic element, with ABD-B and DSX binding sites shown as blue and yellow rectangles respectively. Green dashed lines indicate the positions where central core dimorphic element sequences were joined with flank sequences. Blue dashed line indicates the position where left and right halves of various dimorphic elements were joined. (B–L) GFP-reporter gene activity in female transgenic pupae at 85 hAPF. Activity measurements are represented as the % of the *D. melanogaster* Concestor element female A6 mean ± SEM. The illustration above each image indicates the sequence composition of the evaluated dimorphic elements. Gray, yellow, and brown colors respectively indicate sequence from the Concestor element, Light 2 dimorphic element, and the Dark 1 dimorphic element.
(TIF)

Figure S6 Regulatory activity effects of derived dimorphic element mutations. (A-AA) GFP-reporter gene activities in female transgenic pupae at 85 hAPF. (A) The Concestor element's mean activity measurement in the dorsal A6 segment was set as 100%, all other regulatory activities (B-AA) are reported as a percentage of the Concestor element's activity ± the standard error of the mean (SEM). For each reporter transgene, a representative image is presented. (C–F) Activities for population stock dimorphic element alleles. (G–R) Activities for Concestor elements with a substitution of a single mutation. (S and T) Activities for Concestor elements substituted with two Light 2 (S) and two Dark 1 (T) derived mutations. (U) The regulatory activity of the Dark 1 allele that included the E mutation. (V) The Concestor element's regulatory activity when the native sequence at the site of the E mutation was altered by non-complementary transversion at every 2^nd^ base pair. (W) The Concestor element's regulatory activity when the first 8 of 9 base pairs of the E mutation were deleted. (X) The Concestor element's regulatory activity when only base pair 9 of the E mutation was deleted. (Y) The Concestor element's regulatory activity when the Dsx1 Site was mutated. (Z) The Concestor element's regulatory activity in males when the Dsx1 Site was mutated. (AA) The Concestor element's regulatory activity in males relative to its activity in females.
(TIF)

Table S1 Association between pigmentation phenotype and *bab* dimorphic element genotype.
(DOC)

Table S2 Association between pigmentation phenotype and *bab* dimorphic element genotype.
(DOC)

Table S3 Primers used to PCR amplify *D. melanogaster bab* protein coding exons and their splice junctions.
(DOC)

Table S4 Primers used for PCR-based genotyping of the *D. melanogaster bab* locus.
(DOC)

Table S5 Primer combinations used to amplify and clone dimorphic element alleles and orthologous sequences.
(DOC)

Table S6 Oligonucleotides used to make gel shift assay binding sites.
(DOC)

## References

[1] Conway Morris S (2003) Life's Solution. Inevitable Humans in a Lonely Universe. 1st ed. Cambridge: Cambridge University Press.

[2] Gompel N, Prud'homme B (2009) The causes of repeated genetic evolution. Developmental biology 332: 36–47 Available:http://www.ncbi.nlm.nih.gov/pubmed/19433086. Accessed 12 March 2013. 19433086 10.1016/j.ydbio.2009.04.040

[3] Shapiro MD, Bell MA, Kingsley DM (2006) Parallel genetic origins of pelvic reduction in vertebrates. Proceedings of the National Academy of Sciences of the United States of America 103: 13753–13758 http://www.pubmedcentral.nih.gov/articlerender.fcgi?artid=1564237&tool=pmcentrez&rendertype=abstract. 16945911 10.1073/pnas.0604706103 PMC1564237

[4] Protas ME, Hersey C, Kochanek D, Zhou Y, Wilkens H, et al (2006) Genetic analysis of cavefish reveals molecular convergence in the evolution of albinism. Nature genetics 38: 107–111 Available:http://www.ncbi.nlm.nih.gov/pubmed/16341223. Accessed 1 March 2013. 16341223 10.1038/ng1700

[5] Sucena E, Delon I, Jones I, Payre F, Stern DL (2003) Regulatory evolution of shavenbaby/ovo underlies multiple cases of morphological parallelism. Nature 424: 935–938 Available:http://www.ncbi.nlm.nih.gov/pubmed/12931187. 12931187 10.1038/nature01768

[6] Prud'homme B, Gompel N, Rokas A, Kassner VA, Williams TM, et al (2006) Repeated morphological evolution through cis-regulatory changes in a pleiotropic gene. Nature 440: 1050–1053 Available:http://www.ncbi.nlm.nih.gov/pubmed/16625197. Accessed 10 July 2011. 16625197 10.1038/nature04597

[7] Mundy NI (2005) A window on the genetics of evolution: MC1R and plumage colouration in birds. Proceedings Biological sciences/The Royal Society 272: 1633–1640 Available:http://www.pubmedcentral.nih.gov/articlerender.fcgi?artid=1559852&tool=pmcentrez&rendertype=abstract. Accessed 8 March 2013. 16087416 10.1098/rspb.2005.3107 PMC1559852

[8] Nachman MW, Hoekstra HE, D'Agostino SL (2003) The genetic basis of adaptive melanism in pocket mice. Proceedings of the National Academy of Sciences of the United States of America 100: 5268–5273 Available:http://www.pubmedcentral.nih.gov/articlerender.fcgi?artid=154334&tool=pmcentrez&rendertype=abstract. 12704245 10.1073/pnas.0431157100 PMC154334

[9] Zhen Y, Aardema ML, Medina EM, Schumer M, Andolfatto P (2012) Parallel molecular evolution in an herbivore community. Science (New York, NY) 337: 1634–1637 Available:http://www.ncbi.nlm.nih.gov/pubmed/23019645. Accessed 8 November 2012. 10.1126/science.1226630 PMC3770729 23019645

[10] Zhang J (2006) Parallel adaptive origins of digestive RNases in Asian and African leaf monkeys. Nature genetics 38: 819–823 Available:http://www.ncbi.nlm.nih.gov/pubmed/16767103. Accessed 9 March 2013. 16767103 10.1038/ng1812

[11] Jones FC, Grabherr MG, Chan YF, Russell P, Mauceli E, et al (2012) The genomic basis of adaptive evolution in threespine sticklebacks. Nature 484: 55–61 Available:http://www.nature.com/doifinder/10.1038/nature10944. Accessed 4 April 2012. 22481358 10.1038/nature10944 PMC3322419

[12] Frankel N, Wang S, Stern DL (2012) Conserved regulatory architecture underlies parallel genetic changes and convergent phenotypic evolution. Proceedings of the National Academy of Sciences 109 (51) 20975–9 Available:http://www.pnas.org/cgi/doi/10.1073/pnas.1207715109. Accessed 1 December 2012. 10.1073/pnas.1207715109 PMC3529038 23197832

[13] Bonn S, Furlong EEM (2008) cis-Regulatory networks during development: a view of Drosophila. Current opinion in genetics & development 18: 513–520 Available:http://www.ncbi.nlm.nih.gov/pubmed/18929653. Accessed 14 March 2012. 18929653 10.1016/j.gde.2008.09.005

[14] Levine M, Davidson EH (2005) Gene regulatory networks for development. Proceedings of the National Academy of Sciences of the United States of America 102: 4936–4942 Available:http://www.pubmedcentral.nih.gov/articlerender.fcgi?artid=555974&tool=pmcentrez&rendertype=abstract. 15788537 10.1073/pnas.0408031102 PMC555974

[15] Davidson EH (2006) The Regulatory Genome: Gene Regulatory Networks In Development And Evolution. Burlington, MA: Elsevier Inc. p.

[16] Carroll SB (2008) Evo-devo and an expanding evolutionary synthesis: a genetic theory of morphological evolution. Cell 134: 25–36 Available:http://www.ncbi.nlm.nih.gov/pubmed/18614008. Accessed 24 July 2011. 18614008 10.1016/j.cell.2008.06.030

[17] Arnone MI, Davidson EH (1997) The hardwiring of development: organization and function of genomic regulatory systems. Development (Cambridge, England) 124: 1851–1864 Available:http://www.ncbi.nlm.nih.gov/pubmed/9169833. 10.1242/dev.124.10.1851 9169833

[18] Cooper TF, Ostrowski EA, Travisano M (2007) A negative relationship between mutation pleiotropy and fitness effect in yeast. Evolution 61: 1495–1499. 17542856 10.1111/j.1558-5646.2007.00109.x

[19] Stern DL, Orgogozo V (2009) Is genetic evolution predictable? Science (New York, NY) 323: 746–751 Available:http://www.ncbi.nlm.nih.gov/pubmed/19197055. Accessed 21 July 2011. 10.1126/science.1158997 PMC3184636 19197055

[20] Williams TM, Selegue JE, Werner T, Gompel N, Kopp A, et al (2008) The regulation and evolution of a genetic switch controlling sexually dimorphic traits in Drosophila. Cell 134: 610–623 Available:http://www.pubmedcentral.nih.gov/articlerender.fcgi?artid=2597198&tool=pmcentrez&rendertype=abstract. Accessed 4 August 2011. 18724934 10.1016/j.cell.2008.06.052 PMC2597198

[21] Shirangi TR, Dufour HD, Williams TM, Carroll SB (2009) Rapid evolution of sex pheromone-producing enzyme expression in Drosophila. PLoS biology 7: e1000168 Available:http://www.pubmedcentral.nih.gov/articlerender.fcgi?artid=2711336&tool=pmcentrez&rendertype=abstract. Accessed 25 July 2011. 19652700 10.1371/journal.pbio.1000168 PMC2711336

[22] Chan YF, Marks ME, Jones FC, Villarreal G, Shapiro MD, et al (2010) Adaptive evolution of pelvic reduction in sticklebacks by recurrent deletion of a Pitx1 enhancer. Science (New York, NY) 327: 302–305 Available:http://www.pubmedcentral.nih.gov/articlerender.fcgi?artid=3109066&tool=pmcentrez&rendertype=abstract. Accessed 30 July 2011. 10.1126/science.1182213 PMC3109066 20007865

[23] Jeong S, Rokas A, Carroll SB (2006) Regulation of body pigmentation by the Abdominal-B Hox protein and its gain and loss in Drosophila evolution. Cell 125: 1387–1399 Available:http://www.ncbi.nlm.nih.gov/pubmed/16814723. Accessed 27 August 2011. 16814723 10.1016/j.cell.2006.04.043

[24] Crocker J, Tamori Y, Erives A (2008) Evolution acts on enhancer organization to fine-tune gradient threshold readouts. PLoS biology 6: e263 Available:http://www.pubmedcentral.nih.gov/articlerender.fcgi?artid=2577699&tool=pmcentrez&rendertype=abstract. Accessed 1 March 2012. 18986212 10.1371/journal.pbio.0060263 PMC2577699

[25] Tournamille C, Colin Y, Cartron JP, Le Van Kim C (1995) Disruption of a GATA motif in the Duffy gene promoter abolishes erythroid gene expression in Duffy–negative individuals. Nature genetics 10: 224–228. 7663520 10.1038/ng0695-224

[26] Wang X, Chamberlin HM (2002) Multiple regulatory changes contribute to the evolution of the Caenorhabditis lin-48 ovo gene. Genes & development 16: 2345–2349 Available:http://www.pubmedcentral.nih.gov/articlerender.fcgi?artid=187439&tool=pmcentrez&rendertype=abstract. Accessed 26 July 2012. 12231624 10.1101/gad.996302 PMC187439

[27] Shim S, Kwan KY, Li M, Lefebvre V, Šestan N (2012) Cis-regulatory control of corticospinal system development and evolution. Nature 486: 74–79 Available:http://www.nature.com/doifinder/10.1038/nature11094. Accessed 30 May 2012. 22678282 10.1038/nature11094 PMC3375921

[28] Arnoult L, Su KFY, Manoel D, Minervino C, Magrina J, et al (2013) Emergence and Diversification of Fly Pigmentation Through Evolution of a Gene Regulatory Module. Science 339: 1423–1426 Available:http://www.sciencemag.org/cgi/doi/10.1126/science.1233749. Accessed 22 March 2013. 23520110 10.1126/science.1233749

[29] Rebeiz M, Pool JE, Kassner VA, Aquadro CF, Carroll SB (2009) Stepwise modification of a modular enhancer underlies adaptation in a Drosophila population. Science (New York, NY) 326: 1663–1667 Available:http://www.ncbi.nlm.nih.gov/pubmed/20019281. Accessed 2 August 2011. 10.1126/science.1178357 PMC3363996 20019281

[30] Cretekos CJ, Wang Y, Green ED, Martin JF, Rasweiler JJ, et al (2008) Regulatory divergence modifies limb length between mammals. Genes & development 22: 141–151 Available:http://www.pubmedcentral.nih.gov/articlerender.fcgi?artid=2192750&tool=pmcentrez&rendertype=abstract. Accessed 21 July 2011. 18198333 10.1101/gad.1620408 PMC2192750

[31] Jeong S, Rebeiz M, Andolfatto P, Werner T, True J, et al (2008) The evolution of gene regulation underlies a morphological difference between two Drosophila sister species. Cell 132: 783–793 Available:http://www.ncbi.nlm.nih.gov/pubmed/18329365. Accessed 10 June 2011. 18329365 10.1016/j.cell.2008.01.014

[32] Marcellini S, Simpson P (2006) Two or four bristles: functional evolution of an enhancer of scute in Drosophilidae. PLoS biology 4: e386 Available:http://www.pubmedcentral.nih.gov/articlerender.fcgi?artid=1635746&tool=pmcentrez&rendertype=abstract. Accessed 8 March 2012. 17105353 10.1371/journal.pbio.0040386 PMC1635746

[33] McGregor AP, Orgogozo V, Delon I, Zanet J, Srinivasan DG, et al (2007) Morphological evolution through multiple cis-regulatory mutations at a single gene. Nature 448: 587–590 Available:http://www.ncbi.nlm.nih.gov/pubmed/17632547. Accessed 18 July 2011. 17632547 10.1038/nature05988

[34] Tishkoff SA, Reed FA, Ranciaro A, Voight BF, Babbitt CC, et al (2007) Convergent adaptation of human lactase persistence in Africa and Europe. Nature genetics 39: 31–40 Available:http://www.pubmedcentral.nih.gov/articlerender.fcgi?artid=2672153&tool=pmcentrez&rendertype=abstract. Accessed 7 July 2011. 17159977 10.1038/ng1946 PMC2672153

[35] Frankel N, Erezyilmaz DF, McGregor AP, Wang S, Payre F, et al (2011) Morphological evolution caused by many subtle-effect substitutions in regulatory DNA. Nature 474: 598–603 Available:http://www.nature.com/doifinder/10.1038/nature10200. Accessed 29 June 2011. 21720363 10.1038/nature10200 PMC3170772

[36] Rebeiz M, Jikomes N, Kassner VA, Carroll SB (2011) Evolutionary origin of a novel gene expression pattern through co-option of the latent activities of existing regulatory sequences. Proceedings of the National Academy of Sciences of the United States of America 108: 10036–10043 Available:http://www.pubmedcentral.nih.gov/articlerender.fcgi?artid=3121811&tool=pmcentrez&rendertype=abstract. Accessed 7 July 2011. 21593416 10.1073/pnas.1105937108 PMC3121811

[37] Emera D, Wagner GP (2012) Transformation of a transposon into a derived prolactin promoter with function during human pregnancy. Proceedings of the National Academy of Sciences of the United States of America 109: 11246–51 Available:http://www.ncbi.nlm.nih.gov/pubmed/22733751. Accessed 27 June 2012. 22733751 10.1073/pnas.1118566109 PMC3396485

[38] Werner T, Koshikawa S, Williams TM, Carroll SB (2010) Generation of a novel wing colour pattern by the Wingless morphogen. Nature 464: 1143–1148 Available:http://www.ncbi.nlm.nih.gov/pubmed/20376004. Accessed 15 July 2011. 20376004 10.1038/nature08896

[39] Kopp A, Duncan I (2002) Anteroposterior patterning in adult abdominal segments of Drosophila. Developmental biology 242: 15–30 Available:http://www.ncbi.nlm.nih.gov/pubmed/11795937. Accessed 19 February 2012. 11795937 10.1006/dbio.2001.0529

[40] Wang W, Yoder JH (2012) Hox-mediated regulation of doublesex sculpts sex-specific abdomen morphology in Drosophila. Developmental dynamics 241: 1076–1090 Available:http://www.ncbi.nlm.nih.gov/pubmed/22488883. Accessed 26 July 2012. 22488883 10.1002/dvdy.23791

[41] Couderc J-L, Godt D, Zollman S, Chen J, Li M, et al (2002) The bric à brac locus consists of two paralogous genes encoding BTB/POZ domain proteins and acts as a homeotic and morphogenetic regulator of imaginal development in Drosophila. Development (Cambridge, England) 129: 2419–2433 Available:http://www.ncbi.nlm.nih.gov/pubmed/11973274. 10.1242/dev.129.10.2419 11973274

[42] Kopp A, Duncan I, Carroll SB (2000) Genetic control and evolution of sexually dimorphic characters in Drosophila. Nature 408: 553–559 Available:http://www.ncbi.nlm.nih.gov/pubmed/11117736. 11117736 10.1038/35046017

[43] Parkash R, Sharma V, Kalra B (2009) Impact of body melanisation on desiccation resistance in montane populations of D. melanogaster: Analysis of seasonal variation. Journal of insect physiology 55: 898–908 Available:http://www.ncbi.nlm.nih.gov/pubmed/19538968. Accessed 26 July 2012. 19538968 10.1016/j.jinsphys.2009.06.004

[44] Parkash R, Sharma V, Kalra B (2008) Climatic adaptations of body melanisation in Drosophila melanogaster from Western Himalayas. Fly 2: 111–117 Available:http://www.ncbi.nlm.nih.gov/pubmed/18820467. 18820467 10.4161/fly.6351

[45] Robertson A, Briscoe DA, Louw JH (1977) Variation in abdomen pigmentation in Drosophila melanogaster females. Genetica 47: 73–76.

[46] Robertson A, Louw JH (1966) Polymorphism of genes affecting amount and distribution of black pigment in the abdominal cuticle of D. melanogaster. Drosophila Information Service 41: 154–155.

[47] Kopp A, Graze RM, Xu S, Carroll SB, Nuzhdin S V (2003) Quantitative Trait Loci Responsible for Variation in Sexually Dimorphic Traits in Drosophila melanogaster. Genetics 787: 771–787. 12618413 10.1093/genetics/163.2.771 PMC1462463

[48] Bickel RD, Kopp A, Nuzhdin S V (2011) Composite effects of polymorphisms near multiple regulatory elements create a major-effect QTL. PLoS genetics 7: e1001275 Available:http://www.pubmedcentral.nih.gov/articlerender.fcgi?artid=3020931&tool=pmcentrez&rendertype=abstract. Accessed 26 October 2012. 21249179 10.1371/journal.pgen.1001275 PMC3020931

[49] Wray GA (2000) Editorial: Peering ahead (cautiously). Evolution & development 2: 125–126. 11252566 10.1046/j.1525-142x.2000.00001.x

[50] Abouheif E (2008) Parallelism as the pattern and process of mesoevolution. Evolution & development 10: 3–5 Available:http://www.ncbi.nlm.nih.gov/pubmed/18184352. 18184352 10.1111/j.1525-142X.2007.00208.x

[51] Thornton JW (2004) Resurrecting ancient genes: experimental analysis of extinct molecules. Nature reviews Genetics 5: 366–375 Available:http://www.ncbi.nlm.nih.gov/pubmed/15143319. Accessed 7 March 2012. 10.1038/nrg1324 15143319

[52] Russo CA, Takezaki N, Nei M (1995) Molecular phylogeny and divergence times of drosophilid species. Molecular biology and evolution 12: 391–404 Available:http://www.ncbi.nlm.nih.gov/pubmed/7739381. 7739381 10.1093/oxfordjournals.molbev.a040214

[53] Harms MJ, Thornton JW (2010) Analyzing protein structure and function using ancestral gene reconstruction. Current opinion in structural biology 20: 360–366 Available:http://www.pubmedcentral.nih.gov/articlerender.fcgi?artid=2916957&tool=pmcentrez&rendertype=abstract. Accessed 17 April 2012. 20413295 10.1016/j.sbi.2010.03.005 PMC2916957

[54] Prabhakar S, Visel A, Akiyama JA, Shoukry M, Lewis KD, et al (2008) Human-specific gain of function in a developmental enhancer. Science (New York, NY) 321: 1346–1350 Available:http://www.pubmedcentral.nih.gov/articlerender.fcgi?artid=2658639&tool=pmcentrez&rendertype=abstract. Accessed 30 July 2011. 10.1126/science.1159974 PMC2658639 18772437

[55] Andolfatto P (2005) Adaptive evolution of non-coding DNA in Drosophila. Nature 437: 1149–1152 Available:http://www.ncbi.nlm.nih.gov/pubmed/16237443. Accessed 16 July 2012. 16237443 10.1038/nature04107

[56] Richards S, Liu Y, Bettencourt BR, Hradecky P, Letovsky S, et al (2005) Comparative genome sequencing of Drosophila pseudoobscura: chromosomal, gene, and cis-element evolution. Genome research 15: 1–18 Available:http://www.pubmedcentral.nih.gov/articlerender.fcgi?artid=540289&tool=pmcentrez&rendertype=abstract. Accessed 12 March 2012. 15632085 10.1101/gr.3059305.125 PMC540289

[57] Shen Y, Yue F, McCleary DF, Ye Z, Edsall L, et al (2012) A map of the cis-regulatory sequences in the mouse genome. Nature 488: 116–20 Available:http://www.nature.com/doifinder/10.1038/nature11243. Accessed 2 July 2012. 22763441 10.1038/nature11243 PMC4041622

[58] Stephan W, Li H (2007) The recent demographic and adaptive history of Drosophila melanogaster. Heredity 98: 65–68 Available:http://www.ncbi.nlm.nih.gov/pubmed/17006533. Accessed 27 July 2012. 17006533 10.1038/sj.hdy.6800901

[59] Dawkins R (2004) The Ancestor's Tale. Boston: Houghton Mifflin. p.

[60] Rogers WA, Williams TM (2011) Quantitative Comparison of cis-Regulatory Element (CRE) Activities in Transgenic Drosophila melanogaster. Journal of visualized experiments 2–7 Available:http://www.ncbi.nlm.nih.gov/pubmed/22215325. Accessed 22 January 2012. 10.3791/3395 PMC3369668 22215325

[61] Erdman SE, Chen HJ, Burtis KC (1996) Functional and genetic characterization of the oligomerization and DNA binding properties of the Drosophila doublesex proteins. Genetics 144: 1639–1652 Available:http://www.pubmedcentral.nih.gov/articlerender.fcgi?artid=1207715&tool=pmcentrez&rendertype=abstract. 8978051 10.1093/genetics/144.4.1639 PMC1207715

[62] Wittkopp PJ, Carroll SB, Kopp A (2003) Evolution in black and white: genetic control of pigment patterns in Drosophila. Trends in Genetics 19: 495–504 Available:http://linkinghub.elsevier.com/retrieve/pii/S016895250300194X. Accessed 17 July 2011. 12957543 10.1016/S0168-9525(03)00194-X

[63] Stern DL (2010) Evolution, Development, & the Predictable Genome. 1st ed. Greenwood Village: Roberts & Company Publishers.

[64] Wittkopp PJ, True JR, Carroll SB (2002) Reciprocal functions of the Drosophila yellow and ebony proteins in the development and evolution of pigment patterns. Development (Cambridge, England) 129: 1849–1858 Available:http://www.ncbi.nlm.nih.gov/pubmed/11934851. 10.1242/dev.129.8.1849 11934851

[65] Gompel N, Prud'homme B, Wittkopp PJ, Kassner V a, Carroll SB (2005) Chance caught on the wing: cis-regulatory evolution and the origin of pigment patterns in Drosophila. Nature 433: 481–487 Available:http://www.ncbi.nlm.nih.gov/pubmed/15690032. 15690032 10.1038/nature03235

[66] Baker BS, Ridge KA (1980) Sex and the single cell. i. on the action of major loci affecting sex determination in. Genetics 94: 383–423. 6771185 10.1093/genetics/94.2.383 PMC1214149

[67] Stanojevic D, Small S, Levine M (1991) Regulation of a segmentation stripe by overlapping activators and repressors in the Drosophila embryo. Science 254: 1385–1387. 1683715 10.1126/science.1683715

[68] Small S, Blair a, Levine M (1992) Regulation of even-skipped stripe 2 in the Drosophila embryo. The EMBO journal 11: 4047–4057 Available:http://www.pubmedcentral.nih.gov/articlerender.fcgi?artid=556915&tool=pmcentrez&rendertype=abstract. 1327756 10.1002/j.1460-2075.1992.tb05498.x PMC556915

[69] Ludwig MZ, Patel NH, Kreitman M (1998) Functional analysis of eve stripe 2 enhancer evolution in Drosophila: rules governing conservation and change. Development (Cambridge, England) 125: 949–958 Available:http://www.ncbi.nlm.nih.gov/pubmed/9449677. 10.1242/dev.125.5.949 9449677

[70] Ludwig MZ, Bergman C, Patel NH, Kreitman M (2000) Evidence for stabilizing selection in a eukaryotic enhancer element. Nature 403: 564–567 Available:http://www.ncbi.nlm.nih.gov/pubmed/10676967. 10676967 10.1038/35000615

[71] Ludwig MZ, Palsson A, Alekseeva E, Bergman CM, Nathan J, et al (2005) Functional evolution of a cis-regulatory module. PLoS biology 3: e93 Available:http://www.pubmedcentral.nih.gov/articlerender.fcgi?artid=1064851&tool=pmcentrez&rendertype=abstract. Accessed 11 November 2012. 15757364 10.1371/journal.pbio.0030093 PMC1064851

[72] Swanson CI, Schwimmer DB, Barolo S (2011) Rapid Evolutionary Rewiring of a Structurally Constrained Eye Enhancer. Current biology 21: 1186–96 Available:http://www.ncbi.nlm.nih.gov/pubmed/21737276. Accessed 21 July 2011. 21737276 10.1016/j.cub.2011.05.056 PMC3143281

[73] Wray GA (2007) The evolutionary significance of cis-regulatory mutations. Nature reviews Genetics 8: 206–216 Available:http://www.ncbi.nlm.nih.gov/pubmed/17304246. Accessed 17 July 2011. 10.1038/nrg2063 17304246

[74] Rebeiz M, Williams TM (2011) Experimental Approaches to Evaluate the Contributions of Candidate Cis- regulatory Mutations to Phenotypic Evolution. In: Orgogozo V, Rockman M V., editors. Methods in Molecular Biology. Totowa, NJ: Humana Press, Vol. 772. pp. 351–375. Available:http://www.springerlink.com/index/10.1007/978-1-61779-228-1. Accessed 9 November 2011. 10.1007/978-1-61779-228-1_21 22065449

[75] Clark AG, Eisen MB, Smith DR, Bergman CM, Oliver B, et al (2007) Evolution of genes and genomes on the Drosophila phylogeny. Nature 450: 203–218 Available:http://www.ncbi.nlm.nih.gov/pubmed/17994087. Accessed 1 March 2012. 17994087 10.1038/nature06341

[76] Venken KJT, He Y, Hoskins R a, Bellen HJ (2006) P[acman]: a BAC transgenic platform for targeted insertion of large DNA fragments in D. melanogaster. Science (New York, NY) 314: 1747–1751 Available:http://www.ncbi.nlm.nih.gov/pubmed/17138868. Accessed 7 March 2012. 10.1126/science.1134426 17138868

[77] Groth AC, Fish M, Nusse R, Calos MP (2004) Construction of Transgenic Drosophila by Using the Site-Specific Integrase From Phage phiC31. Genetics 166: 1775–1782. 15126397 10.1534/genetics.166.4.1775 PMC1470814

[78] Staden R (1996) The Staden sequence analysis package. Molecular biotechnology 5: 233–241 Available:http://www.ncbi.nlm.nih.gov/pubmed/8837029. 8837029 10.1007/BF02900361

[79] Rebeiz M, Posakony JW (2004) GenePalette: a universal software tool for genome sequence visualization and analysis. Developmental biology 271: 431–438 doi:10.1016/j.ydbio.2004.04.011 15223345 10.1016/j.ydbio.2004.04.011

[80] Godt D, Couderc JL, Cramton SE, Laski FA (1993) Pattern formation in the limbs of Drosophila: bric à brac is expressed in both a gradient and a wave-like pattern and is required for specification and proper segmentation of the tarsus. Development (Cambridge, England) 119: 799–812 Available:http://www.ncbi.nlm.nih.gov/pubmed/7910551. 10.1242/dev.119.3.799 7910551

[81] Brudno M, Steinkamp R, Morgenstern B (2004) The CHAOS/DIALIGN WWW server for multiple alignment of genomic sequences. Nucleic acids research 32: W41–4 doi:10.1093/nar/gkh361 15215346 10.1093/nar/gkh361 PMC441499

[82] Pool JE, Aquadro CF (2007) The genetic basis of adaptive pigmentation variation in Drosophila melanogaster. Molecular ecology 16: 2844–2851 Available:http://www.pubmedcentral.nih.gov/articlerender.fcgi?artid=2650379&tool=pmcentrez&rendertype=abstract. Accessed 14 March 2012. 17614900 10.1111/j.1365-294X.2007.03324.x PMC2650379

[83] Ekker SC, Jackson DG, von Kessler DP, Sun BI, Young KE, et al (1994) The degree of variation in DNA sequence recognition among four Drosophila homeotic proteins. The EMBO journal 13: 3551–3560 Available:http://www.pubmedcentral.nih.gov/articlerender.fcgi?artid=395259&tool=pmcentrez&rendertype=abstract. 7914870 10.1002/j.1460-2075.1994.tb06662.x PMC395259

