# Supplementary figures and images for: Recurrent Modification of a Conserved Cis-Regulatory Element Underlies Fruit Fly Pigmentation Diversity
Source: PLoS Genet. 2013 Aug 29;9(8):e1003740. doi: 10.1371/journal.pgen.1003740 (PMC3757066; doi:10.1371/journal.pgen.1003740)

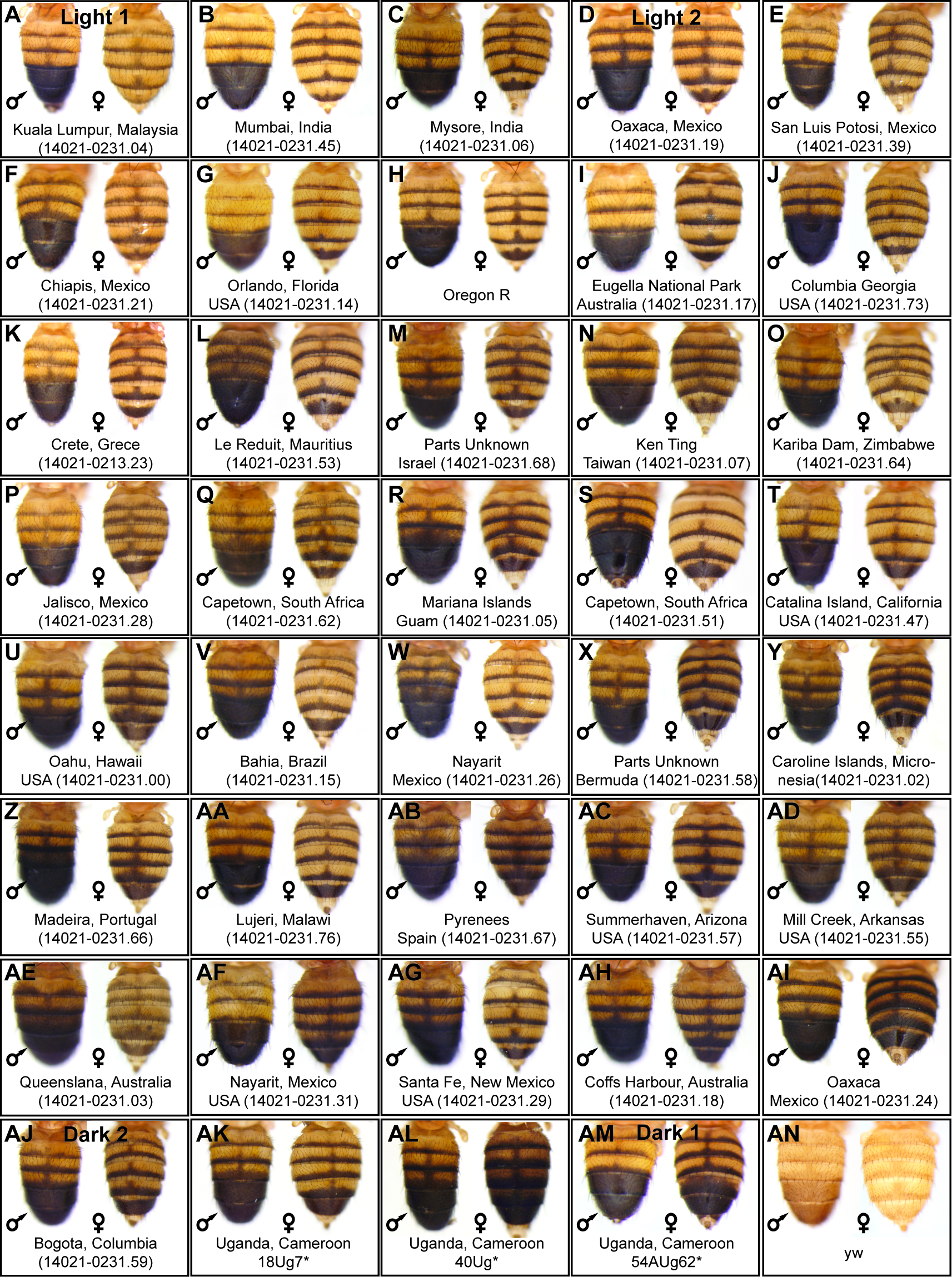

Supplement: Figure S1 — Abdomen pigmentation phenotypes for Drosophila melanogaster population stocks. (A-AN) Whole mount images of adult male and female dorsal abdomens. Geographic locations for the populations from which lab stocks were started are listed and, when applicable, in parentheses are the Drosophila Species Stock Center stock numbers. Representative images for the stocks referred to as (A) Light 1 population, (D) Light 2, (AM) Dark 1, and (AJ) Dark 2. (TIF) [file pgen.1003740.s001.tif]

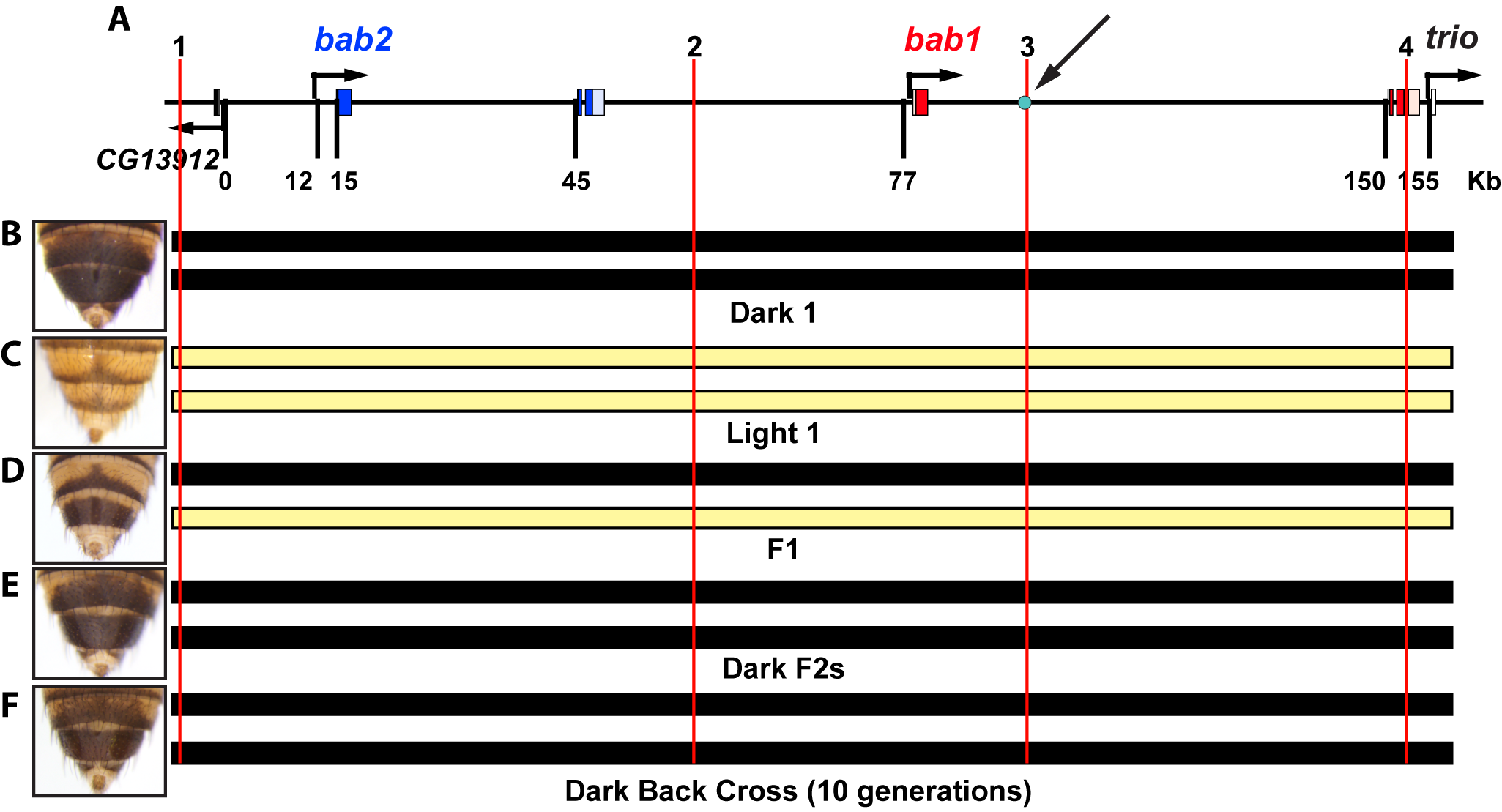

Supplement: Figure S3 — Mapping of the bab genotype-phenotype association. (A) To scale representation of the ∼155 kb bab locus, where the bab1 and bab2 genes are situated between the CG13912 and trio genes. Exons are indicated as the tall rectangles, and sites and directions for each gene's transcription are indicated by the black arrows. The location of polymorphic markers used to establish bab loci haplotypes are indicated by “1”, “2”, “3”, and “4”, and downward projecting red lines. Polymorphism 3 is the BstXI site polymorphism that resides within the Light 1 and Light 2 dimorphic element alleles. Blue dot with arrow indicates location of the dimorphic element. Representative female phenotypes (B–F) for the A5 and A6 segment tergites (Left) and the inferred bab locus haplotypes associated with the pigmentation phenotype (Right). (B) Dark 1 and (C) Light 1 specimens were homozygous for alternate nucleotide states at the four bab locus markers, establishing a Dark 1 and Light 1 haplotypes (Black and Yellow bars respectively). (D) Female F1 progeny from Dark 1 and Light 1 cross were heterozygous for bab locus markers. (E) Phenotypically Dark F2 progeny from parental Dark 1 and Light 1 cross were homozygous for the Dark 1 nucleotide state at each of the four evaluated bab locus markers. (F) Following 10 generations of backcrossing the Dark 1 phenotype into the Light 1 genetic background, a pure line was established where females exhibit the Dark 1 phenotype. This line was homozygous for the Dark 1 nucleotide state at each of the four evaluated bab locus markers. (TIF) [file pgen.1003740.s003.tif]

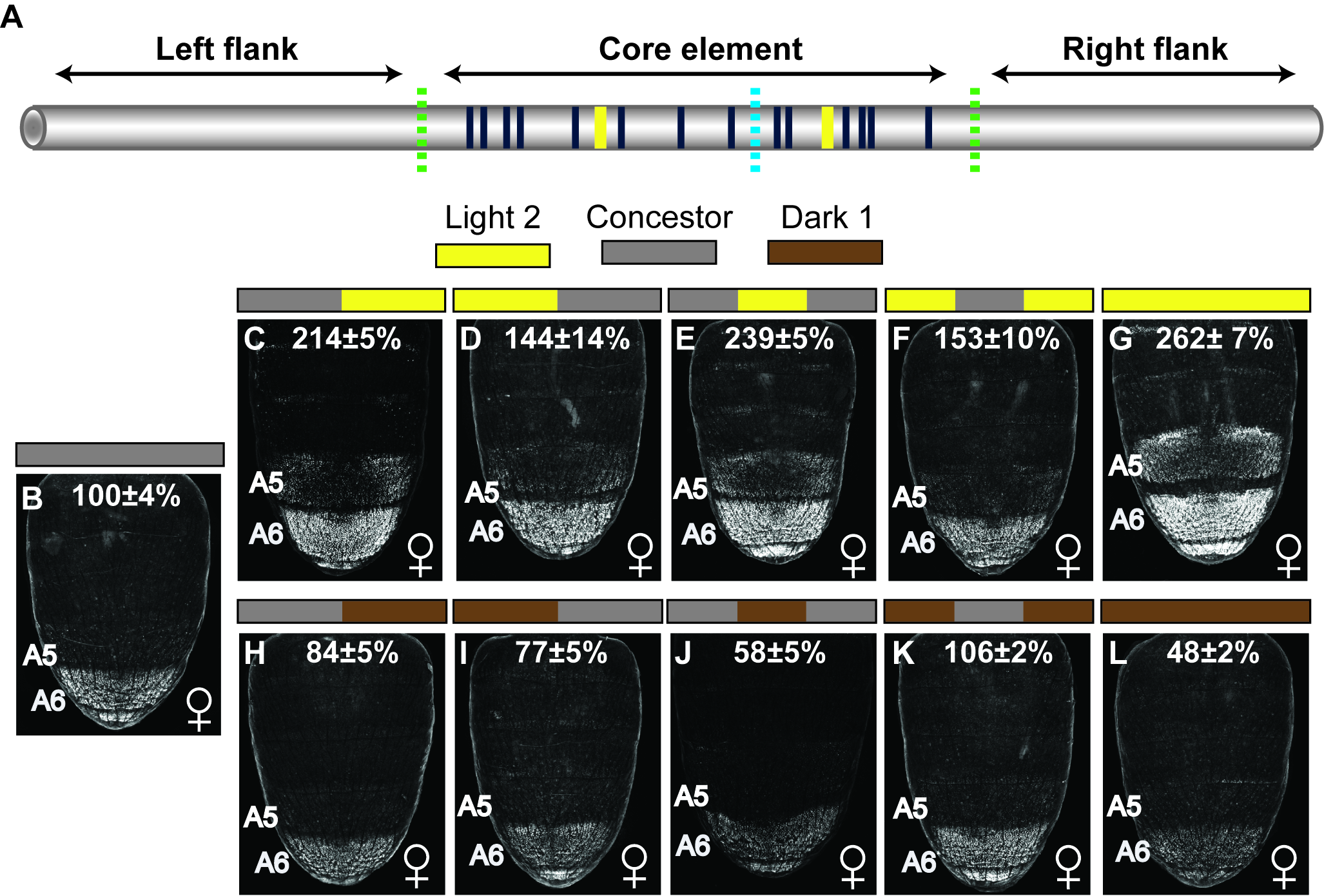

Supplement: Figure S5 — Chimeric dimorphic elements map functionally-relevant derived mutations to the core region. (A) To scale representation of the dimorphic element, with ABD-B and DSX binding sites shown as blue and yellow rectangles respectively. Green dashed lines indicate the positions where central core dimorphic element sequences were joined with flank sequences. Blue dashed line indicates the position where left and right halves of various dimorphic elements were joined. (B–L) GFP-reporter gene activity in female transgenic pupae at 85 hAPF. Activity measurements are represented as the % of the D. melanogaster Concestor element female A6 mean ± SEM. The illustration above each image indicates the sequence composition of the evaluated dimorphic elements. Gray, yellow, and brown colors respectively indicate sequence from the Concestor element, Light 2 dimorphic element, and the Dark 1 dimorphic element. (TIF) [file pgen.1003740.s005.tif]

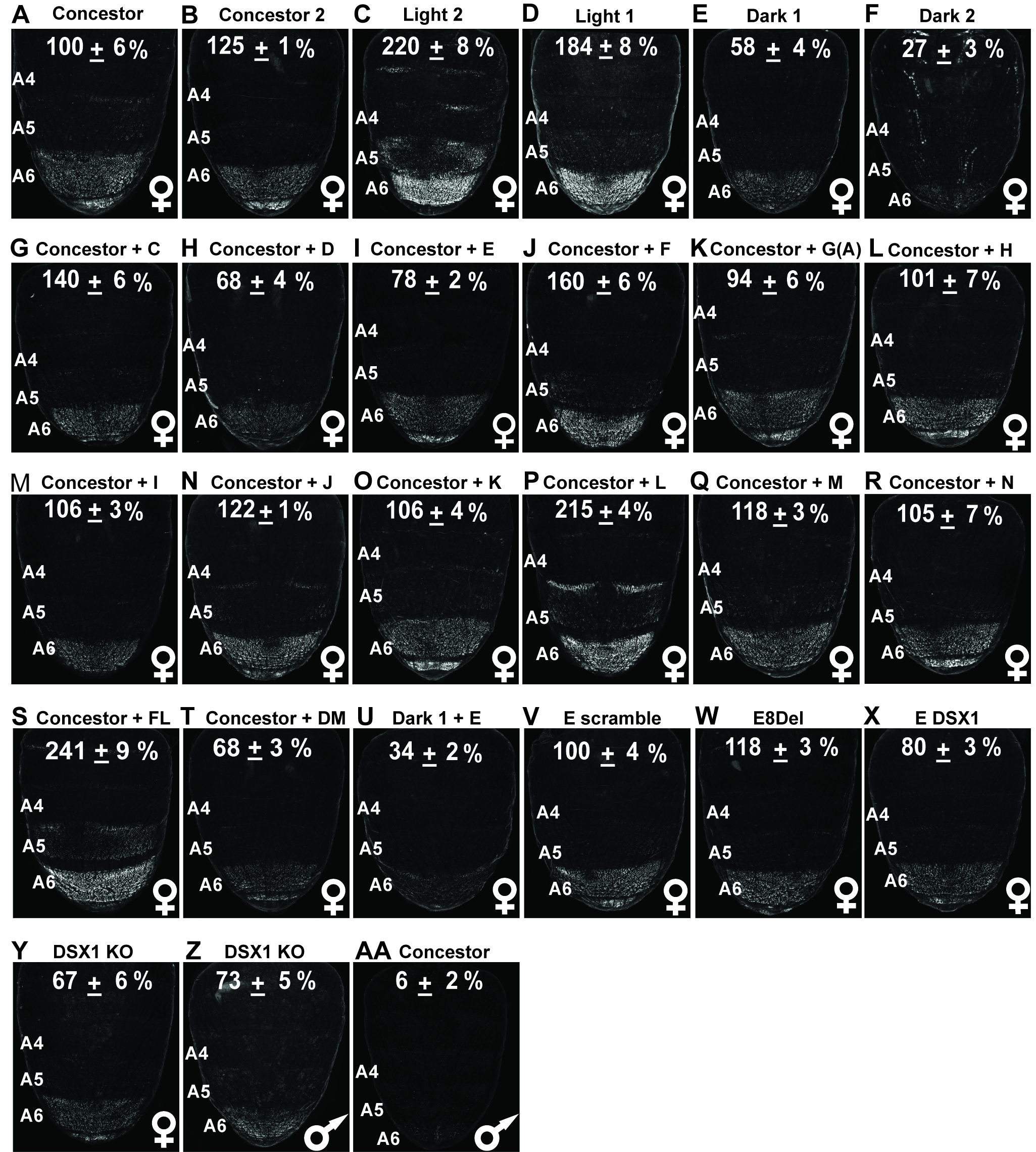

Supplement: Figure S6 — Regulatory activity effects of derived dimorphic element mutations. (A-AA) GFP-reporter gene activities in female transgenic pupae at 85 hAPF. (A) The Concestor element's mean activity measurement in the dorsal A6 segment was set as 100%, all other regulatory activities (B-AA) are reported as a percentage of the Concestor element's activity ± the standard error of the mean (SEM). For each reporter transgene, a representative image is presented. (C–F) Activities for population stock dimorphic element alleles. (G–R) Activities for Concestor elements with a substitution of a single mutation. (S and T) Activities for Concestor elements substituted with two Light 2 (S) and two Dark 1 (T) derived mutations. (U) The regulatory activity of the Dark 1 allele that included the E mutation. (V) The Concestor element's regulatory activity when the native sequence at the site of the E mutation was altered by non-complementary transversion at every 2nd base pair. (W) The Concestor element's regulatory activity when the first 8 of 9 base pairs of the E mutation were deleted. (X) The Concestor element's regulatory activity when only base pair 9 of the E mutation was deleted. (Y) The Concestor element's regulatory activity when the Dsx1 Site was mutated. (Z) The Concestor element's regulatory activity in males when the Dsx1 Site was mutated. (AA) The Concestor element's regulatory activity in males relative to its activity in females. (TIF) [file pgen.1003740.s006.tif]
