# Supplementary material for: Recurrent Modification of a Conserved Cis-Regulatory Element Underlies Fruit Fly Pigmentation Diversity
Source: PLoS Genet. 2013 Aug 29;9(8):e1003740. doi: 10.1371/journal.pgen.1003740 (PMC3757066; doi:10.1371/journal.pgen.1003740)
Supplement: Figure S2 — Sequence alignments for dimorphic elements. (A) Annotated alignment of dimorphic elements used to reconstruct ancestral sequences (Concestor and Concestor 2) from extant D. melanogaster populations. Dimorphic elements from D. mauritiana (mau.5), D. sechellia (sec.38), D. simulans (sim.33), D. yakuba (yak.25), D. lucipennis (luc.41), D. eugracilis (eug.20), and D. fuyamai (fuy.9) were used as out groups. (B) Annotated alignment of orthologous dimorphic elements from D. melanogaster Light 1 allele, D. yakuba (D. yak), D. fuyamai (D. fuy), and D. auraria (D. aur). White font on purple background indicates the AscI and SbfI restriction enzyme sites that were introduced for cloning purposes. Red font on black background indicates polymorphisms among the population-stock alleles. At the top of alignment is the number or letter designation assigned to each polymorphism. Ambiguous sites in the reconstructed concestor sequences are indicated by a gray background color. Characterized ABD-B and DSX binding sites are indicated respectively by white font on a blue background and black font on a yellow background. The BstXI restriction enzyme site used for genotyping is indicated by white font on a maroon background. (DOC) [file pgen.1003740.s002.doc]

**A.**

**AscI 1**

**"Concestor" ggcgcgccCA CATAAAAATC AGCAACAAAG TTGCTCTGGG CCCATAAAAG**

**"Concestor 2" ggcgcgccCA CATAAAAATC AGCAACAAAG TTGCCCTGGG CCCATAAAAG**

mel.00.1 GGCGCGCCCA CATAAAAATC AGCAACAAAG TTGCTCTGGC CCCATAAAAG

mel.01.58 GGCGCGCCCA CATAAAAATC AGCAACAAAG TTGCTCTGGC CCCATAAAAG

mel.02.7 GGCGCGCCCA CATAAAAATC AGCAACAAAC TTGCTCTGGC CCCATAAAAG

mel.04.1 (Light P1) GGCGCGCCCA CATAAAAATC AGCAACAAAG TTGCTCTGGC CCCATAAAAG

mel.05.13 GGCGCGCCCA CATAAAAATC AGCAACAAAG TTGCTCTGGC CCCATAAAAG

mel.05.16 GGCGCGCCCA CATAAAAATC AGCAACAAAC TTGCTCTGGC CCCATAAAAG

mel.07.20 GGCGCGCCCA CATAAAAATC AGCAACAAAG TTGCTCTGGC CCCATAAAAG

mel.14.26 GGCGCGCCCA CATAAAAATC AGCAACAAAG TTGCCCTGGC CCCATAAAAG

mel.17.66 GGCGCGCCCA CATAAAAATC AGCAACAAAG TTGCTCTGGC CCCATAAAAG

mel.18Ug.7 GGCGCGCCCA CATAAAAATC AGCAACAAAC TTGCCCTGGC CCCATAAAAG

mel.19.113 (Light P2) GGCGCGCCCA CATAAAAATC AGCAACAAAC TTGCTCTGGC CCCATAAAAG

mel.23.74 GGCGCGCCCA CATAAAAATC AGCAACAAAG TTGCTCTGGC CCCATAAAAG

mel.24.81 GGCGCGCCCA CATAAAAATC AGCAACAAAG TTGCTCTGGC CCCATAAAAG

mel.26.32 GGCGCGCCCA CATAAAAATC AGCAACAAAG TTGCCCTGGC CCCATAAAAG

mel.34.9 GGCGCGCCCA CATAAAAATC AGCAACAAAG TTGCTCTGGC CCCATAAAAG

mel.39.37 GGCGCGCCCA CATAAAAATC AGCAACAAAC TTGCTCTGGC CCCATAAAAG

mel.40Ug.12 GGCGCGCCCA CATAAAAATC AGCAACAAAG TTGCCCTGGC CCCATAAAAG

mel.45.89 GGCGCGCCCA CATAAAAATC AGCAACAAAG TTGCTCTGGC CCCATAAAAG

mel.CanS.41 GGCGCGCCCA CATAAAAATC AGCAACAAAG TTGCTCTGGC CCCATAAAAG

mel.53.105 GGCGCGCCCA CATAAAAATC AGCAACAAAG TTGCTCTGGC CCCATAAAAG

mel.54Ug.1 (Dark P1) GGCGCGCCCA CATAAAAATC AGCAACAAAG TTGCCCTGGC CCCATAAAAG

mel.59.3 (Dark P2) GGCGCGCCCA CATAAAAATC AGCAACAAAG TTGCTCTGGC CCCATAAAAG

mel.yw.50 GGCGCGCCCA CATAAAAATC AGCAACAAAG TTGCTCTGGC CCCATAAAAG

mel.WI83.25 GGCGCGCCCA CATAAAAATC AGCAACAAAG TTGCTCTGGC CCCATAAAAG

mel.51.100 GGCGCGCCCA CATAAAAATC AGCAACAAAC TTGCTCTGGC CCCATAAAAG

mel.21.2 GGCGCGCCCA CATAAAAATC AGCAACAAAC TTGCTCTGGC CCCATAAAAG

mel.29.3 GGCGCGCCCA CATAAAAATC AGCAACAAAG TTGCTCTGGC CCCATAAAAG

mel.64.3 GGCGCGCCCA CATAAAAATC AGCAACAAAG TTGCTCTGGC CCCATAAAAG

mel.67.1 GGCGCGCCCA CATAAAAATC AGCAACAAAG TTGCTCTGGC CCCATAAAAG

mel.55.2 GGCGCGCCCA CATAAAAATC AGCAACAAAC TTGCTCTGGC CCCATAAAAG

**Closely-Related**

**Outgroup Species**

mau.5 GGCGCGCCCA CATAAAAATC AGCAACAAAG TTGCCCTGGC CCCATAAAAG

sec.38 GGCGCGCCCA CATAAAAATC AGCAACAAAC TTGCCCTGGC CCCATAAAAG

sim.33 GGCGCGCCCA CATAAAAATC AGCAACAAA- TTGCCCTGGC CCCATAAAAG

**Outgroup Species**

yak.25 GGCGCGCCCA CATAAAAATC AGCAACAAAG TTGCCCTGGC CCCATAAAAA

luc.41 GGCGCGCCCA CATAAAAATC AGCAACAAAG TTGCCCTGGC CCCATAAAAA

eug.20 GGCGCGCCCA CATAAAAATC AGCAACAAAG TTGCCCT-GC CCCATAAAAA

fuy.9 GGCGCGCCCA CATAAAAATC AGCAACAAAG TTGCCCTGGC CCCATAAAAA

**2**

**"Concestor" 50 ATTGCAAACA AAAAC--AGA ACAACAGAAT GGCATGGAAT AAAA------**

**"Concestor 2" ATTGCAAACA AAAAC--AGA ACAACAGAAT GGCATGGAAT AAAA------**

mel.00.1 ATTGCAAACA AAAAC--AGA ACAACAGAAT GGCATGGAAT AAAA------

mel.01.58 ATTGCAAACA AAAAC--AGA ACAACAGAAT GGCATGGAAT AAAA------

mel.02.7 ATTGCAAACA AAAAC--AGA ACAACAGAAT GGCATGGAAT AAAA------

mel.04.1 ATTGCAAACA AAAAC--AGA ACAACAGAAT GGCATGGAAT AAAA------

mel.05.13 ATTGCAAACA AAAAC--AGA ACAACAGAAT GGCATGGAAT AAAA------

mel.05.16 ATTGCAAACA AAAAC--AGA ACAACAGAAT GGCATGGAAT AAAA------

mel.07.20 ATTGCAAACA AAAAC--AGA ACAACAGAAT GGCATGGAAT AAAA------

mel.14.26 ATTGCAAACA AAAAC--AGA AC-------- ---------- AAAA------

mel.17.66 ATTGCAAACA AAAAC--AGA ACAACAGAAT GGCATGGAAT AAAA------

mel.18Ug.7 ATTGCAAACA AAAAC--AGA ACAACAGAAT GGCATGGAAT AAAA------

mel.19.113 ATTGCAAACA AAAAC--AGA ACAACAGAAT GGCATGGAAT AAAA------

mel.23.74 ATTGCAAACA AAAAC--AGA ACAACAGAAT GGCATGGAAT AAAA------

mel.24.81 ATTGCAAACA AAAAC--AGA ACAACAGAAT GGGATGGAAT AAAA------

mel.26.32 ATTGCAAACA AAAAC--AGA ACAACAGAAT GGCATGGAAT AAAA------

mel.34.9 ATTGCAAACA AAAAC--AGA ACAACAGAAT GGCATGGAAT AAAA------

mel.39.37 ATTGCAAACA AAAAC--AGA ACAACAGAAT GGCATGGAAT AAAA------

mel.40Ug.12 ATTGCAAACA AAAAC--AGA ACAACAGAAT GGCATGGAAT AAAA------

mel.45.89 ATTGCAAACA AAAAC--AGA ACAACAGAAT GGCATGGAAT AAAA------

mel.CanS.41 ATTGCAAACA AAAAC--AGA ACAACAGAAT GGCATGGAAT AAAA------

mel.53.105 ATTGCAAACA AAAAC--AGA ACAACAGAAT GGCATGGAAT AAAA------

mel.54Ug.1 ATTGCAAACA AAAAC--AGA ACAACAGAAT GGCATGGAAT AAAA------

mel.59.3 ATTGCAAACA AAAAC--AGA ACAACAGAAT GGCATGGAAT AAAA------

mel.yw.50 ATTGCAAACA AAAAC--AGA ACAACAGAAT GGCATGGAAT AAAA------

mel.WI83.25 ATTGCAAACA AAAAC--AGA ACAACAGAAT GGCATGGAAT AAAA------

mel.51.100 ATTGCAAACA AAAAC—-AGA ACAACAGAAT GGCATGGAAT AAAA------

mel.21.2 ATTGCAAACA AAAAC--AGA ACAACAGAAT GGCATGGAAT AAAA------

mel.29.3 ATTGCAAACA AAAAC--AGA ACAACAGAAT GGCATGGAAT AAAA------

mel.64.3 ATTGCAAACA AAAAC--AGA ACAACAGAAT GGCATGGAAT AAAA------

mel.67.1 ATTGCAAACA AAAAC--AGA ACAACAGAAT GGCATGGAAT AAAA------

mel.55.2 ATTGCAAACA AAAAC--AGA ACAACAGAAT GGCATGGAAT AAAA------

**Closely-Related**

**Outgroup Species**

mau.5 ATTGCAAACA AAAAC--AGA ACAACAGAAT GGCATGGAAT AAAA------

sec.38 ATTGCAAACA AAAAC--AGA ACAACAGAAT GGCATGGAAT AAAA------

sim.33 ATTGCAAACA AAAAC--AGA ACAACAGAAT GGCATGGAAT AAAA------

**Outgroup Species**

yak.25 ATTGCAAACA AAAAC--AGA ACAAGCGGAA TGGCATGGAA TAAAA-----

luc.41 ATTGCAAACA AAAAACGAGA ACAACAGAAT GGCATGGAAT AAAA------

eug.20 ATGGCAAACA AAA---GAGA ACAACAGAAT GGCATGGAAT AAAA------

fuy.9 ATTGCAAACA AAAAG--AGA ACAAC--AAT GGCATGGAAT AAAA------

**"Concestor" 92 ---------- --TTTATATG AATAACAAAA AGCAGCTAAA GCA------A**

**"Concestor 2" ---------- --TTTATATG AATAACAAAA AGCAGCTAAA GCA------A**

mel.00.1 ---------- --TTTATATG AATAACAAAA AGCAGCTAAA GCA------A

mel.01.58 ---------- --TTTATATG AATAACAAAA AGCAGCTAAA GCA------A

mel.02.7 ---------- --TTTATATG AATAACAAAA AGCAGCTAAA GCA------A

mel.04.1 ---------- --TTTATATG AATAACAAAA AGCAGCTAAA GCA------A

mel.05.13 ---------- --TTTATATG AATAACAAAA AGCAGCTAAA GCA------A

mel.05.16 ---------- --TTTATATG AATAACAAAA AGCAGCTAAA GCA------A

mel.07.20 ---------- --TTTATATG AATAACAAAA AGCAGCTAAA GCA------A

mel.14.26 ---------- --TTTATATG AATAACAAAA AGCAGCTAAA GCA------A

mel.17.66 ---------- --TTTATATG AATAACAAAA AGCAGCTAAA GCA------A

mel.18Ug.7 ---------- --TTTATATG AATAACAAAA AGCAGCTAAA GCA------A

mel.19.113 ---------- --TTTATATG AATAACAAAA AGCAGCTAAA GCA------A

mel.23.74 ---------- --TTTATATG AATAACAAAA AGCAGCTAAA GCA------A

mel.24.81 ---------- --TTTATATG AATAACAAAA AGCAGCTAAA GCA------A

mel.26.32 ---------- --TTTATATG AATAACAAAA AGCAGCTAAA GCA------A

mel.34.9 ---------- --TTTATATG AATAACAAAA AGCAGCTAAA GCA------A

mel.39.37 ---------- --TTTATATG AATAACAAAA AGCAGCTAAA GCA------A

mel.40Ug.12 ---------- --TTTATATG AATAACAAAA AGCAGCTAAA GCA------A

mel.45.89 ---------- --TTTATATG AATAACAAAA AGCAGCTAAA GCA------A

mel.CanS.41 ---------- --TTTATATG AATAACAAAA AGCAGCTAAA GCA------A

mel.53.105 ---------- --TTTATATG AATAACAAAA AGCAGCTAAA GCA------A

mel.54Ug.1 ---------- --TTTATATG AATAACAAAA AGCAGCTAAA GCA------A

mel.59.3 ---------- --TTTATATG AATAACAAAA AGCAGCTAAA GCA------A

mel.yw.50 ---------- --TTTATATG AATAACAAAA AGCAGCTAAA GCA------A

mel.WI83.25 ---------- --TTTATATG AATAACAAAA AGCAGCTAAA GCA------A

mel.51.100 ---------- --TTTATATG AATAACAAAA AGCAGCTAAA GCA------A

mel.21.2 ---------- --TTTATATG AATAACAAAA AGCAGCTAAA GCA------A

mel.29.3 ---------- --TTTATATG AATAACAAAA AGCAGCTAAA GCA------A

mel.64.3 ---------- --TTTATATG AATAACAAAA AGCAGCTAAA GCA------A

mel.67.1 ---------- --TTTATATG AATAACAAAA AGCAGCTAAA GCA------A

mel.55.2 ---------- --TTTATATG AATAACAAAA AGCAGCTAAA GCA------A

**Closely-Related**

**Outgroup Species**

mau.5 ---------- --TTTATATG AATAACAAAA AGCAGCTAAA GCA------A

sec.38 ---------- --TTTATATG AATAACAAAA AGCAGCTAAA GCA------A

sim.33 ---------- --TTTATATG AATAACAAAA AGCAGCTAAA GCA------A

**Outgroup Species**

yak.25 ---------- --TTTATATG AATAACAAAA AGCAaaagca gctacagcaA

**Distantly-related**

**Outgroup Species**

luc.41 ---------- --TTTATATG AATAACAAAA AGCAGCTAAA AGAAGC---A

eug.20 ---------- --TTTATATG AATAACAAAA AGCAGCTAAA AGAAGC---A

fuy.9 ---------- --TTTATATG AATAACAAAA AGCAGCTAAA AGAAAC---A

**34** **5**

**"Concestor" 124 GCAGCAACAA CAACAG---- --TTTACTGC CCCGGCTCAG CGGTACACTG**

**"Concestor2" GCAGCAACAA CAACAG---- --TTTACTGC CCCGGCTCAG CGGTACACTG**

mel.00.1 GCAGCAACAA CAA**T**AG---- --TTTACTGC CCCGGCTCAG CGGTACACTG

mel.01.58 GCAGCAACAA CAA**T**AG---- --TTTACTGC CCCGGCTCAG CGGTACACTG

mel.02.7 GCAGCAACAA CAA**T**AG---- --TTTACTGC CCCGGCTCAG CGGTACACTG

mel.04.1 GCAGCAACAA CAA**T**AG---- --TTTACTGC CCCGGCTCAG CGGTACACTG

mel.05.13 GCAGCAACAA CAA**T**AG---- --TTTACTGC CCCGGCTCAG CGGTACACTG

mel.05.16 GCAGCAACAA CAA**T**AG---- --TTTACTGC CCCGGCTCAG CGGTACACTG

mel.07.20 GCAGCAACAA CAA**T**AG---- --TTTACTGC CCCGGCTCAG CGGTACACTG

mel.14.26 GCAGCAACAA CAA**T**AG---- --TTTACTGC CCCGGCTCAG CGGTACACTG

mel.17.66 GCAGCAACAA CAA**T**AG---- --TTTACTGC CCCGGCTCAG CGGTACACTG

mel.18Ug.7 GCAGCAACAA CAA**T**AG---- --TTTACTGC CCCGGCTCAG CGGTACACTG

mel.19.113 GCAGCAACAA CAA**T**AG---- --TTTACTGC CCCGGCTCAG CGGTACACTG

mel.23.74 GCAGCAACAA CAA**T**AG---- --TTTACTGC CCCGGCTCAG CGGTACACTG

mel.24.81 GCAGCAACAA CAA**T**AG---- --TTTACTGC CCCGGCTCAG CGGTACACTG

mel.26.32 GCAGCAACAA CAACAG---- --TTTACTGC CCCGGCTCAG CGGTACACTG

mel.34.9 GCAGCAACAA CAA**T**AG---- --TTTACTGC CCCGGCTCAG CGGTACACTG

mel.39.37 GCAGCAACAA CAA**T**AG---- --TTTACTGC CCCGGCTCAG CGGTACACTG

mel.40Ug.12 GCAGCAACAA CAA**T**AG---- --TTTACTGC CCCGGCTCAG CGGTACACTG

mel.45.89 GCAGCAACAA CAA**T**AG---- --TTTACTGC CCCGGCTCAG CGGTACACTG

mel.CanS.41 GCAGCAACAA CAA**T**AG---- --TTTACTGC CCCGGCTCAG CGGTACACTG

mel.53.105 GCAGCAACAA CAA**T**AG---- --TTTACTGC CCCGGCTCAG CGGTACACTG

mel.54Ug.1 GCAGCAACAA CAACAG---- --TTTACTGC CCCGGCTCAG CGGTACACTG

mel.59.3 GCAGCAACAA CA**T**CAG---- --TTTACTGC CCCGTCTCAG CGGTACACTG

mel.yw.50 GCAGCAACAA CAA**T**AG---- --TTTACTGC CCCGGCTCAG CGGTACACTG

mel.WI83.25 GCAGCAACAA CAA**T**AG---- --TTTACTGC CCCGGCTCAG CGGTACACTG

mel.51.100 GCAGCAACAA CAACAG---- --TTTACTGC CCCGGCTCAG CGGTACACTG

mel.21.2 GCAGCAACAA CAATAG---- --TTTACTGC CCCGGCTCAG CGGTACACTG

mel.29.3 GCAGCAACAA CAATAG---- --TTTACTGC CCCGGCTCAG CGGTACACTG

mel.64.3 GCAGCAACAA CAACAG---- --TTTACTGC CCCGGCTCAG CGGTACACTG

mel.67.1 GCAGCAACAA CAATAG---- --TTTACTGC CCCGGCTCAG CGGTACACTG

mel.55.2 GCAGCAACAA CAATAG---- --TTTACTGC CCCGGCTCAG CGGTACACTG

**Closely-Related**

**Outgroup Species**

mau.5 GCAGCAACAA CAA**C**AG---- --TTTACTGC CCCG**G**CTCAG AGGTACACTG

sec.38 GCAACAACAA CAA**C**AACAAC AGTTTACTGC CCCG**G**CTCAG AGATACACTG

sim.33 GCAGCAACAA CAA**C**AG---- --TTTACTGC CCCG**G**CTCAG AGGTACACTG

**Outgroup Species**

yak.25 GCGGCAACAA CAACAG---- --TTTACTGC CCCGGCTTAG TGGTACACTG

**Distantly-related**

**Outgroup Species**

luc.41 ACAACAACA- -----G---- --TTTATTGC CCTGGCTCAG CTGAACACTG

eug.20 GCAACAACAA CAACAG---- --TTTACTGC CCTGCCTTGG CCGTGCACTG

fuy.9 GCAGCAACAA CAACAG---- --TTTACTGC TCTGGCTCAG CAGTACACTG

**6 7 8**

**"Concestor" 168 TGCAAAACGA TTGTACTCCT CCTCATAATA ATAAGAGTA- ----------**

**"Concestor2" TGCAAAACGA TTGTACTCCT CCTCATAATA ATAAGAGTA- ----------**

mel.00.1 TGCAAAACG**-** TTGTACTCCT CCTCATAATA ATA**T**GAGTA- ----------

mel.01.58 TGCAAAACG**-** TTGTACTCCT CCTCATAATA ATA**T**GAGTA- ----------

mel.02.7 TGCAAAACG**-** TTGTACTCCT CCTCATAATA ATA**T**GAGTA- ----------

mel.04.1 TGCAAAACG**-** TTGTACTCCT CCTCATAATA ATA**T**GAGTA- ----------

mel.04.7 TGCAAAACG**-** TTGTACTCCT CCTCATAATA ATA**T**GAGTA- ----------

mel.05.13 TGCAAAACG**-** TTGTACTCCT CCTCATAATA ATA**T**GAGTA- ----------

mel.05.16 TGCAAAACG**-** TTGTACTCCT CCTCATAATA ATA**T**GAGTA- ----------

mel.07.20 TGCAAAACG**-** TTGTACTCCT CCTCATAATA ATA**T**GAGTA- ----------

mel.14.26 TGCAAAACGA TTGTACTCCT CCTCATAATA ATA**T**GAGTA- ----------

mel.17.66 TGCAAAACG**-** TTGTACTCCT CCTCATAATA ATA**T**GAGTA- ----------

mel.18Ug.7 TGCAAAACGA TTGTACTCCT CCTCATAATA ATA**T**GAGTA- ----------

mel.19.113 TGCAAAACG**-** TTGTACTCCT CCTCATAATA ATA**T**GAGTA- ----------

mel.23.74 TGCAAAACG**-** TTGTACTCCT CCTCATAATA ATA**T**GAGTA- ----------

mel.24.81 TGCAAAACG**-** TTGTACTCCT CCTCATAATA ATA**T**GAGTA- ----------

mel.26.32 TGCAAAACGA TTGTACTCCT CCTCATAATA ATAAGTATA- ----------

mel.34.9 TGCAAAACG**-** TTGTACTCCT CCTCATAATA ATAAGTATA- ----------

mel.39.37 TGCAAAACG**-** TTGTACTCCT CCTCATAATA ATA**T**GAGTA- ----------

mel.40Ug.12 TGCAAAACGA TTGTACTCCT CCTCATAATA ATA**T**GAGTA- ----------

mel.45.89 TGCAAAACG**-** TTGTACTCCT CCTCATAATA ATA**T**GAGTA- ----------

mel.CanS.41 TGCAAAACG**-** TTGTACTCCT CCTCATAATA ATA**T**GAGTA- ----------

mel.53.105 TGCAAAACG**-** TTGTACTCCT CCTCATAATA ATA**T**GAGTA- ----------

mel.54Ug.1 TGCAAAACGA TTGTACTCCT CCTCATAATA ATAAG**TA**TA- ----------

mel.59.3 TGCAAAATGA TTGTACTCCT CCTCATAATA ATA**T**GAGTA- ----------

mel.yw.50 TGCAAAACG**-** TTGTACTCCT CCTCATAATA ATA**T**GAGTA- ----------

mel.WI83.25 TGCAAAACG**-** TTGTACTCCT CCTCATAATA ATA**T**GAGTA- ----------

mel.51.100 TGCAAAACGA TTGTACTCCT CCTCATAATA ATA**T**GAGTA- ----------

mel.21.2 TGCAAAACG**-** TTGTACTCCT CCTCATAATA ATA**T**GAGTA- ----------

mel.29.3 TGCAAAACG**-** TTGTACTCCT CCTCATAATA ATA**A**GTATA- ----------

mel.64.3 TGCAAAACGA TTGTACTCCT CCTCATAATA ATA**A**GAGTA- ----------

mel.67.1 TGCAAAACG**-** TTGTACTCCT CCTCATAATA ATA**T**GAGTA- ----------

mel.55.2 TGCAAAACG**-** TTGTACTCCT CCTCATAATA ATA**T**GAGTA- ----------

**Closely-Related**

**Outgroup Species**

mau.5 AGCAAAATG**A** ATGTACTCTT TTTCATACCA ATAACAGGAA G---------

sec.38 TGCAAAATG**A** GTGTGTTCCT CCTCATACCA ATAACAGAAA GTTCT-----

sim.33 AGCAAAATG**A** TTGTGCTCCT CCTCATACCA ATAA------ ---CT-----

**Outgroup Species**

yak.25 TACGAAATA- Aaataactcc ctctcattaa ATAAAAGTAA actaaatcac

**Distantly-related**

**Outgroup Species**

luc.41 AGGAAAATAg ttatgggatt tttga----- ---------- ----------

eug.20 TGGGAAAata tcacttttat ggtctcctta atatttgcca tctctttagc

fuy.9 TGGAAAATA- TTGataccat tcttttttat atccataata aaggccaata

**9 10 11 12**

**"Concestor" 207 ---------- -----TATAA AGTATATAAT ATACTATATA TCACCATTGA**

**"Concestor2" ---------- -----TATAA AGTATATAAT ATACTATATA TCTCCATTGA**

mel.00.1 ---------- -----TATA**G** AGTATATAAT ATACTATATA TC**T**CCATTGA

mel.01.58 ---------- -----TATA**G** AGTATATAAT ATACTATATA TC**T**CCATTGA

mel.02.7 ---------- -----TATA**G** AGTATATAAT ATACTATATA TC**T**CCATTGA

mel.04.1 ---------- -----TATA**G** AGTATATAAT ATACTATATA TC**T**CCATTGA

mel.04.7 ---------- -----TATA**G** AGTATATAAT ATACTATATA TC**T**CCATTGA

mel.05.13 ---------- -----TATA**G** AGTATATAAT ATACTATATA TC**T**CCATTGA

mel.05.16 ---------- -----TATA**G** AGTATATAAT ATACTATATA TC**T**CCATTGA

mel.07.20 ---------- -----TATA**G** AGTATATAAT ATACTATATA TC**T**CCATTGA

mel.14.26 ---------- -----TATA**G** AGTATATAAT ATACTATATA TCACCATTGA

mel.17.66 ---------- -----TATA**G** AGTATATAAT ATACTATATA TC**T**CCATTGA

mel.18Ug.7 ---------- -----TATA**G** AGTATATAAT ATACTATATA TCACCATTGA

mel.19.113 ---------- -----TATA**G** AGTATATAAT ATACTATATA TC**C**CCATTGA

mel.23.74 ---------- -----TATA**G** AGTATATAAT ATACTATATA TC**T**CCATTGA

mel.24.81 ---------- -----TATA**G** AGTATATAAT ATACTATATA TC**T**CCATTTA

mel.26.32 ---------- -----TATAC A--TATATAT ATAATATATA TCACCATTGA

mel.34.9 ---------- -----TATAT ---ATAT-AT ATACTATATA TCACCATTGA

mel.39.37 ---------- -----TATA**G** AGTATATAAT ATACTATATA TC**T**CCATTGA

mel.40Ug.12 ---------- -----TATA**G** AGTATATAAT ATACTATATA TCACCATTGA

mel.45.89 ---------- -----TATA**G** AGTATATAAT ATACTATATA TC**T**CCATTGA

mel.CanS.41 ---------- -----TATA**G** AGTATATAAT ATACTATATA TC**T**CCATTGA

mel.53.105 ---------- -----TATA**G** AGTATATAAT ATACTATATA TC**T**CCATTGA

mel.54Ug.1 ---------- -----TATA**T** **---**ATAT**-**AT ATACTATATA TCACCATTGA

mel.59.3 ---------- -----TATA**G** AGTATATAAT ATACTATATA TCACCATTGA

mel.yw.50 ---------- -----TATA**G** AGTATATAAT ATACTATATA TC**T**CCATTGA

mel.WI83.25 ---------- -----TATAG AGTATATAAT ATACTATATA TC**T**CCATTGA

mel.51.100 ---------- -----TATAG AGTATATAAT ATACTATATA TCACCATTGA

mel.21.2 ---------- -----TATA- --TATA---T ATACTATATA TC**T**CCATTGA

mel.29.3 ---------- -----TATAG AGTATATAAT ATACTATATA TCACCATTGA

mel.64.3 ---------- -----TATAA AGTATATAAT ATACTATATA TC**T**CCATTGA

mel.67.1 ---------- -----TATAG AGTATATAAT ATACTATATA TC**T**CCATTGA

mel.55.2 ---------- -----TATAG AGTATATAAT ATACTATATA TC**T**CCATTGA

**Closely-Related**

**Outgroup Species**

mau.5 TAGTTAATAT GAAAGTATAA AGTAAATATC ATACTATATA TCTCTATAGA

sec.38 ----TAATAT AAAAGTATAA AGTAAATAAC ATACTATATA TCTCTATAGA

sim.33 ----TAATAT AAAAGTATAA AGTAAATAAC CTACCATATA TCTCTATAGA

**Outgroup Species**

yak.25 acgcaagctt tgtaaataat cggtactaca tcctagta-- ----------

**Distantly-related**

**Outgroup Species**

luc.41 ---------- AAATGTAACA ATTAAATAAT ATAATATTTA gggtcatcat

eug.20 cgaggct--- ---------- ---------- ---------- --------TG

fuy.9 gagtattttt actgcatgat agtatttggg agctcataat ttgtaaacTG

**13**

**"Concestor" 242 TAATTTCGAT CATTTTCACC T--------- ---------- ----------**

**"Concestor2" TAATTTCGAT CATTTTCACC T--------- ---------- ----------**

mel.00.1 TAATTGCGAT CATTTTCACC T--------- ---------- ----------

mel.01.58 TAATTTCGAT CATTTTCACC T--------- ---------- ----------

mel.02.7 TAATTTCGAT CATTTTCACC T--------- ---------- ----------

mel.04.1 TAATTTCGAT CATTTTCACC T--------- ---------- ----------

mel.04.7 TAATTTCGAT CATTTTCACC T--------- ---------- ----------

mel.05.13 TAATTTCGAT CATTTTCACC T--------- ---------- ----------

mel.05.16 TAATTTCGAT CATTTTCACC T--------- ---------- ----------

mel.07.20 TAATTTCGAT CATTTTCACC T--------- ---------- ----------

mel.14.26 TAATTTCGAT CATTTTCACC T--------- ---------- ----------

mel.17.66 TAATTTCGAT CATTTTCACC T--------- ---------- ----------

mel.18Ug.7 TAATTTCGAT CATTTTCACC T--------- ---------- ----------

mel.19.113 TAATTTCGAT CATTTTCACC T--------- ---------- ----------

mel.23.74 TAATTTCGAT CATTTTCACC T--------- ---------- ----------

mel.24.81 TAATGTCGAT CATTTTCACC T--------- ---------- ----------

mel.26.32 TA-------- ---------- ---------- ---------- ----------

mel.34.9 TAATTTCGAT CATTTTCACC T--------- ---------- ----------

mel.39.37 TAATTTCGAT CATTTTCACC T--------- ---------- ----------

mel.40Ug.12 TAATTTCGAT CATTTTCACC T--------- ---------- ----------

mel.45.89 TAATTTCGAT CATTTTCACC T--------- ---------- ----------

mel.CanS.41 TAATTTCGAT CATTTTCACC T--------- ---------- ----------

mel.53.105 TAATTTCGAT CATTTTCACC T--------- ---------- ----------

mel.54Ug.1 TAATTTCGAT CATTTTCACC T--------- ---------- ----------

mel.59.3 TAATTTCGAT CATTTTCACC T--------- ---------- ----------

mel.yw.50 TAATTTCGAT CATTTTCACC T--------- ---------- ----------

mel.WI83.25 TAATTTCGAT CATTTTCACC T--------- ---------- ----------

mel.51.100 TAATTTCGAT CATTTTCACC T--------- ---------- ----------

mel.21.2 TAATTTCGAT CATTTTCACC T--------- ---------- ----------

mel.29.3 TAATTTCGAT CATTTTCACC T--------- ---------- ----------

mel.64.3 TAATTTCGAT CATTTTCACC T--------- ---------- ----------

mel.67.1 TAATTTCGAT CATTTTCACC T--------- ---------- ----------

mel.55.2 TAATTTCGAT CATTTTCACC T--------- ---------- ----------

**Closely-Related**

**Outgroup Species**

mau.5 TAGTTTCATC ACCTTTTTTT CACCT----- ---------- ----------

sec.38 TAGTTTCATC GTCTTTTTTT CACCT----- ---------- ----------

sim.33 TAGTTTCATC -----TTTTT CAACCT---- ---------- ----------

**Outgroup Species**

yak.25 ---------- ---------- ----TA---- ---------- ----------

**Distantly-related**

**Outgroup Species**

luc.41 gattgaaggt ttgaaggttt caattgaaag ataaaatctt taattttgta

eug.20 GAAACAAGTT TGTATCTGTT TTTCAtaaga atcgtatcag atttgcctga

fuy.9 AAAACAAGTT TGCTTTGGTT CTTTAgggaa gaaaaaaagg agcttttaaa

**"Concestor" 263 ---------- ---------- ---------- ---------- ----------**

**"Concestor2" ---------- ---------- ---------- ---------- ----------**

mel.00.1 ---------- ---------- ---------- ---------- ----------

mel.01.58 ---------- ---------- ---------- ---------- ----------

mel.02.7 ---------- ---------- ---------- ---------- ----------

mel.04.1 ---------- ---------- ---------- ---------- ----------

mel.04.7 ---------- ---------- ---------- ---------- ----------

mel.05.13 ---------- ---------- ---------- ---------- ----------

mel.05.16 ---------- ---------- ---------- ---------- ----------

mel.07.20 ---------- ---------- ---------- ---------- ----------

mel.14.26 ---------- ---------- ---------- ---------- ----------

mel.17.66 ---------- ---------- ---------- ---------- ----------

mel.18Ug.7 ---------- ---------- ---------- ---------- ----------

mel.19.113 ---------- ---------- ---------- ---------- ----------

mel.23.74 ---------- ---------- ---------- ---------- ----------

mel.24.81 ---------- ---------- ---------- ---------- ----------

mel.26.32 ---------- ---------- ---------- ---------- ----------

mel.34.9 ---------- ---------- ---------- ---------- ----------

mel.39.37 ---------- ---------- ---------- ---------- ----------

mel.40Ug.12 ---------- ---------- ---------- ---------- ----------

mel.45.89 ---------- ---------- ---------- ---------- ----------

mel.CanS.41 ---------- ---------- ---------- ---------- ----------

mel.53.105 ---------- ---------- ---------- ---------- ----------

mel.54Ug.1 ---------- ---------- ---------- ---------- ----------

mel.59.3 ---------- ---------- ---------- ---------- ----------

mel.yw.50 ---------- ---------- ---------- ---------- ----------

mel.WI83.25 ---------- ---------- ---------- ---------- ----------

mel.51.100 ---------- ---------- ---------- ---------- ----------

mel.21.2 ---------- ---------- ---------- ---------- ----------

mel.29.3 ---------- ---------- ---------- ---------- ----------

mel.64.3 ---------- ---------- ---------- ---------- ----------

mel.67.1 ---------- ---------- ---------- ---------- ----------

mel.55.2 ---------- ---------- ---------- ---------- ----------

**Closely-Related**

**Outgroup Species**

mau.5 ---------- ---------- ---------- ---------- ----------

sec.38 ---------- ---------- ---------- ---------- ----------

sim.33 ---------- ---------- ---------- ---------- ----------

**Outgroup Species**

yak.25 264 ---------- ---------- ---------- ---------- ----------

**Distantly-related**

**Outgroup Species**

luc.41 ataggaaaaa atataatttt ctacaaatat ttgaacaatt atttgttaaa

eug.20 tctggcactt aagatacttt tctgaacaaa ctttaagttt t---------

fuy.9 tttaaaatat cattgccatt agaacaggaa aaactactta atatttgtta

**14**

**"Concestor" 263 ---------- ---------- ---------- ---------- ---TTTAACT**

**"Concestor2" ---------- ---------- ---------- ---------- ---TTTAACT**

mel.00.1 ---------- ---------- ---------- ---------- ---TTTAACT

mel.01.58 ---------- ---------- ---------- ---------- ---TTTAACT

mel.02.7 ---------- ---------- ---------- ---------- ---TTTAACT

mel.04.1 ---------- ---------- ---------- ---------- ---TTTAACT

mel.04.7 ---------- ---------- ---------- ---------- ---TTTAACT

mel.05.13 ---------- ---------- ---------- ---------- ---TTTAACT

mel.05.16 ---------- ---------- ---------- ---------- ---TTTAACT

mel.07.20 ---------- ---------- ---------- ---------- ---TTTAACT

mel.14.26 ---------- ---------- ---------- ---------- ---TTTAACT

mel.17.66 ---------- ---------- ---------- ---------- ---TTTAACT

mel.18Ug.7 ---------- ---------- ---------- ---------- ---TTTAACA

mel.19.113 ---------- ---------- ---------- ---------- ---TTTAACT

mel.23.74 ---------- ---------- ---------- ---------- ---TTTAACT

mel.24.81 ---------- ---------- ---------- ---------- ---TTTAACT

mel.26.32 ---------- ---------- ---------- ---------- ---TTTAACT

mel.34.9 ---------- ---------- ---------- ---------- ---TTTAACT

mel.39.37 ---------- ---------- ---------- ---------- ---TTTAACT

mel.40Ug.12 ---------- ---------- ---------- ---------- ---TTTAACA

mel.45.89 ---------- ---------- ---------- ---------- ---TTTAACT

mel.CanS.41 ---------- ---------- ---------- ---------- ---TTTAACT

mel.53.105 ---------- ---------- ---------- ---------- ---TTTAACT

mel.54Ug.1 ---------- ---------- ---------- ---------- ---TTTAACT

mel.59.3 ---------- ---------- ---------- ---------- ---TTTAACT

mel.yw.50 ---------- ---------- ---------- ---------- ---TTTAACT

mel.WI83.25 ---------- ---------- ---------- ---------- ---TTTAACT

mel.51.100 ---------- ---------- ---------- ---------- ---TTTAACT

mel.21.2 ---------- ---------- ---------- ---------- ---TTTAACT

mel.29.3 ---------- ---------- ---------- ---------- ---TTTAACT

mel.64.3 ---------- ---------- ---------- ---------- ---TTTAACT

mel.67.1 ---------- ---------- ---------- ---------- ---TTTAACT

mel.55.2 ---------- ---------- ---------- ---------- ---TTTAACT

**Closely-Related**

**Outgroup Species**

mau.5 ---------- ---------- ---------- ---------- ----------

sec.38 ---------- ---------- ---------- ---------- ----------

sim.33 ---------- ---------- ---------- ---------- ----------

**Outgroup Species ***

yak.25 ---------- ---------- ---------- ---------- - GTTTATTT

**Distantly-related**

**Outgroup Species ***

luc.41 attatatttc aaaggatatt attctTAGAA ATCCCCTTTG ATATTTATTT

eug.20 ---------- ---------- ---------- ---------- ----------

fuy.9 agcct----- ---------- ---------- ---------- ----------

**15 16 17**

**"Concestor" 270 AATTTATGCC CAATATAGTT G--------- ---------- ----------**

**"Concestor2" AATTTATGCC CAATGTAGTT G--------- ---------- ----------**

mel.00.1 AATTTATGCC CAATATAGTT G--------- ---------- ----------

mel.01.58 AATTTATGCC CAATATAGTT G--------- ---------- ----------

mel.02.7 AATTTATGCC CAATATAGTT G--------- ---------- ----------

mel.04.1 AATTTATGCC CAAT**G**TAGTT G--------- ---------- ----------

mel.04.7 AATTTATGCC CAAT**G**TAGTT G--------- ---------- ----------

mel.05.13 AATTTATGCC CAATATAGTT G--------- ---------- ----------

mel.05.16 AATTTATGCC CAATATAGTT G--------- ---------- ----------

mel.07.20 AATTTATGCC CAATATAGTT G--------- ---------- ----------

mel.14.26 AATTTATGCC CAATATAGTT G--------- ---------- ----------

mel.17.66 AATTTATGCC CAATATAGTT G--------- ---------- ----------

mel.18Ug.7 **G**ATTTATGCC CAATATAGTT G--------- ---------- ----------

mel.19.113 AATTTATGCC CAATATAGTT G--------- ---------- ----------

mel.23.74 AATTTATGCC CAATATAGTT G--------- ---------- ----------

mel.24.81 AATTTATGCC CAAT**G**TAGTT G--------- ---------- ----------

mel.26.32 AATTTATGCC CAAT**G**TAGTT G--------- ---------- ----------

mel.34.9 AATTTATG-- -----TAGTT G--------- ---------- ----------

mel.39.37 AATTTATGCC CAATATAGTT G--------- ---------- ----------

mel.40Ug.12 **G**ATTTATGCC CAATATAGTT G--------- ---------- ----------

mel.45.89 AATTTATGCC CAATATAGTT G--------- ---------- ----------

mel.CanS.41 AATTTATGCC CAATATAGTT G--------- ---------- ----------

mel.53.105 AATTTATGCC CAATATAGTT G--------- ---------- ----------

mel.54Ug.1 AATTTATGCC CAAT**G**TAGTT G--------- ---------- ----------

mel.59.3 AATTTATGCC CAATATAGTT G--------- ---------- ----------

mel.yw.50 AATTTATGCC CAATATAGTT G--------- ---------- ----------

mel.WI83.25 AATTTATGCC CAATATAGTT G--------- ---------- ----------

mel.51.100 AATTTATGCC CAATATAGTT G--------- ---------- ----------

mel.21.2 AATTTATGCC CAATATAGTT G--------- ---------- ----------

mel.29.3 AATTTATG-- -----TAGTT G--------- ---------- ----------

mel.64.3 AATTTATGCC CAATATAGTT G--------- ---------- ----------

mel.67.1 AATTTATGCC CAATATAGTT G--------- ---------- ----------

mel.55.2 AATTTATGCC CAATATAGTT G--------- ---------- ----------

**Closely-Related**

**Outgroup Species**

mau.5 AATTTATATC CATTAAA--- ---------- ---------- ----------

sec.38 AGTTTATGAC CATTAAAATT G--------- ---------- ----------

sim.33 TATTTATGTC CATTAAAATT G--------- ---------- ----------

**Outgroup Species**

yak.25 ---------C TATTAATATT ttttttacaa tttatgtgcc caacaaagat

**Distantly-related**

**Outgroup Species**

luc.41 AATTattttg taaaTATATA AACTAATTAA AAAGTTATta ataaatatcc

eug.20 ---------- ---------- ---------- ---------- ----------

fuy.9 ---------- ----TAAATA AAATAAATAC AAATTTATtc caatgcaaaa

**"Concestor" 291 ---------- ---------- ---------- ---------- ----------**

**"Concestor2" ---------- ---------- ---------- ---------- ----------**

mel.00.1 ---------- ---------- ---------- ---------- ----------

mel.01.58 ---------- ---------- ---------- ---------- ----------

mel.02.7 ---------- ---------- ---------- ---------- ----------

mel.04.1 ---------- ---------- ---------- ---------- ----------

mel.04.7 ---------- ---------- ---------- ---------- ----------

mel.05.13 ---------- ---------- ---------- ---------- ----------

mel.05.16 ---------- ---------- ---------- ---------- ----------

mel.07.20 ---------- ---------- ---------- ---------- ----------

mel.14.26 ---------- ---------- ---------- ---------- ----------

mel.17.66 ---------- ---------- ---------- ---------- ----------

mel.18Ug.7 ---------- ---------- ---------- ---------- ----------

mel.19.113 ---------- ---------- ---------- ---------- ----------

mel.23.74 ---------- ---------- ---------- ---------- ----------

mel.24.81 ---------- ---------- ---------- ---------- ----------

mel.26.32 ---------- ---------- ---------- ---------- ----------

mel.34.9 ---------- ---------- ---------- ---------- ----------

mel.39.37 ---------- ---------- ---------- ---------- ----------

mel.40Ug.12 ---------- ---------- ---------- ---------- ----------

mel.45.89 ---------- ---------- ---------- ---------- ----------

mel.CanS.41 ---------- ---------- ---------- ---------- ----------

mel.53.105 ---------- ---------- ---------- ---------- ----------

mel.54Ug.1 ---------- ---------- ---------- ---------- ----------

mel.59.3 ---------- ---------- ---------- ---------- ----------

mel.yw.50 ---------- ---------- ---------- ---------- ----------

mel.WI83.25 ---------- ---------- ---------- ---------- ----------

mel.51.100 ---------- ---------- ---------- ---------- ----------

mel.21.2 ---------- ---------- ---------- ---------- ----------

mel.29.3 ---------- ---------- ---------- ---------- ----------

mel.64.3 ---------- ---------- ---------- ---------- ----------

mel.67.1 ---------- ---------- ---------- ---------- ----------

mel.55.2 ---------- ---------- ---------- ---------- ----------

**Closely-Related**

**Outgroup Species**

mau.5 ---------- ---------- ---------- ---------- ----------

sec.38 ---------- ---------- ---------- ---------- ----------

sim.33 ---------- ---------- ---------- ---------- ----------

**Outgroup Species**

yak.25 ---------- ---------- ---------- ---------- ----------

**Distantly-related**

**Outgroup Species**

luc.41 cctaactttt aaattctgac caaataaaat ctttcttaaa tcatcaccC-

eug.20 ---------- ---------- ---------- ---------- ----------

fuy.9 atacatgttt ttttattcaa aaaaaggctt aactaaactt tctgaacgtg

**"Concestor" 292 ---------- ---------- ---------- ---------- ----------**

**"Concestor2" ---------- ---------- ---------- ---------- ----------**

mel.00.1 ---------- ---------- ---------- ---------- ----------

mel.01.58 ---------- ---------- ---------- ---------- ----------

mel.02.7 ---------- ---------- ---------- ---------- ----------

mel.04.1 ---------- ---------- ---------- ---------- ----------

mel.04.7 ---------- ---------- ---------- ---------- ----------

mel.05.13 ---------- ---------- ---------- ---------- ----------

mel.05.16 ---------- ---------- ---------- ---------- ----------

mel.07.20 ---------- ---------- ---------- ---------- ----------

mel.14.26 ---------- ---------- ---------- ---------- ----------

mel.17.66 ---------- ---------- ---------- ---------- ----------

mel.18Ug.7 ---------- ---------- ---------- ---------- ----------

mel.19.113 ---------- ---------- ---------- ---------- ----------

mel.23.74 ---------- ---------- ---------- ---------- ----------

mel.24.81 ---------- ---------- ---------- ---------- ----------

mel.26.32 ---------- ---------- ---------- ---------- ----------

mel.34.9 ---------- ---------- ---------- ---------- ----------

mel.39.37 ---------- ---------- ---------- ---------- ----------

mel.40Ug.12 ---------- ---------- ---------- ---------- ----------

mel.45.89 ---------- ---------- ---------- ---------- ----------

mel.CanS.41 ---------- ---------- ---------- ---------- ----------

mel.53.105 ---------- ---------- ---------- ---------- ----------

mel.54Ug.1 ---------- ---------- ---------- ---------- ----------

mel.59.3 ---------- ---------- ---------- ---------- ----------

mel.yw.50 ---------- ---------- ---------- ---------- ----------

mel.WI83.25 ---------- ---------- ---------- ---------- ----------

mel.51.100 ---------- ---------- ---------- ---------- ----------

mel.21.2 ---------- ---------- ---------- ---------- ----------

mel.29.3 ---------- ---------- ---------- ---------- ----------

mel.64.3 ---------- ---------- ---------- ---------- ----------

mel.67.1 ---------- ---------- ---------- ---------- ----------

mel.55.2 ---------- ---------- ---------- ---------- ----------

**Closely-Related**

**Outgroup Species**

mau.5 ---------- ---------- ---------- ---------- ----------

sec.38 ---------- ---------- ---------- ---------- ----------

sim.33 ---------- ---------- ---------- ---------- ----------

**Outgroup Species**

yak.25 ---------- ---------- ---------- ---------- ----------

**Distantly-related**

**Outgroup Species**

luc.41 ---------- ---------- ---------- ---------- ----------

eug.20 ---------- ---------- ---------- ----CAAATC CAGCAAAATC

fuy.9 aaacaatatt actaactagg gtatgtacta aataTAATTT GTATAAAATC

**"Concestor" 292 ---------- -------CAT TTCTCTGAGT GTGCAGTAAG TGCCCCAG-A**

**"Concestor2" ---------- -------CAT TTCTCTGAGT GTGCAGTAAG TGCCCCAG-A**

mel.00.1 ---------- -------CAT TTCTCTGAGT GTGCAGTAAG TGCCCCAG-A

mel.01.58 ---------- -------CAT TTCTCTGAGT GTGCAGTAAG TGCCCCAG-A

mel.02.7 ---------- -------CAT TTCTCTGAGT GTGCAGTAAG TGCCCCAG-A

mel.04.1 ---------- -------CAT TTCTCTGAGT GTGCAGTAAG TGCCCCAG-A

mel.04.7 ---------- -------CAT TTCTCTGAGT GTGCAGTAAG TGCCCCAG-A

mel.05.13 ---------- -------CAT TTCTCTGAGT GTGCAGTAAG TGCCCCAG-A

mel.05.16 ---------- -------CAT TTCTCTGAGT GTGCAGTAAG TGCCCCAG-A

mel.07.20 ---------- -------CAT TTCTCTGAGT GTGCAGTAAG TGCCCCAG-A

mel.14.26 ---------- -------CAT TTCTCTGAGT GTGCAGTAAG TGCCCCAG-A

mel.17.66 ---------- -------CAT TTCTCTGAGT GTGCAGTAAG TGCCCCAG-A

mel.18Ug.7 ---------- -------CAT TTCTCTGAGT GTGCAGTAAG TGCCCCAG-A

mel.19.113 ---------- -------CAT TTCTCTGAGT GTGCAGTAAG TGCCCCAG-A

mel.23.74 ---------- -------CAT TTCTCTGAGT GTGCAGTAAG TGCCCCAG-A

mel.24.81 ---------- -------CAT TTCTCTGAGT GTGCAGTAAG TGCCCCAG-A

mel.26.32 ---------- -------CAT TTCTCTGAGT GTGCAGTAAG TGCCCCAG-A

mel.34.9 ---------- -------CAT TTCTCTGAGT GTGCAGTAAG TGCCCCAG-A

mel.39.37 ---------- -------CAT TTCTCTGAGT GTGCAGTAAG TGCCCCAG-A

mel.40Ug.12 ---------- -------CAT TTCTCTGAGT GTGCAGTAAG TGCCCCAG-A

mel.45.89 ---------- -------CAT TTCTCTGAGT GTGCAGTAAG TGCCCCAG-A

mel.CanS.41 ---------- -------CAT TTCTCTGAGT GTGCAGTAAG TGCCCCAG-A

mel.53.105 ---------- -------CAT TTCTCTGAGT GTGCAGTAAG TGCCCCAG-A

mel.54Ug.1 ---------- -------CAT TTCTCTGAGT GTGCAGTAAG TGCCCCAG-A

mel.59.3 ---------- -------CAT TTCTCTGAGT GTGCAGTAAG TGCCCCAG-A

mel.yw.50 ---------- -------CAT TTCTCTGAGT GTGCAGTAAG TGCCCCAG-A

mel.WI83.25 ---------- -------CAT TTCTCTGAGT GTGCAGTAAG TGCCCCAG-A

mel.51.100 ---------- -------CAT TTCTCTGAGT GTGCAGTAAG TGCCCCAG-A

mel.21.2 ---------- -------CAT TTCTCTGAGT GTGCAGTAAG TGCCCCAG-A

mel.29.3 ---------- -------CAT TTCTCTGAGT GTGCAGTAAG TGCCCCAG-A

mel.64.3 ---------- -------CAT TTCTCTGAGT GTGCAGTAAG TGCCCCAG-A

mel.67.1 ---------- -------CAT TTCTCTGAGT GTGCAGTAAG TGCCCCAG-A

mel.55.2 ---------- -------CAT TTCTCTGAGT GTGCAGTAAG TGCCCCAG-A

**Closely-Related**

**Outgroup Species**

mau.5 ---------- ---------G TTCTCTGAGT GTGCAGTAAG TGCCCCAG-A

sec.38 ---------- -------CAT TTCTCTGAGT GTGCAGTAAG TGTCCCAG-A

sim.33 ---------- -------CAT TTCTCTGAGT GTGCAGTAAG TGCCCCAG-A

**Outgroup Species**

yak.25 ---------- ------AAAT TTCTCTAAGT GTGCAGTAAG ----------

**Distantly-related**

**Outgroup Species**

luc.41 ---------- --------AT TTCTCTCTGT GTACAGTAAG TGCTGGAGAA

eug.20 TGAGCCAACT CTATCAAAAC TTCTCTCAGT GTGCAGTAAG ----------

fuy.9 TGGCCAAAAG CAATGCAAAT TTTTTGTAGT GTACAGTAAG TGCCCAAG-A

**"Concestor" 323 ATGCGAATGC ATCTCGGGTT CATCGGCGGG TCGAGTTTGT TGCAACAACC**

**"Concestor2" ATGCGAATGC ATCTCGGGTT CATCGGCGGG TCGAGTTTGT TGCAACAACC**

mel.00.1 ATGCGAATGC ATCTCGGGTT CATCGGCGGG TCGAGTTTGT TGCAACAACC

mel.01.58 ATGCGAATGC ATCTCGGGTT CATCGGCGGG TCGAGTTTGT TGCAACAACC

mel.02.7 ATGCGAATGC ATCTCGGGTT CATCGGCGGG TCGAGTTTGT TGCAACAACC

mel.04.1 ATGCGAATGC ATCTCGGGTT CATCGGCGGG TCGAGTTTGT TGCAACAACC

mel.04.7 ATGCGAATGC ATCTCGGGTT CATCGGCGGG TCGAGTTTGT TGCAACAACC

mel.05.13 ATGCGAATGC ATCTCGGGTT CATCGGCGGG TCGAGTTTGT TGCAACAACC

mel.05.16 ATGCGAATGC ATCTCGGGTT CATCGGCGGG TCGAGTTTGT TGCAACAACC

mel.07.20 ATGCGAATGC ATCTCGGGTT CATCGGCGGG TCGAGTTTGT TGCAACAACC

mel.14.26 ATGCGAATGC ATCTCGGGTT CATCGGCGGG TCGAGTTTGT TGCAACAACC

mel.17.66 ATGCGAATGC ATCTCGGGTT CATCGGCGGG TCGAGTTTGT TGCAACAACC

mel.18Ug.7 ATGCGAATGC ATCTCGGGTT CATCGGCGGG TCGAGTTTGT TGCAACAACC

mel.19.113 ATGCGAATGC ATCTCGGGTT CATCGGCGGG TCGAGTTTGT TGCAACAACC

mel.23.74 ATGCGAATGC ATCTCGGGTT CATCGGCGGG TCGAGTTTGT TGCAACAACC

mel.24.81 ATGCGAATGC ATCTCGGGTT CATCGGCGGG TCGAGTTTGT TGCAACAACC

mel.26.32 ATGCGAATGC ATCTCGGGTT CATCGGCGGG TCGAGTTTGT TGCAACAACC

mel.34.9 ATGCGAATGC ATCTCGGGTT CATCGGCGGG TCGAGTTTGT TGCAACAACC

mel.39.37 ATGCGAATGC ATCTCGGGTT CATCGGCGGG TCGAGTTTGT TGCAACAACC

mel.40Ug.12 ATGCGAATGC ATCTCGGGTT CATCGGCGGG TCGAGTTTGT TGCAACAACC

mel.45.89 ATGCGAATGC ATCTCGGGTT CATCGGCGGG TCGAGTTTGT TGCAACAACC

mel.CanS.41 ATGCGAATGC ATCTCGGGTT CATCGGCGGG TCGAGTTTGT TGCAACAACC

mel.53.105 ATGCGAATGC ATCTCGGGTT CATCGGCGGG TCGAGTTTGT TGCAACAACC

mel.54Ug.1 ATGCGAATGC ATCTCGGGTT CATCGGCGGG TCGAGTTTGT TGCAACAACC

mel.59.3 ATGCGAATGC ATCTCGGGTT CATCGGCGGG TCGAGTTTGT TGCAACAACC

mel.yw.50 ATGCGAATGC ATCTCGGGTT CATCGGCGGG TCGAGTTTGT TGCAACAACC

mel.WI83.25 ATGCGAATGC ATCTCGGGTT CATCGGCGGG TCGAGTTTGT TGCAACAACC

mel.51.100 ATGCGAATGC ATCTCGGGTT CATCGGCGGG TCGAGTTTGT TGCAACAACC

mel.21.2 ATGCGAATGC ATCTCGGGTT CATCGGCGGG TCGAGTTTGT TGCAACAACC

mel.29.3 ATGCGAATGC ATCTCGGGTT CATCGGCGGG TCGAGTTTGT TGCAACAACC

mel.64.3 ATGCGAATGC ATCTCGGGTT CATCGGCGGG TCGAGTTTGT TGCAACAACC

mel.67.1 ATGCGAATGC ATCTCGGGTT CATCGGCGGG TCGAGTTTGT TGCAACAACC

mel.55.2 ATGCGAATGC ATCTCGGGTT CATCGGCGGG TCGAGTTTGT TGCAACAACC

**Closely-Related**

**Outgroup Species**

mau.5 ATGCGAATGC ATCTCGGGTT CATCGGCGGG TCGAGTTTGT TGCAACACCC

sec.38 ATGCGAATGC ATCTCGGGTT CATCGGTGGG TCGAGTTGGT TGCAACACCC

sim.33 ATGCGAATGC ATCTCGGGTT CATCGGCGGG TCGAGTTTGT TGCAACACCC

**Outgroup Species**

yak.25 -------TGC ATCTCGGGTT CATCGGGTTC ---AGTTTGT TGCAACACC-

**Distantly-related**

**Outgroup Species**

luc.41 ATGCGAATGC ATCTCGGGTT CATTGGCGGG TCGAGTTTGT TGCAACACC-

eug.20 -TGCGAATGC ATCTCGGGTT CATCGGCGGG TCGAGTTTGT TGCAACACC-

fuy.9 ATGCGAATGC ATCTCGGGTT CAACGGCGGG TCGAGTTTGT TGCATCACC-

**"Concestor" 373 GAAGAAC--- --------GA AGAAGTTGCA GCGTGCGTTC GGCATTAAAA**

**"Concestor2" GAAGAAC--- --------GA AGAAGTTGCA GCGTGCGTTC GGCATTAAAA**

mel.00.1 GAAGAAC--- --------GA AGAAGTTGCA GCGTGCGTTC GGCATTAAAA

mel.01.58 GAAGAAC--- --------GA AGAAGTTGCA GCGTGCGTTC GGCATTAAAA

mel.02.7 GAAGAAC--- --------GA AGAAGTTGCA GCGTGCGTTC GGCATTAAAA

mel.04.1 GAAGAAC--- --------GA AGAAGTTGCA GCGTGCGTTC GGCATTAAAA

mel.04.7 GAAGAAC--- --------GA AGAAGTTGCA GCGTGCGTTC GGCATTAAAA

mel.05.13 GAAGAAC--- --------GA AGAAGTTGCA GCGTGCGTTC GGCATTAAAA

mel.05.16 GAAGAAC--- --------GA AGAAGTTGCA GCGTGCGTTC GGCATTAAAA

mel.07.20 GAAGAAC--- --------GA AGAAGTTGCA GCGTGCGTTC GGCATTAAAA

mel.14.26 GAAGAAC--- --------GA AGAAGTTGCA GCGTGCGTTC GGCATTAAAA

mel.17.66 GAAGAAC--- --------GA AGAAGTTGCA GCGTGCGTTC GGCATTAAAA

mel.18Ug.7 GAAGAAC--- --------GA AGAAGTTGCA GCGTGCGTTC GGCATTAAAA

mel.19.113 GAAGAAC--- --------GA AGAAGTTGCA GCGTGCGTTC GGCATTAAAA

mel.23.74 GAAGAAC--- --------GA AGAAGTTGCA GCGTGCGTTC GGCATTAAAA

mel.24.81 GAAGAAC--- --------GA AGAAGTTGCA GCGTGCGTTC GGCATTAAAA

mel.26.32 GAAGAAC--- --------GA AGAAGTTGCA GCGTGCGTTC GGCATTAAAA

mel.34.9 GAAGAAC--- --------GA AGAAGTTGCA GCGTGCGTTC GGCATTAAAA

mel.39.37 GAAGAAC--- --------GA AGAAGTTGCA GCGTGCGTTC GGCATTAAAA

mel.40Ug.12 GAAGAAC--- --------GA AGAAGTTGCA GCGTGCGTTC GGCATTAAGA

mel.45.89 GAAGAAC--- --------GA AGAAGTTGCA GCGTGCGTTC GGCATTAAAA

mel.CanS.41 GAAGAAC--- --------GA AGAAGTTGCA GCGTGCGTTC GGCATTAAAA

mel.53.105 GAAGAAC--- --------GA AGAAGTTGCA GCGTGCGTTC GGCATTAAAA

mel.54Ug.1 GAAGAAC--- --------GA AGAAGTTGCA GCGTGCGTTC GGCATTAAAA

mel.59.3 GAAGAAC--- --------GA AGAAGTTGCA GCGTGCGTTC GGCATTAAAA

mel.yw.50 GAAGAAC--- --------GA AGAAGTTGCA GCGTGCGTTC GGCATTAAAA

mel.WI83.25 GAAGAAC--- --------GA AGAAGTTGCA GCGTGCGTTC GGCATTAAAA

mel.51.100 GAAGAAC--- --------GA AGAAGTTGCA GCGTGCGTTC GGCATTAAAA

mel.21.2 GAAGAAC--- --------GA AGAAGTTGCA GCGTGCGTTC GGCATTAAAA

mel.29.3 GAAGAAC--- --------GA AGAAGTTGCA GCGTGCGTTC GGCATTAAAA

mel.64.3 GAAGAAC--- --------GA AGAAGTTGCA GCGTGCGTTC GGCATTAAAA

mel.67.1 GAAGAAC--- --------GA AGAAGTTGCA GCGTGCGTTC GGCATTAAAA

mel.55.2 GAAGAAC--- --------GA AGAAGTTGCA GCGTGCGTTC GGCATTAAAA

**Closely-Related**

**Outgroup Species**

mau.5 GAAGAAC--- --------GA AGAAGTTGCA GCGTGCGTTC GGCATTAAAA

sec.38 GAAGAAC--- --------GA AGAAGTTGCA GCGTGCGTTC GGCATTAAAA

sim.33 GAAGAAC--- --------GA AGAAGTTGCA GCGTGCGTTC GGCATTAAAA

**Outgroup Species**

yak.25 ------C--- --------GA AGAAGTTGCA GCGTGCGTTC GGCATTAAAA

**Distantly-related**

**Outgroup Species**

luc.41 ------CGAA gaagagaaGA AGAACTTGCA GCGTGCGTCC GGCATTAAAA

eug.20 ------C--- --------Ga AGAACTTGCA GCGTGCGTCC GGCATTAAAA

fuy.9 ------C--- --------GA AGAACTTGCA GCGTGCGTCC GGCATTAAAA

**"Concestor" 412 TTGTGTTTAT GCGTGTTCGG TAATTTTATA AAAGTTAAAT TAGTTTTAAG**

**"Concestor2" TTGTGTTTAT GCGTGTTCGG TAATTTTATA AAAGTTAAAT TAGTTTTAAG**

mel.00.1 TTGTGTTTAT GCGTGTTCGG TAATTTTATA AAAGTTAAAT TAGTTTTAAG

mel.01.58 TTGTGTTTAT GCGTGTTCGG TAATTTTATA AAAGTTAAAT TAGTTTTAAG

mel.02.7 TTGTGTTTAT GCGTGTTCGG TAATTTTATA AAAGTTAAAT TAGTTTTAAG

mel.04.1 TTGTGTTTAT GCGTGTTCGG TAATTTTATA AAAGTTAAAT TAGTTTTAAG

mel.04.7 TTGTGTTTAT GCGTGTTCGG TAATTTTATA AAAGTTAAAT TAGTTTTAAG

mel.05.13 TTGTGTTTAT GCGTGTTCGG TAATTTTATA AAAGTTAAAT TAGTTTTAAG

mel.05.16 TTGTGTTTAT GCGTGTTCGG TAATTTTATA AAAGTTAAAT TAGTTTTAAG

mel.07.20 TTGTGTTTAT GCGTGTTCGG TAATTTTATA AAAGTTAAAT TAGTTTTAAG

mel.14.26 TTGTGTTTAT GCGTTTTCGG TAATTTTATA AAAGTTAAAT TAGTTTTAAG

mel.17.66 TTGTGTTTAT GCGTGTTCGG TAATTTTATA AAAGTTAAAT TAGTTTTAAG

mel.18Ug.7 TTGTGTTTAT GCGTGTTCGG TAATTTTATA AAAGTTAAAT TAGTTTTAAG

mel.19.113 TTGTGTTTAT GCGTGTTCGG TAATTTTATA AAAGTTAAAT TAGTTTTAAG

mel.23.74 TTGTGTTTAT GCGTGTTCGG TAATTTTATA AAAGTTAAAT TAGTTTTAAG

mel.24.81 TTGTGTTTAT GCGTGTTCGG TAATTTTATA AAAGTTAAAT TAGTTTTAAG

mel.26.32 TTGTGTTTAT GCGTGTTCGG TAATTTTATA AAAGTTAAAT TAGTTTTAAG

mel.34.9 TTGTGTTTAT GCGTGTTCGG TAATTTTATA AAAGTTAAAT TAGTTTTAAG

mel.39.37 TTGTGTTTAT GCGTGTTCGG TAATTTTATA AAAGTTAAAT TAGTTTTAAG

mel.40Ug.12 TTGTGTTTAT GCGTGTTCGG TAATTTTATA AAAGTTAAAT TAGTTTTAAG

mel.45.89 TTGTGTTTAT GCGTGTTCGG TAATTTTATA AAAGTTAAAT TAGTTTTAAG

mel.CanS.41 TTGTGTTTAT GCGTGTTCGG TAATTTTATA AAAGTTAAAT TAGTTTTAAG

mel.53.105 TTGTGTTTAT GCGTGTTCGG TAATTTTATA AAAGTTAAAT TAGTTTTAAG

mel.54Ug.1 TTGTGTTTAT GCGTGTTCGG TAATTTTATA AAAGTTAAAT TAGTTTTAAG

mel.59.3 TTGTGTTTAT GCGTGTTCGG TAATTTTATA AAAGTTAAAT TAGTTTTAAG

mel.yw.50 TTGTGTTTAT GCGTGTTCGG TAATTTTATA AAAGTTAAAT TAGTTTTAAG

mel.WI83.25 TTGTGTTTAT GCGTGTTCGG TAATTTTATA AAAGTTAAAT TAGTTTTAAG

mel.51.100 TTGTGTTTAT GCGTGTTCGG TAATTTTATA AAAGTTAAAT TAGTTTTAAG

mel.21.2 TTGTGTTTAT GCGTGTTCGG TAATTTTATA AAAGTTAAAT TAGTTTTAAG

mel.29.3 TTGTGTTTAT GCGTGTTCGG TAATTTTATA AAAGTTAAAT TAGTTTTAAG

mel.64.3 TTGTGTTTAT GCGTGTTCGG TAATTTTATA AAAGTTAAAT TAGTTTTAAG

mel.67.1 TTGTGTTTAT GCGTGTTCGG TAATTTTATA AAAGTTAAAT TAGTTTTAAG

mel.55.2 TTGTGTTTAT GCGTGTTCGG TAATTTTATA AAAGTTAAAT TAGTTTTAAG

**Closely-Related**

**Outgroup Species**

mau.5 TTGTGTTTAT GCGTGTTCGG TAATTTTATA AAAGTTAAAT TAGTTTTAAG

sec.38 TTGTGTTTAT GCGTGTTCGG TAATTTTATA AAAGTTAAAT TAGTTTTAAG

sim.33 TTGTGTTTAT GCGTGTTCGG TAATTTTATA AAAGTTAAAT TAGTTTTAAG

**Outgroup Species**

yak.25 TTGTGTTTAT GCGTGTTCGG TAATTTTATA AAAGTTAAAT TAGTTTTAAG

**Distantly-related**

**Outgroup Species**

luc.41 TTGTGTTTAT GCGTGTTTGG TAATTTTATA AAAGTTAAAT TAGTTTTAAG

eug.20 TTGTGTTTAT GCGTGTTTGG TAATTTTATA AAAGTTAAAT TAGTTTTAAG

fuy.9 TTGTGTTTAT GCGTGTTTGG TAATTTTATA AAAGTTAAAT TAGTTTTAAG

**18** **19**

"**Concestor" 462 ACCATAAATT CAGCTCACTC TCTCTCTCTC ------GCTC TTTCT--CTT**

"**Concestor2" ACCATAAATT CAGCTCACTC TCTCTCTC-- ------GCTC TTTCT--CTT**

mel.00.1 ACC**C**TAAATT CAGCTCACTC TCTCTCTCTC ------GCTC TTTCT--CTT

mel.01.58 ACCATAAATT CAGCTCACTC TCTCTCTCTC ------GCTC TTTCT--CTT

mel.02.7 ACCATAAATT CAGCTCACTC TCTCTCTCTC ------GCTC TTTCT--CTT

mel.04.1 ACC**C**TAAATT CAGCTCACTC TCTCTCTCTC ------GCTC TTTCT--CTT

mel.04.7 ACCCTAAATT CAGCTCACTC TCTCTCTCTC ------GCTC TTTCT--CTT

mel.05.13 ACCATAAATT CAGCTCACTC TCTCTCTCTC ------GCTC TTTCT--CTT

mel.05.16 ACCATAAATT CAGCTCACTC TCTCTCTCTC ------GCTC TTTCT--CTT

mel.07.20 ACCATAAATT CAGCTCACTC TCTCTCTCTC ------GCTC TTTCT--CTT

mel.14.26 ACCATAAATT CAGCTCACTC TCTCTCTCTC ------GCTC TTTCT--CTT

mel.17.66 ACCATAAATT CAGCTCACTC TCTCTCTCTC ------GCTC TTTCT--CTT

mel.18Ug.7 ACCATAAATT CAGCTCACTC TCTCTCTCTC --TTTCGCTC TTTCT--CTT

mel.19.113 ACCATAAATT CAGCTCACTC TCTCTCTCTC ------GCTC TTTCT--CTT

mel.23.74 ACCATAAATT CAGCTCACTC TCTCTCTCTC ------GCTC TTTCT--CTT

mel.24.81 ACCATAAATT CAGCTCACTC TCTCTCTCTC TCTTTCGCTC TTTCT--CTT

mel.26.32 ACCATAAATT CAGCTCACTC TCTCTCTCTC TCTTTCGCTC TTTCT--CTT

mel.34.9 ACCATAAATT CAGCTCACTC TCTCTCTC-- ------GCTC TTTCT--CTT

mel.39.37 ACCCTAAATT CAGCTCACTC TCTCTCTCTC ------GCTC TTTCT--CTT

mel.40Ug.12 ACCATAAATT CAGCTCACTC TCTCTCTC-- ------GCTC TTTCT--CTT

mel.45.89 ACCATAAATT CAGCTCACTC TCTCTCTCTC ------GCTC TTTCT--CTT

mel.CanS.41 ACCATAAATT CAGCTCACTC TCTCTCTCTC ------GCTC TTTCT--CTT

mel.53.105 ACCATAAATT CAGCTCACTC TCTCTCTCTC ------GCTC TTTCT--CTT

mel.54Ug.1 ACCATAAATT CAGCTCACTC TCTCTCTCTC --**TTTC**GCTC TTTCT--CTT

mel.59.3 ACCATAAATT CAGCTCACTC TCTCTCTC**--** ------GCTC TTTCT--CTT

mel.yw.50 ACCATAAATT CAGCTCACTC TCTCTCTCTC --TTTCGCTC TTTCT--CTT

mel.WI83.25 ACC**C**TAAATT CAGCTCACTC TCTCTCTCTC ------GCTC TTTCT--CTT

mel.51.100 ACCATAAATT CAGCTCACTC TCTCTCTCTC -–TTTCGCTC TTTCT--CTT

mel.21.2 ACCATAAATT CAGCTCACTC TCTCTCTCTC --TCGCGCTC TTTCT--CTT

mel.29.3 ACCATAAATT CAGCTCACTC TCTCTCTC**--** ------GCTC TTTCT--CTT

mel.64.3 ACCATAAATT CAGCTCACTC TCTCTCTCTC ------GCTC TTTCT--CTT

mel.67.1 ACCATAAATT CAGCTCACTC TCTCTCTCTC ------GCTC TTTCT--CTT

mel.55.2 ACC**C**TAAATT CAGCTCACTC TCTCTCTCTC ------GCTC TTTCT--CTT

**Closely-Related**

**Outgroup Species**

mau.5 ACCATAAATT CAGCTCACTC CCTCTCTC-- ------GCTC TTTCT--CTT

sec.38 ACCATAAATT CAGCTCACTT CCTCTCTC-- ------GCTC TTTCT--CTT

sim.33 ACCATAAATT CAGCTCACTC CCTCTCTC-- ------GCTC TTTCT--CTT

**Outgroup Species**

yak.25 ACCATAAATT CAGCTCACTC TCTCCCTCGG ------CCTC TCTGTCTCTT

**Distantly-related**

**Outgroup Species**

luc.41 ACCATAAATT CAGCTCTCGC A--------C TCTCTGCCAT ----------

eug.20 ACCATAAATT CAGCTCACTC TCT------C TCTCTCtcgc ccgGTCT--C

fuy.9 ACCATAAATT CAGCGCACTC TCTggcata- ---------- ---GTCT--C

**Abd-B 1 Abd-B 2**

**"Concestor" 504 TGCCATTTTA ACTTTTATTA CTCTTAATAT AAAAAAGCTG GCT----AGA**

**"Concestor2" TGCCATTTTA ACTTTTATTA CTCTTAATAT AAAAAAGCTG GCT----AGA**

mel.00.1 TGCCATTTTA ACTTTTATTA CTCTTAATAT AAAAAAGCTG GCT----AGA

mel.01.58 TGCCATTTTA ACTTTTATTA CTCTTAATAT AAAAAAGCTG GCT----AGA

mel.02.7 TGCCATTTTA ACTTTTATTA CTCTTAATAT AAAAAAGCTG GCT----AGA

mel.04.1 TGCCATTTTA ACTTTTATTA CTCTTAATAT AAAAAAGCTG GCT----AGA

mel.04.7 TGCCATTTTA ACTTTTATTA CTCTTAATAT AAAAAAGCTG GCT----AGA

mel.05.13 TGCCATTTTA ACTTTTATTA CTCTTAATAT AAAAAAGCTG GCT----AGA

mel.05.16 TGCCATTTTA ACTTTTATTA CTCTTAATAT AAAAAAGCTG GCT----AGA

mel.07.20 TGCCATTTTA ACTTTTATTA CTCTTAATAT AAAAAAGCTG GCT----AGA

mel.14.26 TGCCATTTTA ACTTTTATTA CTCTTAATAT AAAAAAGCTG GCT----AGA

mel.17.66 TGCCATTTTA ACTTTTATTA CTCTTAATAT AAAAAAGCTG GCT----AGA

mel.18Ug.7 TGCCATTTTA ACTTTTATTA CTCTTAATAT AAAAAAGCTG GCT----AGA

mel.19.113 TGCCATTTTA ACTTTTATTA CTCTTAATAT AAAAAAGCTG GCT----AGA

mel.23.74 TGCCATTTTA ACTTTTATTA CTCTTAATAT AAAAAAGCTG GCT----AGA

mel.24.81 TGCCATTTTA ACTTTTATTA CTCTTAATAT AAAAAAGCTG GCT----AGA

mel.26.32 TGCCATTTTA ACTTTTATTA CTCTTAATAT AAAAAAGCTG GCT----AGA

mel.34.9 TGCCATTTTA ACTTTTATTA CTCTTAATAT AAAAAAGCTG GCT----AGA

mel.39.37 TGCCATTTTA ACTTTTATTA CTCTTAATAT AAAAAAGCTG GCT----AGA

mel.40Ug.12 TGCCATTTTA ACTTTTATTA CTCTTAATAT AAAAAAGCTG GCT----AGA

mel.45.89 TGCCATTTTA ACTTTTATTA CTCTTAATAT AAAAAAGCTG GCT----AGA

mel.CanS.41 TGCCATTTTA ACTTTTATTA CTCTTAATAT AAAAAAGCTG GCT----AGA

mel.53.105 TGCCATTTTA ACTTTTATTA CTCTTAATAT AAAAAAGCTG GCT----AGA

mel.54Ug.1 TGCCATTTTA ACTTTTATTA CTCTTAATAT AAAAAAGCTG GCT----AGA

mel.59.3 TGCCATTTTA ACTTTTATTA CTCTTAATAT AAAAAAGCTG GCT----AGA

mel.yw.50 TGCCATTTTA ACTTTTATTA CTCTTAATAT AAAAAAGCTG GCT----AGA

mel.WI83.25 TGCCATTTTA ACTTTTATTA CTCTTAATAT AAAAAAGCTG GCT----AGA

mel.51.100 TGCCATTTTA ACTTTTATTA CTCTTAATAT AAAAAAGCTG GCT----AGA

mel.21.2 TGCCATTTTA ACTTTTATTA CTCTTAATAT AAAAAAGCTG GCT----AGA

mel.29.3 TGCCATTTTA ACTTTTATTA CTCTTAATAT AAAAAAGCTG GCT----AGA

mel.64.3 TGCCATTTTA ACTTTTATTA CTCTTAATAT AAAAAAGCTG GCT----AGA

mel.67.1 TGCCATTTTA ACTTTTATTA CTCTTAATAT AAAAAAGCTG GCT----AGA

mel.55.2 TGCCATTTTA ACTTTTATTA CTCTTAATAT AAAAAAGCTG GCT----AGA

**Closely-Related**

**Outgroup Species**

mau.5 TGCCAGTTTA ACTTTTATTA CTCTTAATAT AAAAAAGCTG GCT----AGA

sec.38 TGCCATTTTA ACTTTTATTA CTCTTAATAT AAAAAAGCTG GCT----AGA

sim.33 TGCCATTTTA ACTTTTATTA CTCTTAATAT AAAAAAGCTG GCT----AGA

**Outgroup Species**

yak.25 TGCCATTTTA ACTTTTATTA CTCTTAATAT AAAAAAGCTG GCTGGCTAGA

**Distantly-related**

**Outgroup Species**

luc.41 ------TTTA ACTTTTATTA CTTTTAATAT AAAAAAACTG GCT----AGA

eug.20 TGCCATTTTA ACTTTTATTA CTTTTAATAT AAAAAAGCTG GCT----AGA

fuy.9 TGCCATTTTA ACTTTTATTA CTTTTAATAT AAAAAAGCTG Ga----TAGA

**C** **Abd-B 3 Abd-B 4**

**"Concestor" 550 AGC------- --GGGCCAGC TGTAAAAATG CACGCGGTCA TAAAAAGTTG**

**"Concestor2" AGC------- --GGGCCAGC TGTAAAAATG CACGCGGTCA TAAAAAGTTG**

mel.00.1 TGC------- --GGGCCAGC TGTAAAAATG CACGCGGTCA TAAAAAGTTG

mel.01.58 TGC------- --GGGCCAGC TGTAAAAATG CACGCGGTCA TAAAAAGTTG

mel.02.7 TGC------- --GGGCCAGC TGTAAAAATG CACGCGGTCA TAAAAAGTTG

mel.04.1 TGC------- --GGGCCAGC TGTAAAAATG CACGCGGTCA TAAAAAGTTG

mel.04.7 **T**GC------- --GGGCCAGC TGTAAAAATG CACGCGGTCA TAAAAAGTTG

mel.05.13 TGC------- --GGGCCAGC TGTAAAAATG CACGCGGTCA TAAAAAGTTG

mel.05.16 TGC------- --GGGCCAGC TGTAAAAATG CACGCGGTCA TAAAAAGTTG

mel.07.20 TGC------- --GGGCCAGC TGTAAAAATG CACGCGGTCA TAAAAAGTTG

mel.14.26 TGC------- --GGGCCAGC TGTAAAAATG CACGCGGTCA TAAAAAGTTG

mel.17.66 TGC------- --GGGCCAGC TGTAAAAATG CACGCGGTCA TAAAAAGTTG

mel.18Ug.7 TGC------- --GGGCCAGC TGTAAAAATG CACGCGGTCA TAAAAAGTTG

mel.19.113 **T**GC------- --GGGCCAGC TGTAAAAATG CACGCGGTCA TAAAAAGTTG

mel.23.74 TGC------- --GGGCCAGC TGTAAAAATG CACGCGGTCA TAAAAAGTTG

mel.24.81 TGC------- --GGGCCAGC TGTAAAAATG CACGCGGTCA TAAAAAGTTG

mel.26.32 TGC------- --GGGCCAGC TGTAAAAATG CACGCGGTCA TAAAAAGTTG

mel.34.9 TGC------- --GGGCCAGC TGTAAAAATG CACGCGGTCA TAAAAAGTTG

mel.39.37 TGC------- --GGGCCAGC TGTAAAAATG CACGCGGTCA TAAAAAGTTG

mel.40Ug.12 AGC------- --GGGCCAGC TGTAAAAATG CACGCGGTCA TAAAAAGTTG

mel.45.89 AGC------- --GGGCCAGC TGTAAAAATG CACGCGGTCA TAAAAAGTTG

mel.CanS.41 TGC------- --GGGCCAGC TGTAAAAATG CACGCGGTCA TAAAAAGTTG

mel.53.105 TGC------- --GGGCCAGC TGTAAAAATG CACGCGGTCA TAAAAAGTTG

mel.54Ug.1 **T**GC------- --GGGCCAGC TGTAAAAATG CACGCGGTCA TAAAAAGTTG

mel.59.3 **T**GC------- --GGGCCAGC TGTAAAAATG CACGCGGTCA TAAAAAGTTG

mel.yw.50 TGC------- --GGGCCAGC TGTAAAAATG CACGCGGTCA TAAAAAGTTG

mel.WI83.25 TGC------- --GGGCCAGC TGTAAAAATG CACGCGGTCA TAAAAAGTTG

mel.51.100 TGC------- --GGGCCAGC TGTAAAAATG CACGCGGTCA TAAAAAGTTG

mel.21.2 TGC------- --GGGCCAGC TGTAAAAATG CACGCGGTCA TAAAAAGTTG

mel.29.3 TGC------- --GGGCCAGC TGTAAAAATG CACGCGGTCA TAAAAAGTTG

mel.64.3 AGC------- --GGGCCAGC TGTAAAAATG CACGCGGTCA TAAAAAGTTG

mel.67.1 TGC------- --GGGCCAGC TGTAAAAATG CACGCGGTCA TAAAAAGTTG

mel.55.2 TGC------- --GGGCCAGC TGTAAAAATG CACGCGGTCA TAAAAAGTTG

**Closely-Related**

**Outgroup Species C**

mau.5 **A**GC------- --GGGCCAGC TGTAAAAATG CACGCGGTCA TAAAAAGTTG

sec.38 AGC------- --GGGCCAGC TGTAAAAATG CTCGCGGTCA TAAAAAGTTG

sim.33 AGC------- --GGGCCAGC TGTAAAAATG CACGCGGTCA TAAAAAGTTG

**Outgroup Species**

yak.25 AGC------- --GGGCCAGC TGTAAAAATG CATGCGCTCA TAAAAAGTTG

**Distantly-related**

**Outgroup Species**

luc.41 AGCGGCGGCG GTGGGCCAGC TGTAAAAATG CACGCGGTCA TAAAAAATTG

eug.20 AGCGGG---- -----CCAGC TGTAAAAATG CACGCGGTCA TAAAAAGTTG

fuy.9 AGC------- --GGGCCAGC TGTAAAAATG CACGCGGTCA TAAAAAGTTG

**"Concestor" 591 CAGGAGGCAT GTTGCC---- ---------- -AGTTGCCTG CAACCGGCAA**

**"Concestor2" CAGGAGGCAT GTTGCC---- ---------- -AGTTGCCTG CAACCGGCAA**

mel.00.1 CAGGAGGCAT GTTGCC---- ---------- -AGTTGCCTG CAACCGGCAA

mel.01.58 CAGGAGGCAT GTTGCC---- ---------- -AGTTGCCTG CAACCGGCAA

mel.02.7 CAGGAGGCAT GTTGCC---- ---------- -AGTTGCCTG CAACCGGCAA

mel.04.1 CAGGAGGCAT GTTGCC---- ---------- -AGTTGCCTG CAACCGGCAA

mel.04.7 CAGGAGGCAT GTTGCC---- ---------- -AGTTGCCTG CAACCGGCAA

mel.05.13 CAGGAGGCAT GTTGCC---- ---------- -AGTTGCCTG CAACCGGCAA

mel.05.16 CAGGAGGCAT GTTGCC---- ---------- -AGTTGCCTG CAACCGGCAA

mel.07.20 CAGGAGGCAT GTTGCC---- ---------- -AGTTGCCTG CAACCGGCAA

mel.14.26 CAGGAGGCAT GTTGCC---- ---------- -AGTTGCCTG CAACCGGCAA

mel.17.66 CAGGAGGCAT GTTGCC---- ---------- -AGTTGCCTG CAACCGGCAA

mel.18Ug.7 CAGGAGGCAT GTTGCC---- ---------- -AGTTGCCTG CAACCGGCAA

mel.19.113 CAGGAGGCAT GTTGCC---- ---------- -AGTTGCCTG CAACCGGCAA

mel.23.74 CAGGAGGCAT GTTGCC---- ---------- -AGTTGCCTG CAACCGGCAA

mel.24.81 CAGGAGGCAT GTTGCC---- ---------- -AGTTGCCTG CAACCGGCAA

mel.26.32 CAGGAGGCAT GTTGCC---- ---------- -AGTTGCCTG CAACCGGCAA

mel.34.9 CAGGAGGCAT GTTGCC---- ---------- -AGTTGCCTG CAACCGGCAA

mel.39.37 CAGGAGGCAT GTTGCC---- ---------- -AGTTGCCTG CAACCGGCAA

mel.40Ug.12 CAGGAGGCAT GTTGCC---- ---------- -AGTTGCCTG CAACCGGCAA

mel.45.89 CAGGAGGCAT GTTGCC---- ---------- -AGTTGCCTG CAACCGGCAA

mel.CanS.41 CAGGAGGCAT GTTGCC---- ---------- -AGTTGCCTG CAACCGGCAA

mel.53.105 CAGGAGGCAT GTTGCC---- ---------- -AGTTGCCTG CAACCGGCAA

mel.54Ug.1 CAGGAGGCAT GTTGCC---- ---------- -AGTTGCCTG CAACCGGCAA

mel.59.3 CAGGAGGCAT GTTGCC---- ---------- -AGTTGCCTG CAACCGGCAA

mel.yw.50 CAGGAGGCAT GTTGCC---- ---------- -AGTTGCCTG CAACCGGCAA

mel.WI83.25 CAGGAGGCAT GTTGCC---- ---------- -AGTTGCCTG CAACCGGCAA

mel.51.100 CAGGAGGCAT GTTGCC---- ---------- -AGTTGCCTG CAACCGGCAA

mel.21.2 CAGGAGGCAT GTTGCC---- ---------- -AGTTGCCTG CAACCGGCAA

mel.29.3 CAGGAGGCAT GTTGCC---- ---------- -AGTTGCCTG CAACCGGCAA

mel.64.3 CAGGAGGCAT GTTGCC---- ---------- -AGTTGCCTG CAACCGGCAA

mel.67.1 CAGGAGGCAT GTTGCC---- ---------- -AGTTGCCTG CAACCGGCAA

mel.55.2 CAGGAGGCAT GTTGCC---- ---------- -AGTTGCCTG CAACCGGCAA

**Closely-Related**

**Outgroup Species**

mau.5 CAGGAGGCAT GTTGCC---- ---------- -AGTTGCCTG CAACCGGCAA

sec.38 CAGGAGGCAT GTTGCC---- ---------- -AGTTGCCTG CAACCGGCAA

sim.33 CAGGAGGCAT GTTGCC---- ---------- -AGTTGCCTG CAACCGGCAA

**Outgroup Species**

yak.25 CAGGAGGCAT GTTGCC---- ---------- -AGTTGCCAG TTGCC-----

**Distantly-related**

**Outgroup Species**

luc.41 CAGGAGGCAT GTTGCCggt- ---------- ---------- TGCCAAGTTC

eug.20 CAGGAGGCAT GTTGCCGGTT GCcagttgcc ggttgctggc TGCCAAGTTC

fuy.9 CAGGAGGCAT GTTGCTGGTA GCcaagttgc cAGTTGCCGG TTGCC-----

**Abd-B 5**

**"Concestor" 626 CATTCG---- -------C-- --AG------ -AACAGCAGC AACATCGTAA**

**"Concestor2" CATTCG---- -------C-- --AG------ -AACAGCAGC AACATCGTAA**

mel.00.1 CATTCG---- -------C-- --AG------ -AACAGCAGC AACATCGTAA

mel.01.58 CATTCG---- -------C-- --AG------ -AACAGCAGC AACATCGTAA

mel.02.7 CATTCG---- -------C-- --AG------ -AACAGCAGC AACATCGTAA

mel.04.1 CATTCG---- -------C-- --AG------ -AACAGCAGC AACATCGTAA

mel.04.7 CATTCG---- -------C-- --AG------ -AACAGCAGC AACATCGTAA

mel.05.13 CATTCG---- -------C-- --AG------ -AACAGCAGC AACATCGTAA

mel.05.16 CATTCG---- -------C-- --AG------ -AACAGCAGC AACATCGTAA

mel.07.20 CATTCG---- -------C-- --AG------ -AACAGCAGC AACATCGTAA

mel.14.26 CATTCG---- -------C-- --AG------ -AACAGCAGC AACATCGTAA

mel.17.66 CATTCG---- -------C-- --AG------ -AACAGCAGC AACATCGTAA

mel.18Ug.7 CATTCG---- -------C-- --AG------ -AACAGCAGC AACATCGTAA

mel.19.113 CATTCG---- -------C-- --AG------ -AACAGCAGC AACATCGTAA

mel.23.74 CATTCG---- -------C-- --AG------ -AACAGCAGC AACATCGTAA

mel.24.81 CATTCG---- -------C-- --AG------ -AACAGCAGC AACATCGTAA

mel.26.32 CATTCG---- -------C-- --AG------ -AACAGCAGC AACATCGTAA

mel.34.9 CATTCG---- -------C-- --AG------ -AACAGCAGC AACATCGTAA

mel.39.37 CATTCG---- -------C-- --AG------ -AACAGCAGC AACATCGTAA

mel.40Ug.12 CATTCG---- -------C-- --AG------ -AACAGCAGC AACATCGTAA

mel.45.89 CATTCG---- -------C-- --AG------ -AACAGCAGC AACATCGTAA

mel.CanS.41 CATTCG---- -------C-- --AG------ -AACAGCAGC AACATCGTAA

mel.53.105 CATTCG---- -------C-- --AG------ -AACAGCAGC AACATCGTAA

mel.54Ug.1 CATTCG---- -------C-- --AG------ -AACAGCAGC AACATCGTAA

mel.59.3 CATTCG---- -------C-- --AG------ -AACAGCAGC AACATCGTAA

mel.yw.50 CATTCG---- -------C-- --AG------ -AACAGCAGC AACATCGTAA

mel.WI83.25 CATTCG---- -------C-- --AG------ -AACAGCAGC AACATCGTAA

mel.51.100 CATTCG---- -------C-- --AG------ -AACAGCAGC AACATCGTAA

mel.21.2 CATTCG---- -------C-- --AG------ -AACAGCAGC AACATCGTAA

mel.29.3 CATTCG---- -------C-- --AG------ -AACAGCAGC AACATCGTAA

mel.64.3 CATTCG---- -------C-- --AG------ -AACAGCAGC AACATCGTAA

mel.67.1 CATTCG---- -------C-- --AG------ -AACAGCAGC AACATCGTAA

mel.55.2 CATTCG---- -------C-- --AG------ -AACAGCAGC AACATCGTAA

**Closely-Related**

**Outgroup Species**

mau.5 CATCCG---- -------C-- --AC------ -AACAGCAGC AACATCGTAA

sec.38 CATCCG---- -------C-- --AC------ -AACAGCAGC AACATCGTAA

sim.33 CATCCG---- -------C-- --AC------ -AACAGCAGC AACATCGTAA

**Outgroup Species**

yak.25 ---------- --TGCAAC-- --AG------ -AACAGCAGC AACATCGTAA

**Distantly-related**

**Outgroup Species**

luc.41 CCAAGTTGCC CCTGCAACAT CCAC------ -AGCAGCAGC AACATCGTAA

eug.20 CCAAGTTGCC TGTGCAAC-- --ATCCACAG AAACAGCAGC AACATCGTAA

fuy.9 ---------- --TGCAAC-- --ATCCACTG AAACGGCAGC AACATCGTAA

**D E Dsx1 Site**

**"Concestor" 654 AATAACTTCT TGCTCTGCGG TCTGAGTTTG GCCGCAACAA TGTTGCTGCA**

**"Concestor2" AATAACTTCT TGCTCTGCGG TCTGAGTTTG GCCGCAACAA TGTTGCTGCA**

mel.00.1 AATAACTTCT TGCTCTGCGG TCTGAGTTTG GCCGCAACAA TGTTGCTGCA

mel.01.58 AATAACTTCT TGCTCTGCGG TCTGAGTTTG GCCGCAACAA TGTTGCTGCA

mel.02.7 AATAACTTCT TCCTCTGCGG TCTGAGTTTG GCCGCAACAA TGTTGCTGCA

mel.04.1 AATAACTTCT TGCTCTGCGG TCTGAGTTTG GCCGCAACAA TGTTGCTGCA

mel.04.7 AATAACTTCT TGCTCTGCGG TCTGAGTTTG GCCGCAACAA TGTTGCTGCA

mel.05.13 AATAACTTCT TGCTCTGCGG TCTGAGTTTG GCCGCAACAA TGTTGCTGCA

mel.05.16 AATAACTTCT TGCTCTGCGG TCTGAGTTTG GCCGCAACAA TGTTGCTGCA

mel.07.20 AATAACTTCT TGCTCTGCGG TCTGAGTTTG GCCGCAACAA TGTTGCTGCA

mel.14.26 AATAACTTCT TGCTCTGCGG TCTGAGTTTG GCCGCAACAA TGTTGCTGCA

mel.17.66 AATAACTTCT TGCTCTGCGG TCTGAGTTTG GCCGCAACAA TGTTGCTGCA

mel.18Ug.7 AATAACTTCT TCCTCTGCGG TCTGAGTTTG GCCGCAACAA TGTTGCTGCA

mel.19.113 AATAACTTCT TGCTCTGCGG TCTGAGTTTG GCCGCAACAA TGTTGCTGCA

mel.23.74 AATAACTTCT TGCTCTGCGG TCTGAGTTTG GCCGCAACAA TGTTGCTGCA

mel.24.81 AATAACTTCT TGCTCTGCGG TCTGAGTTTG GCCGCAACAA TGTTGCTGCA

mel.26.32 AATAACTTCT TGCTCTGCGG TCTGAGTTTG GCCGCAACAA TGTTGCTGCA

mel.34.9 AATAACTTCT TGCTCTGCGG TCTGAGTTTG GCCGCAACAA TGTTGCTGCA

mel.39.37 AATAACTTCT TGCTCTGCGG TCTGAGTTTG GCCGCAACAA TGTTGCTGCA

mel.40Ug.12 AATAACTTCT TCCTCTGCGG TCTGAGTTTG GCCGCAACAA TGTTGCTGCA

mel.45.89 AATAACTTCT TGCTCTGCGG TCTGAGTTTG GCCGCAACAA TGTTGCTGCA

mel.CanS.41 AATAACTTCT TGCTCTGCGG TCTGAGTTTG GCCGCAACAA TGTTGCTGCA

mel.53.105 AATAACTTCT TGCTCTGCGG TCTGAGTTTG GCCGCAACAA TGTTGCTGCA

mel.54Ug.1 AATAACTTCT T**C**CTCTGCGG TCTGAGTTTG GCCGCAACAA TGTTGCTGCA

mel.59.3 AATAACTTCT TCCTCTGCGG TCTGA**----- ----**CAACAA TGTTGCTGCA

mel.yw.50 AATAACTTCT TCCTCTGCGG TCTGAGTTTG GCCGCAACAA TGTTGCTGCA

mel.WI83.25 AATAACTTCT TGCTCTGCGG TCTGAGTTTG GCCGCAACAA TGTTGCTGCA

mel.51.100 AATAACTTCT TCCTCTGCGG TCTGA----- ----CAACAA TGTTGCTGCA

mel.21.2 AATAACTTCT TGCTCTGCGG TCTGAGTTTG GCCGCAACAA TGTTGCTGCA

mel.29.3 AATAACTTCT TGCTCTGCGG TCTGAGTTTG GCCGCAACAA TGTTGCTGCA

mel.64.3 AATAACTTCT TGCTCTGCGG TCTGAGTTTG GCCGCAACAA TGTTGCTGCA

mel.67.1 AATAACTTCT TGCTCTGCGG TCTGAGTTTG GCCGCAACAA TGTTGCTGCA

mel.55.2 AATAACTTCT TGCTCTGCGG TCTGAGTTTG GCCGCAACAA TGTTGCTGCA

**Closely-Related**

**Outgroup Species D E**

mau.5 AATAACTTCT TGCTCTGCGG TCTGCATTTG GCCGCAACAA TGTTGCTGCA

sec.38 AATAACTTCT TGCTCTGGGG TCTGCATTTG GCCGCAACAA TGTTGCTGCA

sim.33 AATAACTTCT TGCTCTGCGG TCTGCATTTG GCCGCAACAA TGTTGCTGCA

**Outgroup Species**

yak.25 AATAACTTCT TGCTCTGCGG TCTCCGTTTG GCCGCAACAA TGTTGCCGCA

**Distantly-related**

**Outgroup Species**

luc.41 AATAATTTCT TTCTCTGCGG TCTCCGTTTG GCCGCAACAA TGTTGCTGCA

eug.20 AATAATTTCT TGCTCTGCGG TCTCCGCTCG GTTGCAACAA TGTTGCGGCA

fuy.9 AATAATTTCT TGCTCTGCGG TCTCCATTTG GCCGCAACAA TGTTGCTGCA

**Abd-B 6**

**"Concestor" 704 TTTATTCGTA TTATTATTAC ATTTTAATGA ATAATTCTAA TTATATGCAA**

**"Concestor2" TTTATTCGTA TTATTATTAC ATTTTAATGA ATAATTCTAA TTATATGCAA**

mel.00.1 TTTATTCGTA TTATTATTAC ATTTTAATGA ATAATTCTAA TTATATGCAA

mel.01.58 TTTATTCGTA TTATTATTAC ATTTTAATGA ATAATTCTAA TTATATGCAA

mel.02.7 TTTATTCGTA TTATTATTAC ATTTTAATGA ATAATTCTAA TTATATGCAA

mel.04.1 TTTATTCGTA TTATTATTAC ATTTTAATGA ATAATTCTAA TTATATGCAA

mel.04.7 TTTATTCGTA TTATTATTAC ATTTTAATGA ATAATTCTAA TTATATGCAA

mel.05.13 TTTATTCGTA TTATTATTAC ATTTTAATGA ATAATTCTAA TTATATGCAA

mel.05.16 TTTATTCGTA TTATTATTAC ATTTTAATGA ATAATTCTAA TTATATGCAA

mel.07.20 TTTATTCGTA TTATTATTAC ATTTTAATGA ATAATTCTAA TTATATGCAA

mel.14.26 TTTATTCGTA TTATTATTAC ATTTTAATGA ATAATTCTAA TTATATGCAA

mel.17.66 TTTATTCGTA TTATTATTAC ATTTTAATGA ATAATTCTAA TTATATGCAA

mel.18Ug.7 TTTATTCGTA TTATTATTAC ATTTTAATGA ATAATTCTAA TTATATGCAA

mel.19.113 TTTATTCGTA TTATTATTAC ATTTTAATGA ATAATTCTAA TTATATGCAA

mel.23.74 TTTATTCGTA TTATTATTAC ATTTTAATGA ATAATTCTAA TTATATGCAA

mel.24.81 TTTATTCGTA TTATTATTAC ATTTTAATGA ATAATTCTAA TTATATGCAA

mel.26.32 TTTATTCGTA TTATTATTAC ATTTTAATGA ATAATTCTAA TTATATGCAA

mel.34.9 TTTATTCGTA TTATTATTAC ATTTTAATGA ATAATTCTAA TTATATGCAA

mel.39.37 TTTATTCGTA TTATTATTAC ATTTTAATGA ATAATTCTAA TTATATGCAA

mel.40Ug.12 TTTATTCGTA TTATTATTAC ATTTTAATGA ATAATTCTAA TTATATGCAA

mel.45.89 TTTATTCGTA TTATTATTAC ATTTTAATGA ATAATTCTAA TTATATGCAA

mel.CanS.41 TTTATTCGTA TTATTATTAC ATTTTAATGA ATAATTCTAA TTATATGCAA

mel.53.105 TTTATTCGTA TTATTATTAC ATTTTAATGA ATAATTCTAA TTATATGCAA

mel.54Ug.1 TTTATTCGTA TTATTATTAC ATTTTAATGA ATAATTCTAA TTATATGCAA

mel.59.3 TTTATTCGTA TTATTATTAC ATTTTAATGA ATAATTCTAA TTATATGCAA

mel.yw.50 TTTATTCGTA TTATTATTAC ATTTTAATGA ATAATTCTAA TTATATGCAA

mel.WI83.25 TTTATTCGTA TTATTATTAC ATTTTAATGA ATAATTCTAA TTATATGCAA

mel.51.100 TTTATTCGTA TTATTATTAC ATTTTAATGA ATAATTCTAA TTATATGCAA

mel.21.2 TTTATTCGTA TTATTATTAC ATTTTAATGA ATAATTCTAA TTATATGCAA

mel.29.3 TTTATTCGTA TTATTATTAC ATTTTAATGA ATAATTCTAA TTATATGCAA

mel.64.3 TTTATTCGTA TTATTATTAC ATTTTAATGA ATAATTCTAA TTATATGCAA

mel.67.1 TTTATTCGTA TTATTATTAC ATTTTAATGA ATAATTCTAA TTATATGCAA

mel.55.2 TTTATTCGTA TTATTATTAC ATTTTAATGA ATAATTCTAA TTATATGCAA

**Closely-Related**

**Outgroup Species**

mau.5 TTTATTCGTA TTATTATTAC AATTTAATGA ATAATTCTAA TTATATGCGA

sec.38 TTTATTCGTA TTATTATTAC AATTTAATGA ATAATTCTAA TTATATGCGA

sim.33 TTTATTCGTA TTATTATTAC AATTTAATGA ATAATTCTAA TTATATGCGA

**Outgroup Species**

yak.25 TTTATTCGTA TTATTATTAC ATTTTAATGA ATAATTCTAA TTATATGCAA

luc.41 TTTATTCGTA TTATTATTAC ATTTTAATGA TTAATTCTAA TTATATGCGA

eug.20 TTTATTCGTA TTATTATTAC ATTTTAATGA TTAATTCTAA TTATATGCGA

fuy.9 TTTATTCGTA TTATTATTAC ATTTTAATGA TTAATTCTAA TTATATGCGA

**"Concestor" 754 CTTGAATAAG CCCGC----- ---------- --CGA----- ----------**

**"Concestor2" CTTGAATAAG CCCGC----- ---------- --CGA----- ----------**

mel.00.1 CTTGAATAAG CCCGC----- ---------- --CGA----- ----------

mel.01.58 CTTGAATAAG CCCGC----- ---------- --CGA----- ----------

mel.02.7 CTTGAATAAG CCCGC----- ---------- --CGA----- ----------

mel.04.1 CTTGAATAAG CCCGC----- ---------- --CGA----- ----------

mel.04.7 CTTGAATAAG CCCGC----- ---------- --CGA----- ----------

mel.05.13 CTTGAATAAG CCCGC----- ---------- --CGA----- ----------

mel.05.16 CTTGAATAAG CCCGC----- ---------- --CGA----- ----------

mel.07.20 CTTGAATAAG CCCGC----- ---------- --CGA----- ----------

mel.14.26 CTTGAATAAG CCCGC----- ---------- --CGA----- ----------

mel.17.66 CTTGAATAAG CCCGC----- ---------- --CGA----- ----------

mel.18Ug.7 CTTGAATAAG CCCGC----- ---------- --CGA----- ----------

mel.19.113 CTTGAATAAG CCCGC----- ---------- --CGA----- ----------

mel.23.74 CTTGAATAAG CCCGC----- ---------- --CGA----- ----------

mel.24.81 CTTGAATAAG CCCGC----- ---------- --CGA----- ----------

mel.26.32 CTTGAATAAG CCCGC----- ---------- --CGA----- ----------

mel.34.9 CTTGAATAAG CCCGC----- ---------- --CGA----- ----------

mel.39.37 CTTGAATAAG CCCGC----- ---------- --CGA----- ----------

mel.40Ug.12 CTTGAATAAG CCCGC----- ---------- --CGA----- ----------

mel.45.89 CTTGAATAAG CCCGC----- ---------- --CGA----- ----------

mel.CanS.41 CTTGAATAAG CCCGC----- ---------- --CGA----- ----------

mel.53.105 CTTGAATAAG CCCGC----- ---------- --CGA----- ----------

mel.54Ug.1 CTTGAATAAG CCCGC----- ---------- --CGA----- ----------

mel.59.3 CTTGAATAAG CCCGC----- ---------- --CGA----- ----------

mel.yw.50 CTTGAATAAG CCCGC----- ---------- --CGA----- ----------

mel.WI83.25 CTTGAATAAG CCCGC----- ---------- --CGA----- ----------

mel.51.100 CTTGAATAAG CCCGC----- ---------- --CGA----- ----------

mel.21.2 CTTGAATAAG CCCGC----- ---------- --CGA----- ----------

mel.29.3 CTTGAATAAG CCCGC----- ---------- --CGA----- ----------

mel.64.3 CTTGAATAAG CCCGC----- ---------- --CGA----- ----------

mel.67.1 CTTGAATAAG CCCGC----- ---------- --CGA----- ----------

mel.55.2 CTTGAATAAG CCCGC----- ---------- --CGA----- ----------

**Closely-Related**

**Outgroup Species**

mau.5 CTTGAATAAG GCCGC----- ---------- --AGA----- ----------

sec.38 CTTGAATAAG GCCGC----- ---------- --AGA----- ----------

sim.33 CTTGAATAAG GCCGC----- ---------- --AGA----- ----------

**Outgroup Species**

yak.25 CTTGAATAGG GCCGCTGCCG CTGGCTGAGC GTAGA----- ----------

luc.41 CTTGAATAAG GCCGCTGAC- -----TGAGC GCAGA----- ----------

eug.20 CTTGAATAAG GCCGCTGCC- -----TGgct gagaccgacc agtcggttgg

fuy.9 CTTGAATAAG GCCGCTGAC- -----TGAGC GaaaA----- ----------

**Abd-B 7**

**"Concestor" 772 ----TGCCAA TAAAAAG-CG GCGTGGCAAA GTGGAGTGGA C------TGG**

**“Concestor2" ----TGCCAA TAAAAAG-CG GCGTGGCAAA GTGGAGTGGA C------TGG**

mel.00.1 ----TGCCAA TAAAAAG-CG GCGTGGCAAA GTGGAGTGGA C------TGG

mel.01.58 ----TGCCAA TAAAAAG-CG GCGTGGCAAA GTGGAGTGGA C------TGG

mel.02.7 ----TGCCAA TAAAAAG-CG GCGTGGCAAA GTGGAGTGGA C------TGG

mel.04.1 ----TGCCAA TAAAAAG-CG GCGTGGCAAA GTGGAGTGGA C------TGG

mel.04.7 ----TGCCAA TAAAAAG-CG GCGTGGCAAA GTGGAGTGGA C------TGG

mel.05.13 ----TGCCAA TAAAAAG-CG GCGTGGCAAA GTGGAGTGGA C------TGG

mel.05.16 ----TGCCAA TAAAAAG-CG GCGTGGCAAA GTGGAGTGGA C------TGG

mel.07.20 ----TGCCAA TAAAAAG-CG GCGTGGCAAA GTGGAGTGGA C------TGG

mel.14.26 ----TGCCAA TAAAAAG-CG GCGTGGCAAA GTGGAGTGGA C------TGG

mel.17.66 ----TGCCAA TAAAAAG-CG GCGTGGCAAA GTGGAGTGGA C------TGG

mel.18Ug.7 ----TGCCAA TAAAAAG-CG GCGTGGCAAA GTGGAGTGGA C------TGG

mel.19.113 ----TGCCAA TAAAAAG-CG GCGTGGCAAA GTGGAGTGGA C------TGG

mel.23.74 ----TGCCAA TAAAAAG-CG GCGTGGCAAA GTGGAGTGGA C------TGG

mel.24.81 ----TGCCAA TAAAAAG-CG GCGTGGCAAA GTGGAGTGGA C------TGG

mel.26.32 ----TGCCAA TAAAAAG-CG GCGTGGCAAA GTGGAGTGGA C------TGG

mel.34.9 ----TGCCAA TAAAAAG-CG GCGTGGCAAA GTGGAGTGGA C------TGG

mel.39.37 ----TGCCAA TAAAAAG-CG GCGTGGCAAA GTGGAGTGGA C------TGG

mel.40Ug.12 ----TGCCAA TAAAAAG-CG GCGTGGCAAA GTGGAGTGGA C------TGG

mel.45.89 ----TGCCAA TAAAAAG-CG GCGTGGCAAA GTGGAGTGGA C------TGG

mel.CanS.41 ----TGCCAA TAAAAAG-CG GCGTGGCAAA GTGGAGTGGA C------TGG

mel.53.105 ----TGCCAA TAAAAAG-CG GCGTGGCAAA GTGGAGTGGA C------TGG

mel.54Ug.1 ----TGCCAA TAAAAAG-CG GCGTGGCAAA GTGGAGTGGA C------TGG

mel.59.3 ----TGCCAA TAAAAAG-CG GCGTGGCAAA GTGGAGTGGA C------TGG

mel.yw.50 ----TGCCAA TAAAAAG-CG GCGTGGCAAA GTGGAGTGGA C------TGG

mel.WI83.25 ----TGCCAA TAAAAAG-CG GCGTGGCAAA GTGGAGTGGA C------TGG

mel.51.100 ----TGCCAA TAAAAAG-CG GCGTGGCAAA GTGGAGTGGA C------TGG

mel.21.2 ----TGCCAA TAAAAAG-CG GCGTGGCAAA GTGGAGTGGA C------TGG

mel.29.3 ----TGCCAA TAAAAAG-CG GCGTGGCAAA GTGGAGTGGA C------TGG

mel.64.3 ----TGCCAA TAAAAAG-CG GCGTGGCAAA GTGGAGTGGA C------TGG

mel.67.1 ----TGCCAA TAAAAAG-CG GCGTGGCAAA GTGGAGTGGA C------TGG

mel.55.2 ----TGCCAA TAAAAAG-CG GCGTGGCAAA GTGGAGTGGA C------TGG

**Closely-Related**

**Outgroup Species**

mau.5 ----AGCCAA TAAAAAGCCG GCGTGGCAAA GTGGAGTGGA T------TGG

sec.38 ----AGCCAA TAAAAAGCCG GCGTGGCAAA GTGGAGTGGA T------TGG

sim.33 ----AGCCAA TAAAAAGCCG GCGTGGCAAA GTGGAGTGGA T------TGG

**Outgroup Species**

yak.25 ----AACCAA TAAAAAA-TG CCGGGGCAAA GTGGAG---- --TGGATTTC

luc.41 ----GGCCCA TAAAAA---- -CGAGGCAAA GTGGAGTGGC TGTGGATTTT

eug.20 CAGGAGCCAA TAAAAGTTGG CCGGGGCAAA GTGGA----- -GTGGATTTT

fuy.9 ----AGCCAA TAAAAAG-TG CCGAGGCAAA GTGGAGTGGA T------TTT

**F** **Abd-B 8**

**"Concestor" 811 GGATGTGTGG CGCCC----C TGCTAGTGGC ACATAAAAAT TGGCGCAAGT**

**"Concestor2" GGATGTGTGG CGCCC----C TGCTAGTGGC ACATAAAAAT TGGCGCAAGT**

mel.00.1 GTTTGTGTGG CGCCC----C TGCTAGTGGC ACATAAAAAT TGGCGCAAGT

mel.01.58 GTTTGTGTGG CGCCC----C TGCTAGTGGC ACATAAAAAT TGGCGCAAGT

mel.02.7 GGATGTGTGG CGCCC----C TGCTTGTGGC ACATAAAAAT TGGCGCAAGT

mel.04.1 G**TT**TGTGTGG CGCCC----C TGCTAGTGGC ACATAAAAAT TGGCGCAAGT

mel.04.7 GTTTGTGTGG CGCCC----C TGCTAGTGGC ACATAAAAAT TGGCGCAAGT

mel.05.13 GTTTGTGTGG CGCCC----C TGCTAGTGGC ACATAAAAAT TGGCGCAAGT

mel.05.16 GTTTGTGTGG CGCCC----C TGCTAGTGGC ACATAAAAAT TGGCGCAAGT

mel.07.20 GTTTGTGTGG CGCCC----C TGCTAGTGGC ACATAAAAAT TGGCGCAAGT

mel.14.26 GTTTGTGTGG CGCCC----C TGCTAGTGGC ACATAAAAAT TGGCGCAAGT

mel.17.66 GTTTGTGTGG CGCCC----C TGCTAGTGGC ACATAAAAAT TGGCGCAAGT

mel.18Ug.7 GGATGTGTGG CGCCC----C TGCTTGTGGC ACATAAAAAT TGGCGCAAGT

mel.19.113 G**TT**TGTGTGG CGCCC----C TGCTAGTGGC ACATAAAAAT TGGCGCAAGT

mel.23.74 GTTTGTGTGG CGCCC----C TGCTAGTGGC ACATAAAAAT TGGCGCAAGT

mel.24.81 GGTTGTGTGG CGCCC----C TGCTAGTGGC ACATAAAAAT TGGCGCAAGT

mel.26.32 GGTTGTGTGG CGCCC----C TGCTAGTGGC ACATAAAAAT TGGCGCAAGT

mel.34.9 GTTTGTGTGG CGCCC----C TGCTAGTGGC ACATAAAAAT TGGCGCAAGT

mel.39.37 GTTTGTGTGG CGCCC----C TGCTAGTGGC ACATAAAAAT TGGCGCAAGT

mel.40Ug.12 GGTTGTGTGG CGCCC----C TGCTAGTGGC ACATAAAAAT TGGCGCAAGT

mel.45.89 GTTTGTGTGG CGCCC----C TGCTAGTGGC ACATAAAAAT TGGCGCAAGT

mel.CanS.41 GTTTGTGTGG CGCCC----C TGCTAGTGGC ACATAAAAAT TGGCGCAAGT

mel.53.105 GTTTGTGTGG CGCCC----C TGCTAGTGGC ACATAAAAAT TGGCGCAAGT

mel.54Ug.1 GGATGTGTGG CGCCC----C TGCTAGTGGC ACATAAAAAT TGGCGCAAGT

mel.59.3 GGATGTGTGG CGCCC----C TGCTAGTGGC ACATAAAAAT TGGCGCAAGT

mel.yw.50 GTTTGTGTGG CGCCC----C TGCTAGTGGC ACATAAAAAT TGGCGCAAGT

mel.WI83.25 GTTTGTGTGG CGCCC----C TGCTAGTGGC ACATAAAAAT TGGCGCAAGT

mel.51.100 GGTTGTGTGG CGCCC----C TGCTAGTGGC ACATAAAAAT TGGCGCAAGT

mel.21.2 GTTTGTGTGG CGCCC----C TGCTAGTGGC ACATAAAAAT TGGCGCAAGT

mel.29.3 GTTTGTGTGG CGCCC----C TGCTAGTGGC ACATAAAAAT TGGCGCAAGT

mel.64.3 GGATGTGTGG CGCCC----C TGCTAGTGGC ACATAAAAAT TGGCGCAAGT

mel.67.1 GTTTGTGTGG CGCCC----C TGCTAGTGGC ACATAAAAAT TGGCGCAAGT

mel.55.2 GTTTGTGTGG CGCCC----C TGCTAGTGGC ACATAAAAAT TGGCGCAAGT

**Closely-Related**

**Outgroup Species F**

mau.5 CGATGTGTGG CGCCC----C AGCTAGTGGC ACATAAAAAT TGGCGCAAGT

sec.38 CGATGTGTGG CGCCC----C AGCTAGTGGC ACATAAAAAT TGGCGCAAGT

sim.33 CGATGTGTGG CGCCC----C GGCTAGTGGC ACATAAAAAT TGGCGCAAGT

**Outgroup Species**

yak.25 GGATGTGTGG CGCCT----C TGCTAGTGGC ACATAAAAAT TGGTGCAAGT

luc.41 GGCCGTGTGG CGCCC----C TTGTGGTGCA ACATAAAAAT TGGCGCAAGT

eug.20 GGCCGTGTGG CGCCCC-TGC CACTAGTGCC ACATAAAAGT TGGCGCAAGT

fuy.9 GGCCGTGTGG CGCCCCGTGC TGCTAGTGGC ACATAAAAAT TGGCGCAAGT

**Abd-B 9 G**

**"Concestor" 857 TAATTGTGGT AGTTATTTGC TG-TTTTGCC ATTTGGTCAT TTTACAATTT**

**"Concestor2" TAATTGTGGT AGTTATTTGC TG-TTTTGCC ATTTGGTCAT TTTACAATTT**

mel.00.1 TAATTGTGGT AGTTATTTGC TG-TTTTGCC ATTTGGTCAT TTTACAATTT

mel.01.58 TAATTGTGGT AGTTATTTGC TG-TTTTGCC ATTTGGTCAT TTTACAATTT

mel.02.7 TAATTGTGGT AGTTATTTGC TG-TTTTGCC ATTTGGTCAT TTTACAATTT

mel.04.1 TAATTGTGGT AGTTATTTGC TG-TTTTGCC ATTTGGTCAT TTTACAATTT

mel.04.7 TAATTGTGGT AGTTATTTGC TG-TTTTGCC ATTTGGTCAT TTTACAATTT

mel.05.13 TAATTGTGGT AGTTATTTGC TG-TTTTGCC ATTTGGTCAT TTTACAATTT

mel.05.16 TAATTGTGGT AGTTATTTGC TG-TTTTGCC ATTTGGTCAT TTTACAATTT

mel.07.20 TAATTGTGGT AGTTATTTGC TG-TTTTGCC ATTTGGTCAT TTTACAATTT

mel.14.26 TAATTGTGGT AGTTATTTGC TG-TTTTGCC ATTTGGTCAT TTTACAATTT

mel.17.66 TAATTGTGGT AGTTATTTGC TG-TTTTGCC ATTTGGTCAT TTTACAATTT

mel.18Ug.7 TAATTGTGGT AGTTATTTGC TG-TTTTGCC ATTTGGTCAT TTTACAATTT

mel.19.113 TAATTGTGGT AGTTATTTGC TG-TTTTGCC ATTTGGTCAT TTTACAATTT

mel.23.74 TAATTGTGGT AGTTATTTGC TG-TTTTGCC ATTTGGTCAT TTTACAATTT

mel.24.81 TAATTGTGGT AGTTATTTGC TG-TTTTGCC ATTTGGTCAT TTTACAATTG

mel.26.32 TAATTGTGGC AGTTATTTGC TG-TTTTGCC ATTTGGTCAT TTTACAATTG

mel.34.9 TAATTGTGGT AGTTATTTGC TG-TTTTGCC ATTTGGTCAT TTTACAATTT

mel.39.37 TAATTGTGGT AGTTATTTGC TG-TTTTGCC ATTTGGTCAT TTTACAATTT

mel.40Ug.12 TAATTGTGGT AGTTATTTGC TG-TTTTGCC ATTTGGTCAT TTTACAATTG

mel.45.89 TAATTGTGGT AGTTATTTGC TG-TTTTGCC ATTTGGTCAT TTTACAATTT

mel.CanS.41 TAATTGTGGT AGTTATTTGC TG-TTTTGCC ATTTGGTCAT TTTACAATTT

mel.53.105 TAATTGTGGT AGTTATTTGC TG-TTTTGCC ATTTGGTCAT TTTACAATTT

mel.54Ug.1 TAATTGTGGT AGTTATTTGC TG-TTTTGCC ATTTGGTCAT TTTACAATT**A**

mel.59.3 TAATTGTGGT AGTTATTTGC TG-TTTTGCC ATTTGGTCAT TTTACAATT**G**

mel.yw.50 TAATTGTGGT AGTTATTTGC TG-TTTTGCC ATTTGGTCAT TTTACAATTT

mel.WI83.25 TAATTGTGGT AGTTATTTGC TG-TTTTGCC ATTTGGTCAT TTTACAATTT

mel.51.100 TAATTGTGGT AGTTATTTGC TG-TTTTGCC ATTTGGTCAT TTTACAATTT

mel.21.2 TAATTGTGGT AGTTATTTGC TG-TTTTGCC ATTTGGTCAT TTTACAATTT

mel.29.3 TAATTGTGGT AGTTATTTGC TG-TTTTGCC ATTTGGTCAT TTTACAATT**G**

mel.64.3 TAATTGTGGT AGTTATTTGC TG-TTTTGCC ATTTGGTCAT TTTACAATTT

mel.67.1 TAATTGTGGT AGTTATTTGC TG-TTTTGCC ATTTGGTCAT TTTACAATTT

mel.55.2 TAATTGTGGT AGTTATTTGC TG-TTTTGCC ATTTGGTCAT TTTACAATTT

**Closely-Related**

**Outgroup Species G**

mau.5 TAATTGTGGT AGTTATTTGC TG-TTTTGCC ATTTGGTCAT TTTACAATTT

sec.38 TAATTGTGGT AGTTATTTGC TG-TTTTGCC ATTTGGTCAT TTTACAATTT

sim.33 TAATTGTGGT AGTTATTTGC TG-TTTTGCC ATTTGGTCAT TTTACAATTT

**Outgroup Species**

yak.25 TAATTGTGGT AGTTATTTGC TGTT-TTGCC ATTTGGTCAT TTTACAATTT

luc.41 TAATTGTGGT AGTTATTTGC TGTTTTTGCC ATTTGGTCAT TTTACAATTT

eug.20 TAATTGTGGT AGTTATTTGC TGTT-TTGCC ATTTGGTCAT TTTACAATTT

fuy.9 TAATTGTGGT AGTTATTTGC TGTT-TTGCC ATTTGGCCGT TTTACAATTT

**Abd-B 10**

**"Concestor" 906 TACCATTTCA GC-------- ------CACA ACTT------ -----TTCGC**

**"Concestor2" TACCATTTCA GC-------- ------CACA ACTT------ -----TTCGC**

mel.00.1 TACCATTTCA GC-------- ------CACA ACTT------ -----TTCGC

mel.01.58 TACCATTTCA GC-------- ------CACA ACTT------ -----TTCGC

mel.02.7 TACCATTTCA GC-------- ------CACA ACTT------ -----TTCGC

mel.04.1 TACCATTTCA GC-------- ------CACA ACTT------ -----TTCGC

mel.04.7 TACCATTTCA GC-------- ------CACA ACTT------ -----TTCGC

mel.05.13 TACCATTTCA GC-------- ------CACA ACTT------ -----TTCGC

mel.05.16 TACCATTTCA GC-------- ------CACA ACTT------ -----TTCGC

mel.07.20 TACCATTTCA GC-------- ------CACA ACTT------ -----TTCGC

mel.14.26 TACCATTTCA GC-------- ------CACA ACTT------ -----TTCGC

mel.17.66 TACCATTTCA GC-------- ------CACA ACTT------ -----TTCGC

mel.18Ug.7 TACCATTTCA GC-------- ------CACA ACTT------ -----TTCGC

mel.19.113 TACCATTTCA GC-------- ------CACA ACTT------ -----TTCGC

mel.23.74 TACCATTTCA GC-------- ------CACA ACTT------ -----TTCGC

mel.24.81 TACCATTTCA GC-------- ------CACA ACTT------ -----TTCGC

mel.26.32 TACCATTTCA GC-------- ------CACA ACTT------ -----TTCGC

mel.34.9 TACCATTTCA GC-------- ------CACA ACTT------ -----TTCGC

mel.39.37 TACCATTTCA GC-------- ------CACA ACTT------ -----TTCGC

mel.40Ug.12 TACCATTTCA GC-------- ------CACA ACTT------ -----TTCGC

mel.45.89 TACCATTTCA GC-------- ------CACA ACTT------ -----TTCGC

mel.CanS.41 TACCATTTCA GC-------- ------CACA ACTT------ -----TTCGC

mel.53.105 TACCATTTCA GC-------- ------CACA ACTT------ -----TTCGC

mel.54Ug.1 TACCATTTCA GC-------- ------CACA ACTT------ -----TTCGC

mel.59.3 TACCATTTCA GC-------- ------CACA ACTT------ -----TTCGC

mel.yw.50 TACCATTTCA GC-------- ------CACA ACTT------ -----TTCGC

mel.WI83.25 TACCATTTCA GC-------- ------CACA ACTT------ -----TTCGC

mel.51.100 TACCATTTCA GC-------- ------CACA ACTT------ -----TTCGC

mel.21.2 TACCATTTCA GC-------- ------CACA ACTT------ -----TTCGC

mel.29.3 TACCATTTCA GC-------- ------CACA ACTT------ -----TTCGC

mel.64.3 TACCATTTCA GC-------- ------CACA ACTT------ -----TTCGC

mel.67.1 TACCATTTCA GC-------- ------CACA ACTT------ -----TTCGC

mel.55.2 TACCATTTCA GC-------- ------CACA ACTT------ -----TTCGC

**Closely-Related**

**Outgroup Species**

mau.5 TACCccctct ccacc----- ---------- ---------- ----------

sec.38 TACCATTTCA GC-------- ------CACA ACTT------ -----TTCGC

sim.33 TACCATTTCA GC-------- ------CACA ACTT------ -----TTCTC

**Outgroup Species**

yak.25 TACCATTTCA CCAT------ TTCAGCCACA ACTT------ -----TTAGC

luc.41 TACCATTTTA CCAT------ TTGAGCCACA gcacaacttt tcgcaTTCGC

eug.20 TACCATTTTC CATTCTGCCA TTCTGCCACA ACTT------ -----TTTCC

fuy.9 TACCATTct- GC-------- ------CACA ACTT------ -----TTCGC

**H** **I**  **J** **Dsx2 Site**

**"Concestor" 931 ACTGCTCCCC CCC------- --------TC TCCCAGCACA ACAATGTTGC**

**"Concestor2" ACTGCTCCCC CCC------- --------TC TCCCAGCACA ACAATGTTGC**

mel.00.1 ACTGCTCCCC CCC------- --------TT TCCCAGCACA ACAATGTTGC

mel.01.58 ACTGCTCCCC CCCCC----- --------TT TCCCAGCACA ACAATGTTGC

mel.02.7 ACTGCTCCCC CCCCC----- --------TC TCCCAGTACA ACAATGTTGC

mel.04.1 ACTGCTCCCC CCC------- --------TT TCCCAGCACA ACAATGTTGC

mel.04.7 ACTGCTCCCC CCC------- --------TT TCCCAGCACA ACAATGTTGC

mel.05.13 ACTGCTCCCC CCC------- --------TT TCCCAGCACA ACAATGTTGC

mel.05.16 ACTGCTCCCC CCC------- --------TT TCCCAGCACA ACAATGTTGC

mel.07.20 ACTGCTCCCC CCC------- --------TT TCCCAGCACA ACAATGTTGC

mel.14.26 ACTGCTCCCC CCCCCCCCCC --------TC TCCCAGCACA ACAATGTTGC

mel.17.66 ACTGCTCCCC CCCCCC---- --------TC TCCCAGTACA ACAATGTTGC

mel.18Ug.7 ACTGCTCCCC CCCCCCCC-- --------TC TCCCAGCACA ACAATGTTGC

mel.19.113 ACTGCTCCCC CCC**C------** --------TC TCCCAG**T**ACA ACAATGTTGC

mel.23.74 ACTGCTCCCC CCC------- --------TT TCCCAGCACA ACAATGTTGC

mel.24.81 ACTGCTCCCC CCCCC----- --------TC TCCCAGCACA ACAATGTTGC

mel.26.32 ACTGCTCCCC CCCC------ --------TC TCCCAGTACA ACAATGTTGC

mel.34.9 ACTGCTCCCC CCC------- --------TT TCCCAGCACA ACAATGTTGC

mel.39.37 ACTGCTCCCC CCC------- --------TT TCCCAGCACA ACAATGTTGC

mel.40Ug.12 ACTGCTCCCC CCC------- --------TT TCCCAGCACA ACAATGTTGC

mel.45.89 ACTGCTCCCC CCC------- --------TT TCCCAGCACA ACAATGTTGC

mel.CanS.41 ACTGCTCCCC CCC------- --------TT TCCCAGCACA ACAATGTTGC

mel.53.105 ACTGCTCCCC CCC------- --------TT TCCCAGCACA ACAATGTTGC

mel.54Ug.1 ACTGCTCCCC CCC**CCCC--- --------**TC TCCCAGCACA ACAATGTTGC

mel.59.3 ACTGCTCCCC CCC**CCC---- --------**TC TCCCAGCACA ACAATGTTGC

mel.yw.50 ACTGCTCCCC CCCCCC---- --------TC TCCCAGCACA ACAATGTTGC

mel.WI83.25 ACTGCTCCCC CCC------- --------TT TCCCAGCACA ACAATGTTGC

mel.51.100 ACTGCTCCCC CCC------- --------TT TCCCAGCACA ACAATGTTGC

mel.21.2 ACTGCTCCCC CCC------- --------TT TCCCAGCACA ACAATGTTGC

mel.29.3 ACTGCTCCCC CCC------- --------TT TCCCAGCACA ACAATGTTGC

mel.64.3 ACTGCTCCCC CCC------- --------TC TCCCAGCACA ACAATGTTGC

mel.67.1 ACTGCTCCCC CCC------- --------TT TCCCAGCACA ACAATGTTGC

mel.55.2 ACTGCTCCCC CCC------- --------TT TCCCAGCACA ACAATGTTGC

**Closely-Related**

**Outgroup Species H I J**

mau.5 -----CCGCC CCTC------ ---------A ACCCAACGCA ACAATGTTGC

sec.38 ACTGCCACCC CCTCTCCACC CCGCCCAACA ACCCAAC**GCA** ACAATGTTGC

sim.33 ACTGCCACCC CCTCTCCACC CCGCCCCTCA ACCCAAC**GCA** ACAATGTTGC

**Outgroup Species**

yak.25 **ACTGCTCCC**- ---------- --TTCG---- -CCCGACGCA ACAATGTTGC

luc.41 ATTGCTCCGT TT-------- ---------- -GCAACATTG TTGCGTTTGC

eug.20 AACTG----- ---------- ---------- -----GTGCA ACATTGTTGC

fuy.9 ATTGCTCCGC TTGCCTGGTG CAACAATGT- GC-------- -CGCAGTCGC

**Abd-B 11** **K** **Abd-B 12**

**"Concestor" 960 GGCATTCTCG CAC-TTT-A- CGAGGCG--- TTTTTTT--A TATCACTTAC**

**"Concestor2" GGCATTCTCG CAC-TTT-A- CGAGGCG--- TTTTTTT--A TATCACTTAC**

mel.00.1 GGCATTCTCG CAC-TTT-A- CGAGGCG**TTT** TTTTTTT--A TATCACTTAC

mel.01.58 GGCATTCTCG CAC-TTT-A- CGAGGCG**TTT** TTTTTTT--A TATCACTTAC

mel.02.7 GGCATTCTCG CAC-TTT-AG CGAGGCG-TT TTTTTTT--A TATCACTTAC

mel.04.1 GGCATTCTCG CAC-TTT-A- CGAGGCG**TTT** TTTTTTT--A TATCACTTAC

mel.04.7 GGCATTCTCG CAC-TTT-A- CGAGGCG**TTT** TTTTTTT--A TATCACTTAC

mel.05.13 GGCATTCTCG CAC-TTT-A- CGAGGCG**TTT** TTTTTTT--A TATCACTTAC

mel.05.16 GGCATTCTCG CAC-TTT-A- CGAGGCG**TTT** TTTTTTT--A TATCACTTAC

mel.07.20 GGCATTCTCG CAC-TTT-A- CGAGGCG**TTT** TTTTTTT--A TATCACTTAC

mel.14.26 GGCATTCTCG CAC-TTT-A- CGAGGCG**TTT** TTTTTTT--A TATCACTTAC

mel.17.66 GGCATTCTCG CAC-TTT-A- CGAGGCG**TTT** TTTTTTT--A TATCACTTAC

mel.18Ug.7 GGCATTCTCG CAC-TTT-A- CGAGGCG**TTT** TTTTTTT--A TATCACTTAC

mel.19.113 GGCATTCTCG CAC-TTT-A- CGAGGCG-**TT** TTTTTTT--A TATCACTTAC

mel.23.74 GGCATTCTCG CAC-TTT-A- CGAGGCG**TTT** TTTTTTT--A TATCACTTAC

mel.24.81 GGCATTCTCG CAC-TTT-A- CGAGGCG**TTT** TTTTTTT--A TATCACTTAC

mel.26.32 GGCATTCTCG CAC-TTT-A- CGAGGCG-TT TTTTTTT--A TATCACTTAC

mel.34.9 GGCATTCTCG CAC-TTT-A- CGAGGCG**TTT** TTTTTTT--A TATCACTTAC

mel.39.37 GGCATTCTCG CAC-TTT-A- CGAGGCG**TTT** TTTTTTT--A TATCACTTAC

mel.40Ug.12 GGCATTCTCG CAC-TTT-A- CGAGGCG**TTT** TTTTTTT--A TATCACTTAC

mel.45.89 GGCATTCTCG CAC-TTT-A- CGAGGCG**TTT** TTTTTTT--A TATCACTTAC

mel.CanS.41 GGCATTCTCG CAC-TTT-A- CGAGGCG**TTT** TTTTTTT--A TATCACTTAC

mel.53.105 GGCATTCTCG CAC-TTT-A- CGAGGCG**TTT** TTTTTTT--A TATCACTTAC

mel.54Ug.1 GGCATTCTCG CAC-TTT-A- CGAGGCG--**T** TTTTTTT--A TATCACTTAC

mel.59.3 GGCATTCTCG CAC-TTT-A- CGAGGCG-**TT** TTTTTTT--A TATCACTTAC

mel.yw.50 GGCATTCTCG CAC-TTT-A- CGAGGCG**TTT** TTTTTTT--A TATCACTTAC

mel.WI83.25 GGCATTCTCG CAC-TTT-A- CGAGGCG**TTT** TTTTTTT--A TATCACTTAC

mel.51.100 GGCATTCTCG CAC-TTT-A- CGAGGCG-TT TTTTTTT—-A TATCACTTAC

mel.21.2 GGCATTCTCG CAC-TTT-A- CGAGGCG**TTT** TTTTTTT--A TATCACTTAC

mel.29.3 GGCATTCTCG CAC-TTT-A- CGAGGCG--**T** TTTTTTT--A TATCACTTAC

mel.64.3 GGCATTCTCG CAC-TTT-A- CGAGGCG--**T** TTTTTTT--A TATCACTTAC

mel.67.1 GGCATTCTCG CAC-TTT-A- CGAGGCG**TTT** TTTTTTT--A TATCACTTAC

mel.55.2 GGCATTCTCG CAC-TTT-A- CGAGGCG**TTT** TTTTTTT--A TATCACTTAC

**Closely-Related**

**Outgroup Species K**

mau.5 GGCATTCTCG CAC-TTT-A- CGAGGCG--- -TTTTTT--A TATCACTTAC

sec.38 GGCATTCTCG CAC-TTT-A- CGAGGCG--- -TTTTTT--A TATCACTTAC

sim.33 GGCATTCTCG CAC-TTT-A- CGAGGCG--- -TTTTTT--A TATCACTTAC

**Outgroup Species**

yak.25 GGCATTCTCG CAC-TTT-A- CGAGGCG--- -TTTTTT--A TATCAC----

luc.41 TGCATTCTCG CAC-TTTTA- CGAGGCG--- -TTTTTT--A TATCAC----

eug.20 CGCATTCTCG CAC-TTTTA- CGAGGCG--- --TTTTT--A TATCAC----

fuy.9 TGCATTCTCG CACtTTT-A- CGAGGCG--- -TTTTTT--A TATCAC----

**Abd-B 13 L**

**"Concestor" 1002 TTTACTTAGT TGATTAAGGG CGTGGCCGAT GGGCCAGATA CATGCTTAGA**

**"Concestor2" TTTACTTAGT TGATTAAGGG CGTGGCCGAT GGGCCAGATA CATGCTTAGA**

mel.00.1 TTTACTTAGT TGATTAAGGG CGTGGCCGAT GGGCCAGATA CATGCTTAGA

mel.01.58 TTTACTTAGT TGATTAAGGG CGTGGCCGAT GGGCCAGATA CATGCTTAGA

mel.02.7 TTTACTTAGT TGATT**G**AGGG CGTGGCCGAT GGGCCAGATA CATGCTTAGA

mel.04.1 TTTACTTAGT TGATTAAGGG CGTGGCCGAT GGGCCAGATA CATGCTTAGA

mel.04.7 TTTACTTAGT TGATTAAGGG CGTGGCCGAT GGGCCAGATA CATGCTTAGA

mel.05.13 TTTACTTAGT TGATTAAGGG CGTGGCCGAT GGGCCAGATA CATGCTTAGA

mel.05.16 TTTACTTAGT TGATTAAGGG CGTGGCCGAT GGGCCAGATA CATGCTTAGA

mel.07.20 TTTACTTAGT TGATTAAGGG CGTGGCCGAT GGGCCAGATA CATGCTTAGA

mel.14.26 TTTACTTAGT TGATTAAGGG CGTGGCCGAT GGGCCAGATA CATGCTTAGA

mel.17.66 TTTACTTAGT TGATTAAGGG CGTGGCCGAT GGGCCAGATA CATGCTTAGA

mel.18Ug.7 TTTACTTAGT TGATTAAGGG CGTGGCCGAT GGGCCAGATA CATGCTTAGA

mel.19.113 TTTACTTAGT TGATT**G**AGGG CGTGGCCGAT GGGCCAGATA CATGCTTAGA

mel.23.74 TTTACTTAGT TGATTAAGGG CGTGGCCGAT GGGCCAGATA CATGCTTAGA

mel.24.81 TTTACTTAGT TGATTAAGGG CGTGGCCGAT GGGCCAGATA CATGCTTAGA

mel.26.32 TTTACTTAGT TGATTAAGGG CGTGGCCGAT GGGCCAGATA CATGCTTAGA

mel.34.9 TTTACTTAGT TGATTAAGGG CGTGGCCGAT GGGCCAGATA CATGCTTAGA

mel.39.37 TTTACTTAGT TGATTAAGGG CGTGGCCGAT GGGCCAGATA CATGCTTAGA

mel.40Ug.12 TTTACTTAGT TGATTAAGGG CGTGGCCGAT GGGCCAGATA CATGCTTAGA

mel.45.89 TTTACTTAGT TGATTAAGGG CGTGGCCGAT GGGCCAGATA CATGCTTAGA

mel.CanS.41 TTTACTTAGT TGATTAAGGG CGTGGCCGAT GGGCCAGATA CATGCTTAGA

mel.53.105 TTTACTTAGT TGATTAAGGG CGTGGCCGAT GGGCCAGATA CATGCTTAGA

mel.54Ug.1 TTTACTTAGT TGATTAAGGG CGTGGCCGAT GGGCCAGATA CATGCTTAGA

mel.59.3 TTTACTTAGT TGATTAAGGG CGTGGCCGAT GGGCCAGATA CATGCTTAGA

mel.yw.50 TTTACTTAGT TGATTAAGGG CGTGGCCGAT GGGCCAGATA CATGCTTAGA

mel.WI83.25 TTTACTTAGT TGATTAAGGG CGTGGCCGAT GGGCCAGATA CATGCTTAGA

mel.51.100 TTTACTTAGT TGATTAAGGG CGTGGCCGAT GGGCCAGATA CATGCTTAGA

mel.21.2 TTTACTTAGT TGATTAAGGG CGTGGCCGAT GGGCCAGATA CATGCTTAGA

mel.29.3 TTTACTTAGT TGATTAAGGG CGTGGCCGAT GGGCCAGATA CATGCTTAGA

mel.64.3 TTTACTTAGT TGATTAAGGG CGTGGCCGAT GGGCCAGATA CATGCTTAGA

mel.67.1 TTTACTTAGT TGATTAAGGG CGTGGCCGAT GGGCCAGATA CATGCTTAGA

mel.55.2 TTTACTTAGT TGATTAAGGG CGTGGCCGAT GGGCCAGATA CATGCTTAGA

**Closely-Related**

**Outgroup Species L**

mau.5 TTTACTTAGT TGATTAAGGG CGTGGCCGAT GGGCCAGATA --TGCTTAGA

sec.38 TTTACTTAGT TGATTAAGGG CGTGGCCGAT GGGCCAGATA --TGCTTAGA

sim.33 TTTACTTAGT TGATTAAGGG CGTGGCCGAT GGGCCAGATA --TGCTTAGA

**Outgroup Species**

yak.25 -TTACTTAGT TGATTAAGGG CGTTGCCGAT GGGCCAGATA --TGCTTAGA

luc.41 -TTACTTAGT TGATTAAGGG CGTGGCCGAT GGGCCAGATA --TGTTTAGA

eug.20 -TTACTTAGT TGATTAAGGG CGTGGCCGAT GGGCGAGATA --TGTTTAGA

fuy.9 -TTACTTAGT TGATTAAGGG CGTGGCCGAT GGGAAAGATA --TGTTTAGA

**M**

**"Concestor" 1052 TTTGCTCC-- ---------- ---------- ---AG----C AGTGGGCTGC**

**"Concestor2" TTTGCTCC-- ---------- ---------- ---AG----C AGTGGGCTGC**

mel.00.1 TTTGCTCC-- ---------- ---------- ---AG----C AGTGGGCTGC

mel.01.58 TTTGCTCC-- ---------- ---------- ---AG----C AGTGGGCTGC

mel.02.7 TTTGCTCC-- ---------- ---------- ---AG----C AGTGGGCTGC

mel.04.1 TTTGCTCC-- ---------- ---------- ---AG----C AGTGGGCTGC

mel.04.7 TTTGCTCC-- ---------- ---------- ---AG----C AGTGGGCTGC

mel.05.13 TTTGCTCC-- ---------- ---------- ---AG----C AGTGGGCTGC

mel.05.16 TTTGCTCC-- ---------- ---------- ---AG----C AGTGGGCTGC

mel.07.20 TTTGCTCC-- ---------- ---------- ---AG----C AGTGGGCTGC

mel.14.26 TTTGCTCC-- ---------- ---------- ---AG----C AGTGGGCTGC

mel.17.66 TTTGCTCC-- ---------- ---------- ---AG----C AGTGGGCTGC

mel.18Ug.7 TTTGCTCC-- ---------- ---------- ---AG----C AGTGGGCTGC

mel.19.113 TTTGCTCC-- ---------- ---------- ---AG----C AGTGGGCTGC

mel.23.74 TTTGCTCC-- ---------- ---------- ---AG----C AGTGGGCTGC

mel.24.81 TTTGCTCC-- ---------- ---------- ---AG----C AGTGGGCTGC

mel.26.32 TTTGCTCC-- ---------- ---------- ---AG----C AGTGGGCTGC

mel.34.9 TTTGCTCC-- ---------- ---------- ---AG----C AGTGGGCTGC

mel.39.37 TTTGCTCC-- ---------- ---------- ---AG----C AGTGGGCTGC

mel.40Ug.12 TTTGCTCC-- ---------- ---------- ---AG----C AGTGGGCTGC

mel.45.89 TTTGCTCC-- ---------- ---------- ---AG----C AGTGGGCTGC

mel.CanS.41 TTTGCTCC-- ---------- ---------- ---AG----C AGTGGGCTGC

mel.53.105 TTTGCTCC-- ---------- ---------- ---AG----C AGTGGGCTGC

mel.54Ug.1 TTTGCTCC-- ---------- ---------- ---AG----C AGTGG**T**CTGC

mel.59.3 TTTGCTCC-- ---------- ---------- ---AG----C AGTGGGCTGC

mel.yw.50 TTTGCTCC-- ---------- ---------- ---AG----C AGTGGGCTGC

mel.WI83.25 TTTGCTCC-- ---------- ---------- ---AG----C AGTGGGCTGC

mel.51.100 TTTGCTCC-- ---------- ---------- ---AG----C AGTGGGCTGC

mel.21.2 TTTGCTCC-- ---------- ---------- ---AG----C AGTGGGCTGC

mel.29.3 TTTGCTCC-- ---------- ---------- ---AG----C AGTGGGCTGC

mel.64.3 TTTGCTCC-- ---------- ---------- ---AG----C AGTGGGCTGC

mel.67.1 TTTGCTCC-- ---------- ---------- ---AG----C AGTGGGCTGC

mel.55.2 TTTGCTCC-- ---------- ---------- ---AG----C AGTGGGCTGC

**Closely-Related**

**Outgroup Species**

mau.5 TTTGCTCTAG C--------- ---------- ---------- ----------

sec.38 TTTGCTGTA- ---------- ---------- ---------- --------GC

sim.33 TTTGCTCTA- ---------- ---------- ---------- --------GC

**Outgroup Species** **M**

yak.25 TTTGGTCTAT GTATCCccgt ---------- ----G----G AGTGGGCTGT

luc.41 TTTGCTCTTT GTATtaggtt tcaggcttca gTCAG----G AGTGGGCTGC

eug.20 TTTGCTCTTT GTATCCcggc attccttcag gttttccccG AGTGGGCTGC

fuy.9 TTTGCTCTTT GTATCCggac attcta---- -TCAG----G AGTGGGTTGC

**Abd-B 14**

**"Concestor" 1073 ATTTTACGAC CCTCA----- --AAACCCGA TCCAAAT--- ----------**

**"Concestor2" ATTTTACGAC CCTCA----- --AAACCCGA TCCAAAT--- ----------**

mel.00.1 ATTTTACGAC CCTCA----- --AAACCCGA TCCAAAT--- ----------

mel.01.58 ATTTTACGAC CCTCA----- --AAACCCGA TCCAAAT--- ----------

mel.02.7 ATTTTACGAC CCTCA----- --AAACCCGA TCCAAAT--- ----------

mel.04.1 ATTTTACGAC CCTCA----- --AAACCCGA TCCAAAT--- ----------

mel.04.7 ATTTTACGAC CCTCA----- --AAACCCGA TCCAAAT--- ----------

mel.05.13 ATTTTACGAC CCTCA----- --AAACCCGA TCCAAAT--- ----------

mel.05.16 ATTTTACGAC CCTCA----- --AAACCCGA TCCAAAT--- ----------

mel.07.20 ATTTTACGAC CCTCA----- --AAACCCGA TCCAAAT--- ----------

mel.14.26 ATTTTACGAC CCTCA----- --AAACCCGA TCCAAAT--- ----------

mel.17.66 ATTTTACGAC CCTCA----- --AAACCCGA TCCAAAT--- ----------

mel.18Ug.7 ATTTTACGAC CCTCA----- --AAACCCGA TCCAAAT--- ----------

mel.19.113 ATTTTACGAC CCTCA----- --AAACCCGA TCCAAAT--- ----------

mel.23.74 ATTTTACGAC CCTCA----- --AAACCCGA TCCAAAT--- ----------

mel.24.81 ATTTTACGAC CCTCA----- --AAACCCGA TCCAAAT--- ----------

mel.26.32 ATTTTACGAC CCTCA----- --AAACCCGA TCCAAAT--- ----------

mel.34.9 ATTTTACGAC CCTCA----- --AAACCCGA TCCAAAT--- ----------

mel.39.37 ATTTTACGAC CCTCA----- --AAACCCGA TCCAAAT--- ----------

mel.40Ug.12 ATTTTACGAC CCTCA----- --AAACCCGA TCCAAAT--- ----------

mel.45.89 ATTTTACGAC CCTCA----- --AAACCCGA TCCAAAT--- ----------

mel.CanS.41 ATTTTACGAC CCTCA----- --AAACCCGA TCCAAAT--- ----------

mel.53.105 ATTTTACGAC CCTCA----- --AAACCCGA TCCAAAT--- ----------

mel.54Ug.1 ATTTTACGAC CCTCA----- --AAACCCGA TCCAAAT--- ----------

mel.59.3 ATTTTACGAC CCTCA----- --AAACCCGA TCCAAAT--- ----------

mel.yw.50 ATTTTACGAC CCTCA----- --AAACCCGA TCCAAAT--- ----------

mel.WI83.25 ATTTTACGAC CCTCA----- --AAACCCGA TCCAAAT--- ----------

mel.51.100 ATTTTACGAC CCTCA----- --AAACCCGA-TCCAAAT--- ----------

mel.21.2 ATTTTACGAC CCTCA----- --AAACCCGA-TCCAAAT--- ----------

mel.29.3 ATTTTACGAC CCTCA----- --AAACCCGA-TCCAAAT--- ----------

mel.64.3 ATTTTACGAC CCTCA----- --AAACCCGA-TCCAAAT--- ----------

mel.67.1 ATTTTACGAC CCTCA----- --AAACCCGA-TCCAAAT--- ----------

mel.55.2 ATTTTACGAC CCTCA----- --AAACCCGA-TCCAAAT--- ----------

**Closely-Related**

**Outgroup Species**

mau.5 GTTTTACGAC CCTCA----- --AAACCCGA ACCAAAT--- ----------

sec.38 GTTTTACGAC CCTCA----- --AAACCCGA TCCAAAT--- ----------

sim.33 GTTTTACGAC CCTCA----- --AAACCCGA TCCAAAT--- ----------

**Outgroup Species**

yak.25 GTTTTACGAC CCTCA----- --AAACCCGA TCGAAACGGA AAGAGAc---

luc.41 ATTTTACGAC CCTCCGAAAC CCAAACCCGA TCGAAAC--- ----------

eug.20 ATTTTACGAC CCTCC----- ---AAGCCGA TCGAAACGGA AACtctgacg

fuy.9 ATTTTACGAC CCGCC----- --AAAGCCGA TCAAAACGGA AACAGAag--

**N**

**"Concestor" 1103 -GGAAAAT-- ------ATGA AAATATGGC- ------TAAT CCGCTTATGA**

**"Concestor2" -GGAAAAT-- ------ATGA AAATATGGC- ------TAAT CCGCTTATGA**

mel.00.1 -GGAAAAT-- ------ATGA AAATACGGC- ------TAAT CCGCTTATGA

mel.01.58 -GGAAAAT-- ------ATGA AAATACGGC- ------TAAT CCGCTTATGA

mel.02.7 -GGATAAT-- ------ATGA AAATATCGC- ------TAAT CCGCTTATGA

mel.04.1 -GGAAAAT-- ------ATGA AAATA**C**GGC- ------TAAT CCGCTTATGA

mel.04.7 -GGAAAAT-- ------ATGA AAATA**C**GGC- ------TAAT CCGCTTATGA

mel.05.13 -GGAAAAT-- ------ATGA AAATACGGC- ------TAAT CCGCTTATGA

mel.05.16 -GGAAAAT-- ------ATGA AAATACGGC- ------TAAT CCGCTTATGA

mel.07.20 -GGAAAAT-- ------ATGA AAATACGGC- ------TAAT CCGCTTATGA

mel.14.26 -GGAAAAT-- ------ATGA AAATACGGG- ------TAAT CCGCTTATGA

mel.17.66 -GGAAAAT-- ------ATGA AAATACGGC- ------TAAT CCGCTTATGA

mel.18Ug.7 -GGAAAAT-- ------ATGA AAATACGGC- ------TAAT CCGCTTATGA

mel.19.113 -GGAAAAT-- ------ATGA AAATACGGC- ------TAAT CCGCTTATGA

mel.23.74 -GGAAAAT-- ------ATGA AAATACGGC- ------TAAT CCGCTTATGA

mel.24.81 -GGAAAAT-- ------ATGA AAATACGGC- ------TAAT CCGCTTATGA

mel.26.32 -GGAAAAT-- ------ATGA AAATACGGC- ------TAAT CCGCTTATGA

mel.34.9 -GGAAAAT-- ------ATGA AAATACGGC- ------TAAT CCGCTTATGA

mel.39.37 -GGAAAAT-- ------ATGA AAATACGGC- ------TAAT CCGCTTATGA

mel.40Ug.12 -GGAAAAT-- ------ATGA AAATACGGC- ------TAAT CCGCTTATGA

mel.45.89 -GGAAAAT-- ------ATGA AAATACGGC- ------TAAT CCGCTTATGA

mel.CanS.41 -GGAAAAT-- ------ATGA AAATACGGC- ------TAAT CCGCTTATGA

mel.53.105 -GGAAAAT-- ------ATGA AAATACGGC- ------TAAT CCGCTTATGA

mel.54Ug.1 -GGAAAAT-- ------ATGA AAATATGGC- ------TAAT CCGCTTATGA

mel.59.3 -GGAAAAT-- ------ATGA AAATATGGC- ------TAAT CCGCTTATGA

mel.yw.50 -GGAAAAT-- ------ATGA AAATACGGC- ------TAAT CCGCTTATGA

mel.WI83.25 -GGAAAAT-- ------ATGA AAATACGGC- ------TAAT CCGCTTATGA

mel.51.100 -GGAAAAT-- ------ATGA AAATATGGC- ------TAAT CCGCTTATGA

mel.21.2 -GGAAAAT-- ------ATGA AAATACGGC- ------TAAT CCGCTTATGA

mel.29.3 -GGAAAAT-- ------ATGA AAATACGGC- ------TAAT CCGCTTATGA

mel.64.3 -GGAAAAT-- ------ATGA AAATATGGC- ------TAAT CCGCTTATGA

mel.67.1 -GGAAAAT-- ------ATGA AAATACGGC- ------TAAT CCGCTTATGA

mel.55.2 -GGAAAAT-- ------ATGA AAATACGGC- ------TAAT CCGCTTATGA

**Closely-Related**

**Outgroup Species N**

mau.5 -AGAAAAT-- ------ATGA A---ATGGC- ------TAAT CCGCTTATGA

sec.38 -GGAAAAT-- ------ATGA A---ATGGC- ------TAAT CCGCTTATGA

sim.33 -GGAAAAT-- ------ATGA A---ATGGC- ------TAAT CCGCTTATGA

**Outgroup Species**

yak.25 ----AAAT-- ------ATGA AA---TGGT- ------TAAT CCGCTTATGG

luc.41 -GGAAACGAA gaaaacATGA AA---TGGG- ------TAAT CCGCTTATGG

eug.20 gGGAAAAT-- ------ATGA AA---TGGTa atcggcttat gggtgctaag

fuy.9 ----AAAT-- ------ATGA AA---TGGCc tctgggTAAT CCGCTTATGG

**20 21**

**"Concestor" 1137 GCACAACAAA TTGGTTCACA CACTTCGATC GAAATTACTT GCGATCGCCT**

**"Concestor2" GCACAACAAA TTGGTTCACA CACTTCGATC GAAATTACTT GCGATCGCCT**

mel.00.1 GCACAACAAA TTGGTTCACA CACTTCGATC GAAATTACTT GCGATCGCC**A**

mel.01.58 GCACAACAAA TTGGTTCACA CACTTCGATC GAAATTACTT GCGATCGCC**A**

mel.02.7 GCACAACAAA TTGGTTCACA CACTTCGATC GAAATTACTT GCGATCGCCT

mel.04.1 GCACAACAAA TTGGTTCACA CACTTCGATC GAAATTACTT GCGATCGCC**A**

mel.04.7 GCACAACAAA TTGGTTCACA CACTTCGATC GAAATTACTT GCGATCGCC**A**

mel.05.13 GCACAACAAA TTGGTTCACA CACTTCGATC GAAATTACTT GCGATCGCC**A**

mel.05.16 GCACAACAAA TTGGTTCACA CACTTCGATC GAAATTACTT GCGATCGCC**A**

mel.07.20 GCACAACAAA TTGGTTCACA CACTTCGATC GAAATTACTT GCGATCGCC**A**

mel.14.26 GCACAACAAA TTGGTTCACA CACTTCGATC GAAATTACTT GCGATCGCC**A**

mel.17.66 GCACAACAAA TTGGTTCACA CACTTCGATC GAAATTACTT GCGATCGCC**A**

mel.18Ug.7 GCACAACAAA TTGGTTCACA CACTTCGATC GAAATTACTT **T**CGATCGCCT

mel.19.113 GCACAACAAA TTGGTTCACA CACTTCGATC GAAATTACTT GCGATCGCC**A**

mel.23.74 GCACAACAAA TTGGTTCACA CACTTCGATC GAAATTACTT GCGATCGCC**A**

mel.24.81 GCACAACAAA TTGGTTCACA CACTTCGATC GAAATTACTT GCGATCGCCT

mel.26.32 GCACAACAAA TTGGTTCACA CACTTCGATC GAAATTACTT GCGATCGCCT

mel.34.9 GCACAACAAA TTGGTTCACA CACTTCGATC GAAATTACTT GCGATCGCC**A**

mel.39.37 GCACAACAAA TTGGTTCACA CACTTCGATC GAAATTACTT GCGATCGCC**A**

mel.40Ug.12 GCACAACAAA TTGGTTCACA CACTTCGATC GAAATTACTT GCGATCGCC**A**

mel.45.89 GCACAACAAA TTGGTTCACA CACTTCGATC GAAATTACTT GCGATCGCCT

mel.CanS.41 GCACAACAAA TTGGTTCACA CACTTCGATC GAAATTACTT GCGATCGCC**A**

mel.53.105 GCACAACAAA TTGGTTCACA CACTTCGATC GAAATTACTT GCAATCGCC**A**

mel.54Ug.1 GCACAACAAA TTGGTTCACA CACTTCGATC GAAATTACTT **T**CGATCGCCT

mel.59.3 GCACAACAAA TTGGTTCACA CACTTCGATC GAAATTACTT GCGATCGCCT

mel.yw.50 GCACAACAAA TTGGTTCACA CACTTCGATC GAAATTACTT GCGATCGCC**A**

mel.WI83.25 GCACAACAAA TTGGTTCACA CACTTCGATC GAAATTACTT GCGATCGCC**A**

mel.51.100 GCACAACAAA TTGGTTCACA CACTTCGATC GAAATTACTT GCGATCGCCT

mel.21.2 GCACAACAAA TTGGTTCACA CACTTCGATC GAAATTACTT GCGATCGCC**A**

mel.29.3 GCACAACAAA TTGGTTCACA CACTTCGATC GAAATTACTT **T**CGATCGCCT

mel.64.3 GCACAACAAA TTGGTTCACA CACTTCGATC GAAATTACTT GCGATCGCCT

mel.67.1 GCACAACAAA TTGGTTCACA CACTTCGATC GAAATTACTT GCGATCGCC**A**

mel.55.2 GCACAACAAA TTGGTTCACA CACTTCGATC GAAATTACTT GCGATCGCC**A**

**Closely-Related**

**Outgroup Species**

mau.5 GCACAACAAA ATGTTACACA CACTCCGATC GAAATTACTT GCAATCGCAT

sec.38 GCACAACAAA ctcaaacac- ---------- ---------- ----------

sim.33 GCACAACAAA ATGTTACACA CACTCCGATC GAAATTACTT GTGATCGCAT

**Outgroup Species**

yak.25 TAACAACAAA ATGTT----A CACTTCGATC GAAATCACTT GCGATCGCAT

luc.41 GTAacgaaca gtatacagat ctgaattact aagatcgtaa tttggt---T

eug.20 agttctaaga cctttcgatt tggaacttga gtttactatc aaatttgggg

fuy.9 TTATAACAAT ATTatagaaa tttcgataag atttgaaata agatcttttt

**22 23 24 25**

**"Concestor" 1187 T------TTG ATTGGTTTCA GTGTATTGCT TTAACTAGCA GGTGAACACT**

**"Concestor2" T------TTG ATTGGTTTCA GTGTATTGCT TTAACTAGCA GGTGAACACT**

mel.00.1 T------TTG ATTGGTTTCA **A**TGTATTGCT TTAACT**G**GCA GGTGAACACT

mel.01.58 T------TTG ATTGGTTTCA **A**TGTATTGCT TTAACT**G**GCA GGTGAACACT

mel.02.7 T------TTG ATTG**C**TTTCA GTGTATTGCT TTAACT**G**GCA GGTGAACACT

mel.04.1 T------TTG ATTGGTTTCA **A**TGTATTGCT TTAACT**G**GCA GGTGAACACT

mel.04.7 T------TTG ATTGGTTTCA **A**TGTATTGCT TTAACT**G**GCA GGTGAACACT

mel.05.13 T------TTG ATTGGTTTCA **A**TGTATTGCT TTAACT**G**GCA GGTGAACACT

mel.05.16 T------TTG ATTGGTTTCA **A**TGTATTGCT TTAACT**G**GCA GGTGAACACT

mel.07.20 T------TTG ATTGGTTTCA **A**TGTATTGCT TTAACT**G**GCA GGTGAACACT

mel.14.26 T------TTG ATTGGTTTCA **A**TGTATTGCT TTAACT**G**GCA GGTGAACACT

mel.17.66 T------TTG ATTGGTTTCA **A**TGTATTGCT TTAACT**G**GCA GGTGAACACT

mel.18Ug.7 T------TTG ATTG**C**TTTCA GTGTATTGCT TTAACT**G**GCA GGTGAACACT

mel.19.113 T------TTG ATTGGTTTCA **A**TGTATTGCT TTAACT**G**GCA GGTGAACACT

mel.23.74 T------TTG ATTGGTTTCA **A**TGTATTGCT TTAACT**G**GCA GGTGAACACT

mel.24.81 T------TTG ATTG**C**TTTCA GTGCATTGCT TTAACT**G**GCA GGTGAACACT

mel.26.32 T------TTG ATTG**C**TTTCA GTGTATTGCT TTAACTAGCA GGTGAACACT

mel.34.9 T------TTG ATTGGTTTCA **A**TGTATTGCT TTAACT**G**GCA GGTGAACACT

mel.39.37 T------TTG ATTGGTTTCA **A**TGTATTGCT TTAACT**G**GCA GGTGAACACT

mel.40Ug.12 T------TTG ATTGGTTTCA **A**TGTATTGCT TTAACT**G**GCA GGTGAACACT

mel.45.89 T------TTG ATTG**C**TTTCA GTGTATTGCT TTAACTAGCA GGTGAACACT

mel.CanS.41 T------TTG ATTGGTTTCA **A**TGTATTGCT TTAACT**G**GCA GGTGAACACT

mel.53.105 T------TTG ATTGGTTTCA **A**TGTATTGCT TTAACT**G**GCA GGTGAACACT

mel.54Ug.1 T------TTG ATTG**C**TTTCA GTGTATTGCT TTAACT**G**GCA GGTGAACACT

mel.59.3 T------TTG ATTG**C**TTTCA GTGTATTGCT TTAACT**G**GCA GGTGAACACT

mel.yw.50 T------TTG ATTGGTTTCA **A**TGTATTGCT TTAACT**G**GCA GGTGAACACT

mel.WI83.25 T------TTG ATTGGTTTCA **A**TGTATTGCT TTAACT**G**GCA GGTGAACACT

mel.51.100 T------TTG ATTGCTTTCA GTGTATTGCT TTAACT**G**GCA GGTGAACACT

mel.21.2 T------TTG ATTGGTTTCA **A**TGTATTGCT TTAACT**G**GCA GGTGAACACT

mel.29.3 T------TTG ATTG**C**TTTCA GTGTATTGCT TTAACT**G**GCA GGTGAACACT

mel.64.3 T------TTG ATTGGTTTCA GTGTATTGCT TTAACTAGCA GGTGAACACT

mel.67.1 T------TTG ATTGGTTTCA **A**TGTATTGCT TTAACT**G**GCA GATGAACACT

mel.55.2 T------TTG ATTGGTTTCA **A**TGTATTGCT TTAACT**G**GCA GGTGAACACT

***BstXI* site**

**Closely-Related**

**Outgroup Species * * ***

mau.5 T------TTA ATGTT----- -----TTACT TTAACTAGCA GGTGAACACT

sec.38 ---------- ---------- ---------- ------AGCA GGTAAACTCT

sim.33 T------TTA AGTGGTTTCA GTGTATT-CT TTAACTAGCA GATGAACACT

**Outgroup Species**

yak.25 T------TTA AatggcTTTA GTTTATCGCT TCAACTAGCA GGTGAAAATC

luc.41 T------TTG AAATGTTTTA ATTTATTCCT TTCttaataa aggtaatttt

eug.20 ctgaatgggt ttgtaattta gtgtttatta cttgactgaa tcggatctat

fuy.9 ttgactaTTA AATGGTTTCA ATCCACTGCC TTttgataat ggttaatttg

**26 27**

**"Concestor" 1231 TTGTTT---- --------TT TATCTAACGA TTCTTACTAT TTAATATCCT**

**"Concestor2" TTGTTT---- --------TT TATCTAACGA TTCTTACTAT TTATTATCCT**

mel.00.1 TTGTTT---- --------TT TATCTAACGA TTCTTACTAT TTAATATCCT

mel.01.58 TTGTTT---- --------TT TATCTAACGA TTCTTACTAT TTAATATCCT

mel.02.7 TTGTTT---- --------TT TATCTAACGA TTCTTACTAT TTA**T**TATCCT

mel.04.1 TTGTTT---- --------TT TATCTAACGA TTCTTACTAT TTAATATCCT

mel.04.7 TTGTTT---- --------TT TATCTAACGA TTCTTACTAT TTAATATCCT

mel.05.13 TTGTTT---- --------TT TATCTAACGA TTCTTACTAT TTAATATCCT

mel.05.16 TTGTTT---- --------TT TATCTAACGA TTCTTACTAT TTAATATCCT

mel.07.20 TTGTTT---- --------TT TATCTAACGA TTCTTACTAT TTAATATCCT

mel.14.26 TTGTTT---- --------TT TATCTAACGA TTCTTACTAT TTAATATCCT

mel.17.66 TTGTTT---- --------TT TATCTAACGA TTCTTACTAT TTAATATCCT

mel.18Ug.7 TTGTTT---- --------TT TATCTAACGA TTCTTACTAT TTAATATCCT

mel.19.113 TTGTTT---- --------TT TATCTAACGA TTCTTACTAT TTAATATCCT

mel.23.74 TTGTTT---- --------TT TATCTAACGA TTCTTACTAT TTAATATCCT

mel.24.81 TTGTTT---- --------TT TATCTAACGA TTCTTACTAT TTAATATCCT

mel.26.32 TTGTTT---- --------TT TATCTAACGA TTCTTACTAT TTAATATCCT

mel.34.9 TTGTTT---- --------TT TATCTAACGA TTCTTACTAT TTAATATCCT

mel.39.37 TTGTTT---- --------TT TATCTAACGA TTCTTACTAT TTAATATCCT

mel.40Ug.12 TTGTTT---- --------TT TATCTAACGA TTCTTACTAT TTAATATCCT

mel.45.89 TTGTTT---- --------TT TATCTAACGA TTCTTACTAT TTAATATCCT

mel.CanS.41 TTGTTT---- --------TT TATCTAACGA TTCTTACTAT TTAATATCCT

mel.53.105 TTGTTT---- --------TT TATCTAACGA TTCTTACTAT TTAATATCCT

mel.54Ug.1 TTGTTT---- --------TT TATCTAACGA TTCTTACTAT TTAATATCCT

mel.59.3 TTGTTT---- --------TT TATCTAACGA TTCTTACTAT TTA**T**TATCCT

mel.yw.50 TTGTTT---- --------TT TATCTAACGA TTCTTACTAT TTAATATCCT

mel.WI83.25 TTGTTT---- --------TT TATCTAACGA TTCTTACTAT TTAATATCCT

mel.51.100 TTGTTT---- --------TT TATCTAACGA TTCTTACTAG TTA**T**TATCCT

mel.21.2 TTGTTT---- --------TT TATCTAACGA TTCTTACTAT TTAATATCCT

mel.29.3 TTGTTT---- --------TT TATCTAACGA TTCTTACTAT TTAATATCCT

mel.64.3 TTGTTT---- --------TT TATCTAACGA TTCTTACTAT TTAATATCCT

mel.67.1 TTGTTT---- --------TT TATCTAACGA TTCTTACTAT TTAATATCCT

mel.55.2 TTGTTT---- --------TT TATCTAACGA TTCTTACTAT TTAATATCCT

**Closely-Related**

**Outgroup Species**

mau.5 TTGTTGttac cttactaTTT ACC-TTACTA TTCTTACTAT TTGTTGTCCT

sec.38 TTGTt----- -------TTT ACC-TTAATA TTCTTACTAT TTGTTGTCCT

sim.33 TGGTTG---- --------TT ACC-TTACTA TTCTTACTAT TTGTTGTCCT

**Outgroup Species**

yak.25 GGGTTT---- --------TG AGT-TAACGA TTTGTACCAG TTATTGGCCA

luc.41 tagttgacat ttttagacca atacttcatt ataatattgg tccagaattt

eug.20 agggaaatat taagagttct gccttgaagg ttcctgaaaa tgaatctaaa

fuy.9 gtttaaaatt gtaaatgttc agtttccgat taatttctat cagcccattt

**28 29 30 31**

**"Concestor" 1269 AGTCAATTAA TGTATTTTCC AGTACTTCCA TCGATATCAC AGAGTTCCCA**

**"Concestor2" AGTCAATTAA TGTATTTTCC AGTACTTCTA TCGATATCTC AGAGTTCCCA**

mel.00.1 AGTCAATTAA TGTATTTTCC A**C**TACTTCCA TCGATATCAC AGAGTTCCCA

mel.01.58 AGTCAATTAA TGTATTTTCC A**C**TACTTCCA TCGATATCAC AGAGTTCCCA

mel.02.7 AGTCAATTAA TGTATTTTCC AGTACTTCCA TCGATATC**T**C AGAGTTCCCA

mel.04.1 AGTCAATTAA TGTATTTTCC A**C**TACTTCCA TCGATATCAC AGAGTTCCCA

mel.04.7 AGTCAATTAA TGTATTTTCC A**C**TACTTCCA TCGATATCAC AGAGTTCCCA

mel.05.13 AGTCAATTAA TGTATTTTCC A**C**TACTTCCA TCGATATC**T**C AGAGTTCCCA

mel.05.16 AGTCAATTAA TGTATTTTCC A**C**TACTTCCA TCGATATC**T**C AGAGTTCCCA

mel.07.20 AGTCAATTAA TGTATTTTCC A**C**TACTTCCA TCGATATCAC AGAGTTCCCA

mel.14.26 AGTCAATTAA TGTATTTTCC A**C**TACTTCCA TCGATATCAC AGACTTCCCA

mel.17.66 AGTCAATTAA TGTATTTTCC A**C**TACTTCCA TCGATATCAC AGAGTTCCCA

mel.18Ug.7 AGTCAATTAA TGTATTTTCC A**C**TACTTCCA TCGATATC**T**C AGAGTTCCCA

mel.19.113 AGTCAATTAA TGTATTTTCC A**C**TACTTCCA TCGATATCAC AGAGTTCCCA

mel.23.74 AGTCAATTAA TGTATTTTCC A**C**TACTTCCA TCGATATCAC AGAGTTCCCA

mel.24.81 AGTCAATTAA TGTATTTTCC A**C**TACTTCCA TCGATATCAC AGAGTTTCCA

mel.26.32 AGTCAATTAA TGTATTTTCC A**C**TACTTCCA TCGATATC**T**C AGAGTTCCCA

mel.34.9 AGTCAATTAA TGTATTTTCC A**C**TACTTCCA TCGATATCAC AGAGTTCCCA

mel.39.37 AGTCAATTAA TGTATTTTCC A**C**TACTTCCA TCGATATCAC AGAGTTCCCA

mel.40Ug.12 AGTCAATTAA TGTATTTTCC A**C**TACTTCCA TCGATATC**T**C AGAGTTCCCA

mel.45.89 AGTCAATTAA TGTATTTTCC A**C**TACTTCCA TCGATATC**T**C AGAGTTCCCA

mel.CanS.41 AGTCAATTAA TGTATTTTCC A**C**TACTTCCA TCGATATCAC AGAGTTCCCA

mel.53.105 AGTCAATTAA TGTATTTTCC A**C**TACTTCCA TCGATATCAC AGAGTTCCCA

mel.54Ug.1 AGTCAATTAA TGTATTTTCC AGTACTTC**T**A TCGATATC**T**C AGAGTTCCCA

mel.59.3 AGTCAATTAA T**A**TATTTTCC AGTACTTC**T**A TCGATATC**T**C AGAGTTCCCA

mel.yw.50 AGTCAATTAA TGTATTTTCC A**C**TACTTCCA TCGATATCAC AGAGTTCCCA

mel.WI83.25 AGTCAATTAA TGTATTTTCC A**C**TACTTCCA TCGATATCAC AGAGTTCCCA

mel.51.100 AGTCAATTAA TGTATTTTCC A**C**TACTTCCA TCGATATC**T**C AGAGTTCCCA

mel.21.2 AGTCAATTAA TGTATTTTCC A**C**TACTTCCA TCGATATCAC AGAGTTCCCA

mel.29.3 AGTCAATTAA TGTATTTTCC A**C**TACTTCCA TCGATATCAC AGAGTTCCCA

mel.64.3 AGTCAATTAA TGTATTTTCC AGTACTTCCA TCGATATCAC AGAGTTCCCA

mel.67.1 AGTCAATTAA TGTATTTTCC A**C**TACTTCCA TCGATATCAC AGAGTTCCCA

mel.55.2 AGTCAATTAA TGTATTTTCC A**C**TACTTCCA TCGATATCAC AGAGTTCCCA

**Closely-Related**

**Outgroup Species**

mau.5 AGTCAATTAA TGTATTTTCC AGTACTTCTA TCGATATTCC CAACCCCTAG

sec.38 AGTCAATTAA TGTATTTTCC AGTGCTTTCA TCGATATTCC CAACCCATAC

sim.33 AGTCAATTAA TGTATTTTCC GGTACTTCCA TCGATATTCC CAACCCATAC

**Outgroup Species**

yak.25 AGTCCCCTGA AGTATTTTCC AGTACTTCTC CGttttccga ccatcgacca

luc.41 gtaaagtttc ccaaataaat gaaatatgaa ttatttaaac atttttctgc

eug.20 cttgctgctg taagtttatg caaaatatga agtcaaggga gttctcccta

fuy.9 tataagaaag tttttttttg ctatttgcct ttttgttttt caaattaatt

**"Concestor" 1319 TT-------- ---------- ---------- ---------- ----------**

**"Concestor2" TT-------- ---------- ---------- ---------- ----------**

mel.00.1 TT-------- ---------- ---------- ---------- ----------

mel.01.58 TT-------- ---------- ---------- ---------- ----------

mel.02.7 TT-------- ---------- ---------- ---------- ----------

mel.04.1 TT-------- ---------- ---------- ---------- ----------

mel.04.7 TT-------- ---------- ---------- ---------- ----------

mel.05.13 TT-------- ---------- ---------- ---------- ----------

mel.05.16 TT-------- ---------- ---------- ---------- ----------

mel.07.20 TT-------- ---------- ---------- ---------- ----------

mel.14.26 TT-------- ---------- ---------- ---------- ----------

mel.17.66 TT-------- ---------- ---------- ---------- ----------

mel.18Ug.7 TT-------- ---------- ---------- ---------- ----------

mel.19.113 TT-------- ---------- ---------- ---------- ----------

mel.23.74 TT-------- ---------- ---------- ---------- ----------

mel.24.81 TT-------- ---------- ---------- ---------- ----------

mel.26.32 TT-------- ---------- ---------- ---------- ----------

mel.34.9 TT-------- ---------- ---------- ---------- ----------

mel.39.37 TT-------- ---------- ---------- ---------- ----------

mel.40Ug.12 TT-------- ---------- ---------- ---------- ----------

mel.45.89 TT-------- ---------- ---------- ---------- ----------

mel.CanS.41 TT-------- ---------- ---------- ---------- ----------

mel.53.105 TT-------- ---------- ---------- ---------- ----------

mel.54Ug.1 TT-------- ---------- ---------- ---------- ----------

mel.59.3 TT-------- ---------- ---------- ---------- ----------

mel.yw.50 TT-------- ---------- ---------- ---------- ----------

mel.51.100 TT-------- ---------- ---------- ---------- ----------

mel.21.2 TT-------- ---------- ---------- ---------- ----------

mel.29.3 TT-------- ---------- ---------- ---------- ----------

mel.64.3 TT-------- ---------- ---------- ---------- ----------

mel.67.1 TT-------- ---------- ---------- ---------- ----------

mel.55.2 TT-------- ---------- ---------- ---------- ----------

**Closely-Related**

**Outgroup Species**

mau.5 ---------- ---------- ---------- ---------- ----------

sec.38 ---------- ---------- ---------- ---------- ----------

sim.33 ---------- ---------- ---------- ---------- ----------

**Outgroup Species**

yak.25 TTtcagag-- ---------- ---------- ---------- ----------

luc.41 catccggtat tctataaaat atttaattct tatctgaaat attttagaaa

eug.20 Taagttcttc tcaattagtt catgtctctg taagccttaa agtgaa----

fuy.9 Tctaggatat attaaacatt ctgtattttt aaccattctt tcaataaata

**"Concestor" 1321 ---------- ---------- ---------- ---------- ----------**

**"Concestor2" ---------- ---------- ---------- ---------- ----------**

mel.00.1 ---------- ---------- ---------- ---------- ----------

mel.01.58 ---------- ---------- ---------- ---------- ----------

mel.02.7 ---------- ---------- ---------- ---------- ----------

mel.04.1 ---------- ---------- ---------- ---------- ----------

mel.04.7 ---------- ---------- ---------- ---------- ----------

mel.05.13 ---------- ---------- ---------- ---------- ----------

mel.05.16 ---------- ---------- ---------- ---------- ----------

mel.07.20 ---------- ---------- ---------- ---------- ----------

mel.14.26 ---------- ---------- ---------- ---------- ----------

mel.17.66 ---------- ---------- ---------- ---------- ----------

mel.18Ug.7 ---------- ---------- ---------- ---------- ----------

mel.19.113 ---------- ---------- ---------- ---------- ----------

mel.23.74 ---------- ---------- ---------- ---------- ----------

mel.24.81 ---------- ---------- ---------- ---------- ----------

mel.26.32 ---------- ---------- ---------- ---------- ----------

mel.34.9 ---------- ---------- ---------- ---------- ----------

mel.39.37 ---------- ---------- ---------- ---------- ----------

mel.40Ug.12 ---------- ---------- ---------- ---------- ----------

mel.45.89 ---------- ---------- ---------- ---------- ----------

mel.CanS.41 ---------- ---------- ---------- ---------- ----------

mel.53.105 ---------- ---------- ---------- ---------- ----------

mel.54Ug.1 ---------- ---------- ---------- ---------- ----------

mel.59.3 ---------- ---------- ---------- ---------- ----------

mel.yw.50 ---------- ---------- ---------- ---------- ----------

mel.WI83.25 ---------- ---------- ---------- ---------- ----------

mel.51.100 ---------- ---------- ---------- ---------- ----------

mel.21.2 ---------- ---------- ---------- ---------- ----------

mel.29.3 ---------- ---------- ---------- ---------- ----------

mel.64.3 ---------- ---------- ---------- ---------- ----------

mel.67.1 ---------- ---------- ---------- ---------- ----------

mel.55.2 ---------- ---------- ---------- ---------- ----------

**Closely-Related**

**Outgroup Species**

mau.5 ---------- ---------- ---------- ---------- ----------

sec.38 ---------- ---------- ---------- ---------- ----------

sim.33 ---------- ---------- ---------- ---------- ----------

**Outgroup Species**

yak.25 ---------- ---------- ---------- ---------- ----------

luc.41 ttcaattata aaggtactat tttataaagt taatattcaa cataagataa

eug.20 ---------- ---------- ---------- ---------- ----------

fuy.9 cttttcttta tagactaatt tatttaaaaa acaaaacatg ttgtatataa

**"Concestor" 1321 ---------- ---------- ---------- ---------- ----------**

**"Concestor2" ---------- ---------- ---------- ---------- ----------**

mel.00.1 ---------- ---------- ---------- ---------- ----------

mel.01.58 ---------- ---------- ---------- ---------- ----------

mel.02.7 ---------- ---------- ---------- ---------- ----------

mel.04.1 ---------- ---------- ---------- ---------- ----------

mel.04.7 ---------- ---------- ---------- ---------- ----------

mel.05.13 ---------- ---------- ---------- ---------- ----------

mel.05.16 ---------- ---------- ---------- ---------- ----------

mel.07.20 ---------- ---------- ---------- ---------- ----------

mel.14.26 ---------- ---------- ---------- ---------- ----------

mel.17.66 ---------- ---------- ---------- ---------- ----------

mel.18Ug.7 ---------- ---------- ---------- ---------- ----------

mel.19.113 ---------- ---------- ---------- ---------- ----------

mel.23.74 ---------- ---------- ---------- ---------- ----------

mel.24.81 ---------- ---------- ---------- ---------- ----------

mel.26.32 ---------- ---------- ---------- ---------- ----------

mel.34.9 ---------- ---------- ---------- ---------- ----------

mel.39.37 ---------- ---------- ---------- ---------- ----------

mel.40Ug.12 ---------- ---------- ---------- ---------- ----------

mel.45.89 ---------- ---------- ---------- ---------- ----------

mel.CanS.41 ---------- ---------- ---------- ---------- ----------

mel.53.105 ---------- ---------- ---------- ---------- ----------

mel.54Ug.1 ---------- ---------- ---------- ---------- ----------

mel.59.3 ---------- ---------- ---------- ---------- ----------

mel.yw.50 ---------- ---------- ---------- ---------- ----------

mel.WI83.25 ---------- ---------- ---------- ---------- ----------

mel.51.100 ---------- ---------- ---------- ---------- ----------

mel.21.2 ---------- ---------- ---------- ---------- ----------

mel.29.3 ---------- ---------- ---------- ---------- ----------

mel.64.3 ---------- ---------- ---------- ---------- ----------

mel.67.1 ---------- ---------- ---------- ---------- ----------

mel.55.2 ---------- ---------- ---------- ---------- ----------

**Closely-Related**

**Outgroup Species**

mau.5 ---------- ---------- ---------- ---------- ----------

sec.38 ---------- ---------- ---------- ---------- ----------

sim.33 ---------- ---------- ---------- ---------- ----------

**Outgroup Species**

yak.25 ---------- ---------- ---------- ---------- ----------

luc.41 agtaataaat tgaatttcgt agattcatac tttactataa aatctattta

eug.20 ---------- ---------- ---------- ---------- ----------

fuy.9 aaaacttcaa tttaattttg atattttaat caatacttaa aaaccctgaa

**"Concestor" 1321 ---------- ---------- ---------- ---------- ----------**

**"Concestor2" ---------- ---------- ---------- ---------- ---------**

mel.00.1 ---------- ---------- ---------- ---------- ----------

mel.01.58 ---------- ---------- ---------- ---------- ----------

mel.02.7 ---------- ---------- ---------- ---------- ----------

mel.04.1 ---------- ---------- ---------- ---------- ----------

mel.04.7 ---------- ---------- ---------- ---------- ----------

mel.05.13 ---------- ---------- ---------- ---------- ----------

mel.05.16 ---------- ---------- ---------- ---------- ----------

mel.07.20 ---------- ---------- ---------- ---------- ----------

mel.14.26 ---------- ---------- ---------- ---------- ----------

mel.17.66 ---------- ---------- ---------- ---------- ----------

mel.18Ug.7 ---------- ---------- ---------- ---------- ----------

mel.19.113 ---------- ---------- ---------- ---------- ----------

mel.23.74 ---------- ---------- ---------- ---------- ----------

mel.24.81 ---------- ---------- ---------- ---------- ----------

mel.26.32 ---------- ---------- ---------- ---------- ----------

mel.34.9 ---------- ---------- ---------- ---------- ----------

mel.39.37 ---------- ---------- ---------- ---------- ----------

mel.40Ug.12 ---------- ---------- ---------- ---------- ----------

mel.45.89 ---------- ---------- ---------- ---------- ----------

mel.CanS.41 ---------- ---------- ---------- ---------- ----------

mel.53.105 ---------- ---------- ---------- ---------- ----------

mel.54Ug.1 ---------- ---------- ---------- ---------- ----------

mel.59.3 ---------- ---------- ---------- ---------- ----------

mel.yw.50 ---------- ---------- ---------- ---------- ----------

mel.WI83.25 ---------- ---------- ---------- ---------- ----------

mel.51.100 ---------- ---------- ---------- ---------- ----------

mel.21.2 ---------- ---------- ---------- ---------- ----------

mel.29.3 ---------- ---------- ---------- ---------- ----------

mel.64.3 ---------- ---------- ---------- ---------- ----------

mel.67.1 ---------- ---------- ---------- ---------- ----------

mel.55.2 ---------- ---------- ---------- ---------- ----------

**Closely-Related**

**Outgroup Species**

mau.5 ---------- ---------- ---------- ---------- ----------

sec.38 ---------- ---------- ---------- ---------- ----------

sim.33 ---------- ---------- ---------- ---------- ----------

**Outgroup Species**

yak.25 ---------- ---------- ---------- ---------- ----------

luc.41 tacgacctga aattctttta attttccgaa atacattaaa tattaattaa

eug.20 ---------- ---------- ---------- ---------- --ATATATAT

fuy.9 agcatgtttc aattaatttg taacttagta ctacatgaag tgATGTAAAG

**"Concestor" 1321 ---------- ---------- ---------- ---------- ----------**

**"Concestor2" ---------- ---------- ---------- ---------- ----------**

mel.00.1 ---------- ---------- ---------- ---------- ----------

mel.01.58 ---------- ---------- ---------- ---------- ----------

mel.02.7 ---------- ---------- ---------- ---------- ----------

mel.04.1 ---------- ---------- ---------- ---------- ----------

mel.04.7 ---------- ---------- ---------- ---------- ----------

mel.05.13 ---------- ---------- ---------- ---------- ----------

mel.05.16 ---------- ---------- ---------- ---------- ----------

mel.07.20 ---------- ---------- ---------- ---------- ----------

mel.14.26 ---------- ---------- ---------- ---------- ----------

mel.17.66 ---------- ---------- ---------- ---------- ----------

mel.18Ug.7 ---------- ---------- ---------- ---------- ----------

mel.19.113 ---------- ---------- ---------- ---------- ----------

mel.23.74 ---------- ---------- ---------- ---------- ----------

mel.24.81 ---------- ---------- ---------- ---------- ----------

mel.26.32 ---------- ---------- ---------- ---------- ----------

mel.34.9 ---------- ---------- ---------- ---------- ----------

mel.39.37 ---------- ---------- ---------- ---------- ----------

mel.40Ug.12 ---------- ---------- ---------- ---------- ----------

mel.45.89 ---------- ---------- ---------- ---------- ----------

mel.CanS.41 ---------- ---------- ---------- ---------- ----------

mel.53.105 ---------- ---------- ---------- ---------- ----------

mel.54Ug.1 ---------- ---------- ---------- ---------- ----------

mel.59.3 ---------- ---------- ---------- ---------- ----------

mel.yw.50 ---------- ---------- ---------- ---------- ----------

mel.WI83.25 ---------- ---------- ---------- ---------- ----------

mel.51.100 ---------- ---------- ---------- ---------- ----------

mel.21.2 ---------- ---------- ---------- ---------- ----------

mel.29.3 ---------- ---------- ---------- ---------- ----------

mel.64.3 ---------- ---------- ---------- ---------- ----------

mel.67.1 ---------- ---------- ---------- ---------- ----------

mel.55.2 ---------- ---------- ---------- ---------- ----------

**Closely-Related**

**Outgroup Species**

mau.5 ---------- ---------- ---------- ---------- ----------

sec.38 ---------- ---------- ---------- ---------- ----------

sim.33 ---------- ---------- ---------- ---------- ----------

**Outgroup Species**

yak.25 ---------- ---------- ---------- ---------- ----------

luc.41 tatacctatt atttttcttc acttctgtgt tctattaaat attaaattgt

eug.20 ATCTATCCC- ---------- ---------- ---------- ----------

fuy.9 ATATGTCCG- ---------- ---------- ---------- ----------

**"Concestor" 1321 ---------- ---------- ---------- ---------- ----------**

**"Concestor2" ---------- ---------- ---------- ---------- ----------**

mel.00.1 ---------- ---------- ---------- ---------- ----------

mel.01.58 ---------- ---------- ---------- ---------- ----------

mel.02.7 ---------- ---------- ---------- ---------- ----------

mel.04.1 ---------- ---------- ---------- ---------- ----------

mel.04.7 ---------- ---------- ---------- ---------- ----------

mel.05.13 ---------- ---------- ---------- ---------- ----------

mel.05.16 ---------- ---------- ---------- ---------- ----------

mel.07.20 ---------- ---------- ---------- ---------- ----------

mel.14.26 ---------- ---------- ---------- ---------- ----------

mel.17.66 ---------- ---------- ---------- ---------- ----------

mel.18Ug.7 ---------- ---------- ---------- ---------- ----------

mel.19.113 ---------- ---------- ---------- ---------- ----------

mel.23.74 ---------- ---------- ---------- ---------- ----------

mel.24.81 ---------- ---------- ---------- ---------- ----------

mel.26.32 ---------- ---------- ---------- ---------- ----------

mel.34.9 ---------- ---------- ---------- ---------- ----------

mel.39.37 ---------- ---------- ---------- ---------- ----------

mel.40Ug.12 ---------- ---------- ---------- ---------- ----------

mel.45.89 ---------- ---------- ---------- ---------- ----------

mel.CanS.41 ---------- ---------- ---------- ---------- ----------

mel.53.105 ---------- ---------- ---------- ---------- ----------

mel.54Ug.1 ---------- ---------- ---------- ---------- ----------

mel.59.3 ---------- ---------- ---------- ---------- ----------

mel.yw.50 ---------- ---------- ---------- ---------- ----------

mel.WI83.25 ---------- ---------- ---------- ---------- ----------

mel.51.100 ---------- ---------- ---------- ---------- ----------

mel.21.2 ---------- ---------- ---------- ---------- ----------

mel.29.3 ---------- ---------- ---------- ---------- ----------

mel.64.3 ---------- ---------- ---------- ---------- ----------

mel.67.1 ---------- ---------- ---------- ---------- ----------

mel.55.2 ---------- ---------- ---------- ---------- ----------

**Closely-Related**

**Outgroup Species**

mau.5 ---------- ---------- ---------- ---------- ----------

sec.38 ---------- ---------- ---------- ---------- ----------

sim.33 ---------- ---------- ---------- ---------- ----------

**Outgroup Species**

yak.25 ---------- ---------- ---------- ---------- ----------

luc.41 tgtctaaaac attgaaaaaa ttcaatacgt taggggtaat ttttaataaa

eug.20 ---------- ---------- ---------- ---------- ----------

fuy.9 ---------- ---------- ---------- ---------- ----------

**"Concestor" 1321 ---------- ---------- ---------- ---------- ----------**

**"Concestor2" ---------- ---------- ---------- ---------- ----------**

mel.00.1 ---------- ---------- ---------- ---------- ----------

mel.01.58 ---------- ---------- ---------- ---------- ----------

mel.02.7 ---------- ---------- ---------- ---------- ----------

mel.04.1 ---------- ---------- ---------- ---------- ----------

mel.04.7 ---------- ---------- ---------- ---------- ----------

mel.05.13 ---------- ---------- ---------- ---------- ----------

mel.05.16 ---------- ---------- ---------- ---------- ----------

mel.07.20 ---------- ---------- ---------- ---------- ----------

mel.14.26 ---------- ---------- ---------- ---------- ----------

mel.17.66 ---------- ---------- ---------- ---------- ----------

mel.18Ug.7 ---------- ---------- ---------- ---------- ----------

mel.19.113 ---------- ---------- ---------- ---------- ----------

mel.23.74 ---------- ---------- ---------- ---------- ----------

mel.24.81 ---------- ---------- ---------- ---------- ----------

mel.26.32 ---------- ---------- ---------- ---------- ----------

mel.34.9 ---------- ---------- ---------- ---------- ----------

mel.39.37 ---------- ---------- ---------- ---------- ----------

mel.40Ug.12 ---------- ---------- ---------- ---------- ----------

mel.45.89 ---------- ---------- ---------- ---------- ----------

mel.CanS.41 ---------- ---------- ---------- ---------- ----------

mel.53.105 ---------- ---------- ---------- ---------- ----------

mel.54Ug.1 ---------- ---------- ---------- ---------- ----------

mel.59.3 ---------- ---------- ---------- ---------- ----------

mel.yw.50 ---------- ---------- ---------- ---------- ----------

mel.WI83.25 ---------- ---------- ---------- ---------- ----------

mel.51.100 ---------- ---------- ---------- ---------- ----------

mel.21.2 ---------- ---------- ---------- ---------- ----------

mel.29.3 ---------- ---------- ---------- ---------- ----------

mel.64.3 ---------- ---------- ---------- ---------- ----------

mel.67.1 ---------- ---------- ---------- ---------- ----------

mel.55.2 ---------- ---------- ---------- ---------- ----------

**Closely-Related**

**Outgroup Species**

mau.5 ---------- ---------- ---------- ---------- ----------

sec.38 ---------- ---------- ---------- ---------- ----------

sim.33 ---------- ---------- ---------- ---------- ----------

**Outgroup Species**

yak.25 ---------- ---------- ---------- ---------- ----------

luc.41 ggtaatttaa aataagagtt taagtacatt ttttttaaag cactaaatat

eug.20 ---------- ---------- ---------- ---------- ----------

fuy.9 ---------- ---------- ---------- ---------- ----------

**"Concestor" 1321 ---------- ---------- ---------- ---------- ----------**

**"Concestor2" 1321 ---------- ---------- ---------- ---------- ----------**

mel.00.1 ---------- ---------- ---------- ---------- ----------

mel.01.58 ---------- ---------- ---------- ---------- ----------

mel.02.7 ---------- ---------- ---------- ---------- ----------

mel.04.1 ---------- ---------- ---------- ---------- ----------

mel.04.7 ---------- ---------- ---------- ---------- ----------

mel.05.13 ---------- ---------- ---------- ---------- ----------

mel.05.16 ---------- ---------- ---------- ---------- ----------

mel.07.20 ---------- ---------- ---------- ---------- ----------

mel.14.26 ---------- ---------- ---------- ---------- ----------

mel.17.66 ---------- ---------- ---------- ---------- ----------

mel.18Ug.7 ---------- ---------- ---------- ---------- ----------

mel.19.113 ---------- ---------- ---------- ---------- ----------

mel.23.74 ---------- ---------- ---------- ---------- ----------

mel.24.81 ---------- ---------- ---------- ---------- ----------

mel.26.32 ---------- ---------- ---------- ---------- ----------

mel.34.9 ---------- ---------- ---------- ---------- ----------

mel.39.37 ---------- ---------- ---------- ---------- ----------

mel.40Ug.12 ---------- ---------- ---------- ---------- ----------

mel.45.89 ---------- ---------- ---------- ---------- ----------

mel.CanS.41 ---------- ---------- ---------- ---------- ----------

mel.53.105 ---------- ---------- ---------- ---------- ----------

mel.54Ug.1 ---------- ---------- ---------- ---------- ----------

mel.59.3 ---------- ---------- ---------- ---------- ----------

mel.yw.50 ---------- ---------- ---------- ---------- ----------

mel.WI83.25 ---------- ---------- ---------- ---------- ----------

mel.51.100 ---------- ---------- ---------- ---------- ----------

mel.21.2 ---------- ---------- ---------- ---------- ----------

mel.29.3 ---------- ---------- ---------- ---------- ----------

mel.64.3 ---------- ---------- ---------- ---------- ----------

mel.67.1 ---------- ---------- ---------- ---------- ----------

mel.55.2 ---------- ---------- ---------- ---------- ----------

**Closely-Related**

**Outgroup Species**

mau.5 ---------- ---------- ---------- ---------- ----------

sec.38 ---------- ---------- ---------- ---------- ----------

sim.33 ---------- ---------- ---------- ---------- ----------

**Outgroup Species**

yak.25 ---------- ---------- ---------- ---------- ----------

luc.41 atagttgctt acaaaagttt gtaaaaaatc ctttaatttt cccattaatt

eug.20 ---------- ---------- ---------- ---------- ----------

fuy.9 ---------- ---------- ---------- ---------- ----------

**"Concestor" 1321 ---------- ---------- ---------- ---------- ----------**

**"Concestor2" ---------- ---------- ---------- ---------- ----------**

mel.00.1 ---------- ---------- ---------- ---------- ----------

mel.01.58 ---------- ---------- ---------- ---------- ----------

mel.02.7 ---------- ---------- ---------- ---------- ----------

mel.04.1 ---------- ---------- ---------- ---------- ----------

mel.04.7 ---------- ---------- ---------- ---------- ----------

mel.05.13 ---------- ---------- ---------- ---------- ----------

mel.05.16 ---------- ---------- ---------- ---------- ----------

mel.07.20 ---------- ---------- ---------- ---------- ----------

mel.14.26 ---------- ---------- ---------- ---------- ----------

mel.17.66 ---------- ---------- ---------- ---------- ----------

mel.18Ug.7 ---------- ---------- ---------- ---------- ----------

mel.19.113 ---------- ---------- ---------- ---------- ----------

mel.23.74 ---------- ---------- ---------- ---------- ----------

mel.24.81 ---------- ---------- ---------- ---------- ----------

mel.26.32 ---------- ---------- ---------- ---------- ----------

mel.34.9 ---------- ---------- ---------- ---------- ----------

mel.39.37 ---------- ---------- ---------- ---------- ----------

mel.40Ug.12 ---------- ---------- ---------- ---------- ----------

mel.45.89 ---------- ---------- ---------- ---------- ----------

mel.CanS.41 ---------- ---------- ---------- ---------- ----------

mel.53.105 ---------- ---------- ---------- ---------- ----------

mel.54Ug.1 ---------- ---------- ---------- ---------- ----------

mel.59.3 ---------- ---------- ---------- ---------- ----------

mel.yw.50 ---------- ---------- ---------- ---------- ----------

mel.WI83.25 ---------- ---------- ---------- ---------- ----------

mel.51.100 ---------- ---------- ---------- ---------- ----------

mel.21.2 ---------- ---------- ---------- ---------- ----------

mel.29.3 ---------- ---------- ---------- ---------- ----------

mel.64.3 ---------- ---------- ---------- ---------- ----------

mel.67.1 ---------- ---------- ---------- ---------- ----------

mel.55.2 ---------- ---------- ---------- ---------- ----------

**Closely-Related**

**Outgroup Species**

mau.5 ---------- ---------- ---------- ---------- ----------

sec.38 ---------- ---------- ---------- ---------- ----------

sim.33 ---------- ---------- ---------- ---------- ----------

**Outgroup Species**

yak.25 ---------- ---------- ---------- ---------- ----------

luc.41 cattactttg cagaccttga cctaaaataa atgtatcatc cCATCGATTT

eug.20 ---------- ---------- ---------- ---------- -CATTGATTT

fuy.9 ---------- ---------- ---------- ---------- -CATCGATTT

**"Concestor" 1321 ---------- ---------- ---------- ----TCGCAA AGTCACATAT**

**"Concestor2" ---------- ---------- ---------- ----TCGCAA AGTCACATAT**

mel.00.1 ---------- ---------- ---------- ----TCGCAA AGTCACATAT

mel.01.58 ---------- ---------- ---------- ----TCGCAA AGTCACATAT

mel.02.7 ---------- ---------- ---------- ----TCGCAA AGTCACACAT

mel.04.1 ---------- ---------- ---------- ----TCGCAA AGTCACATAT

mel.04.7 ---------- ---------- ---------- ----TCGCAA AGTCACATAT

mel.05.13 ---------- ---------- ---------- ----TCGCAA AGTCACATAT

mel.05.16 ---------- ---------- ---------- ----TCGCAA AGTCACATAT

mel.07.20 ---------- ---------- ---------- ----TCGCAA AGTCACATAT

mel.14.26 ---------- ---------- ---------- ----TCGCAA AGTCACATAT

mel.17.66 ---------- ---------- ---------- ----TCGCAA AGTCACATAT

mel.18Ug.7 ---------- ---------- ---------- ----TCGCAA AGTCACATAT

mel.19.113 ---------- ---------- ---------- ----TCGCAA AGTCACATAT

mel.23.74 ---------- ---------- ---------- ----TCGCAA AGTCACATAT

mel.24.81 ---------- ---------- ---------- ----TCGCAA AGTCACATAT

mel.26.32 ---------- ---------- ---------- ----TCGCAA AGTCACATAT

mel.34.9 ---------- ---------- ---------- ----TCGCAA AGTCACATAT

mel.39.37 ---------- ---------- ---------- ----TCGCAA AGTCACATAT

mel.40Ug.12 ---------- ---------- ---------- ----TCGCAA AGTCACATAT

mel.45.89 ---------- ---------- ---------- ----TCGCAA AGTCACATAT

mel.CanS.41 ---------- ---------- ---------- ----TCGCAA AGTCACATAT

mel.53.105 ---------- ---------- ---------- ----TCGCAA AGTCACATAT

mel.54Ug.1 ---------- ---------- ---------- ----TCGCAA AGTCACATAT

mel.59.3 ---------- ---------- ---------- ----TCGCAA AGTCACATAT

mel.yw.50 ---------- ---------- ---------- ----TCGCAA AGTCACATAT

mel.WI83.25 ---------- ---------- ---------- ----TCGCAA AGTCACATAT

mel.51.100 ---------- ---------- ---------- ----TCGCAA AGTCACATAT

mel.21.2 ---------- ---------- ---------- ----TCGCAA AGTCACATAT

mel.29.3 ---------- ---------- ---------- ----TCGCAA AGTCACATAT

mel.64.3 ---------- ---------- ---------- ----TCGCAA AGTCACATAT

mel.67.1 ---------- ---------- ---------- ----TCGCAA AGTCACATAT

mel.55.2 ---------- ---------- ---------- ----TCGCAA AGTCACATAT

**Closely-Related**

**Outgroup Species**

mau.5 ---------- ---------- ---------- ----TCGCAA AGTCACATAT

sec.38 ---------- ---------- ---------- ----TCCCAA AGTCACATAT

sim.33 ---------- ---------- ---------- ----TCCCAA AGTCACATAT

**Outgroup Species**

yak.25 ---------- --------TC CCCCTTTCCC AATGTCCCAA TGTCGCATAT

luc.41 TCCAGAGCCC CGTTTttcAG CCCGTTCCCC TCGAGTCCCT TAACCCATGT

eug.20 TCCtctcttc agcccctttg ccacccctga actccctt-- AGCCTCGTGT

fuy.9 TCCAGAAACC CCTTTcatcc CCTTTCCCTT GAC-ACCCCA AGCCTCATGT

**32**

**"Concestor" 1337 TTGTTCTTTT ATAACGTGAA ----CGCGT- ----ACC--- GCGAAGGCCC**

**"Concestor2" TTGTTCTTTT ATAACGTGAA ----CGCGT- ----ACC--- GCGAAGGCCC**

mel.00.1 TTGTTCTTTT ATAAC**A**TGAA ----CGCGT- ----ACC--- GCGAAGGCCC

mel.01.58 TTGTTCTTTT ATAAC**A**TGAA ----CGCGT- ----ACC--- GCGAAGGCCC

mel.02.7 TTGTTCTTTT ATAACGTGAA ----CGCGT- ----ACC--- GCGAAGGCCC

mel.04.1 TTGTTCTTTT ATAAC**A**TGAA ----CGCGT- ----ACC--- GCGAAGGCCC

mel.04.7 TTGTTCTTTT ATAAC**A**TGAA ----CGCGT- ----ACC--- GCGAAGGCCC

mel.05.13 TTGTTCTTTT ATAACGGGAA ----CGCGT- ----ACC--- GCGAAGGCCC

mel.05.16 TTGTTCTTTT ATAACGGGAA ----CGCGT- ----ACC--- GCGAAGGCCC

mel.07.20 TTGTTCTTTT ATAAC**A**TGAA ----CGCGT- ----ACC--- GCGAAGGCCC

mel.14.26 TTGTTCTTTT ATAAC**A**TGAA ----CGCGT- ----ACC--- GCGAAGGCCC

mel.17.66 TTGTTCTTTT ATAAC**A**TGAA ----CGCGT- ----ACC--- GCGAAGGCCC

mel.18Ug.7 TTGTTCTTTT ATAACGTGAA ----CGCGT- ----ACC--- GCGAAGGCCC

mel.19.113 TTGTTCTTTT ATAAC**A**TGAA ----CGCGT- ----ACC--- GCGAAGGCCC

mel.23.74 TTGTTCTTTT ATAAC**A**TGAA ----CGCGT- ----ACC--- GCGAAGGCCC

mel.24.81 TTGTTCTTTT ATAACGTGAA ----CGCGT- ----ACC--- GCGAAGGCCC

mel.26.32 TTGTTCTTTT ATAAC**A**TGAA ----CGCGT- ----ACC--- GCGAAGGCCC

mel.34.9 TTGTTCTTTT ATAAC**A**TGAA ----CGCGT- ----ACC--- GCGAAGGCCC

mel.39.37 TTGTTCTTTT ATAAC**A**TGAA ----CGCGT- ----ACC--- GCGAAGGCCC

mel.40Ug.12 TTGTTCTTTT ATAAC**A**TGAA ----CGCGT- ----ACC--- GCGAAGGCCC

mel.45.89 TTGTTCTTTT ATAACGTGAA ----CGCGT- ----ACC--- GCAAAGGCCC

mel.CanS.41 TTGTTCTTTT ATAAC**A**TGAA ----CGCGT- ----ACC--- GCGAAGGCCC

mel.53.105 TTGTTCTTTT ATAAC**A**TGAA ----CGCGT- ----ACC--- GCGAAGGCCC

mel.54Ug.1 TTGTTCTTTT ATAAC**A**TGAA ----CGCGT- ----ACC--- GCGAAGGCCC

mel.59.3 TTGTTCTTTT ATAACGTGAA ----CGCGT- ----ACC--- GCGAAGGCCC

mel.yw.50 TTGTTCTTTT ATAAC**A**TGAA ----CGCGT- ----ACC--- GCGAAGGCCC

mel.WI83.25 TTGTTCTTTT ATAAC**A**TGAA ----CGCGT- ----ACC--- GCGAAGGCCC

mel.51.100 TTGTTCTTTT ATAAC**A**TGAA ----CGCGT- ----ACC--- GCGAAGGCCC

mel.21.2 TTGTTCTTTT ATAAC**A**TGAA ----CGCGT- ----ACC--- GCGAAGGCCC

mel.29.3 TTGTTCTTTT ATAAC**A**TGAA ----CGCGT- ----ACC--- GCGAAGGCCC

mel.64.3 TTGTTCTTTT ATAACGTGAA ----CGCGT- ----ACC--- GCGAAGGCCC

mel.67.1 TTGTTCTTTT ATAAC**A**TGAA ----CGCGT- ----ACC--- GCGAAGGCCC

mel.55.2 TTGTTCTTTT ATAAC**A**TGAA ----CGCGT- ----ACC--- GCGAAGGCCC

**Closely-Related**

**Outgroup Species**

mau.5 TTGTTCTTTT ATAACGCGAA ----CGCGT- ----ACC--- GCGAAGGTCC

sec.38 TTGTTCTTTT ATAACGTGAA ----CGAGT- ----ACC--- GCGAAGGCCC

sim.33 TTGTTCTTTT ATAACGTGAA ----CGAGT- ----ACCgcg GCGAAGGTCC

**Outgroup Species**

yak.25 TTGTTCTTTT ATAACGCGAA ----CGCGT- ----ACC--- GCGAAGGCCC

luc.41 TTGTTCTTTT ATAACGTCAA ----CGCGTC GCGAACC--- GAGAAGGCCT

eug.20 TTGTTCTTTT ACAACGTCAA ----CGCGcC GCGCACC--- GAGGAGGCCC

fuy.9 TTGTTATTTT ACAACGTCAc acgtCGCGA- ----ACC--- GAGAAGGTCT

**33**

**"Concestor" 1375 CATAAAGTGT TCGTAATAAA --ATATATTG TGCAATAGTT ATAC------**

**"Concestor2" CATAAAGTGT TCGTAATAAA --ATATATTG TGCAATAGTT ATAC------**

mel.00.1 CATAAAGTGT TCG**C**AATAAA --ATATATTG TGCAATAGTT ATAC------

mel.01.58 CATAAAGTGT TCG**C**AATAAA --ATATATTG TGCAATAGTT ATAC------

mel.02.7 CATAAAGTGT TCG**C**AATAAA --ATATATTG TGCAATAGTT ATAC------

mel.04.1 CATAAAGTGT TCG**C**AATAAA --ATATATTG TGCAATAGTT ATAC------

mel.04.7 CATAAAGTGT TCG**C**AATAAA --ATATATTG TGCAATAGTT ATAC------

mel.05.13 CATAAAGTGT TCG**C**AATAAA --ATATATTG TGCAATAGTT ATAC------

mel.05.16 CATAAAGTGT TCG**C**AATAAA --ATATATTG TGCAATAGTT ATAC------

mel.07.20 CATAAAGTGT TCG**C**AATAAA --ATATATTG TGCAATAGTT ATAC------

mel.14.26 CATAAAGTGT TCG**C**AATAAA --ATATATTG TGCAATAGTT ATAC------

mel.17.66 CATAAAGTGT TCG**C**AATAAA --ATATATTG TGCAATAGTT ATAC------

mel.18Ug.7 CATAAAGTGT TCG**C**AATAAA --ATATATTG TGCAATAGTT ATAC------

mel.19.113 CATAAAGTGT TCG**C**AATAAA --ATATATTG TGCAATAGTT ATAC------

mel.23.74 CATAAAGTGT TCG**C**AATAAA --ATATATTG TGCAATAGTT ATAC------

mel.24.81 CATAAAGTGT TCG**C**AATAAA --ATATATTG TGCAATAGTT ATAC------

mel.26.32 CATAGAGTGT TCGTAATAAA --ATATATTG TGCAATAGTT ATAC------

mel.34.9 CATAAAGTGT TCG**C**AATAAA --ATATATTG TGCAATAGTT ATAC------

mel.39.37 CATAAAGTGT TCG**C**AATAAA --ATATATTG TGCAATAGTT ATAC------

mel.40Ug.12 CATAAAGTGT TCGTAATAAA --ATATATTG TGCAATAGTT ATAC------

mel.45.89 CATAAAGTGT TCGTAATAAA --ATATATTG TGCAATAGTT ATAC------

mel.CanS.41 CATAAAGTGT TCG**C**AATAAA --ATATATTG TGCAATAGTT ATAC------

mel.53.105 CATAAAGTGT TCG**C**AATAAA --ATATATTG TGCAATAGTT ATAC------

mel.54Ug.1 CATAAAGTGT TCG**C**AATAAA --ATATATTG TGCAATAGTT ATAC------

mel.59.3 CATAAAGTGT TCG**C**AATAAA --ATATATTG TGCAATAGTT ATAC------

mel.yw.50 CATAAAGTGT TCG**C**AATAAA --ATATATTG TGCAATAGTT ATAC------

mel.WI83.25 CATAAAGTGT TCG**C**AATAAA --ATATATTG TGCAATAGTT ATAC------

mel.51.100 CATAAAGTGT TCG**C**AATAAA --ATATATTG TGCAATAGTT ATAC------

mel.21.2 CATAAAGTGT TCG**C**AATAAA --ATATATTG TGCAATAGTT ATAC------

mel.29.3 CATAAAGTGT TCG**C**AATAAA --ATATATTG TGCAATAGTT ATAC------

mel.64.3 CATAAAGTGT TCGTAATAAA --ATATATTG TGCAATAGTT ATAC------

mel.67.1 CATAAAGTGT TCG**C**AATAAA --ATATATTG TGCAATAGTT ATAC------

mel.55.2 CATAAAGTGT TCG**C**AATAAA --ATATATTG TGCAATAGTT ATAC------

**Closely-Related**

**Outgroup Species**

mau.5 CATAAAGTGT TCGTAATAAA --ATATATTG TGCAATATTT GTGCTATAGT

sec.38 CATAAAGTGT TCGTAATAAA --ATATATTG TGCAATATTT GTGCTATAGT

sim.33 CATAAAGTGT TCGTAATAAA --ATATATTG TGCAATATTT GTGCTATAGT

**Outgroup Species**

yak.25 CATAAAGTGT TCGTAATAAA --ATATATTG TGCAATATTT GTGCTATAGT

luc.41 CATAAAGTGT TCCTAATAAA atATATATTG TGCAATATTT T-GCTATAGT

eug.20 CATAAAGTGT TCGCAATAAA --ATATATTG TGCAATATTT A-GCTATAGT

fuy.9 CATAAAGTGT TCGTAATAAA --ATATATTG TACAATATTT G-GCTATAGT

**34 35 3637**

**"Concestor" 1417 -----AGCCA CTCATATACA TTATATACAA TATATATAT- -------GTG**

**"Concestor2" -----AGCCA CTCATATACA TTATATACAA TATATATAT- -------GTG**

mel.00.1 -----AGCCA CTCATATACA TTATATACAA TATATATAT- -------**A**TG

mel.01.58 -----AGCCA CTCATATACA TTATATACAA TATATATAT- -------**A**TG

mel.02.7 -----AGCCA CTCATATACA TTATATACAA TATATATAT- -------GTG

mel.04.1 -----AGCCA CTCATATACA TTATATACAA TATATATAT- -------**A**TG

mel.04.7 -----AGCCA CTCATATACA TTATATACAA TATATATAT- -------**A**TG

mel.05.13 -----AGCCA CTCATATACA TTATATACAA TATATATAT- -------**A**TG

mel.05.16 -----AGCCA CTCATATACA TTATATACAA TATATATAT- -------**A**TG

mel.07.20 -----AGCCA CTCATATACA TTATATACAA TATATATAT- -------**A**TG

mel.14.26 -----AGCCA CTCATATACA TTATATACAA TATATATAT- -------**A**TG

mel.17.66 -----AGCCA CTCATATACA TTATATACAA TATATATAT- -------**A**TG

mel.18Ug.7 -----AGCCA CTCATATACA TTATATACAA TATATATAT- -------GTG

mel.19.113 -----AGCCA CTCATATACA TTATATACAA TATATATAT- -------**A**TG

mel.23.74 -----AGCCA CTCATATACA TTATATACAA TATATATAT- -------**A**TG

mel.24.81 -----AGCCA CTCATATACA TTATATACAA TATATATAT- -------GTG

mel.26.32 -----AGCCA CTCATATACA TTATATACAA TATATATgta tatggatGTA

mel.34.9 -----AGCCA CTCATATACA TTATATACAA TATATATAT- -------**A**TG

mel.39.37 -----AGCCA CTCATATACA TTATATACAA TATATATAT- -------**A**TG

mel.40Ug.12 -----AGCCA CTCATATACA TTATATACAA TATATATAT- -------GTG

mel.45.89 -----AGCCA CTCATATACA TTATATACAA TATATATAT- -------GTG

mel.CanS.41 -----AGCCA CTCATATACA TTATATACAA TATATATAT- -------**A**TG

mel.53.105 -----AGCCA CTCATATACA TTATATACAA TATATATAT- -------**A**TG

mel.54Ug.1 -----AGCCA CTCATATACA TTATATACAA TATATATAT- -------**A**TG

mel.59.3 -----AGCCA CTCATATACA TTATATACAA TATATATAT- -------**A**TG

mel.yw.50 -----AGCCA CTCATATACA TTATATACAA TATATATAT- -------**A**TG

mel.WI83.25 -----AGCCA CTCATATACA TTATATACAA TATATATAT- -------**A**TG

mel.51.100 -----AGCCA CTCATATACA ATATATACAA TATATATAT- -------**A**TG

mel.21.2 -----AGCCA CTCATATACA TTATATACAA TATATATAT- -------**A**TG

mel.29.3 -----AGCCA CTCATATACA TTATATACAA TATATATAT- -------GTG

mel.64.3 -----AGCCA CTCATATACA TTATATACAA TATATATAT- -------GTG

mel.67.1 -----AGCCA CTCATATACA TTATATACAA TATATATAT- -------**A**TG

mel.55.2 -----AGCCA CTCATATACA TTATATACAA TATATATAT- -------**A**TG

**Closely-Related**

**Outgroup Species**

mau.5 TATACAGCCA CTCATATACA TTATATATAC ATAT------ ------GTGT

sec.38 TATACAGCCA CTC------- --ATATACGT TATATATATA TGCAtgtGTG

sim.33 TATACAGCCA CTC------- --ATATACAT TATATATATA TGCA----TG

**Outgroup Species**

yak.25 TATACAGCCG CTC------- --GTATACAT TATATATA-- ----------

luc.41 TATATAGCCA CTCATATACA TTATATATAT Gtgcccaact atatatacac

eug.20 TATATAGCCA CTCATATACA TTATATACAT Gcg------- ----------

fuy.9 TATATAGCCA CTCATATACA TTATATATAT ---------- ----------

**38 39**

**"Concestor" 1454 TGGATGTGTA TGTGCACAAC TATATAGATG TGTTG-TATA TAAATTG---**

**"Concestor2" TGGATGTGTA TGTGCACAAC TATATAGATG TGTTG-TATA TAAATTG---**

mel.00.1 TGGATGTGTA TGTGCACAAC **C**ATATAGATG TGTTG-TATA TAAATTG---

mel.01.58 TGGATGTGTA TGTGCACAAC **C**ATATAGATG TGTTG-TATA TAAATTG---

mel.02.7 TGGATGTGTA TGTGCACAAC TATATAGATG TGTTG-TATA TAAATTG---

mel.04.1 TGGATGTGTA TGTGCACAAC **C**ATATAGATG TGTTG-TATA TAAATTG---

mel.04.7 TGGATGTGTA TGTGCACAAC **C**ATATAGATG TGTTG-TATA TAAATTG---

mel.05.13 TGGATGTGTA TGTGCACAAC **C**ATATAGATG TGTTG-TATA TAAATTG---

mel.05.16 TGGATGTGTA TGTGCACAAC **C**ATATAGATG TGTTG-TATA TAAATTG---

mel.07.20 TGGATGTGTA TGTGCACAAC **C**ATATAGATG TGTTG-TATA TAAATTG---

mel.14.26 TGGATGTGTA TGTGCACAAC **C**ATATAGATG TGTTG-TATA TAAATTG---

mel.17.66 TGGATGTGTA TGTGCACAAC **C**ATATAGATG TGTTG-TATA TAAATTG---

mel.18Ug.7 TGGATGTGTA TGTGCACAAC TATATAGATG TGTTG-TATA TAAATTG---

mel.19.113 TGGATGTGTA TGTGCACAAC **C**ATATAGATG TGTTG-TATA TAAATTG---

mel.23.74 TGGATGTGTA TGTGCACAAC **C**ATATAGATG TGTTG-TATA TAAATTG---

mel.24.81 TGGATGTGTA TGTGCACAAC TATATAGATG TGTTG-TATA TAAATTG---

mel.26.32 TGGATGTGTA TGTGCACAAC **C**ATATAGATG TGTTG-TATA TAAATTG---

mel.34.9 TGGATGTGTA TGTGCACAAC **C**ATATAGATG TGTTG-TATA TAAATTG---

mel.39.37 TGGATGTGTA TGTGCACAAC **C**ATATAGATG TGTTG-TATA TAAATTG---

mel.40Ug.12 TGGATGTGTA TGTGCACAAC TATATAGATG TGTTG-TATA TAAATTG---

mel.45.89 TGGATGTGTA TGTGCACAAC TATATAAC-- TGTTG-TATA TAAATTG---

mel.CanS.41 TGGATGTGTA TGTGCACAAC **C**ATATAGATG TGTTG-TATA TAAATTG---

mel.53.105 TGGATGTGTA TGTGCACAAC **C**ATATAGATG TGTTG-TATA TAAATTG---

mel.54Ug.1 TGGATGTGTA TGTGCACAAC **C**ATATAGATG TGTTG-TATA TAAATTG---

mel.59.3 TGGATGTGTA TGTGCACAAC **C**ATATAGATG TGTTG-TATA TAAATTG---

mel.yw.50 TGGATGTGTA TGTGCACAAC **C**ATATAGATG TGTTG-TATA TAAATTG---

mel.WI83.25 TGGATGTGTA TGTGCACAAC **C**ATATAGATG TGTTG-TATA TAAATTG---

mel.51.100 TGGATGTGTA TGTGCACAAC **C**ATATAGATG TGTTG-TATA TAAATTG---

mel.21.2 TGGATGTGTA TGTGCACAAC **C**ATATAGATG TGTTG-TATA TAAATTG---

mel.29.3 TGGATGTGTA TGTGCACAAC TATATAGATG TGTTG-TATA TAAATTG---

mel.64.3 TGGATGTGTA TGTGCACAAC TATATAGATG TGTTG-TATA TAAATTG---

mel.67.1 TGGATGTGTA TGTGCACAAC **C**ATATAGATG TGTTG-TATA TAAATTG---

mel.55.2 TGGATGTGTA TGTGCACAAC **C**ATATAGATG TGTTG-TATA TAAATTG---

**Closely-Related**

**Outgroup Species**

mau.5 ---------- ---------- TATATAGATG TGTTG-TATA TAAATTG---

sec.38 TGTGTGTGT- -------AAC TATATAGATG TGTTG-TATA TAAATTG---

sim.33 TGTGTGTGT- -------AAC TATATAGATG TGTTG-TATA TAAATTG---

**Outgroup Species**

yak.25 --------TA TGTGCGCAAC TATATAGATG TGTATATAGA TATAaattgc

luc.41 ---------- ---------- ---------- ---------A TATATTG---

eug.20 ---------- -----GCAAC TATATAGATG TATATATATA TATATTG---

fuy.9 ---------A TGAGCGCAAC TATATAGATG TGTATATATA gaaggctgtc

**40**

**"Concestor" 1500 ---CCATCCC ATTGCTTATC -ATCGCCTTT ATAGGTAGAA TGTAATTTGT**

**"Concestor2" ---CCATCCC ATTGCTTATC -ATCGCCTTT ATAGGTAGAA TGTAATTTGT**

mel.00.1 ---CCATCCC ATTGCTTATC -ATCGCCTTT ATAGGTAGAA TGTAATTTGT

mel.01.58 ---CCATCCC ATTGCTTATC -ATCGCCTTT ATAGGTAGAA TGTAATTTCT

mel.02.7 ---CCATCCC ATTGCTTATC -ATCGCCTTT ATAGGTAGAA TGTAATTTGT

mel.04.1 ---CCATCCC ATTGCTTATC -ATCGCCTTT ATAGGTAGAA TGTAATTTCT

mel.04.7 ---CCATCCC ATTGCTTATC -ATCGCCTTT ATAGGTAGAA TGTAACTTCT

mel.05.13 ---CCATCCC ATTGCTTATC -ATCGCCTTT ATAGGTAGAA TGTAATTTGT

mel.05.16 ---CCATCCC ATTGCTTATC -ATCGCCTTT ATAGGTAGAA TGTAATTTCT

mel.07.20 ---CCATCCC ATTGCTTATC -ATCGCCTTT ATAGGTAGAA TGTAGTTTGG

mel.14.26 ---CCATCCC ATTGCTTATC -ATCGCCTTT ATAGGTAGAA TGTAATTTCG

mel.17.66 ---CCATCCC ATTGCTTATC -ATCGCCTTT ATAGGTAGAA TGTAATTTCG

mel.18Ug.7 ---CCATCCC ATTGCTTATC -ATCGCCTTT ATAGGTAGAA TGTAATTTGG

mel.19.113 ---CCATCCC ATTGCTTATC -ATCGCCTTT ATAGGTAGAA TGTAATTTCG

mel.23.74 ---CCATCCC ATTGCTTATC -ATCGCCTTT ATAGGTAGAA TGTAATTTCT

mel.24.81 ---CCATCCC ATTGCTTATC -ATCGCCTTT ATAGGTAGAA TGTAATTTCT

mel.26.32 ---TCATCCC ATTGCTTATC -ATCGCCTTT ATAGGTAGAA TGTAATTTGG

mel.34.9 ---CCATCCC ATTGCTTATC -ATCGCCTTT ATAGGTAGAA TGTAATTTCG

mel.39.37 ---CCATCCC ATTGCTTATC -ATCGCCTTT ATAGGTAGAA TGTAATTTCT

mel.40Ug.12 ---CCATCCC ATTGCTTATC -ATCGCCTTT ATAGGTAGAA TGTAATTTCT

mel.45.89 ---CCATCCC AT----TATC -ATCGCCTTT ATAGGTAGAA TGTAATTTCT

mel.CanS.41 ---CCATCCC ATTGCTTATC -ATCGCCTTT ATAGGTAGAA TGTAATTTGT

mel.53.105 ---CCATCCC ATTGCTTATC -ATCGCCTTT ATAGGTAGAA TGTAATTTCT

mel.54Ug.1 ---CCATCCC ATTGCTTATC -ATCGCCTTT ATAGGTAGAA TGTAATTTCT

mel.59.3 ---CCATCCC ATTGCTTATC -ATCGCCTTT ATAGGTAGAA TGTAATTTCG

mel.yw.50 ---CCATCCC ATTGCTTATC -ATCGCCTTT ATAGGTAGAA TGTAATTTGG

mel.WI83.25 ---CCATCCC ATTGCTTATC -ATCGCCTTT ATAGGTAGAA TGTAATTTCT

mel.51.100 ---CCATCCC ATTGCTTATC –ATCGCCTTT ATAGGTAGAA TGTAATTTCG

mel.21.2 ---CCATCCC ATTGCTTATC –ATCGCCTTT ATAGGTAGAA TGTAATTTCG

mel.29.3 ---CCATCCC ATTGCTTATC –ATCGCCTTT ATAGGTAGAA TGTAATTTCG

mel.64.3 ---CCATCCC ATTGCTTATC –ATCGCCTTT ATAGGTAGAA TGTAATTTCG

mel.67.1 ---CCATCCC ATTGCTTATC –ATCGCCTTT ATAGGTAGAA TGTAATTTCG

mel.55.2 ---CCATCCC ATTGCTTATC –ATCGCCTTT ATAGGTAGAA TGTAATTTCG

**Closely-Related**

**Outgroup Species**

mau.5 ---CCATCCC ATTGCTTATC -ACCGCCTTT ATAGGTAGAA TGTAATTTCG

sec.38 ---CCATCCC ATTGCTTATC -ATCGCCTTT ATAGGTAGAA TGTAATTTCG

sim.33 ---CCATCCC ATTGCTTATC -ATCGCCTTT ATAGGTAGAA TGTAATTTGG

**Outgroup Species**

yak.25 catCCATCCC AT----TGCT TATCGCCTTT ATAGGTAGAA TGTAATTTGT

luc.41 ---CCATCCC AT----TGCT TATCGCCTTT ATAGGTAGAA TGTAATTTGT

eug.20 ---CCAGCCC AT----TGCT TATCGCCTTT ATAGGTAGAA TGTAATTTGT

fuy.9 ccac------ ------TGCT TATCGCCTTT ATAGGTAGAA TGTAATTTCT

**SbfI**

**"Concestor" 1546 TTTTATGTGC CGTTTTGcct gcagg**

**“Concestor2" TTTTATGTGC CGTTTTGcct gcagg**

mel.00.1 TTTTATGTGC CGTTTTGCCT GCAGG

mel.01.58 TTTTATGCGC CGTTTTGCCT GCAGG

mel.02.7 TTTTATGTGC CGTTTTGCCT GCAGG

mel.04.1 TTTTATGTGC AGTTTTGCCT GCAGG

mel.04.7 TTTTATGCGC CGTTTTGCCT GCAGG

mel.05.13 TTTTATGTGC CGTTTTGCCT GCAGG

mel.05.16 TTTTATGCGC AGTTTTGCCT GCAGG

mel.07.20 TTTTATGCGC CGTTTTGCCT GCAGG

mel.14.26 TTTTATGCGC AGTTTTGCCT GCAGG

mel.17.66 TTATATGTGC AGTTTTGCCT GCAGG

mel.18Ug.7 TTTTATGTGC CGTTTTGCCT GCAGG

mel.19.113 TTTTATGCGC CGTTTTGCCT GCAGG

mel.23.74 TTTTATGCGC CGTTTTGCCT GCAGG

mel.24.81 TCTTATGCGC CGTTTTGCCT GCAGG

mel.26.32 TTTTATGTGC AGTTTTGCCT GCAGG

mel.34.9 TTTTATGTGC CGTTTTGCCT GCAGG

mel.39.37 TTTTATGCGC AGTTTTGCCT GCAGG

mel.40Ug.12 TTTTATGCGC AGTTTTGCCT GCAGG

mel.45.89 TTTTATGCGC AGTTTTGCCT GCAGG

mel.CanS.41 TTTTATGCGC CGTTTTGCCT GCAGG

mel.53.105 TTTTATGTGC CGTTTTGCCT GCAGG

mel.54Ug.1 TTTTATGCGC CGTTTTGCCT GCAGG

mel.59.3 TTTTATGCGC CGTTTTGCCT GCAGG

mel.yw.50 TTTTATGTGC CGTTTTGCCT GCAGG

mel.WI83.25 TTTTATGTGC CGTTTTGCCT GCAGG

mel.51.100 TTTTATGCGC AGTTTTGCCT GCAGG

mel.21.2 TTTTATGCGC AGTTTTGCCT GCAGG

mel.29.3 TTTTATGCGC AGTTTTGCCT GCAGG

mel.64.3 TTTTATGCGC AGTTTTGCCT GCAGG

mel.67.1 TTTTATGCGC AGTTTTGCCT GCAGG

mel.55.2 TTTTATGCGC AGTTTTGCCT GCAGG

**Closely-Related**

**Outgroup Species**

mau.5 TTTTATGCGC CGTTTTGCCT GCAGG

sec.38 TTTTATGCGC CGTTTTGCCT GCAGG

sim.33 TTTTATGCGC CGTTTTGCCT GCAGG

**Outgroup Species**

yak.25 TTTTATGTGC CGTTTTGCCT GCAGG

luc.41 TTTTATGCGC CGTTTTGCCT GCAGG

eug.20 TTTTATGCGC AGTTTTGCCT GCAGG

fuy.9 TTTTATGTGC AGTTTTGCCT GCAGG

**B.**

**AscI**

D. mel Light 1 1 --GGCGCGCC CACATAAAAA TCAGCAACAA AGTTGCTCTG GCCCCATAAA

D. yak 1 --GGCGCGCC CACATAAAAA TCAGCAACAA AGTTGCCCTG GCCCCATAAA

D. fuy 1 --GGCGCGCC CACATAAAAA TCAGCAACAA AGTTGCCCTG GCCCCATAAA

D. aur 1 --GGCGCGCC CACATAAAAA TCAGCAACAA AGTTGCCCTG GCCCCATAAA

D. mel Light 1 49 AGATTGCAAA CAAAAAC--A GAACAACAGA ATGGCATGGA ATAAAATTTA

D. yak 51 AAATTGCAAA CAAAAAC--A GAACAACGGA ATGGCATGGA ATAAAATTTA

D. fuy 49 AAATTGCAAA CAAAAAgaga acaac----A ATGGCATGGA ATAAAATTTA

D. aur 49 AAATTGCTAA CAAAAAaggA GAACAACAGA ATGGCATGGA ATAAAATTTA

D. mel Light 1 97 TATGAATAAC AAAAAGCAGC TAAAgca--- ---AGCAGCA ACAACAATAG

D. yak 99 TATGAATAAC AAAAAGCAaa agcagctaca gcaAGCGGCA ACAACAACAG

D. fuy 95 TATGAATAAC AAAAAGCAGC TAAAagaaac ---AGCAGCA ACAACAACAG

D. aur 99 TATGAATAAC AAAAgcagca gtagt----- ------AGCA ACAACAACAG

D. mel Light 1 141 TTTACTGCCC CGGCTCAGCG GTACACTGTG CAAAACGTTG tactcctcct

D. yak 149 TTTACTGCCC CGGCTTAGTG GTACACTGTA CGAAATAAaa taactccctc

D. fuy 142 TTTACTGCTC TGGCTCAGCA GTACACTGTG GAAAATATTG ataccattct

D. aur 138 TTTACGGCCC TGGCTCAACA GTACACAGAG AGAAAaaata ttcacgactt

D. mel Light 1 191 cat------- ---------- ---------- ---------- ----------

D. yak 199 tcattaaata aaagtaaact aaatcacacg caagctttgt aaataatcgg

D. fuy 192 tttttatatc cataataaag gccaatagag tatttttact gcatgatagt

D. aur 188 ttcttagaca aaattatatt agtttgatgt agaaaaattt ttggtgttta

D. mel Light 1 194 ---------- ---------- ---------- ---------- ----------

D. yak 249 tactacatcc tagtatagtt tatttctatt aatatttttt ttacaattta

D. fuy 242 atttgggagc tcataatttg taaactgaaa acaagtttgc tttggttctt

D. aur 238 atatctttgt atatatatgg tttttatttt gtaagaaagt ggtttttaaa

D. mel Light 1 194 ---------- ---------- ---------- ---------- ----------

D. yak 299 tgtgcccaac aaagatgaa- ---------- ---------- ----------

D. fuy 292 tagggaagaa aaaaaggagc ttttaaattt aaaatatcat tgccattaga

D. aur 288 gacgcaataa atcttaagtc ccttaaatat aaataaaata ttattcaata

D. mel Light 1 194 ---------- ---------- ---------- ---------- ----------

D. yak 318 ---------- ---------- ---------- ---------- ----------

D. fuy 342 acaggaaaaa ctacttaata tttgttaagc cttaaataaa ataaatacaa

D. aur 338 tagtatttaa atgttaatac atatttttta tattatttta attactaatt

D. mel Light 1 194 ---------- ---------- ---------- ---------- ----------

D. yak 318 ---------- ---------- ---------- ---------- ----------

D. fuy 392 atttattcca atgcaaaaat acatgttttt ttattcaaaa aaaggcttaa

D. aur 388 tgtgttttac ttttcacaga ggcagtcaga aaagggctgc cttttaggca

D. mel Light 1 194 ---------- ---------A ATAATATGAG TATATAGAGT ATATAATAta

D. yak 318 ---------- ---------- ---------- ---------- ----------

D. fuy 442 ctaaactttc tgaacgtgaA ACAATATTAC TAACTAGGGT ATGTACTAaa

D. aur 438 aactttgatt aacattttaa ggtatttcag gaatcttttt aacaagataa

D. mel Light 1 225 ctatatatct ccattgataa tttcgatcat tttcaccttt taactaattt

D. yak 318 ---------- ---------- ---------- ---------- ----------

D. fuy 492 tataatttgt ataaaatctg gccaaaagca atgcaaattt tttgtagtgt

D. aur 488 aataatatgc taagaa---- ---------- ---------- ----------

D. mel Light 1 275 atgcccaatg tagttgcATT TCTCTGAGTG TGCAGTAAGT GCCCCAGAAT

D. yak 318 ---------- -------ATT TCTCTAAGTG TGCAGTAag- ----------

D. fuy 542 a--------- ---------- ---------- --CAGTAAGT GCCCAAGAAT

D. aur 504 ---------- ---------T TCTTTCAGTG CCAAGTAAGT GCCcgg----

D. mel Light 1 325 GCGAATGCAT CTCGGGTTCA TCG--GCGGG TCGAGTTTGT TGCAACAacc

D. yak 340 -----TGCAT CTCGGGTTCA TCG--Ggttc ---AGTTTGT TGCAA--CAC

D. fuy 561 GCGAATGCAT CTCGGGTTCA ACG--GCGGG TCGAGTTTGT TGCAT--CAC

D. aur 531 --GAATGCAT CTCGGGTTCA TCGagGCAGG TCGAGTTTGT TGCAACACAC

D. mel Light 1 373 gaagaaCGAA GAAGTTGCAG CGTGCGTTCG GCATTAAAAT TGTGTTTATG

D. yak 378 C-----CGAA GAAGTTGCAG CGTGCGTTCG GCATTAAAAT TGTGTTTATG

D. fuy 607 C-----CGAA GAACTTGCAG CGTGCGTCCG GCATTAAAAT TGTGTTTATG

D. aur 579 C-----CGAA GAAGTTGCAG CATGCGTCCG GCATTAAAAT TGTGTTTATG

D. mel Light 1 423 CGTGTTCGGT AATTTTATAA AAGTTAAATT AGTTTTAAGA CCCTAAATTC

D. yak 423 CGTGTTCGGT AATTTTATAA AAGTTAAATT AGTTTTAAGA CCATAAATTC

D. fuy 652 CGTGTTTGGT AATTTTATAA AAGTTAAATT AGTTTTAAGA CCATAAATTC

D. aur 624 CGTGTTTGGT AATTTTATAA AAGTTAAATT AGTTTTAAGA CCATAAATTC

D. mel Light 1 473 AGCTCACTCT CTCTCTCtcg --CTCTTTC- --------TC TTTGCCATTT

D. yak 473 AGCTCACTCT CTCCCTCggc ctCTCTGTC- --------TC TTTGCCATTT

D. fuy 702 AGCGCACTCT CTggcat--- ---------- ------AGTC TCTGCCATTT

D. aur 674 AGCGCACTCT CGCTggcgca gttccccatg gcccgaAGTC TCTGCCATTT

D. mel Light 1 512 TAACTTTTAT TACTCTTAAT ATAAA----- AAAGCTGGCT ----AGATGC

D. yak 514 TAACTTTTAT TACTCTTAAT ATAAA----- AAAGCTGGCT ggCTAGAAGC

D. fuy 733 TAACTTTTAT TACTTTTAAT ATAAA----- AAAGCTGG-- --ATAGAAGC

D. aur 724 TAACTTTTAT TACTTTTAAT ATAAAGAAAA AAAGGTGG-- --CTAGGAGC

**Abd-B 1 Abd-B 2**

D. mel Light 1 553 GGGCCAGCTG TAAAAAT--- GCACGCGGTC ATAAAAAGTT GCAGGAGG--

D. yak 559 GGGCCAGCTG TAAAAAT--- GCATGCGCTC ATAAAAAGTT GCAGGAGGCA

D. fuy 774 GGGCCAGCTG TAAAAAT--- GCACGCGGTC ATAAAAAGTT GCAGGAGGCA

D. aur 770 AGGCCAGCTG TAGTAAAAAT GCACGCGGTC ATAAAAAGTT GCAGGaggca

**Abd-B 3 Abd-B 4**

D. mel Light 1 598 ---------- ---CATGTTG CCAGTTGCCT GCAACCGGCA acattcgCAG

D. yak 606 TGTTGCc--- ---------- --AGTTGCCA GTTGCCTGCA A------CAG

D. fuy 821 TGTTGCtggt agcCAAGTTG CCAGTTGCCG GTTGCCTGCA A------CAt

D. aur 820 tctacatcga cgtccacatc cacatcgcca tcgggctgga gtccccggga

D. mel Light 1 635 -------AAC A---GCAGCA ACATCGTAAA ATAACTTCTT GCTCTGCGGT

D. yak 635 -------AAC A---GCAGCA ACATCGTAAA ATAACTTCTT GCTCTGCGGT

D. fuy 865 ccactgaAAC G---GCAGCA ACATCGTAAA ATAATTTCTT GCTCTGCGGT

D. aur 870 tcggttggta tgttGCAGCA ACATCGTAAA ATAATTTCTT GCTCTGCGGT

**Abd-B 5**

D. mel Light 1 675 CTGAGTTTGG CCGCAACAAT GTTGCTGCAT TTATTCGTAT TATTATTACA

D. yak 675 CTCCGTTTGG CCGCAACAAT GTTGCCGCAT TTATTCGTAT TATTATTACA

D. fuy 912 CTCCATTTGG CCGCAACAAT GTTGCTGCAT TTATTCGTAT TATTATTACA

D. aur 920 CTCCGTTTGG CCGCAACAAT GTTGCCGCAT TTATTCGTAT TATTATTACA

**Dsx1 Site Abd-B 6**

D. mel Light 1 725 TTTTAATGAA TAATTCTAAT TATATGCAAC TTGAATAAGC CCGC------

D. yak 725 TTTTAATGAA TAATTCTAAT TATATGCAAC TTGAATaggg ccgctgccgc

D. fuy 962 TTTTAATGAT TAATTCTAAT TATATGCGAC TTGAATAAGG CCGCTGAC--

D. aur 970 TTTTAATGAT TAATTCTAAT TATATGCGAC TTGAATAAGG CCGCCGAA--

D. mel Light 1 769 ---------- -CGATG---- ---------- ---CCAATAA A--AGCGGCG

D. yak 775 tggcTGAGCG TAGAAA---- ---------- ---CCAATAA A--AATGCCG

D. fuy 1010 ----TGAGCG AAAAAG---- ---------- ---CCAATAA A--AGTGCCG

D. aur 1018 ----TGGCCG AATGAGaaat gctctggcag cggCCAATAA AAAATGGCCG

**Abd-B 7**

D. mel Light 1 790 TGGCAAAGTG GAGTGGACTG GG-------T TTGTGTGGCG ccc-------

D. yak 807 GGGCAAAGTG GAGTGGATTT CG-------G ATGTGTGGCG cct-------

D. fuy 1038 AGGCAAAGTG GAGTGGATTT TG-------G CCGTGTGGCG CCCCgtg---

D. aur 1064 GGGCAAAGTG GAGTGttttt tttttggtcG CCGTGTGGCG CCCCagggag

D. mel Light 1 826 ----CTGCTA GTGGCACATA AAAATTGGCG CAAGTTAATT GTGGTAGTTA

D. yak 843 ----CTGCTA GTGGCACATA AAAATTGGTG CAAGTTAATT GTGGTAGTTA

D. fuy 1078 ----CTGCTA GTGGCACATA AAAATTGGCG CAAGTTAATT GTGGTAGTTA

D. aur 1114 gcgaCTCGTA GTGGGGCATA AAAATTGGTG Ct-GTTAATT GTGGTAGTTA

**Abd-B 8**

D. mel Light 1 872 TTTGCTGTTT TGCCATTTGG TCATTTTACA ATTTTACCAT TTCAg--C--

D. yak 889 TTTGCTGTTT TGCCATTTGG TCATTTTACA ATTTTACCAT TTCAccattt

D. fuy 1124 TTTGCTGTTT TGCCATTTGG CCGTTTTACA ATTTTACCAT TCT-------

D. aur 1163 TTTGCTGTTT TGCCATTTGG CCATTTCACA ATTTTACCAT TCTgccaC--

**Abd-B 9 Abd-B 10**

D. mel Light 1 918 ----CACAAC TTTTCGCACT GCTCCCcccc tttccCAGC- ----------

D. yak 939 caGCCACAAC TTTTAGCACT GCTCCCTTCG CCCGACG--- ----------

D. fuy 1167 --GCCACAAC TTTTCGCATT GCTCCGCTTG CCTGGTGCAA CAATGTTGCC

D. aur 1211 ----CACAAC TTTTCACATT GCTCTGGTTG CTCCGCGCAA CAAAGTTGCA

**DSX 2**

D. mel Light 1 961 ----ACAACA ATGTTGCGGC ATTCTCGCAC -TTTACGAGG CG-TTTTTTT

D. yak 983 ----ACAACA ATGTTGCGGC ATTCTCGCAC -TTTACGAGG CG--------

D. fuy 1215 G--------- CAGTCGCTGC ATTCTCGCAC TTTTACGAGG CG--------

D. aur 1257 tccaagagtt gcccTGCCGC ATTCTCGCAC TTTTACGAGG CGTTTTTTTT

**Dsx2 Site Abd-B 11**

D. mel Light 1 998 -----TTTAT ATCACTTACT TTACTTAGTT GATTAAGGGC GTGGCCGATG

D. yak 1012 -------TTT TTTATATCAC TTACTTAGTT GATTAAGGGC GTTGCCGATG

D. fuy 1248 -------TTT TTTATATCAC TTACTTAGTT GATTAAGGGC GTGGCCGATG

D. aur 1307 ccccttaTTT TTTATATCAC TTACTTAGTT GATTAAGGGC GTGGCCGATG

**Abd-B 12 Abd-B 13**

D. mel Light 1 1042 GGCCAGatac ATGCTTAGAT TTGCTCc--- ---------- -------AGC

D. yak 1055 GGCCAGAT-- ATGCTTAGAT TTGGTCTATG TATCCccgt- --------GG

D. fuy 1291 GGaaAGAT-- ATGTTTAGAT TTGCTCTTTG TATCCGgaca ttctatcAGG

D. aur 1357 GGCCAGAT-- ATGTTTAGAT TTGGTCTTTG TATCCGtcct aa-------G

D. mel Light 1 1072 AGTGGGCTGC ATTTTACGAC CCTCAAAACC CGATCCAAAt ggaaaatatg

D. yak 1094 AGTGGGCTGT GTTTTACGAC CCTCAAAACC CGATCGAAAC GGAAAGAGAc

D. fuy 1339 AGTGGGTTGC ATTTTACGAC CCGCCAAAGC CGATCAAAAC GGAAACAGAa

D. aur 1398 AGTGGGCTGC ATTTTACGAG CCTCGAAAGg tgatcgaaat ggctacggaa

**Abd-B 14**

D. mel Light 1 1122 aaaatac--- -----GGC-- -----TAATC CGCTTATGAG CACAACAAAT

D. yak 1144 -A---AATAT GAAATGGT-- -----TAATC CGCTTATGGT AACAACAAAA

D. fuy 1389 gA---AATAT GAAATGGCCT CTGGGTAATC CGCTTATGGT TATAACAATA

D. aur 1448 agaggAATAT CAAATGGGTT CGAGGTAATC CGCTTacgaa atgagctcct

D. mel Light 1 1157 TGgttcacAC ACTTCGATCG AAATTACTTG CGATCGCcat ttgattggtT

D. yak 1183 TGtt----AC ACTTCGATCG AAATCACTTG CGATCGCatt ttaaatggcT

D. fuy 1436 Ttatagaaat ttcgataaga tttgaaataa gatctttttt tgactattaa

D. aur 1498 tagaatcctc acactgtgat cacagctgaa ttattgccat acttatatct

D. mel Light 1 1207 TCAATGTATT GCTTTAACTG GCAGGTGAAc actttgtttt ttatcTAACG

D. yak 1229 TTAGTTTATC GCTTCAACTA GCAGGTGAAa atcgggtttt gagt-TAACG

D. fuy 1486 atggtttcaa tccactgcct tttgataatg gttaat---- ----------

D. aur 1548 gacattaaaa attctcttgt ttaccttatt tttaatgacc ttaattactg

D. mel Light 1 1257 ATTCTTACTA TTTAATATCC TAGTCaatta atGTATTTTC CACTACTTCc

D. yak 1278 ATTTGTACCA GTTATTGGCC AAGTCccctg aaGTATTTTC CAGTACTTCt

D. fuy 1522 ---------- ---------- ---------- ---------- ----------

D. aur 1598 ccttagttat aacttacata aatgggactt tatttaacag gtttcggtgg

D. mel Light 1 1307 atcgatatca cagagttccc atttcgca-- ---------- ----------

D. yak 1328 ccgttttccg accatcgacc atttcagagt ccccctttcc caatgt----

D. fuy 1522 ---------- ---------- ---------- ---------- ----------

D. aur 1648 aaatttgatt agaatggaag ttttaaggga ttttttaata agccactaag

D. mel Light 1 1335 ---------- ---------- ---------- ---------- ----------

D. yak 1374 ---------- ---------- ---------- ---------- ----------

D. fuy 1522 ---------- ---------- ---------- ---------- ----------

D. aur 1698 aaattttaca ggttacttgg ttatttgcag tattaacgtt ggaaacctaa

D. mel Light 1 1335 ---------- ---------- ---------- ---------- ----------

D. yak 1374 ---------- ---------- ---------- ---------- ----------

D. fuy 1522 ---------- ---------- ---------- ---------- ----------

D. aur 1748 acaatttttc tgttaaaaat atattttaaa cataatttaa taaatttatt

D. mel Light 1 1335 ---------- ---------- ---------- ---------- ----------

D. yak 1374 ---------- ---------- ---------- ---------- ----------

D. fuy 1522 ---------- ---------- ---------- --TTGGTTTA AAATTGTAAA

D. aur 1798 aaatagcaag attgagagct catgaatttt gcTTGTTTAA ATATTTTAAA

D. mel Light 1 1335 ---------- ---------- ---------- ---------- ----------

D. yak 1374 ---------- ---------- ---------- ---------- ----------

D. fuy 1540 TGTTcagttt ccgattaatt tctatcagcc cattttataa gaaagttttt

D. aur 1848 TGTTttaaaa ttaaattaga agcatgataa atttttaaat aataccacta

D. mel Light 1 1335 ---------- ---------- ---------- ---------- ----------

D. yak 1374 ---------- ---------- ---------- ---------- ----------

D. fuy 1590 ttttgctatt tgcctttttg tttttcaaat taatttctag gatatattaa

D. aur 1898 cgttttaaag ccaatttaag tgccgatttt attttgtaga ttttattaca

D. mel Light 1 1335 ---------- ---------- ---------- ---------- ----------

D. yak 1374 ---------- ---------- ---------- ---------- ----------

D. fuy 1640 acattctgta tttttaacca ttctttcaat aaatactttt ctttatagac

D. aur 1948 aagtcaggtt ctaaagtcta caattttagt tcggtttaat caccttaact

D. mel Light 1 1335 ---------- ---------- ---------- ---------- ----------

D. yak 1374 ---------- ---------- ---------- ---------- ----------

D. fuy 1690 taatttattt aaaaaacaaa acatgttgta tataaaaaac ttcaatttaa

D. aur 1998 ccataaccat gccaagtgaa atctttccgc tagtatctta taaaaatgtt

D. mel Light 1 1335 ---------- ---------- ---------- ---------- ----------

D. yak 1374 ---------- ---------- ---------- ---------- ----------

D. fuy 1740 ttttgatatt ttaatcaata cttaaaaacc ctgaaagcat gtttcaatta

D. aur 2048 gttctacaaa tggtgtttta tttcccaggc cttgaggtga ta--------

D. mel Light 1 1335 ---------- ---------- ---------- ---------- ----------

D. yak 1374 ---------- ---------- ---------- ---------- ----------

D. fuy 1790 atttgtaact tagtactaca tgaagtgatg TAAAGATATG TCCGCATCGA

D. aur 2090 ---------- ---------- ---------- TAAAGATACC ACCGCATCGA

D. mel Light 1 1335 ---------- ---------- ---------- ---------- -A------AG

D. yak 1374 ---------- ---------- ---------- -------CCC AA------TG

D. fuy 1840 TTTTCCAGAa acccctttca tcccctttcc cttgacaCCC CA------AG

D. aur 2110 TTTTCCAGAg agaccccctt gtcagcccac attcccctta attcccttAG

D. mel Light 1 1338 TCACATATTT GTTCTTTTAT AACATGAA-- --CGCGTACC GCGAAGG---

D. yak 1381 TCGCATATTT GTTCTTTTAT AACGCGAA-- --CGCGTACC GCGAAGG---

D. fuy 1884 CCTCATGTTT GTTATTTTAC AACGTCAcac gtCGCGAACC GAGAAGG---

D. aur 2160 CCAAATGTTT GTTATTTTAT AACGTCAA-- --CGCGTcgc gaaccgagaa

D. mel Light 1 1381 ---CCCCATA AAGTGTTCGC AATAAAATAT ATTGTGCAAT AGTT------

D. yak 1424 ---CCCCATA AAGTGTTCGT AATAAAATAT ATTGTGCAAT ATTTGTGCTA

D. fuy 1931 ---TCTCATA AAGTGTTCGT AATAAAATAT ATTGTACAAT ATTTG-GCTA

D. aur 2206 ggcCCCCATA AAGTGTTTGT AATAAAATAT ATTGTGCAAT ATTT-TGCTA

D. mel Light 1 1422 -----ATACA GCCACTCATA TACATTATAT Acaatatata tatatgtggA

D. yak 1471 TAGTTATACA GCCGCTCGTA TACATTATAT Ata------- ----------

D. fuy 1977 TAGTTATATA GCCACTCATA TACATTATAT ATAT------ ---------A

D. aur 2255 TAGTTATAGA GCCACTCATA TACATTATAT ATAT------ ---------A

D. mel Light 1 1467 TGTGTATGTG CACAACCATA TAGATGTGTt gTATATAAAT TG--------

D. yak 1504 ----TATGTG CGCAACTATA TAGATGTGTA -TATAgatat aaattgccat

D. fuy 2012 Tga------G CGCAACTATA TAGATGTGTA -TATATAgaa ggctgtccca

D. aur 2290 TATAAATGTG GGCAACTATA TAAATGTGTA -TATATATAT TG--------

D. mel Light 1 1509 CCATCCCATT GCTTatcATC GCCTTTATAG GTAGAATGTA ATTTCTTTTT

D. yak 1549 CCATCCCATT GCTT---ATC GCCTTTATAG GTAGAATGTA ATTTCTTTTT

D. fuy 2055 c--------T GCTT---ATC GCCTTTATAG GTAGAATGTA ATTTCTTTTT

D. aur 2331 CCATGCCATT GCTT---ATC GCCTTTATAG GTAGAATGTA ATTTCGTTTT

**SbfI**

D. mel Light 1 1559 ATGTGCAGTT TTGCCTGCAG G

D. yak 1596 ATGCGCAGTT TTGCCTGCAG G

D. fuy 2094 ATGTGCAGTT TTGCCTGCAG G

D. aur 2378 ATGCGCAGTT TTGCCTGCAG G
