# Supplementary material for: Recurrent Modification of a Conserved Cis-Regulatory Element Underlies Fruit Fly Pigmentation Diversity
Source: PLoS Genet. 2013 Aug 29;9(8):e1003740. doi: 10.1371/journal.pgen.1003740 (PMC3757066; doi:10.1371/journal.pgen.1003740)
Supplement: Figure S4 — Protein coding sequence variation for the bab alleles. To scale representations of the (A) Bab1 and (B) Bab2 proteins, including the BTB Domain (red) and Bab conserved domain (CD, blue). The positions of nonsynonymous differences between the Light 1 and Dark 1 sequences are annotated and compared to the amino acid states for the D. melanogaster genome strain and the outgroup species D. sechellia. The aligned DNA sequences for (C) bab1 and (D) bab2 protein-coding exons and adjacent splice donor and acceptor sequences (shown with black text on gray background. (DOC) [file pgen.1003740.s004.doc]

**
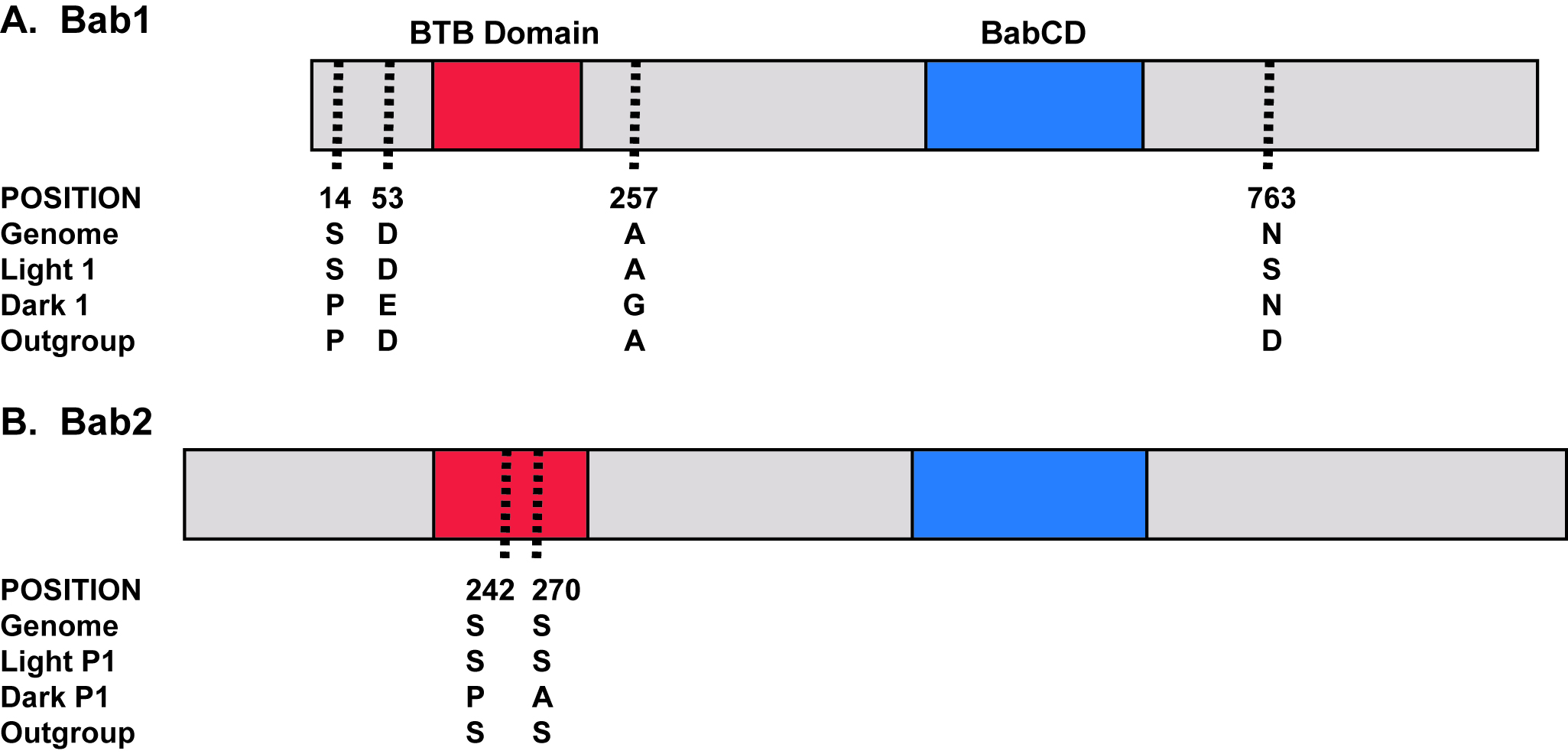
**

**C.**

**bab1 1^st^ exon**

bab1 (genome) 1 ATGGCGTCGG CGCAGGCGGA GACGAATGTC GGCTTGGCGT CCGAACAGGG

bab1 (Light P1) 1 ATGGCGTCGG CGCAGGCGGA GACGAATGTC GGCTTGGCGT CCGAACAGGG

bab1 (Dark P1) 1 ATGGCGTCGG CGCAGGCGGA GACGAATGTC GGCTTGGCGC CCGAACAGGG

bab1 (D.sec.) 1 ATGGCGTCGG CGCAGGCGGA GACGAATGTC GGTGTGGCGC CCGAACAGGG

bab1 (genome) 51 ACCAGTGGCT CAGAGGCAGC GCAAAGGGAC GGGATCGGGC GCCGATTCGC

bab1 (Light P1) 51 ACCAGTGGCT CAGAGGCAGC GCAAAGGGAC GGGATCGGGC GCCGATTCGC

bab1 (Dark P1) 51 ACCAGTGGCT CAGAGGCAGC GCAAAGGGAC GGGATCGGGC GCCGATTCGC

bab1 (D.sec.) 51 ACCAGTGGCC CAGAGGCAGC GCAAGGGGAC GGGATCGGGC GCCGATTCGC

bab1 (genome) 101 CCAAGAGTAA CAGAAGCTCG CCCACTCAGC AGGAGGAGAA GCGTATCAAA

bab1 (Light P1) 101 CCAAGAGTAA CAGAAGCTCG CCCACTCAGC AGGAGGAGAA GCGTATCAAA

bab1 (Dark P1) 101 CCAAGAGTAA CAGAAGCTCG CCCACTCAGC AGGAGGAGAA GCGTATCAAA

bab1 (D.sec.) 101 CCAAGAGTAA CAGGAGCTCG CCCACTGAGC AGGAGGAGAA GCGTATCAAA

bab1 (genome) 151 AGCGAGGATC GCACTTCACC AACTGGCGGG GCCAAGGACG AGGACAAGGA

bab1 (Light P1) 151 AGCGAGGATC GCACTTCACC AACTGGCGGG GCCAAGGACG AGGACAAGGA

bab1 (Dark P1) 151 AGCGAGGAAC GCACTTCACC CACTGGCGGG GCCAAGGACG AGGACAAGGA

bab1 (D.sec.) 151 AGCGAGGATC GCACTTCACC CACTGGCGGA GCCAAGGACG AGGAAAAGGA

bab1 (genome) 201 GAGTCAAGGT CATGCTGTAG CCGGAGGGGG AGGATCTTCG CCCGTCAGTT

bab1 (Light P1) 201 GAGTCAAGGT CATGCTGTAG CCGGAGGGGG AGGATCTTCG CCCGTCAGTT

bab1 (Dark P1) 201 GAGTCAAGGT CATGCTGTAG CCGGAGGGGG AGGATCTTCG CCCGTCAGTT

bab1 (D.sec.) 201 GAGTCAAGGT CATGCTGGAG CCGGAGGGGG AGGATCTTCG CCAGTGAGTT

bab1 (genome) 251 CGCCACAGGG CAGGAGTTCT TCGGTAGCCT CGCCCAGTTC CAGCTCCCAG

bab1 (Light P1) 251 CGCCACAGGG CAGGAGTTCT TCGGTAGCCT CGCCCAGTTC CAGCTCCCAG

bab1 (Dark P1) 251 CGCCACAGGG CAGGAGTTCT TCGGTAGCCT CGCCCAGTTC CAGCTCCCAG

bab1 (D.sec.) 251 CGCCACAGGG CAGGAGTTCT TCGGTGGCCT CGCCCAGTTC CAGCTCCCAG

bab1 (genome) 301 CAATTCTGCC TGCGCTGGAA CAACTATCAG ACGAACCTGA CCACCATCTT

bab1 (Light P1) 301 CAATTCTGCC TGCGCTGGAA CAACTATCAG ACGAACCTGA CCACCATCTT

bab1 (Dark P1) 301 CAATTCTGCC TGCGCTGGAA CAACTATCAG ACGAACCTGA CCACCATCTT

bab1 (D.sec.) 301 CAATTCTGCC TGCGCTGGAA CAACTACCAG ACGAACCTGA CCACCATCTT

bab1 (genome) 351 TGACCAGCTG CTCCAGAACG AGTGCTTCGT GGACGTGACC TTGGCATGCG

bab1 (Light P1) 351 TGACCAGCTG CTCCAGAACG AGTGCTTCGT GGACGTGACC TTGGCATGCG

bab1 (Dark P1) 351 TGACCAGCTG CTCCAGAACG AGTGTTTCGT GGACGTGACC TTGGCATGCG

bab1 (D.sec.) 351 CGACCAGCTG CTCCAGAACG AGTGCTTCGT GGACGTGACC TTGGCCTGCG

bab1 (genome) 401 ATGGTCGGTC CATGAAGGCC CACAAGATGG TCCTGTCCGC CTGCTCGCCC

bab1 (Light P1) 401 ATGGTCGGTC CATGAAGGCC CACAAGATGG TCCTGTCCGC CTGCTCGCCC

bab1 (Dark P1) 401 ATGGTCGGTC CATGAAGGCC CACAAGATGG TTCTGTCCGC CTGCTCGCCC

bab1 (D.sec.) 401 ATGGTCGCTC TATGAAGGCC CACAAGATGG TCCTGTCCGC CTGCTCGCCC

bab1 (genome) 451 TACTTCCAAA CACTTCTGGC CGAAACGCCC TGCCAGCATC CCATTGTGAT

bab1 (Light P1) 451 TACTTCCAAA CACTTCTGGC CGAAACGCCC TGCCAGCATC CCATTGTGAT

bab1 (Dark P1) 451 TACTTCCAAA CACTTCTGGC CGAAACGCCC TGCCAGCATC CCATTGTGAT

bab1 (D.sec.) 451 TACTTCCAAA CGCTTCTGGC CGAGACGCCC TGCCAGCATC CCATTGTGAT

bab1 (genome) 501 CATGCGGGAC GTAAATTGGT CGGATCTCAA GGCCATTGTG GAGTTCATGT

bab1 (Light P1) 501 CATGCGGGAC GTAAATTGGT CGGATCTCAA GGCCATTGTG GAGTTCATGT

bab1 (Dark P1) 501 CATGCGGGAC GTAAACTGGT CGGATCTCAA GGCCATTGTG GAGTTCATGT

bab1 (D.sec.) 501 CATGCGGGAC GTAAACTGGT CGGATCTCAA GGCCATTGTG GAGTTCATGT

bab1 (genome) 551 ATCGCGGCGA GATCAACGTG AGCCAGGACC AGATAGGTCC TCTGCTCAGG

bab1 (Light P1) 551 ATCGCGGCGA GATCAACGTG AGCCAGGACC AGATAGGTCC TCTGCTCAGG

bab1 (Dark P1) 551 ATCGCGGCGA GATCAACGTG AGCCAGGACC AGATAGGTCC TCTGCTCAGG

bab1 (D.sec.) 551 ACCGCGGCGA GATCAACGTG AGCCAGGACC AGATAGGTCC TCTGCTCAGA

bab1 (genome) 601 ATAGCTGAGA TGTTGAAAGT GCGTGGTCTG GCGGATGTGA CCCATATGGA

bab1 (Light P1) 601 ATAGCTGAGA TGTTGAAAGT GCGTGGTCTG GCGGATGTGA CCCATATGGA

bab1 (Dark P1) 601 ATAGCTGAGA TGTTGAAAGT GCGTGGTCTG GCGGATGTGA CCCATATGGA

bab1 (D.sec.) 601 ATAGCTGAGA TGTTGAAAGT GCGCGGTCTG GCGGATGTGA CCCACATGGA

bab1 (genome) 651 GGCGGCCACG GCAGCAGCGG CTGCCGCTTC GTCGGAGAGA ATGCCCTCCT

bab1 (Light P1) 651 GGCGGCCACG GCAGCAGCGG CTGCCGCTTC GTCGGAGAGA ATGCCCTCCT

bab1 (Dark P1) 651 GGCGGCCACG GCAGCAGCGG CTGCCGCTTC GTCGGAGAGA ATGCCCTCCT

bab1 (D.sec.) 651 GGCGGCCACG GCAGCAGCGG CTGCCGCTTC GTCGGAGAGG ATGCCCTCCT

bab1 (genome) 701 CGCCCAAGGA GAGCACTTCA ACTTCCAGAA CTGAACACGA CAGGGAACGG

bab1 (Light P1) 701 CGCCCAAGGA GAGCACTTCA ACTTCCAGAA CTGAACACGA CAGGGAACGG

bab1 (Dark P1) 701 CGCCCAAGGA GAGCACTTCA ACTTCTAGAA CTGAACACGA CAGGGAACGG

bab1 (D.sec.) 701 CGCCCAAGGA GAGTACTTCA ACTTCCAGAA CTGAACATGA CAGGGAACGG

bab1 (genome) 751 GAGGCCGAGG AGCTGCTGGC CTTCATGCAG CCCGAGAAGA AGCTACGCAC

bab1 (Light P1) 751 GAGGCCGAGG AGCTGCTGGC CTTCATGCAG CCCGAGAAGA AGCTACGCAC

bab1 (Dark P1) 751 GAGGCCGAGG AGCTGCTGGG CTTCATGCAG CCCGAGAAGA AGCTACGCAC

bab1 (D.sec.) 751 GAGGCCGAGG AGCTACTGGC CTTCATGCAG CCCGAGAAGA AGCTACGCAC

bab1 (genome) 801 TTCGGACTGG GATCCCGCTG AGCTGAGGCT CTCCCCACTG GAGCGGCAGC

bab1 (Light P1) 801 TTCGGACTGG GATCCCGCTG AGCTGAGGCT CTCCCCACTG GAGCGGCAGC

bab1 (Dark P1) 801 TTCGGACTGG GATCCCGCTG AGCTGAGGCT TTCCCCACTG GAGCGGCAGC

bab1 (D.sec.) 801 TTCGGACTGG GATCCCGCTG AGCTGAGGCT TTCCCCACTG GAGCGGCAGC

bab1 (genome) 851 AGGGCAGGAA TGTAAGAAAG CGCCGGTGGC CATCGGCGGA CACAATATTC

bab1 (Light P1) 851 AGGGCAGGAA TGTAAGAAAG CGCCGGTGGC CATCGGCGGA CACAATATTC

bab1 (Dark P1) 851 AGGGCAGGAA TGTAAGAAAG CGCCGGTGGC CATCGGCGGA CACAATATTC

bab1 (D.sec.) 851 AGGGCAGGAA TGTGAGAAAG CGCCGGTGGC CCTCGGCGGA CACAATATTC

bab1 (genome) 901 AATCCACCCG CACCACCCAG TCCACTGAGC AGCCTGATTG CGGCCGAAAG

bab1 (Light P1) 901 AATCCACCCG CACCACCCAG TCCACTGAGC AGCCTGATTG CGGCCGAAAG

bab1 (Dark P1) 901 AATCCACCCG CACCACCCAG TCCACTGAGC AGCCTGATTG CGGCCGAAAG

bab1 (D.sec.) 901 AATCCACCCG CACCACCCAG TCCACTGAGC AGCCTGATAG CCGCCGAAAG

bab1 (genome) 951 GATGGAGCTG GAGCAAAAGG AAAGAGAGAG ACAGAGGGAC TGTTCGCTGA

bab1 (Light P1) 951 GATGGAGCTG GAGCAAAAGG AAAGAGAGAG ACAGAGGGAC TGTTCGCTGA

bab1 (Dark P1) 951 GATGGAGCTG GAGCAAAAGG AAAGAGAGAG ACAGAGGGAC TGTTCGCTGA

bab1 (D.sec.) 951 GATGGAGCTG GAGCAAAAGG AAAGAGAGAG ACAGAGGGAC TGTTCGCTGA

bab1 (genome) 1001 TGACACCCCC ACCCAAACCA CCAATGAGCA GTGGCTCCAC AGTGGGAGCC

bab1 (Light P1) 1001 TGACACCCCC ACCCAAACCA CCAATGAGCA GTGGCTCCAC AGTGGGAGCC

bab1 (Dark P1) 1001 TGACACCCCC ACCCAAACCA CCAATGAGCA GTGGCTCCAC AGTGGGAGCC

bab1 (D.sec.) 1001 TGACACCTCC ACCCAAACCA CCACTGAGCA GTGGCTCCGC AGCGGGAGCC

bab1 (genome) 1051 ACGAGGCGCC TGGAGACCGC CATCCACGCC CTGGACATGC CATCGCCGGC

bab1 (Light P1) 1051 ACGAGGCGCC TGGAGACCGC CATCCACGCC CTGGACATGC CATCGCCGGC

bab1 (Dark P1) 1051 ACGAGGCGCC TGGAGACCGC CATCCACGCC CTGGACATGC CATCGCCGGC

bab1 (D.sec.) 1051 ACGAGGCGCT TGGAGACCGC TATCCATGCT CTGGACATGC CATCGCCGGC

bab1 (genome) 1101 TGCCACGCCA GGACC-TCTG TCCCGATCGT CGA-GACCTC ACTCGCAGAG

bab1 (Light P1) 1101 TGCCACGCCA GGACC-TCTG TCCCGATCGT CGA-GACCTC ACTCGCAGAG

bab1 (Dark P1) 1101 TGCCACGCCA GGACC-TCTG TCCCGATCGT CGA-GACCTC ACTCGCAGAG

bab1 (D.sec.) 1101 TGCCACGCCA GGACC-TCTC TCCCGATCCT CGA-GACCAC ACTCGCAGAG

bab1 (genome) 1149 CCCCCAGCAG CAGCAGGCAC AGCAGCAGGG TCAGCTTCCT TTGCCCCTGC

bab1 (Light P1) 1149 CCCCCAGCAG CAGCAGGCAC AGCAGCAGGG TCAGCTTCCT TTGCCCCTGC

bab1 (Dark P1) 1149 CCCCCAGCAG CAGCAGGCAC AGCAGCAGGG TCAGCTTCCT TTGCCCCTGC

bab1 (D.sec.) 1149 CCCCCAGCAG CAGCAGGCAC AGCAGCAGGG TCAGCTTCCT TTGCCCCTGC

bab1 (genome) 1199 CCCTGCATCC GCACCATCAC GCATCACCCG CCCCACATCC CTCCCAGACC

bab1 (Light P1) 1199 CCCTGCATCC GCACCATCAC GCATCACCCG CCCCACATCC CTCCCAGACC

bab1 (Dark P1) 1199 CCCTGCATCC GCACCATCAC GCATCACCCG CCCCACATCC CTCCCAGACC

bab1 (D.sec.) 1199 CCCTGCATCC GCACCACCAC GCATCACCCG CCCCACATCC CTCCCAGACC

bab1 (genome) 1249 GCCGGATCAG CCCACCACCC -GGCATCGCC TGCTGGAGAT TCCCGTTTTC

bab1 (Light P1) 1249 GCCGGATCAG CCCACCACCC -GGCATCGCC TGCTGGAGAT TCCCGTTTTC

bab1 (Dark P1) 1249 GCCGGATCAG CCCACCACCC -GGCATCGCC TGCTGGAGAT TCCCGTTTTC

bab1 (D.sec.) 1249 GCCGGATCAG CCCACCACCC -GCCATCGCC CGCTGGAGAC TCCCGTTTTT

bab1 (genome) 1298 CCCTCGGCCC AGCAGCCGCC ATGGCCGCTG CCAGGGAACT GAGTGGCCTG

bab1 (Light P1) 1298 CCCTCGGCCC AGCAGCCGCC ATGGCCGCTG CCATGGAACT GAGTGGCCTG

bab1 (Dark P1) 1298 CCCTCGGCCC AGCAGCCGCC ATGGCCGCTG CCATGGAACT GAGTGGCCTG

bab1 (D.sec.) 1298 CCCTCGGACC CGCAGCCGCC ATGGCCGCTG CCATGGAACT GAGTGGCCTG

bab1 (genome) 1348 GGACCAGGTC CGTCCGCCGA GCCACGCCTT CCGCCTCCAC CGCCGCACCA

bab1 (Light P1) 1348 GGACCAGGTC CGTCCGCCGA GCCACGCCTT CCGCCTCCAC CGCCGCACCA

bab1 (Dark P1) 1348 GGACCAGGTC CGTCCGCCGA GCCACGCCTT CCGCCTCCAC CGCCGCACCA

bab1 (D.sec.) 1348 GGACCTGGTC CCTCCGCCGA GCCACGCCTA CCGCCTCCAC CGCCGCACCA

bab1 (genome) 1398 CCATGGCGGT GGTGGAGTGG GCGGCGGGGG AGTTGGAGGA GGAGGTGCAG

bab1 (Light P1) 1398 CCATGGCGGT GGTGGAGTGG GCGGCGGGGG AGTTGGAGGA GGAGGTGCAG

bab1 (Dark P1) 1398 CCATGGCGGT GGTGGAGTGG GCGGCGGGGG AGTTGGAGGA GGAGGTGCAG

bab1 (D.sec.) 1398 CCATGGCGGT GGTGGAGTGG GCGGTGGGGG AGTT---GGA GGAGGTGCAG

bab1 (genome) 1448 GCGGAGTGGG TTCAGGCGGG GGATCCTCGC TCGCCGATGA CTTGGAGATC

bab1 (Light P1) 1448 GCGGAGTGGG TTCAGGCGGG GGATCCTCGC TCGCCGATGA CTTGGAGATC

bab1 (Dark P1) 1448 GCGGAGTGGG TTCAGGCGGG GGATCCTCGC TCGCCGATGA CTTGGAGATC

bab1 (D.sec.) 1445 GCGGAGTGGG TTCAGGCGGG GGATCCTCGC TCGCCGATGA CTTGGAGATC

bab1 (genome) 1498 AAGCCAGGGA TCGCCGAGAT GATCCGAGAG GAAGAAAGGG TGAGT-----

bab1 (Light P1) 1498 AAGCCAGGGA TCGCCGAGAT GATCCGAGAG GAAGAAAGGG TGAGT-----

bab1 (Dark P1) 1498 AAGCCAGGGA TCGCCGAGAT GATCCGAGAG GAAGAAAGGG TGAGT-----

bab1 (D.sec.) 1495 AAGCCAGGGA TCGCCGAGAT GATCCGAGAG GAAGAAAGGG TGAGT-----

**bab1 2nd exon**

bab1 (genome) 1 CAGGCCAAAA TGATGGAGAA CTCGCACGCC TGGATGGGCG CCACCGGATC

bab1 (Light P1) 1 CAGGCCAAAA TGATGGAGAA CTCGCACGCC TGGATGGGCG CCACCGGATC

bab1 (Dark P1) 1 CAGGCCAAAA TGATGGAGAA CTCGCACGCC TGGATGGGCG CCACCGGATC

bab1 (D.sec.) 1 CAGGCCAAAA TGATGGAGAA CTCGCACGCC TGGATGGGCG CCACCGGATC

bab1 (genome) 51 AACGCTGGCA GGTTCGT

bab1 (Light P1) 51 AACGCTGGCA GGTTCGT

bab1 (Dark P1) 51 AACGCTGGCA GGTTCGT

bab1 (D.sec.) 51 CACGCTGGCA GGTTCGT

**bab1 3rd exon**

bab1 (genome) 1 CAGCAGACAG CTACCAGTAC CAGCTGCAGT CCATGTGGCA AAAGTGCTGG

bab1 (Light P1) 1 CAGCAGACAG CTACCAGTAC CAGCTGCAGT CCATGTGGCA AAAGTGCTGG

bab1 (Dark P1) 1 CAGCAGACAG CTACCAGTAC CAGCTGCAGT CAATGTGGCA AAAGTGCTGG

bab1 (D.sec.) 1 CAGCAGACAG CTACCAGTAC CAGCTGCAGT CCATGTGGCA GAAGTGCTGG

bab1 (genome) 51 AACACCAACC AGAATCTGAT GCATCACATG CGCTTCCGCG AGCGAGGTCC

bab1 (Light P1) 51 AACACCAACC AGAATCTGAT GCATCACATG CGCTTCCGCG AGCGAGGTCC

bab1 (Dark P1) 51 AACACCAACC AGAATCTGAT GCATCACATG CGCTTCCGCG AGCGAGGTCC

bab1 (D.sec.) 51 AACACCAACC AGAACCTGAT GCACCACATG CGCTTCCGCG AGCGAGGTCC

bab1 (genome) 101 TCTGAAGTCG TGGCGACCCG AGACCATGGC GGAGGCCATT TTCAGTGTGC

bab1 (Light P1) 101 TCTGAAGTCG TGGCGACCCG AGACCATGGC GGAGGCCATT TTCAGTGTGC

bab1 (Dark P1) 101 TCTGAAGTCC TGGCGACCCG AGACCATGGC GGAGGCCATT TTCAGTGTGC

bab1 (D.sec.) 101 TCTCAAGTCC TGGCGTCCGG AGACCATGGC GGAGGCCATT TTCAGTGTGC

bab1 (genome) 151 TAAAGGAGGG TCTATCGCTA TCTCAGGCCG CCCGCAAGTA CGACATCCCG

bab1 (Light P1) 151 TAAAGGAGGG CCTATCGCTA TCCCAGGCCG CCCGCAAGTA CGATATCCCG

bab1 (Dark P1) 151 TAAAGGAGGG TCTATCGCTA TCTCAGGCCG CCCGCAAGTA CGACATCCCG

bab1 (D.sec.) 151 TGAAGGAGGG CCTTTCGCTC TCCCAGGCCG CCCGCAAGTA CGACATCCCG

bab1 (genome) 201 TATCCAACAT TCGTGCTCTA TGCGAACAGG GTGCACAATA TGCTGGGACC

bab1 (Light P1) 201 TATCCAACAT TCGTGCTCTA TGCGAACAGG GTGCACAATA TGCTGGGACC

bab1 (Dark P1) 201 TATCCAACAT TCGTGCTCTA TGCGAACAGG GTGCACAATA TGCTGGGACC

bab1 (D.sec.) 201 TATCCCACGT TCGTGCTCTA TGCCAACAGG GTGCACAATA TGCTGGGACC

bab1 (genome) 251 ATCCATTGAC GGCGGGCCCG ATTTGCGGCC CAAGGGGCGT GGCAGGCCGC

bab1 (Light P1) 251 TTCCATTGAC GGCGGGCCCG ATTTGCGGCC CAAGGGGCGT GGCAGGCCGC

bab1 (Dark P1) 251 ATCCATTGAC GGCGGGCCCG ATTTGCGGCC CAAGGGGCGT GGCAGGCCGC

bab1 (D.sec.) 251 TTCCATTGAC GGCGGGCCCG ATCTGCGGCC CAAGGGGCGT GGCAGGCCGC

bab1 (genome) 301 AGCGAATCCT TCTGGGCATC TGGCCCGACG AGCACATTAA GGGCGTCATC

bab1 (Light P1) 301 AGCGAATCCT TCTGGGCATC TGGCCCGACG AGCACATTAA GGGCGTCATC

bab1 (Dark P1) 301 AGCGAATCCT TTTGGGCATC TGGCCCGACG AGCACATTAA GGGCGTCATC

bab1 (D.sec.) 301 AGCGAATCCT TCTGGGCATC TGGCCCGACG AGCACATCAA GGGCGTCATC

bab1 (genome) 351 AAGACGGTGG TCTTTCGCGA CACCAAGGAC ATCAAGGACG AGAGCCTGGC

bab1 (Light P1) 351 AAGACGGTGG TCTTTCGCGA CACCAAGGAC ATCAAGGACG AGAGCCTGGC

bab1 (Dark P1) 351 AAGACGGTGG TCTTTCGCGA CACCAAGGAC ATCAAGGACG AGAGCCTGGC

bab1 (D.sec.) 351 AAGACGGTGG TCTTTCGCGA CACCAAGGAC ATCAAGGACG AGAGCCTAGC

bab1 (genome) 401 CGCTCACATG CCACCCTACG GTCGACATTC GGTAGGT

bab1 (Light P1) 401 CGCCCACATG CCACCTTACG GTCGACATTC GGTAGGT

bab1 (Dark P1) 401 CGCCCACATG CCACCCTACG GTCGACATTC GGTAGGT

bab1 (D.sec.) 401 CGCCCACATG CCACCCTACG GTCGACATTC GGTAGGT

**bab1 4th exon**

bab1 (genome) 1 AGCCCGCGTT TCCCTTGCAG GACCTCCCTC TCAGCTATCC CGGAGCCAGT

bab1 (Light P1) 1 AGCCCGCGTT TCCATTGCAG GACCTCCCTC TCAGCTATCC CGGAGCCAGT

bab1 (Dark P1) 1 AGCCCGCGTT TCCCTTGCAG GACCTCCCTC TCAGCTATCC CGGAGCCAGT

bab1 (D.sec.) 1 AGCCCGCGTT TCCCTTGCAG GACCTCCCTC TCAGCTATCC CGGAGCCAGT

bab1 (genome) 51 GGCGCCCTGG CAGGCGCGCC CAGCTCCATG GCCTGTCCGA ATGGCAGTGG

bab1 (Light P1) 51 GGCGCCCTGG CAGGCGCGCC CAGCTCCATG GCCTGTCCGA ATGGCAGTGG

bab1 (Dark P1) 51 GGCGCCCTGG CAGGCGCGCC CAGCTCCATG GCCTGTCCGA ATGGCAGTGG

bab1 (D.sec.) 51 GGCGCCTTGG CAGGCGCGCC CAGCTCCTTG GCCTGTCCGA ATGGCAGTGG

bab1 (genome) 101 ACCGCAGACC GGAGTGGGCG TGGCCGGAGA GCAGCATATG TCACAGGAAA

bab1 (Light P1) 101 ACCGCAGACC GGAGTGGGCG TGGCCGGAGA GCAGCATATG TCACAGGAAA

bab1 (Dark P1) 101 ACCGCAGACC GGAGTGGGCG TGGCCGGAGA GCAGCATATG TCACAGGAAA

bab1 (D.sec.) 101 ACCGCAGACC GGAGTGGGCG TGGCCGGAGA GCAGCACATG TCGCAGGAAA

bab1 (genome) 151 CGGCCGCCGC GGTGGCCGCC GTGGCGCACA ACATCCGCCA GCAGATGCAA

bab1 (Light P1) 151 CGGCCGCCGC GGTGGCCGCC GTGGCGCACA ACATCCGCCA GCAGATGCAA

bab1 (Dark P1) 151 CGGCCGCCGC GGTGGCCGCC GTGGCGCACA ACATCCGCCA GCAGATGCAA

bab1 (D.sec.) 151 CGGCCGCCGC GGTGGCCGCC GTGGCGCACA ACATCCGCCA GCAGATGCAA

bab1 (genome) 201 ATGGCAGCGG TTCCGCCCGG CTTATTCAAT CTGCCGCCTC ATCCGGGAGT

bab1 (Light P1) 201 ATGGCAGCGG TTCCGCCCGG CTTATTCAAT CTGCCGCCTC ATCCGGGAGT

bab1 (Dark P1) 201 ATGGCAGCGG TTCCGCCCGG CTTATTCAAT CTGCCGCCTC ATCCGGGAGT

bab1 (D.sec.) 201 ATGGCAGCGG TTCCGCCCGG CTTATTCAAT CTGCCGCCTC ATCCGGGAGT

bab1 (genome) 251 GGGCGGTGGA GTGGGCAACG TTCCCGGCGC AGCTGGAGGC AGGGCCAGCA

bab1 (Light P1) 251 TGGCGGTGGA GTGGGCAGCG TTCCCGGCGC AGCTGGAGGC AGGGCCAGCA

bab1 (Dark P1) 251 TGGCGGTGGA GTGGGCAACG TTCCCGGCGC AGCTGGAGGC AGGGCCAGCA

bab1 (D.sec.) 251 GGGCGGTGGA GTGGGCAGCG TTCCCGGCGC AGCTGGAGGC AGGGCCAGCA

bab1 (genome) 301 TATCGCCGGC CCTGAGCAGT GGCTCCGGAC CAAGGCACGC TCCCTCGCCC

bab1 (Light P1) 301 TATCGCCGGC CCTGAGCAGT GGCTCCGGAC CAAGGCACGC TCCCTCGCCC

bab1 (Dark P1) 301 TATCGCCGGC CCTGAGCAGT GGCTCCGGAC CAAGGCACGC TCCCTCGCCC

bab1 (D.sec.) 301 TATCGCCGGC CCTGAGCAGT GGCTCCGGGC CCAGGCACGC TCCCTCGCCC

bab1 (genome) 351 TGCGGTCCCG CCGGCCTCCT GCCGAACCTG CCGCCCAGCA TGGCCGTCGC

bab1 (Light P1) 351 TGCGGTCCCG CCGGCCTCCT GCCGAACCTG CCGCCCAGCA TGGCCGTCGC

bab1 (Dark P1) 351 TGCGGTCCCG CCGGCCTCCT GCCGAACCTG CCGCCCAGCA TGGCCGTCGC

bab1 (D.sec.) 351 TGCGGGCCCG CCGGCCTCCT GCCG------ ---CCCAGCA TGGCCGTCGC

bab1 (genome) 401 TCTGCACCAC CAGCAGCAAC AGCAGGCGGC GCACCACCAC ATGCAGCAGC

bab1 (Light P1) 401 TCTGCACCAC CAGCAGCAAC AGCAGGCGGC GCACCACCAC ATGCAGCAGC

bab1 (Dark P1) 401 TCTGCACCAC CAGCAGCAAC AGCAGGCGGC GCACCACCAC ATGCAGCAGC

bab1 (D.sec.) 392 TCTGCACCAC CAGCAGCAAC AGCAGGCGGC GCACCACCAC ATGCAGCAGC

bab1 (genome) 451 TGCACCTGCA GCAGCAACAG GCCCACTTGC ACCACCATCA G---------

bab1 (Light P1) 451 TGCACCTGCA GCAGCAACAG GCCCACTTGC ACCACCATCA GCAGCAACAG

bab1 (Dark P1) 451 TGCACCTGCA GCAGCAACAG GCCCACTTGC ACCACCATCA GCAGCAACAG

bab1 (D.sec.) 442 TCCACCTGCA GCAGCAACAG GCCCACTTGC ACCACCATCA ----------

bab1 (genome) 491 ---CAGCAAC AGCAACAGCA GCAGCAGCAG CACCATCAGG GCGGCCATCA

bab1 (Light P1) 501 CAACAGCAGC AGCAGCAGCA GCAGCAGCAG CACCATCAGG GCGGCCATCA

bab1 (Dark P1) 501 CAACAGCAGC AGCAGCAGCA GCAGCAGCAG CACCATCAGG GCGGCCATCA

bab1 (D.sec.) 482 ---------- -------GCA GCAGCAGCAG CACCATCAGG GCGGCCATCA

bab1 (genome) 539 GGTGGCCCAC AAGTCCGGTT TCGGTGCCAG CTCCAGTTCC TCAGCCTCCT

bab1 (Light P1) 551 GGTGGCCCAC AAGTCCGGTT TCGGTGCCAG CTCCAGTTCC TCAGCCTCCT

bab1 (Dark P1) 551 GGTGGCCCAC AAGTCCGGTT TCGGTGCCAG CTCCAGTTCC TCAGCCTCCT

bab1 (D.sec.) 515 GGTGGCCCAC AAGTCCGGTT TCGGTGCCAG CTCCAGTTCC TCAGCCTCCT

bab1 (genome) 589 CGTCGTCAAT GGGCCAGCAC CATGCGCCCA AGGCCAAGAG CAGTCCGTTG

bab1 (Light P1) 601 CGTCGTCAAT GGGCCAGCAC CATGCGCCCA AGGCCAAGAG CAGTCCGTTG

bab1 (Dark P1) 601 CGTCGTCAAT GGGCCAGCAC CATGCGCCCA AGGCCAAGAG CAGTCCGTTG

bab1 (D.sec.) 565 CGTCGTCAAT GGGCCAGCAC CATGCGCCCA AGGCCAAGAG CAGTCCGTTG

bab1 (genome) 639 CGCAGCGAAA CGCCTCGGCT GCACTCCCCG CTCGGCGATC TTGGCCTGGA

bab1 (Light P1) 651 CGCAGCGAAA CGCCTCGCCT GCACTCCCCG CTCGGCGATC TTGGCCTGGA

bab1 (Dark P1) 651 CGCAGCGAAA CGCCTCGCCT GCACTCCCCG CTCGGCGATC TTGGCCTGGA

bab1 (D.sec.) 615 CGCAGCGAAA CGCCTCGGCT GCACTCCCCG CTCGGCGATC TTGGCCTGGA

bab1 (genome) 689 CATGGCCAGC TACAAGCGCG AATTCTCGCC CAGCCGCCTC TTCGCCGAGG

bab1 (Light P1) 701 CATGGCCAGC TACAAGCGCG AATTCTCGCC CAGCCGCCTC TTCGCCGAGG

bab1 (Dark P1) 701 CATGGCCAGC TACAAGCGCG AATTCTCGCC CAGCCGCCTC TTCGCCGAGG

bab1 (D.sec.) 665 CATGGCCAGC TACAAGCGCG AGTTCTCGCC CAGCCGCCTC TTCGCCGAGG

bab1 (genome) 739 ATCTGGCCGA GCTGGTGGGC GCCAGTGTCT CATCTTCCTC ATCATCGGCG

bab1 (Light P1) 751 ATCTGGCCGA GCTGGTGGGC GCCAGTGTCT CATCTTCCTC ATCATCGGCG

bab1 (Dark P1) 751 ATCTGGCCGA GCTGGTGGGC GCCAGTGTCT CATCTTCCTC ATCATCGGCG

bab1 (D.sec.) 715 ATCTGGCCGA GCTGGTGGGC GCCAGTGTCT CCTCTTCCTC ATCTTCGGCG

bab1 (genome) 789 GCGGCAGCGA CGGCTCCTCC GGAAAGATCG GCAGGAGCAG CTTCCGCAGC

bab1 (Light P1) 801 GCGGCAGCGA CGGCTCCTCC GGAAAGATCG GCGGGAGCAG CTTCCGCAGC

bab1 (Dark P1) 801 GCGGCAGCGA CGGCTCCTCC GGAAAGATCG GCGGGAGCAG CTTCCGCAGC

bab1 (D.sec.) 765 GCGGCAGCGA CGGCTCCTCC AGAAAGAGCG GCGGGAGCAG CTTCCGCAGC

bab1 (genome) 839 CACAGGCGCG GATGCACCCA GTTCCTCGAG CAGTGGAGGC ATCAAGGTGG

bab1 (Light P1) 851 CGCAGGCGCG GATGCACCCA GTTCCTCGAG CAGTGGAGGC ATCAAGGTGG

bab1 (Dark P1) 851 CGCAGGCGCG GATGCACCCA GTTCCTCGAG CAGTGGAGGC ATCAAGGTGG

bab1 (D.sec.) 815 CGCAGGCGCA GATGCATCCA GTTCCTCGAG CAGTGGAGGC ATCAAGGTGG

bab1 (genome) 889 AACCCATTAC CACCACTAGC GAGTAAAGGG AGTAAAGGGA GGGTGAAACG

bab1 (Light P1) 901 AACCCATTAC CACGACTAGC GAGTAAAGGG AGTAAAGGGA GGGTGAAACG

bab1 (Dark P1) 901 AACCCATTAC CACCACTAGC GAGTAAAGGG AGTAAAGGGA GGGTGAAACG

bab1 (D.sec.) 865 AACCCATCAC CACCACTAGC GAGTAAAGGG AG-------- -GGTGAAACG

bab1 (genome) 939 AAGGAAATGA TAAAGTTGA- ---------- -GAAATGATA ATGGGTGAAT

bab1 (Light P1) 951 AAGGAAATGA TAAAGTTGAG AT-------- -GAAATGATA ATGGGTGAAT

bab1 (Dark P1) 951 AAGGAAATGA TAAAGTTGAG ATGAAAGTTG AGAAATGATA ATGGGTGAAT

bab1 (D.sec.) 906 AACGAAATGA TAAG------ -TGAAAGTTG AGAAATATTA ATGGGTGAAT

bab1 (genome) 977 GAACGCAAAT CAGAAGCTTC GGCAGCTTTA CTTGGCCTG

bab1 (Light P1) 992 GAACGCAAAT CAGAAGCTTC GGCAGCTTTA CTTGGCCTG

bab1 (Dark P1) 1001 GAACGCAAAT CAGAAGCTTC GGCAGCTTTA CTTGGCCTG

bab1 (D.sec.) 949 GAACGCGAAT CAGAAGCTTC GGCAGCTTTA CTTGGCCTG

**D.**

**bab2 2nd exon**

Genome 1 ATGGACATGA CCAAACAGAT TGTGGACTTT GAAATAAAGT CGGAACTGAT

Light P1 1 ATGGACATGA CCAAACAGAT TGTGGACTTT GAAATAAAGT CGGAACTGAT

Dark P1 1 ATGGACATGA CCAAACAGAT TGTGGACTTT GAAATAAAGT CGGAACTGAT

D. sechellia 1 ATGGACATGA CCAAACAGAT TGTGGACTTT GAAATAAAGT CGGAACTGCT

Genome 51 CGGCGAAATC GATCAGTTCG AGGCGAGTGA CTACACAATG GCTCCACCGG

Light P1 51 CGGCGAAATC GATCAGTTCG AGGCGAGTGA CTACACAATG GCTCCACCGG

Dark P1 51 CGGCGAAATC GATCAGTTCG AGGCGAGTGA CTACACAATG GCTCCACCGG

D. sechellia 51 CGGCGAAATC GATCAGTTCG AGGCGAGTGA CTACACAATG GCTCCACAGG

Genome 101 AAGAGCCTAA GATGGTGGAA GAGTCCCCCC AGTTGGGTCA TCTAGAGGAC

Light P1 101 AAGAGCCTAA GATGGTGGAA GAGTCCACCC AGTTGGGTCA CCTAGAGGAC

Dark P1 101 AAGAGCCTAA GATGGTGGAA GAGTCCCCCC AGTTGGGTCA TCTAGAGGAC

D. sechellia 101 AGGAGCCTAA GATGGTGGCA GAGTCCCCCC AGTTGGAGCA TCTCGAGGAC

Genome 151 CAGAACAGAA AGTACTCACC CGAAAGGGAG GTTGAACCCA CTCTGCAGGA

Light P1 151 CAGAACAGAA AGTACACACC CGAAAGGGAG GTTGAACCCA CTTTGCAGGA

Dark P1 151 CAGAACAGAA AGTACTCACC CGAAAGGGAG GTTGAACCCA CTCTGCAGGA

D. sechellia 151 CAGAACAGAA AGTACTCCCC CGAAAGAGAG GTGGAACCCA CTCTGGAGGA

Genome 201 TCCAAGTGAG GTGGTTGATC AAATGCAAAA AGATACGGAG AGCGTTGGAG

Light P1 201 TCCAAGTGAG GTGGTTGATC AAATGCAAAA AGATGCTGAG AACGTTGGAG

Dark P1 201 TCCAAGTGAG GTGGTTGATC AAATGCAAAA AGATACGGAG AGCGTTGGAG

D. sechellia 201 TCAGGGTGAG ATGGTTGATC AAATGCAAAA AGATGCTGAG AGCGTTGGCG

Genome 251 AAGTCAAGTC ACCCGAGAAG GATGTGGAAA CGGAGCTGGT GAAGTCCAAG

Light P1 251 AAGTCAAGTC ACCCGAGAAG GATGTGGAAA CGGAGCTGGT GAAGTCCAAG

Dark P1 251 AAGTCAAGTC ACCCGAGAAG GATGTGGAAA CGGAGCTGGT GAAGTCCAAG

D. sechellia 251 AAGTGAAGTC ACCCGAGAAA GATGTGGAAA CGGAGCTGGT GAAGCCCAAG

Genome 301 GCGAGTCCGA TGAACGACCA AGCTTTGACT CCCCCACCAC GACCTCTGAC

Light P1 301 GCGAGTCCGA TGAACGACCA AGCTTTGACT CCCCCACCAC GACCTCTGAC

Dark P1 301 GCGAGTCCGA TGAACGACCA AGCTTTGACT CCCCCACCAC GACCTCTGAC

D. sechellia 301 GAGAGTCCGA TGAACGACCA AGCTCTGACT CCCCCACCAC GACCTCTGAC

Genome 351 CTCCAGTGAA GTGGTGGGTC TCCGGGATCC CGAACATACC GAGCTGCGCA

Light P1 351 CTCCAGTGAA GTGGTGGGTC TCCGGGATCC CGAACACACC GAGCTGCGCA

Dark P1 351 CTCCAGTGAA GTGGTGGGTC TCCGGGATCC CGAACACACC GAGCTGCGCA

D. sechellia 351 CTCCAGTGAA GTGGTGGGTC TCCGGGATCC CGAGCACACC GAGCTGCGCA

Genome 401 TGTGCCTGGA GGCCAAGAAG TCGCGCTCCC TACCAGTTTC CCCACAGCCT

Light P1 401 TGTGCCTGGA GGCCAAGAAG TCGCGCTCCC TACCAGTTTC CCCACAGCCT

Dark P1 401 TGTGCCTGGA GGCCAAGAAG TCGCGCTCCC TACCAGTTTC CCCACAGCCT

D. sechellia 401 TGTGCCTGGA GTCCAAGAAG TCGCGCTCCC TACCGGTTTC CCCACAGCCC

Genome 451 CAACCAAATC TTAAGCTAGC CGGATCGGCG CTCTTTGAAT TCGGTCAGAG

Light P1 451 CAACCAAATC TTAAGCTAGC CGGATCGGCG CTCTTTGAAT TCGGTCAGAG

Dark P1 451 CAACCAAATC TTAAGCTAGC CGGATCGGCG CTCTTTGAAT TCGGTCAGAG

D. sechellia 451 CAACAAAGTC TTAAGCTAGC CGGATCGGCG CTCTTTGAAT TCGGCCAAAG

Genome 501 ATCCTCTCCC GTGGAGACCA AGATCAAAAC CAATCCAGAG ACAAAACCGC

Light P1 501 ATCCTCTCCC GTGGAGACCA AGATCAAAAC CAATCCGGAG ACAAAACCGC

Dark P1 501 ATCCTCTCCC GTGGAGACCA AGATCAAAAC CAATCCAGAG ACAAAACCGC

D. sechellia 501 ATCCTCTCCA GTGGAGACCA AGATCAAAAC CAATCCCGAG ACGAAACCGC

Genome 551 CGAGGCGCAA AATAGTTCCT CCCAGCGGCG AGGGGCAGCA GTTCTGCCTG

Light P1 551 CGAGGCGCAA AATAGTTCCT CCCAGCGGCG AGGGTCAGCA GTTCTGCCTG

Dark P1 551 CGAGGCGCAA AATAGTTCCT CCCAGCGGCG AGGGGCAGCA GTTCTGCCTG

D. sechellia 551 CGAGACGCAA AATAGTTCCT CCCAGCGGCG AGGGGCAGCA GTTCTGCCTG

Genome 601 AGGTGGAACA ACTATCAGTC TAACCTGACC AATGTCTTTG ACGAACTCCT

Light P1 601 AGGTGGAACA ACTATCAGTC TAACCTGACC AATGTCTTTG ACGAACTACT

Dark P1 601 AGGTGGAACA ACTATCAGTC TAACCTGACC AATGTCTTTG ACGAACTCCT

D. sechellia 601 AGGTGGAACA ACTATCAGTC CAACCTGACC AATGTCTTTG ACGAACTCCT

Genome 651 TCAGAGCGAG TCCTTCGTGG ACGTGACCTT GTCCTGCGAA GGCCACTCGA

Light P1 651 TCAGAGCGAG TCCTTCGTGG ACGTGACCTT GTCCTGCGAA GGCCACTCGA

Dark P1 651 TCAGAGCGAG TCCTTCGTGG ACGTGACCTT GTCCTGCGAA GGCCACTCGA

D. sechellia 651 GCAGAGCGAG TCCTTCGTGG ACGTTACCTT GTCCTGCGAA GGCCACTCGA

Genome 701 TCAAGGCACA CAAGATGGTG CTATCCGCCT GCTCACCCTA CTTCCAGGCC

Light P1 701 TCAAGGCCCA CAAGATGGTG CTATCCGCCT GCTCACCCTA CTTCCAGGCC

Dark P1 701 TCAAGGCACA CAAGATGGTG CTACCCGCCT GCTCACCCTA CTTCCAGGCC

D. sechellia 701 TCAAGGCCCA CAAGATGGTG CTATCCGCCT GCTCGCCCTA CTTCCAGGCC

Genome 751 CTGTTCTACG ACAATCCCTG CCAGCACCCC ATCATCATCA TGCGGGACGT

Light P1 751 CTGTTCTACG ACAATCCCTG CCAGCATCCC ATCATCATCA TGCGGGACGT

Dark P1 751 CTGTTCTACG ACAATCCCTG CCAGCACCCC ATCATCATCA TGCGGGACGT

D. sechellia 751 CTGTTCTACG ACAATCCCTG CCAGCACCCC ATCATCATCA TGCGGGACGT

Genome 801 CAGCTGGTCC GACCTGAAGG CCCTGGTGGA GTTCATGTAC AAGGGGGAGA

Light P1 801 CAGCTGGTCC GATCTGAAGG CCCTGGTGGA GTTCATGTAC AAGGGGGAGA

Dark P1 801 CAGCTGGGCC GACCTGAAGG CCCTGGTGGA GTTCATGTAC AAGGGGGAGA

D. sechellia 801 CAGCTGGTCC GACCTGAAGG CCCTGGTGGA GTTCATGTAC AAGGGGGAGA

Genome 851 TCAACGTCTG CCAGGATCAG ATAAACCCCC TGCTCAAAGT GGCCGAAACC

Light P1 851 TCAACGTCTG TCAGGATCAG ATAAATCCCC TGCTCAAAGT GGCCGAAACC

Dark P1 851 TCAACGTCTG CCAGGATCAG ATAAACCCCC TGCTCAAAGT GGCCGAAACC

D. sechellia 851 TCAACGTCTG CCAGGATCAG ATAAACCCCC TGCTCAAAGT GGCCGAAACC

Genome 901 CTGAAGATCA GGGGTCTGGC GGAGGTCAGT GCGGGCAGGG GCGAGGGAGG

Light P1 901 CTGAAGATCA GGGGTCTGGC GGAGGTCAGT GCGGGCAGGG GCGAGGGAGG

Dark P1 901 CTGAAGATCA GGGGTCTGGC GGAGGTCAGT GCGGGCAGGG GCGAGGGAGG

D. sechellia 901 CTGAAGATCA GGGGTCTGGC GGAGGTCAGT GCGGGCAGGG GCGAGGGAGG

Genome 951 CGCCTCCGCA CTTCCCATGT CCGCCTTCGA CGATGAGGAC GAGGAGGAGG

Light P1 951 CGCCTCCGCA CTTCCCATGT CCGCCTTCGA CGATGAGGAC GAGGAGGAGG

Dark P1 951 CGCCTCCGCA CTTCCCATGT CCGCCTTCGA CGATGAGGAC GAGGAAGAGG

D. sechellia 951 CGCCTCCGCA CTTCCCATGT CCGCCTTCGA CGATGAGGAC GAGGAGGAGG

Genome 1001 AACTGGCCTC GGCCACTGCA ATTCTGCAGC AGGACGGTGA TGCCGATCCC

Light P1 1001 AACTGGCCTC GGCCACTGCA ATTCTGCGGC AGGACGGTGA CGCCGATCCC

Dark P1 1001 AACTGGCCTC GGCCACTGCA ATTCTGCAGC AGGACGGTGA TGCCGATCCC

D. sechellia 1001 AACTGGCCTC GGCCACTGCT ATTCTGCGGC AGGAGGGTGA CGCCGATCCC

Genome 1051 GATGAGGAGA TGAAGGCCAA GAGGCCCAGA CTGCTGCCCG AGGGAGTCTT

Light P1 1051 GATGAGGAGA TGAAGGCCAA GAGGCCCAGA CTGCTGCCCG AGGGAGTTTT

Dark P1 1051 GATGAGGAGA TGAAGGCCAA GAGGCCCAGA CTGCTGCCCG AGGGAGTCTT

D. sechellia 1051 GACGAGGAGA TGAAGGCCAA GAGACCCAGA CTGCTGCCCG ATGGAGTCTT

Genome 1101 GGACTTGAAT CAGCGACAAA GGAAGCGGTC CAGGGATGGC AGCTACGCCA

Light P1 1101 GGACTTGAAT CAGCGACAAA GAAAGCGGTC CAGGGATGGC AGCTACGCCA

Dark P1 1101 GGACTTGAAT CAGCGACAAA GGAAGCGGTC CAGGGATGGC AGCTACGCCA

D. sechellia 1101 GGACTTGAAT CAGCGACAAA GGAAGCGGTC CAGGGATGGC AGCTACGCCA

Genome 1151 CTCCAAGTCC ATCCCTTCAG GGCGGAGAGT CCGAGATCTC GGAGAGGGGC

Light P1 1151 CTCCGAGTCC ATCCCTCCAC GGCGGATAGT CCGAGATCTC GGACAGGGGC

Dark P1 1151 CTCCAAGTCC ATCCCTTCAG GGCGGAGAGT CCGAGATCTC GGAGAGGGGC

D. sechellia 1151 CTCCGAGTCC CTCCCTCCAG GGCGGTGAGT CCGAGATCTC GGAGAGGGGC

Genome 1201 TCATCC-GGC ACTCCGGGAC AGAGCCAG-- AGCCAACCCC TGGCCATGAC

Light P1 1201 TCATCC-GGC ACTCCGGGAC AGAGCCAG-- AGCCAACCTC TGGCCATGAC

Dark P1 1201 TCATCC-GGC ACTCCGGGAC AGAGCCAG-- AGCCAACCCC TGGCCATGAC

D. sechellia 1201 TCATCC-GGC ACTCCTGGAC AGAACCAG-- AGCCAGCCCC TGGCCATGAC

Genome 1248 CA--CCTCCA CCATTGTGCG CAATCCCTTC GCCTCCCCCA ATCCTCAGAC

Light P1 1248 CA--CCTCCA CCATAGTGCG TAATCCATTC GCCTCCCCCA ATCCTCAGAC

Dark P1 1248 CA--CCTCCA CCATTGTGCG CAATCCCTTC GCCTCCCCCA ATCCTCAGAC

D. sechellia 1248 CA--CCTCCA CCATAGTGCG CAATCCCTTC GCCTCGCCCA ATCCCCAGAC

Genome 1296 CTTGGAGGGC AGGAACAGCG CCATGAATGC AGTAGCAAAC -CAGAGGAAA

Light P1 1296 CTTGGAGGGC AGGAACAGCG CAATGAATGC AGTAGCAAAC -CAGAGGAAA

Dark P1 1296 CTTGGAGGGC AGGAACAGCG CCATGAATGC AGTAGCAAAC -CAGAGGAAA

D. sechellia 1296 CTTGGATGGC AGGAACAGCG CCTTGAATGC AGCAGCAAGC -CAGAGGAAA

Genome 1345 TCACCAGCAC CAACAGCGAC AGGTCACAGC AATGGGAACA GCGGCGCCGC

Light P1 1345 TCACCAGCAC CAACAGCGAC AGGTCACAGC AATGGGAACA GCGGCGCCGC

Dark P1 1345 TCACCAGCAC CAACAGCGAC AGGTCACAGC AATGGGAACA GCGGCGCCGC

D. sechellia 1345 TCACCAGCAC CAACAGCG-- -GGTCACAGC AATGGAAACA GCGGCGCCGC

Genome 1395 CATGCACTCC CCACCCGGGG GCGTGGCCGT CCAGTCCGCC CTTCCGCCCC

Light P1 1395 CATGCACTCC CCACCCGGGG GCGTGGCCGT CCAGTCCGCC CTTCCGCCCC

Dark P1 1395 CATGCACTCC CCACCCGGGG GCGTGGCCGT CCAGTCCGCC CTTCCGCCCC

D. sechellia 1392 CATGCACTCC CCACCCGGGG GCGTGGCCGT GCAGTCCGCC CTGCCGCCCC

Genome 1445 ACATGGCCGC CATCGTGCCG CCACCCCCCT CCGCCATGCA CCATCATGCC

Light P1 1445 ACATGGCCGC AATCGTGCCG CCACCCCCTT CCGCCATGCA CCATCATGCC

Dark P1 1445 ACATGGCCGC CATCGTGCCG CCACCCCCTT CCGCCATGCA CCATCATGCC

D. sechellia 1442 ACATGGCCGC CATCGTGCCC CCACCCCAAT CCGCCATGCA CCACCATGCC

Genome 1495 CAGCAACTGG CCGCCCAGCA CCAGCTGGCC CACTCGCACG CCATGGCCAG

Light P1 1495 CAGCAACTGG CCGCCCAGCA CCAGCTGGCC CACTCGCACG CCATGGCCAG

Dark P1 1495 CAGCAACTGG CCGCCCAGCA CCAGCTGGCC CACTCGCACG CCATGGCCAG

D. sechellia 1492 CAGCAACTGG CCGCCCAGCA CCAGCTGGCC CACTCGCACG CCATGGCCAG

Genome 1545 CGCCTTGGCA GCCGCAGCCG CCGGAGCTGG CGCAGCGGGA GCGGGCGGAG

Light P1 1545 CGCCTTGGCA GCCGCAGCCG CCGGAGCTGG CGCAGCGGGA GCGGGCGGAG

Dark P1 1545 CGCCTTGGCA GCCGCAGCCG CCGGAGCTGG CGCAGCGGGA GCGGGCGGAG

D. sechellia 1542 CGCCTTGGCA GCCGCAGCCG CCGGAGCGGG CGCAGCAGGA GCGGGCGGAG

Genome 1595 CAGGATCTGG CAGTGGATCG GGCGCCAGTG CTCCGACTGG AGGAACAGGA

Light P1 1595 CAGGATCTGG CAGTGGATCG GGCGCCAGTG CTCCGACTGG AGGAACAGGA

Dark P1 1595 CAGGATCTGG CAGTGGATCG GGCGCCAGTG CTCCGACTGG AGGAACAGGA

D. sechellia 1592 CAGGATCCGG CAGTGGATCG GGCGCCAGTG CTCCGACTGG AGGAACGGGA

Genome 1645 GTGGCGGGAA GTGGAGCCGG CGCGGCGGTG GGATCCCATC ACGATGACAT

Light P1 1645 GTGGCGGGAA GTGGAGCCGG CGCGGCGGTG GGATCTCATC ACGATGACAT

Dark P1 1645 GTGGCGGGAA GTGGAGCCGG CGCGGCGGTG GGATCCCATC ACGATGACAT

D. sechellia 1642 GTGGCGGGTA GTGGAGCCGG CGCGGCGGTG GGATCCCATC ACGATGACAT

Genome 1695 GGAGATCAAG CCAGAAATCG CCGAGATGAT ACGCGAAGAA GAGAGGGTGA

Light P1 1695 GGAGATCAAG CCAGAAATCG CCGAGATGAT ACGCGAAGAA GAGAGGGTGA

Dark P1 1695 GGAGATCAAG CCAGAAATCG CCGAGATGAT ACGCGAAGAA GAGAGGGTGA

D. sechellia 1692 GGAGATCAAG CCAGAAATCG CAGAGATGAT TCGCGAAGAG GAGAGGGTGA

Genome 1745 GT-------- ---------- ---------- ---------- ----------

Light P1 1745 GT-------- ---------- ---------- ---------- ----------

Dark P1 1745 GT-------- ---------- ---------- ---------- ----------

D. sechellia 1742 GT-------- ---------- ---------- ---------- ----------

**bab2 3rd exon**

Genome 1 CAGGCCAAGA TGATCGAGAG TGGAGGCCAC GGTGGATGGA TGGGAGCGGC

Light P1 1 CAGGCCAAGA TGATCGAGAG TGGAGGCCAC GGTGGATGGA TGGGAGCGGC

Dark P1 1 CAGGCCAAGA TGATCGAGAG TGGAGGCCAC GGTGGATGGA TGGGAGCGGC

D. sechellia 1 CAGGCCAAGA TGATCGAGAG TGGAGGCCAC GGTGGCTGGA TGGGAGCTGC

Genome 51 AGCTGCGGCA ACTGGAGCAG CTTCTGTGGC GGGTAAGT

Light P1 51 AGCTGCGGCA ACTGGAGCAG CTTCTGTGGC GGGTAAGT

Dark P1 51 AGCTGCGGCA ACTGGAGCAG CTTCTGTGGC GGGTAAGT

D. sechellia 51 AGCTGCGGCA ACTGGAGCAG CTTCTGTGGC GGGTAAGT

**bab2 4th exon**

Genome 1 CAGCAGATAG CTACCAGTAC CAGCTACAGT CCATGTGGCA GAAGTGCTGG

Light P1 1 CAGCAGATAG CTACCAGTAC CAGCTACAGT CCATGTGGCA GAAGTGCTGG

Dark P1 1 CAGCAGATAG CTACCAGTAC CAGCTACAGT CCATGTGGCA GAAGTGCTGG

D. sechellia 1 CAGCAGATAG CTACCAGTAC CAGCTGCAGT CCATGTGGCA GAAGTGCTGG

Genome 51 AACACCAATC AGCAGAACCT GGTGCAGCAG CTCAGATTCC GCGAGCGCGG

Light P1 51 AACACCAATC AGCAGAACCT GGTGCAGCAG CTCAGATTCC GCGAGCGCGG

Dark P1 51 AACACCAATC AGCAGAACCT GGTGCAGCAG CTCAGATTCC GCGAGCGCGG

D. sechellia 51 AACACCAACC AGCAGAACCT GGTGCAGCAG CTCAGGTTCC GCGAGCGCGG

Genome 101 CCCATTGAAG TCCTGGCGAC CCGAGGCCAT GGCCGAGGCC ATTTTCAGTG

Light P1 101 CCCATTGAAG TCCTGGCGAC CCGAGGCCAT GGCCGAGGCC ATCTTCAGTG

Dark P1 101 CCCATTGAAG TCCTGGCGAC CCGAGGCCAT GGCCGAGGCC ATTTTCAGTG

D. sechellia 101 CCCACTGAAG TCCTGGCGAC CCGAGGCCAT GGCCGAGGCC ATCTTCAGTG

Genome 151 TCCTGAAGGA GGGGCTCTCC CTGTCACAGG CTGCCCGCAA GTTCGACATA

Light P1 151 TCCTGAAGGA GGGGCTCTCC CTGTCGCAGG CTGCCCGAAA GTTTGACATA

Dark P1 151 TCCTGAAGGA GGGGCTCTCC CTGTCACAGG CTGCCCGCAA GTTCGACATA

D. sechellia 151 TCCTGAAGGA GGGGCTCTCC CTGTCGCAGG CTGCCCGCAA GTTCGACATC

Genome 201 CCCTATCCCA CCTTCGTCCT GTACGCCAAT CGGGTGCACA ACATGCTGGG

Light P1 201 CCCTATCCCA CCTTCGTCCT GTACGCCAAT CGGGTGCACA ACATGCTGGG

Dark P1 201 CCCTATCCCA CCTTCGTCCT GTACGCCAAT CGGGTGCACA ACATGCTGGG

D. sechellia 201 CCCTATCCCA CCTTCGTCCT GTACGCCAAT CGGGTGCACA ACATGCTGGG

Genome 251 ACCCTCGCTG GATGGCGGAG CTGATCCGCG GCCAAAGGCA CGCGGTCGTC

Light P1 251 ACCCTCGCTG GATGGCGGAG CTGATCCGCG GCCAAAGGCA CGCGGTCGTC

Dark P1 251 ACCCTCGCTG GATGGCGGAG CTGATCCGCG GCCAAAGGCA CGCGGTCGTC

D. sechellia 251 ACCCTCGCTG GATGGCGGAG CTGATCCGCG GCCAAAGGCA CGCGGTCGTC

Genome 301 CCCAGAGGAT CCTGCTGGGC ATGTGGCCGG AGGAGCTCAT CCGTAGCGTC

Light P1 301 CCCAGAGGAT CCTGCTGGGC ATGTGGCCGG AGGAGCTCAT CCGTAGCGTC

Dark P1 301 CCCAGAGGAT CCTGCTGGGC ATGTGGCCGG AGGAGCTCAT CCGTAGCGTC

D. sechellia 301 CCCAGAGGAT CCTGTTGGGC ATGTGGCCGG AGGAGCTCAT CCGCAGCGTC

Genome 351 ATTAAGGCCG TGGTGTTCCG GGACTATCGC GAGATTAAGG AGGACATGAG

Light P1 351 ATTAAGGCCG TGGTGTTCCG GGACTATCGC GAGATTAAGG AGGACATGAG

Dark P1 351 ATTAAGGCCG TGGTGTTCCG GGACTATCGC GAGATTAAGG AGGACATGAG

D. sechellia 351 ATCAAGGCCG TGGTGTTCCG GGATTATCGC GAGATCAAGG AGGACATGAG

Genome 401 CGCCCATCAG TACGCCAATG GACAGGGTCA TGGTGTAAGT

LightP1 401 CGCCCATCAG TACGCCAATG GACAGGGTCA TGGTGTAAGT

DarkP1 401 CGCCCATCAG TACGCCAATG GACAGGGTCA TGGTGTAAGT

D.sechellia 401 TGCCCACCAG TACGCCAATG GACAGGGCCA TGGTGTAAGT

**bab2 5th exon**

Genome 1 CAGACCTATA TCGGAGGAGG AACCACCACG AATGGCTACC ACAGTGCTGC

Light P1 1 CAGACCTATA TCGGAGGAGG AACCACCACG AATGGCTACC ACAGTGCTGC

Dark P1 1 CAGACCTATA TCGGAGGAGG AACCACCACG AATGGCTACC ACAGTGCTGC

D. sechellia 1 CAGACATATA TCGGAGGAGG CACCACCACG AATGGCTACC ACAGTGCTGC

Genome 51 CGCAGCCAAG CTGGCGGCTC AGAACGCTGC ACTGGCTCCG CCGGACGCAG

Light P1 51 CGCAGCCAAG CTGGCGGCTC AGAACGCTGC ACTGGCTCCG CCGGACGCAG

Dark P1 51 CGCAGCCAAG CTGGCGGCTC AGAACGCTGC ACTGGCTCCG CCGGACGCAG

D. sechellia 51 GGCAGCCAAG CTGGCGGCCC AGAATGCTGC ACTGGCTCCG CCGGACGCAG

Genome 101 GAAGTCCGCT GAGCTCCATG ACGGAAACCC TTCGCCGCCA GATCCTCTCG

Light P1 101 GAAGTCCGCT GAGCTCCATG ACGGAAACCC TTCGCCGCCA GATCCTCTCG

Dark P1 101 GAAGTCCGCT GAGCTCCATG ACGGAAACCC TTCGCCGCCA GATCCTCTCG

D. sechellia 101 GAAGTCCGCT GAGCTCAATG ACGGAGACCC TGCGCCGCCA GATCCTCTCG

Genome 151 CAGCAGCAGC AACATCAGCA GCACCACCAG CAGCAGGCAC ACCATCAGCA

Light P1 151 CAGCAGCAGC AACATCAGCA GCACCACCAG CAGCAGGCAC ACCATCAGCA

Dark P1 151 CAGCAGCAGC AACATCAGCA GCACCACCAG CAGCAGGCAC ACCATCAGCA

D. sechellia 151 CAGCAGCAGC AACATCAGCA GCACCATCAG CAGCAGGCGC ACCACCAGCA

Genome 201 ACAGCCCTCG CACCACCAGC AACAGTCGCC CCACGCCCAG TCCATGAACA

Light P1 201 ACAGCCCTCG CACCACCAGC AACAGTCGCC CCACGCCCAG TCCATGAACA

Dark P1 201 ACAGCCCTCG CACCACCAGC AACAGTCGCC CCACGCCCAG TCCATGAACA

D. sechellia 201 GCAGCCCTCG CACCACCAGC AGCAGTCGCC CCACGCCCAG TCCATGAACA

Genome 251 TGTACAAGTC CCCGGCCTAT CTGCAGCGAT CCGAGATCGA AGATCAAGTA

Light P1 251 TGTACAAGTC CCCGGCCTAT CTGCAGCGAT CCGAGATCGA AGATCAAGTA

Dark P1 251 TGTACAAGTC CCCGGCCTAT CTGCAGCGAT CCGAGATCGA AGATCAAGTA

D. sechellia 251 TGTACAAGTC CCCGGCCTAT CTGCAGCGAT CCGAGATCGA AGATCAAGTA

Genome 301 TCCGCAGCGG CGGCCGTGGC AGCGGCGGCG GCCAAGCACC AGCAGCAGCA

Light P1 301 TCCGCAGCGG CGGCCGTGGC AGCGGCGGCG GCCAAGCACC AGCAGCAGCA

Dark P1 301 TCCGCAGCGG CGGCCGTGGC AGCGGCGGCG GCCAAGCACC AGCAGCAGCA

D. sechellia 301 TCCGCAGCGG CGGCCGTGGC AGCAGCGGCG GCCAAGCACC AGCAGCAGCA

Genome 351 GGGTGAGCGA AGGGGTTCGG AGAACCTGCC CGACCTCAGT GCCCTGGGCC

Light P1 351 GGGTGAGCGA AGGGGTTCGG AGAACCTGCC CGACCTCAGT GCCCTGGGCC

Dark P1 351 GGGTGAGCGA AGGGGTTCGG AGAACCTGCC CGACCTCAGT GCCCTGGGCC

D. sechellia 351 GGGTGAGCGA AGGGGTTCGG AGAACCTGCC CGACCTCAGT GCCCTGGGCC

Genome 401 TGATGGGTCT GCCCGGCCTG AATGTGATGC CCTCACGGGG ATCGGGTGGA

Light P1 401 TGATGGGTCT GCCCGGCCTG AATGTGATGC CCTCACGGGG ATCGGGTGGA

Dark P1 401 TGATGGGTCT GCCCGGCCTG AATGTGATGC CCTCACGGGG ATCGGGTGGA

D. sechellia 401 TGATGGGTCT GCCGGGGCTG AACGTGATGC CCTCGCGGGG ATCGGGTGGA

Genome 451 GGAAGTGGTG GCGCAGCGCC GAATAGTGCC GCCTCCTATG CCCGCGAGTT

Light P1 451 GGAAGTGGTG GCGCAGCGCC GAATAGTGCC GCCTCCTATG CCCGCGAGTT

Dark P1 451 GGAAGTGGTG GCGCAGCGCC GAATAGTGCC GCCTCCTATG CCCGCGAGTT

D. sechellia 451 GGAAGTGGTG GCGCAGCTCC GAACAGTGCC GCCTCCTATG CCCGCGAGTT

Genome 501 ATCCCGCGAA AGGGAACGCG ATCGGGAGCG CGAAAGGGAG CGGGAGCTGT

Light P1 501 ATCCCGCGAA AGGGAACGCG ATCGGGAGCG CGAAAGGGAG CGGGAGCTGT

Dark P1 501 ATCCCGCGAA AGGGAACGCG ATCGGGAGCG CGAAAGGGAG CGGGAGCTGT

D. sechellia 501 GTCCCGCGAA AGGGAGCGCG ATCGGGAGCG CGAGAGGGAG AGGGAGCTGT

Genome 551 CCCGCCAGTA TGGCAGCCAG TCGCGGGGAT CGAGCTCCGG TTCCGGAAGC

Light P1 551 CCCGCCAGTA TGGCAGCCAG TCGCGGGGAT CGAGCTCCGG TTCCGGAAGC

Dark P1 551 CCCGCCAGTA TGGCAGCCAG TCGCGGGGAT CGAGCTCCGG TTCCGGAAGC

D. sechellia 551 CGCGCCAGTA TGGTAGCCAG TCGCGGGGAT CGAGCTCCGG TTCCGGAAGC

Genome 601 GCCAAGTCCC TGACCGCCAG CCAAAGACCA GGAGCCGCCT CGCCGTACTC

Light P1 601 GCCAAGTCCC TGACCGCCAG CCAAAGACCA GGAGCCGCCT CGCCGTACTC

Dark P1 601 GCCAAGTCCC TGACCGCCAG CCAAAGACCA GGAGCCGCCT CGCCGTACTC

D. sechellia 601 GCCAAGTCCC TGACCGCCAG CCAAAGACCA GGAGCCGCCT CGCCGTACTC

Genome 651 CGCCGCCCAC TATGCCAAAC ATCAGGCGAG TGCCTACAAC AAGAGGTTTC

Light P1 651 CGCCGCCCAC TATGCCAAAC ATCAGGCGAG TGCCTACAAC AAGAGGTTTC

Dark P1 651 CGCCGCCCAC TATGCCAAAC ATCAGGCGAG TGCCTACAAC AAGAGGTTTC

D. sechellia 651 CGCCGCCCAC TATGCCAAAC ATCAGGCGAG CGCCTACAAC AAGAGGTTTC

Genome 701 TCGAGAGCCT GCCCGCCGGC ATTGACTTGG AGGCCTTCGC CAACGGACTG

Light P1 701 TCGAGAGCCT GCCCGCCGGC ATTGACTTGG AGGCCTTCGC CAACGGACTG

Dark P1 701 TCGAGAGCCT GCCCGCCGGC ATTGACTTGG AGGCCTTCGC CAACGGACTG

D. sechellia 701 TCGAGAGCCT GCCCGCCGGC ATCGACTTGG AGGCCTTCGC CAACGGGCTG

Genome 751 CTCCAGAAGT CGGTGAACAA GAGTCCGCGC TTCGAGGACT TCTTCCCGGG

Light P1 751 CTCCAGAAGT CGGTGAACAA GAGTCCGCGC TTCGAGGACT TCTTCCCGGG

Dark P1 751 CTCCAGAAGT CGGTGAACAA GAGTCCGCGC TTCGAGGACT TCTTCCCGGG

D. sechellia 751 CTCCAGAAGT CGGTGAACAA GAGTCCGCGC TTCGAGGACT TCTTCCCGGG

Genome 801 ACCCGGCCAG GACATGAGTG AACTGTTTGC CAATCCGGAC GCGAGTGCAG

Light P1 801 ACCCGGCCAG GACATGAGTG AACTGTTTGC CAATCCGGAC GCGAGTGCAG

Dark P1 801 ACCCGGCCAG GACATGAGTG AACTGTTTGC CAATCCGGAC GCGAGTGCAG

D. sechellia 801 ACCCGGCCAG GACATGAGTG AACTGTTTGC CAATCCGGAC GCGAGTGCAG

Genome 851 CTGCCGCGGC GGCCGCCTAC GCGCCTCCTG GCGCCATCCG CGAATCGCCT

Light P1 851 CTGCCGCGGC GGCCGCCTAC GCGCCTCCTG GCGCCATCCG CGAATCGCCT

Dark P1 851 CTGCCGCGGC GGCCGCCTAC GCGCCTCCTG GCGCCATCCG CGAATCGCCT

D. sechellia 851 CTGCCGCGGC GGCCGCCTAT GCGCCTCCAG GCGCCATCCG GGAATCGCCT

Genome 901 CTGATGAAGA TCAAGCTGGA GCAGCAGCAT GCCACCGAAC TGCCGCACGA

Light P1 901 CTGATGAAGA TCAAGCTGGA GCAGCAGCAT GCCACCGAAC TGCCGCACGA

Dark P1 901 CTGATGAAGA TCAAGCTGGA GCAGCAGCAT GCCACCGAAC TGCCGCACGA

D. sechellia 901 CTGATGAAGA TCAAGCTGGA GCAGCAGCAT GCCACCGAAC TGCCGCACGA

Genome 951 GGATTGA

Light P1 951 GGATTGA

Dark P1 951 GGATTGA

D. sechellia 951 GGATTGA

**Splice junctions are shown with the gray background color.**
