# Supplementary material for: Recurrent Modification of a Conserved Cis-Regulatory Element Underlies Fruit Fly Pigmentation Diversity
Source: PLoS Genet. 2013 Aug 29;9(8):e1003740. doi: 10.1371/journal.pgen.1003740 (PMC3757066; doi:10.1371/journal.pgen.1003740)
Supplement: Table S1 — Association between pigmentation phenotype and bab dimorphic element genotype. (DOC) [file pgen.1003740.s007.doc]

**Table S1.** Association between pigmentation phenotype and *bab* dimorphic element genotype.

|  | Observed Genotype Counts, n (%) | | | | Expected Genotype Counts | | | |  |
| --- | --- | --- | --- | --- | --- | --- | --- | --- | --- |
| **Phenotype** | ***L1L1*** | ***L1D1*** | ***D1D1*** | **Total** | ***L1L1*** | ***L1D1*** | ***D1D1*** | **Total** | ***P*** |
| Light | 16 | 0 | 0 | 16 | 4 | 8 | 4 | 16 | <0.00001 |
| Intermediate | 0 | 11 | 0 | 11 | 2.75 | 5.5 | 2.75 | 11 | 0.0041 |
| Dark | 0 | 0 | 16 | 16 | 4 | 8 | 4 | 16 | <0.00001 |
| Total | 16 | 11 | 16 | 43 | 10.75 | 21.5 | 10.75 | 43 |  |

*L1L1* and *D1D1* respectively indicate individuals homozygous for the Light 1 and Dark population dimorphic element alleles and *L1D1* indicates heterozygotes.

In each row of the contingency table, the p-value was derived using the Chi-Square test.
