# Supplementary material for: Recurrent Modification of a Conserved Cis-Regulatory Element Underlies Fruit Fly Pigmentation Diversity
Source: PLoS Genet. 2013 Aug 29;9(8):e1003740. doi: 10.1371/journal.pgen.1003740 (PMC3757066; doi:10.1371/journal.pgen.1003740)
Supplement: Table S2 — Association between pigmentation phenotype and bab dimorphic element genotype. (DOC) [file pgen.1003740.s008.doc]

**Table S2.** Association between pigmentation phenotype and *bab* dimorphic element genotype.

|  | Observed Genotype Counts, n (%) | | | | Expected Genotype Counts | | | |  |
| --- | --- | --- | --- | --- | --- | --- | --- | --- | --- |
| **Phenotype** | ***L1L1*** | ***L1D2*** | ***D2D2*** | **Total** | ***L1L1*** | ***L1D2*** | ***D2D2*** | **Total** | ***P*** |
| Light | 5 | 0 | 0 | 5 | 1.25 | 2.5 | 1.25 | 5 | <0.001 |
| Dark | 0 | 3 | 14 | 17 | 4.25 | 8.5 | 4.25 | 17 | <0.00001 |
| Total | 5 | 3 | 14 | 22 | 5.5 | 11 | 5.5 | 22 |  |

*L1L1* and *D2D2* respectively indicate individuals homozygous for the Light 1 and Dark 2 population dimorphic element alleles and *L1D2* indicates heterozygotes.

In each row of the contingency table the p-value was derived using the Chi-Square test.
