# Supplementary material for: Recurrent Modification of a Conserved Cis-Regulatory Element Underlies Fruit Fly Pigmentation Diversity
Source: PLoS Genet. 2013 Aug 29;9(8):e1003740. doi: 10.1371/journal.pgen.1003740 (PMC3757066; doi:10.1371/journal.pgen.1003740)
Supplement: Table S3 — Primers used to PCR amplify D. melanogaster bab protein coding exons and their splice junctions. (DOC) [file pgen.1003740.s009.doc]

**Table S3.** Primers used to PCR amplify *D. melanogaster* *bab* protein coding exons and their splice junctions.

| **Gene** | **Exon** | **PCR Size** | **Primer Sequence** | **Primer Name** |
| --- | --- | --- | --- | --- |
| *bab1* | 1 | 1192 | CTTCTCTTAACTGGCATATGTACTAAC | bab1 exon 1 Fwd |
| *bab1* | 1 |  | AGCATCCCATTGTGATCATG | bab1 exon 1 seq 2 |
| *bab1* | 1 | 983 | CGCAATCAGGCTGCTCAGTG | bab1 exon 1 seq 1 |
| *bab1* | 1 |  | GGTGATCCAACGATCAACGATCAACG | bab1 exon 1 Rvs |
| *bab1* | 2 | 211 bp | CAGCTGGTACTGGTAGCTGTCTG | bab1 genotype 1A |
| *bab1* | 2 |  | AGCTTTCTTCTCTTGCCTCACTTTGC | bab1 exon 2,3 Rvs |
| *bab1* | 3 | 527 bp | GAATTTGGCAAAGTCCGCTAAGCG | bab1 exon 3 Fwd 2 |
| *bab1* | 3 |  | GGCAGCTGGAAACCAACTGATCG | bab1 exon 3 Rvs 2 |
| *bab1* | 4 | 746 bp | AGGCGTAGGCTTACACAGGCCAAG | bab1 exon 4 Fwd |
| *bab1* | 4 |  | TGGAGGCAGGGCCAGCATATC | bab1 exon 4 seq 2 |
| *bab1* | 4 | 676 bp | TGCGCAACGGACTGCTCTTGG | bab1 exon 4 seq 1 |
| *bab1* | 4 |  | GGGATTTCTTGAACCGAACTAACTGC | bab1 exon 4 Rvs |
| *bab2* | 2 | 1291 bp | GCTGGAAAAATCGCTATGCCTCAG | bab2 exon 2 Fwd |
| *bab2* | 2 |  | CAAGGCACACAAGATGGTGC | bab2 exon 2 seq 2 |
| *bab2* | 2 | 1138 bp | GCAGCAGTCTGGGCCTCTTG | bab2 exon 2 seq 1 |
| *bab2* | 2 |  | CGCAGAAAACCTGCAAGACAACCG | bab2 exon 2 Rvs |
| *bab2* | 3 & 4 | 646 bp | AGTCTTCCAAGGAAATTTGCAAC | bab2 exon 3,4 Fwd2 |
| *bab2* | 3 & 4 |  | TGCAAGTTCCTGCACAGTGAAC | bab2 exon 3,4 Rvs2 |
| *bab2* | 5 | 1036 bp | CAGCGTGTGTCCCAATGCCAC | bab2 exon 5 Fwd |
| *bab2* | 5 |  | TTCTAACCCACTATTTCGCCCTC | bab2 exon 5 Rvs |
