# Supplementary material for: Recurrent Modification of a Conserved Cis-Regulatory Element Underlies Fruit Fly Pigmentation Diversity
Source: PLoS Genet. 2013 Aug 29;9(8):e1003740. doi: 10.1371/journal.pgen.1003740 (PMC3757066; doi:10.1371/journal.pgen.1003740)
Supplement: Table S4 — Primers used for PCR-based genotyping of the D. melanogaster bab locus. (DOC) [file pgen.1003740.s010.doc]

**Table S4.** Primers used for PCR-based genotyping of the *D. melanogaster* *bab* locus.

| **#** | **Description** | **Size (bp)** | **Primer Sequence** | **Primer Name** |
| --- | --- | --- | --- | --- |
| 1 | Upstream of *bab2*  indel (*Trh* intron 3) | Light P1 = 119  Dark P1 = ~140 | GAGCTCCAAGAAAACGGTGCC | Trh intron 3 Fwd |
|  |  |  | ACCTGAGGAGGTGAAAACCTG | Trh intron 3 Rvs |
| 2 | *bab1* & *bab2*  intergenic region indel | Light P1 = 128  Dark P1 = 107 | CTCGGGTTCCTCGCTTGTC | TREindelFwd |
|  |  |  | GCCCCAACACATCCCAGACTG | TREindelRvs |
| 3 | *bab1* intron (in  dimorphic element) RFLP | 381* | ATGCTTAGATTTGCTCCAGCAGTGG | BstXI Fwd 1 |
|  |  |  | GAGTGGCTGTATAACTATTGCAC | BstXI Rvs 1 |
| 4 | *bab1* intron 3 indel | Light P1 = ~600  Dark P1 = ~200 | CTAACCCGAGAGCAGTTAGTTCG | bab1 intron 3 Fwd 2 |
|  |  |  | GACATCAAGGACGAGAGCCTGG | bab1 intron 3 Rvs 3 |

*Light P1 has a *BstXI* restriction enzyme site where the PCR product is cleaved into a 232 and 149 base pair fragment by BstXI. Both Dark P1 and Dark P2 alleles lack this site.
