# Supplementary material for: Recurrent Modification of a Conserved Cis-Regulatory Element Underlies Fruit Fly Pigmentation Diversity
Source: PLoS Genet. 2013 Aug 29;9(8):e1003740. doi: 10.1371/journal.pgen.1003740 (PMC3757066; doi:10.1371/journal.pgen.1003740)
Supplement: Table S5 — Primer combinations used to amplify and clone dimorphic element alleles and orthologous sequences. (DOC) [file pgen.1003740.s011.doc]

**Table S5.** Primer combinations used to amplify and clone dimorphic element alleles and orthologous sequences.

| **Range** | **Sequence** | **Name** | **Site** |
| --- | --- | --- | --- |
| Sophophora^a^ | ggcgcgccCACATAAAAATCAGCAACAAASTTGC | sub1orthoF1 | AscI |
|  | cctgcaggCAAAACKGCRCATAAAAMSAAATTACA | dimorphic Rvs1 | SbfI |

**^a^**This primer combination has proven capable to amplify dimorphic element sequences from species representing the diverse lineages of the Sophophora subgenus.

**Note:** restriction enzyme sequences for cloning CRE sequences are indicated by lower case letters at the 5’ end of the primers.
