# Supplementary material for: Recurrent Modification of a Conserved Cis-Regulatory Element Underlies Fruit Fly Pigmentation Diversity
Source: PLoS Genet. 2013 Aug 29;9(8):e1003740. doi: 10.1371/journal.pgen.1003740 (PMC3757066; doi:10.1371/journal.pgen.1003740)
Supplement: Table S6 — Oligonucleotides used to make gel shift assay binding sites. (DOC) [file pgen.1003740.s012.doc]

**Table S6.** Oligonucleotides used to make gel shift assay binding sites.

| **Binding Site** | **Sequence (5’ to 3’)** | **Name** |
| --- | --- | --- |
| Dsx 1 Concestor | TTTGGCCGCAACAATGTTGCTGCATTTA | Dsx1 con Top |
|  | TAAATGCAGCAACATTGTTGCGGCCAAA | Dsx1 con Bottom |
| Dsx1 E mutant | CGGTCTGACAACAATGTTGCTGCATTTA | Dsx1 delta E Top |
|  | TAAATGCAGCAACATTGTTGTCAGACCG | Dsx1 delta E Bottom |
| Dsx 1 KO | TTTGGCCGCAGGGGGCGTGCTGCATTTA | Dsx1 KO Top |
|  | TAAATGCAGCACGCCCCCTGCGGCCAAA | Dsx1 KO Bottom |
